# Reductive rearrangement of substituted quinolines to 2,3-disubstituted indoles enabled by water activation

**DOI:** 10.1039/d5sc08793g

**Published:** 2025-12-22

**Authors:** Nico Spreckelmeyer, Jieun Kim, Jessika Lammert, Elena Sophia Horst, Jingjing Zhang, Armido Studer

**Affiliations:** a Organisch-Chemisches Institut, Universität Münster Corrensstraße 40 48149 Münster Germany studer@uni-muenster.de

## Abstract

Herein, we report a selective reductive rearrangement of substituted quinolines into indoles—a privileged structural motif widely found in natural products and bioactive molecules. This quinoline skeletal editing is accomplished through water activation mediated by photocatalytically generated phosphine radical cations. The developed protocol provides a robust and broadly applicable approach for synthesizing diverse indole derivatives from readily available quinoline substrates.

## Abstract 2

Reductive rearrangement of substituted quinolines to 2,3-disubstituted indoles enabled by photoredox catalyzed phosphine mediated water activation is reported.

## Graphical abstract

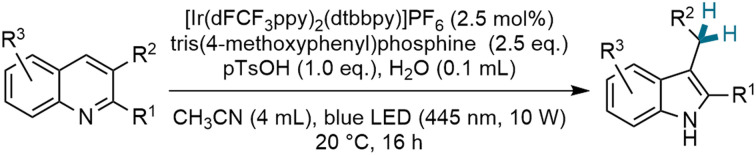

## Introduction

**Fig. 1:**
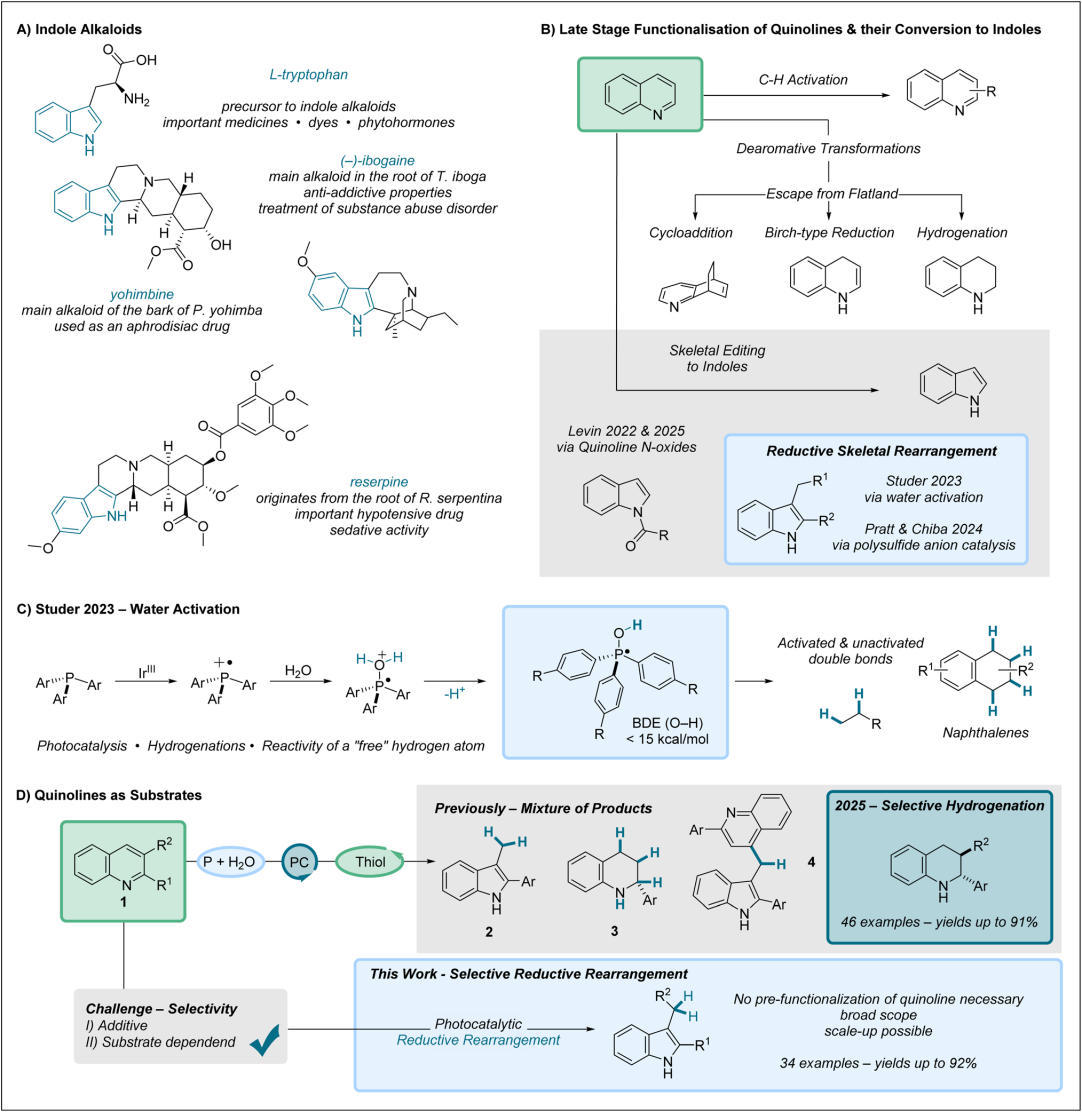
(A) Examples of indole alkaloids derived from l-tryptophan. (B) Late-stage functionalisation of quinolines and their conversion to indole scaffolds by skeletal editing. (C) Phosphine mediated water activation for hydrogenation. (D) Employing quinolines as substrates in the system leads to a mixture of products & further optimization towards selective reductive rearrangement.

N-heterocycles play a major role in drug discovery.^1–6^ Quinolines—and even more so indoles—demonstrate their significance through their prevalence in various developed drugs and naturally occurring bioactive molecules. Prominent examples include indole alkaloids naturally derived from l-tryptophan—such as reserpine, yohimbine, and members of the ibogaine family—which have found broad use in medicine (Fig. 1A).^7^

Late-stage functionalization of heterocyclic compounds—such as quinolines—plays a crucial role in rapidly expanding the chemical space of potential drug candidates (Fig. 1B, top). A wide range of reactions have been developed to expand the structural diversity within (hetero)aromatic scaffolds. Along these lines, C–H activation has emerged as a powerful strategy for the peripheral diversification of N-heterocycles, allowing access to an expanded spectrum of molecular structures.^8–11^ In this context, the *meta*-functionalisation of pyridines and quinolines has recently gained great attention.^12–14^ Furthermore, escaping the “flatland” of planar aromatic structures toward non-aromatic three-dimensional frameworks might be even more valuable. This can be achieved through various approaches, including hydrogenation and other dearomatization strategies.^15–19^ Hydrogenations can be carried out, for example, *via* transition-metal-catalyzed reductions with H_2_ and other approaches; however, challenges often arise in controlling both stereoselectivity and chemoselectivity.^20–30^ Arene hydrogenation can also proceed *via* radical intermediates, employing established methods such as the *Birch* reduction or using SmI_2_ as a reductant, among others.^31–43^ In recent years, photomediated dearomatization *via* triplet excitation, energy transfer (EnT) or single-electron transfer (SET) processes has attracted growing attention.^44,45^ Notably, beyond hydrogenation, photochemical approaches can also be employed to construct bi- and tricyclic structures through cycloadditions and coupling reactions.^46–53^

Incorporating skeletal editing steps into (hetero)arene hydrogenation cascades can increase the complexity of the resulting structures and further expand the accessible chemical space, as demonstrated herein for the reductive transformation of quinolines to indoles (Fig. 1B, bottom).^54–56^ Highlighting the different properties of these molecular scaffolds—quinolines are electron-poor aromatic systems with basic properties while indoles are rather electron rich. Quinolines can be accessed from indoles by reaction with carbenes through cyclopropanation followed by ring expansion, known as the Ciamician–Dennstedt reaction.^57–60^ Carbon-atom deletion in quinolines to access indoles can be accomplished by pre-functionalizing the nitrogen, for example *via* N-oxides or N-acylimides.^61–64^ In this context, Kaneko's groundbreaking photochemical rearrangement of quinoline N-oxides to N-acylindoles was subsequently advanced by Levin,^65^ which upon further exploration led to a general and selective direct carbon deletion at either the 2- or 3-position, yielding the corresponding indole products.^66^ This represents divergent skeletal editing, allowing access to multiple products from a single substrate, an area of increasing attention.^67,68^

Rather unexplored remains the strategy to selectively induce a reductive rearrangement of non-pre-functionalized quinolines to give indoles *via* controlled structural reorganization of the heteroarene scaffold. This was shown by our group through photocatalytic water activation and also in joint elegant efforts by Pratt and Chiba through polysulfide anion catalysis especially covering C4 substituted quinolines.^52,69^ In our work, we found that photocatalytically generated phosphine radical cations immediately react with water (Fig. 1C).^69^ Deprotonation of such adducts then provides highly reactive phosphoranyl radicals with a very low O–H bond dissociation energy (<15 kcal mol^−1^) enabling the reduction of alkenes as well as naphthalenes through intermolecular hydrogen atom transfer in combination with an arylthiol cocatalyst. Of note, deprotonation of such reactive HO–phosphoranyl radicals leads to phosphine oxide radical anions that can be employed as potent ground state single electron reductants (measured −3.1 V *vs.* SCE).^70^

Encouraged by our initial studies on the reductive rearrangement of quinolines to indoles we decided to investigate this highly valuable transformation in greater detail, as only moderate yields, limited selectivity, and a requirement for a 2-aryl substituent was reported in our first disclosure (Fig. 1D).^69^ The main challenges arise from the selective formation of the indole core 2 and accordingly the suppression of “simple” hydrogenation to give tetrahydroquinoline 3.^30^ Moreover, the formation of another undesired byproduct 4, which arises from rearrangement followed by addition to a second quinoline, must also be suppressed.^71^ Herein, we report reaction conditions that enable the selective formation of indoles from quinolines *via* reductive rearrangement using our water-activation system.

## Results and discussion

For reaction optimization, we selected the model substrate 1a. Extensive experimentation revealed that a quantitative yield of 2a (86% isolated) can be achieved for the reductive skeletal editing with phosphine P1 (P(*p*-MeOC_6_H_4_)_3_, 2.5 equiv.), the Ir-based photocatalyst PC ([Ir(dF(CF_3_)ppy)_2_(dtbbpy)]PF_6_, 2.5 mol%), in the presence of *p*-toluenesulfonic acid (pTsOH, 1 equiv.) in acetonitrile/water (40 : 1) upon blue LED irradiation for 24 hours at 20 °C (Table 1, entry 5). The tetrahydroquinoline 3a and the dimerization product 4a were not formed under these conditions. We later found that the isolated yield of 2a (92%) can be slightly further improved by shortening the reaction time to 16 hours, likely as a result of a slow decomposition of 2a under the applied condition (entry 10). Comparing these results with the previously reported “acid-free” conditions (entry 1), a far higher conversion (99% *versus* 28%) could be achieved and the yield of 2a could be significantly improved (92% isolated *versus* traces).^69^ Surprisingly, reaction with the HCl-salt as the substrate (1a-HCl) in the absence of pTsOH provided the indole 2a (68%) besides the tetrahydroquinoline 3a (15%), indicating that the counter anion of the quinoline salt somehow influences chemodivergency (entry 2). Repeating the experiment with the HCl-salt in the presence of an arylthiol cocatalyst (TripSH) further increased the yield of the tetrahydroquinoline byproduct 3a (30%, entry 3). This observation further indicates that the use of a HAT cocatalyst biases product selectivity in favor of the hydrogenation product. However, using the HCl-salt as the substrate in combination with phosphine P2 (XPhos), a high yield of the targeted rearrangement product 2a was achieved (96%, entry 4). For the screening of other phosphines, we refer to the SI. Since the preformation of the HCl-salt requires a precipitation step, the protocol using simple addition of pTsOH to the reaction mixture was considered more practical. Moreover, P2 is costlier than P1 and less general. Quinoline protonation is required, as with 0.5 equiv. of pTsOH, only a 53% conversion was achieved, revealing that the unprotonated quinoline does likely only show very little reactivity under these conditions (entry 6). Lowering the amount of phosphine also had a detrimental effect on the reaction outcome (entry 7) and the amount of water also slightly affected reactivity (entries 8 & 9). Control reactions gave negative results showing the necessity of all reagents (entries 11 & 12).

**Table 1:** Optimization of the reaction conditions

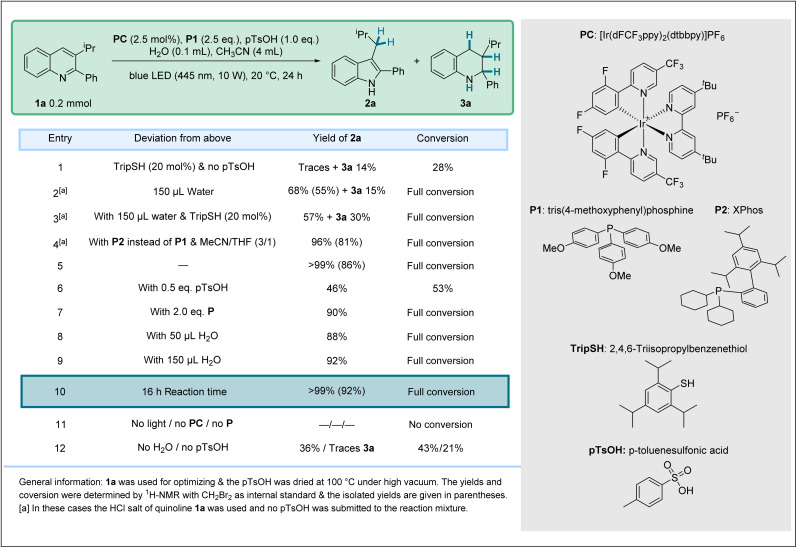

**Fig. 2:**
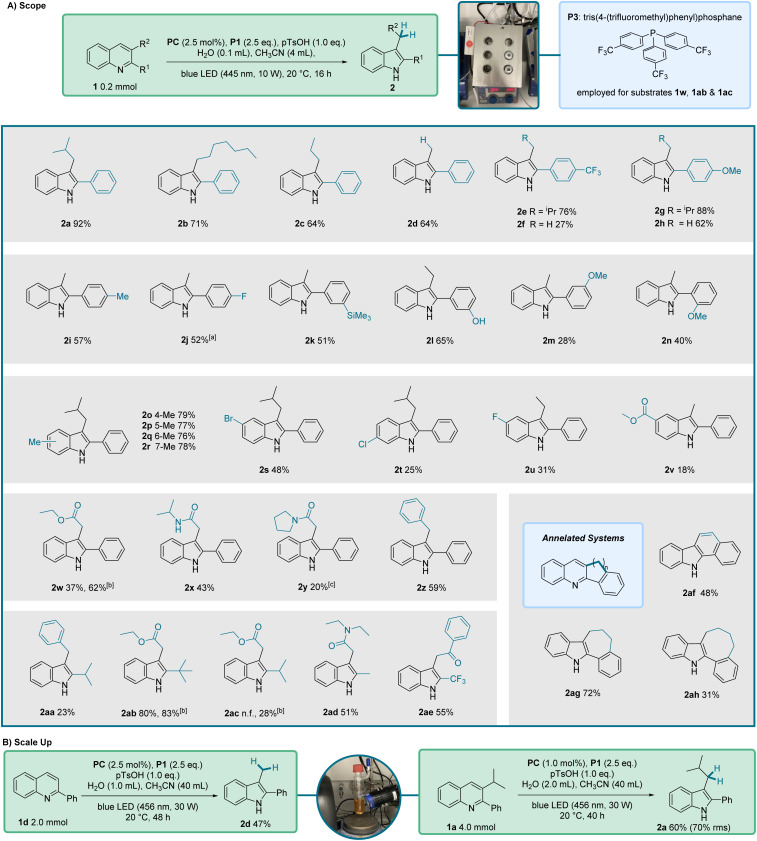
(A) Overview of the scope of the optimized reaction conditions, shown at the top. ^*a*^In this case 7% of defluorinated side-product (3-methyl-2-phenylindole) coeluted and a separation from 2j was not possible. ^*b*^P3 (P(C_6_H_4_pCF_3_)_3_) was used instead of P1 and the water-amount was reduced to 50 µL. ^*c*^2-Phenylindole was isolated was formed in 18% as a side product. (B) Scale up experiments. Abbreviations: n.f. – not formed.

To elucidate the generality of our reaction we submitted a wide range of quinolines to the optimized conditions (Fig. 2A). The 3-alkyl-substituent was varied keeping the 2-phenyl substituent and we found that linear 3-alkyl groups led to slightly lower yields as compared to the branched isopropyl-substituted congener (see 2a–2c). Of note, the disubstituted 3-ethyl-2-phenylquinoline (1c) and the monosubstituted 2-phenylquinoline (1d) provided the same yield, showing that monosubstituted quinolines are eligible substrates. We then investigated the electronic-effect that is exerted by the 2-aryl substituent on the reaction outcome. Installing a trifluoromethyl group in *para*-position resulted in a slightly reduced yield (2e, 76%). However, with the monosubstituted quinoline 1f carrying the *p*-CF_3_C_6_H_4_-substituent, the yield significantly decreased to 27% (2f). In comparison, the presence of an electron-donating *para*-methoxy group on the 2-aryl substituent had only a minor effect on the reaction efficiency, see 2g (88%) and 2h (62%). Similar yields were achieved with the *para*-methyl and *para*-fluoro congeners (2i and 2j) demonstrating that except for the trifluoromethyl compound, all products in this series were obtained in yields greater than 50%. A *meta*-trimethylsilyl (2k, 51%) and a *meta*-hydroxy (2l, 65%) substituent were well tolerated in the 2-aryl group, however, the 2-*meta*-methoxyphenyl-quinoline (1m) delivered the targeted indole 2m (28%) in low yield only. A slightly reduced yield was also noted for the *ortho*-methoxy-substituted 2-aryl quinoline 1n, likely for steric reasons (2n, 40%).

We extended our studies to examine the substituent effects on the non-heterocyclic ring of the starting quinoline. Quinolines 1o–1r, bearing a methyl group at positions 5 to 8, were tested and afforded comparable yields ranging from 76–79%. The presence of halogen substituents exerted a more pronounced effect. Compounds 2s–2u exhibited reduced yields, with values ranging from 48% to 31% for bromine and fluorine substituents at the 5-position of the indole ring, and 25% for a chlorine substituent at the 6-position. The lowest yield in this series was noted for the quinoline with a methoxycarbonyl substituent at position 6 (2v, 18%). We assume that the reduced yields (2s–2v) arise from radical fragmentations of the intermediates or the decomposition of the products under the reaction conditions.

The 3-position of 2,3-disubstituted-quinoline starting materials was diversified and it could be observed that ester, amide and aryl-functionalities were tolerated and the corresponding indoles were isolated with good yields (2w–2z, 43–62%), except for the secondary amide 2y that was isolated in 20% yield only along with 18% of 2-phenylindole as a side product. Notably, the good yield for the reductive rearrangement of ethyl ester 1w was achieved upon using phosphine P3 (P(*p*-CF_3_C_6_H_4_)_3_) in place of P1.

We next addressed the 2-position of the quinoline core to determine whether an aryl functionality is required to facilitate the reaction. In this series, the 3-substituent was varied as well. Pleasingly, we could observe that isopropyl (2aa and 2ac), *tert*-butyl (2ab), methyl (2ad) and trifluoro (2ae) groups were all tolerated as 2-substituents, significantly enlarging the scope of our reaction. The corresponding products were isolated in low to very good yields (23–83%). Furthermore, we found that 2,3-ring-annelated quinolines could be rearranged to give a carbazole 2af (48%) and indoles 2ag (72%) and 2ah (31%), demonstrating that a combined ring-contraction and ring-enlargement of the quinoline starting material is possible.

We scaled up the reaction for substrate 1d to a 2 mmol scale and were able to isolate the product in 47% yield, compared to 64% at the 0.2 mmol scale (Fig. 2B). Further, indole 2a was successfully prepared in 60% yield at 4 mmol scale. In this case, a lower catalyst loading and reduced solvent volume were used, which led to solubility issues and a slower reaction, explaining the presence of unreacted starting material. Nevertheless, the reaction had to be stopped due to product decomposition observed for this substrate during the reaction. Taking this into account, a yield of 70%, based on the consumed starting material, was obtained.

**Fig. 3:**
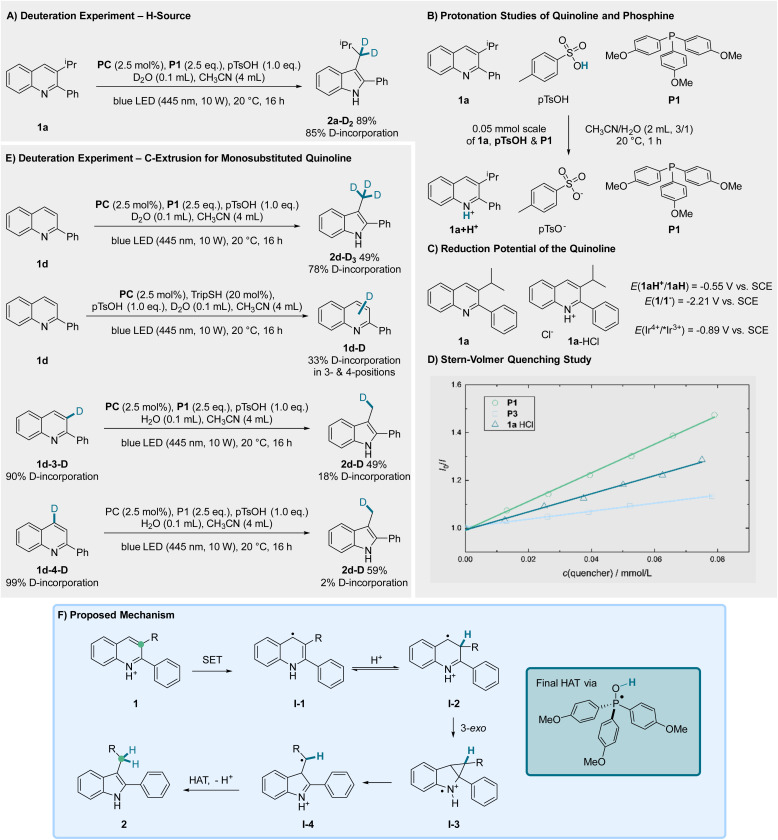
Mechanistic investigations including (A) deuteration experiments, (B) chemoselective protonation of the quinoline, (C) redox potentials, (D) Stern–Volmer quenching study, (E) deuteration experiments with C2 substituted quinoline and (F) proposed mechanism.

To investigate the reaction mechanism, several control experiments were conducted (Fig. 3). Exchange of H_2_O with D_2_O showed that the incorporated hydrogen atoms originate from the added water (Fig. 3A). In our previous work we showed that the Ir-based PC used can be oxidatively quenched by the protonated quinoline.^30^ Further, NMR studies confirmed that quinoline 1a is protonated by pTsOH, while the phosphine P1 remains in its neutral form (Fig. 3B). Cyclovoltammetry measurement (CV) of 1a revealed a reduction potential of *E* = −2.61 V *vs.* Fc/Fc^+^ (−2.21 V *vs.* SCE) and two signals for the protonated quinoline 1a-HCl at *E* = −0.95 V *vs.* Fc/Fc^+^ (−0.55 V *vs.* SCE) and *E* = −2.52 V *vs.* Fc/Fc^+^ (−2.12 V *vs.* SCE) (Fig. 3C). Thus, the excited PC (*E*(Ir^4+^/*Ir^3+^) = −0.89 V *vs.* SCE) should be able to SET-reduce the protonated quinoline but not the unprotonated form. According to the Stern–Volmer quenching experiments it can be stated that none of the used components – phosphines P1 and P3 as well as the protonated quinoline 1a-HCl – are quenching with a significant higher rate (Fig. 3D). Therefore, an initial reductive quenching of the PC by the phosphine or an oxidative quenching by the protonated quinoline is feasible.

In our initial work^69^ we proposed a neophyl-type rearrangement for the reductive transformation of 2-monosubstituted quinolines through a radical intermediate in 3-position resulting in the extrusion of the C4 carbon atom. Contrary to this, Chiba and Pratt proposed a 3-*exo* cyclization of an intermediate radical at the 4-position, followed by ring-opening and reduction based on DFT-calculations and ^13^C-labeling.^52^ Considering the 2,3-disubstituted quinolines our results perfectly align with the proposed mechanism of Chiba and Pratt, as judged from the substitution pattern of the product indoles. Thus, the protonated quinoline is first reduced *via* SET from the PC to form an enamine radical intermediate I-1. Protonation leads to I-2, which sets the stage for the 3-*exo* cyclisation to generate I-3. Ring-opening then leads to the distonic radical cation I-4 which upon HAT from the HOPAr_3_-radical and deprotonation eventually gives the rearranged indole 2 (Fig. 3F).

To investigate, whether the reductive rearrangement of 2-monosubstituted quinolines may also be explained by the Pratt/Chiba mechanism, we prepared 2-phenylquinoline with D-incorporation in 3- as well as 4-position (Fig. 3E). According to the proposed reaction mechanism, deuterium should remain incorporated in the molecule if it was connected to the C3 carbon atom, while the C4 D-labeling should get lost.^71^ We first subjected the non-labeled quinoline 1d to our reaction conditions by using D_2_O in place of H_2_O. Interestingly we found a higher D-incorporation at the methyl-substituent than expected (78%, 66% expected), indicating that an H/D-exchange might take place under the reaction conditions along with the concluding deuterium atom transfer from the DOPAr_3_ phosphoranyl radical to the indolylmethyl radical. This exchange presumably occurs after reduction at the enamine radical intermediate stage, indicating a reversible equilibrium between intermediates I-1 and I-2. Along these lines, when 1d was reacted with the PC, pTsOH, TripSH with D_2_O under irradiation, a 33% D-incorporation was found over the positions 3 and 4 (27% mono- and 3% disubstituted) in the non-rearranged quinoline. When substrates 1d-3-D and 1d-4-D were subjected to the standard reaction conditions, a remaining deuterium incorporation of 18% was observed for 1d-3-D, whereas nearly any deuterium was retained in 1d-4-D, as expected based on the suggested mechanism. The labeling loss for 1d-3-D is likely a result of the reversible protonation from I-1 in agreement with the studies above. Taken together, we suggest that the 3-*exo*-cyclization is also occurring for the reductive rearrangement of 2-monosubstituted quinolines. Moreover, these isotope labeling studies gave us the insight that the formed reduced intermediate I-1 can likely be reversibly protonated under the applied conditions.

## Conclusions

In summary, we developed a general reductive rearrangement of 2-monosubstituted and 2,3-disubstituted quinolines to indoles *via* extrusion of the carbon atom at the 3-position, applying a photocatalytic phosphine mediated water activation process. Moreover, the reaction proceeds without the need for any pre-functionalization of the quinoline substrate, underscoring the practical simplicity of this method. The formed indoles could be isolated in moderate to very good yields and the reaction showed good functional group tolerance. Scalability of the reductive rearrangement was demonstrated. This method provides a valuable addition to the toolbox in the growing field of skeletal editing.

## Author contributions

N. S., J. K., J. Z. and A. S. conceived all studies and experiments. N. S., J. K. and J. L. synthesized all compounds. CV measurements were conducted by E. S. H. All authors discussed the results and contributed to the preparation of the manuscript. All authors have given approval to the final version of the manuscript.

## Conflicts of interest

There are no conflicts to declare.

## Supplementary Material

SC-017-D5SC08793G-s001

## Data Availability

The data (NMR, HRMS, IR, melting points, CVs) that support the findings of this study have been uploaded as part of the supplementary information (SI). Supplementary information is available. See DOI: https://doi.org/10.1039/d5sc08793g.
