# Supplementary material for: Reductive rearrangement of substituted quinolines to 2,3-disubstituted indoles enabled by water activation
Source: Chem Sci. 2025 Dec 22;17(7):3775–82. doi: 10.1039/d5sc08793g (PMC12758485; doi:10.1039/d5sc08793g)
Supplement: SC-017-D5SC08793G-s001 [file SC-017-D5SC08793G-s001.pdf]

Supplementary Information (SI) for Chemical Science.
This journal is © The Royal Society of Chemistry 2025

Supporting information

Reductive Rearrangement of Substituted Quinolines to 2,3-Disubstituted Indoles Enabled by Water Activation

Nico Spreckelmeyer, Jieun Kim, Jessika Lammert, Elena Sophia Horst,
Jingjing Zhang, Armido Studer

Table of content

1. General Information 3
2. Synthesis of Starting Material 5
   2.1. Overview of Synthesized Quinolines 5
   2.2. General Procedures for Starting Material Synthesis 6
   2.3. Synthesis and Characterization of Starting Material 10
3. Reaction Optimization 29
4. Synthesis of Products 37
   4.1. Overview of Synthesized Indoles 37
   4.2. General Procedure for Product Synthesis 38
   4.3. Synthesis and Characterization of Products 39
   4.4. Synthesis and Characterization of Side Products 54
   4.5. Unsuccessful Substrates 57
5. Scale-Up Experiments 58
6. Mechanistic Investigations 61
   6.1. Deuteration Experiments – Hydrogen Source 61
   6.2. Protonation Studies 64
   6.3. Cyclic Voltammetry 67
   6.4. Stern–Volmer Quenching 68
   6.5. Deuteration Experiments — Investigations for 2-monosubstituted Quinolines 70
7. NMR Spectra 83
8. References 174

1. General Information

Reactions that are sensitive to air and/or hydrolysis were carried out in an argon atmosphere. The glassware used for this purpose was dried at 100 °C for at least 24 h before use or dried under vacuum using a heat gun. Syringes and cannulas were used for the transfer of reagents and dry solvent.

Chemicals used were purchased from ABCR, BLDPharm, Doug Discovery, Sigma-Aldrich or TCI and used as received unless otherwise stated. Commercially available **solvents** (acetonitrile (MeCN), acetone, 1,4-dioxane, toluene, dimethyl sulfoxide (DMSO), ethyl acetate (EtOAc), dimethylformamide (DMF), ethanol (EtOH) and dichloroethane (DCE)) were purchased from Acros Organics (99+%, extra dry via molecular sieve). Tetrahydrofuran (THF) and dichloromethane (DCM) were refluxed and distilled over sodium and P_2O_5, respectively. Additional solvents for extractions and column chromatographic purifications were distilled prior to use.

Purification by **column chromatography** was carried out with Merck silica gel (40-63 µm particle size) at an overpressure of up to 0.5 bar. The solvent mixtures used are shown in the corresponding syntheses. The separation of the substances was checked by thin-layer chromatography on Merck Silica Gel 60 F254 wetted aluminum plates. The substances were visualized using UV light (254 nm or 366 nm) and a potassium permanganate immersion solution (1.5 g $KMnO_4$, 10 g K_2CO_3, 1.25 mL NaOH (10%) in 200 mL H_2O).

NMR spectra were measured at room temperature on one of the following spectrometers:

- Bruker Avance II 300 (^{1}H-NMR 300 Hz, ^{13}C-NMR 76 Hz, ^{19}F-NMR 282 MHz, ^{31}P-NMR 122 MHz)
- Bruker NEO 400 (^{1}H-NMR 400 Hz, ^{13}C-NMR 101 Hz, ^{19}F-NMR 376 MHz, ^{29}Si-NMR 80 MHz)
- Agilent DD2 500 (^{1}H-NMR 500 Hz, ^{13}C-NMR 126 Hz, ^{19}F-NMR 470 MHz)
- Agilent DD2 600 (^{1}H-NMR 599 Hz, ^{13}C-NMR 151 Hz)

The data was analyzed using MestReNova software, version 14.3.2 from Mestrelab Research S.L. The corresponding chemical shifts [δ] = ppm were determined with respect to the residual solvent peaks from $CDCl_3$ (^{1}H-NMR at δ_H = 7.26 ppm and ^{13}C-NMR at δ_C = 77.16 ppm) or DMSO-d_6 (^{1}H-NMR at δ_H = 2.50 ppm and ^{13}C-NMR at δ_C = 39.52 ppm) or acetone-d_6 (^{1}H-NMR at δ_H = 2.05 ppm and ^{13}C-NMR at δ_C = 206.26 ppm), respectively. The coupling constants [J] = Hz are given with an accuracy of 0.1 Hz and the multiplicities according to the following scheme: s (singlet), d (doublet), t (triplet), q (quartet), qui (quintet), sex (sextet), hept (heptet) and m (multiplet).

Mass spectra were performed by the MS department of the Institute of Organic Chemistry at the University of Münster. The exact masses are given as mass to charge ratios m/z and were

determined by exact mass determination (high resolution mass spectroscopy, HRMS) using the electrospray ionization (ESI) method and the atmospheric pressure chemical ionization (APCI) method. The following mass spectrometers were used for this purpose:

- Exploris 120 Electrospray Orbitrap
- LTQ Orbitrap XL.

All given accurate masses are checked via the HRMS-Checker for documentation errors.[1] Known compounds were analyzed via GC/MS analysis within our lab. The masses are given as mass to charge ratios m/z and were determined by electron ionization (EI) method. The following GC/MS systems were used for this purpose:

- Agilent 7820A GC system with Agilent 5977B MSD
- Agilent Intuvo 9000 GC system with Agilent 5977C GC/MSD

IR spectra were recorded with a Jasco-FT/IR-4X spectrometer. The wavenumbers of the absorption bands are given in cm^{-1}. **Melting points** were measured with a Büchi Melting Point M-560.

Photochemical reactions were carried out using various light sources. The photoreactor shown in **figure 1** was used as the standard experimental setup. The reactor form was tempered to 20 °C using a cooling system. An LED light source was installed in the bottom of the reactor (λ = 445 nm and P = 10 W). The sealable vials shown were used for reactions in the photoreactor. Alternatively, reactions were also irradiated with a Kessil lamp PR160-456 nm at λ = 456 nm (P = 30 W), whereby a Schlenk tube was used as a reaction vessel, which was ventilated with a fan during the reaction. The light source used is labeled with the corresponding wavelength and power in the respective reaction instructions.

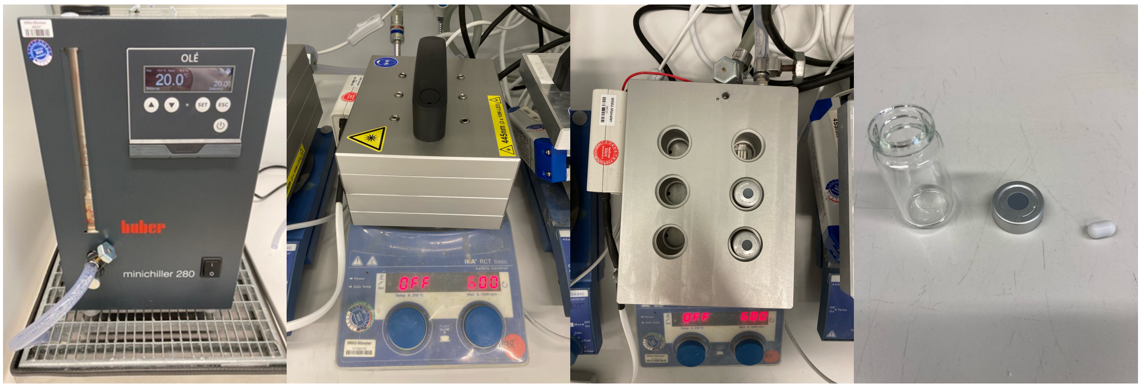

Figure 1 Reaction set up of the photo boxes used for the **GP8** for the indole rearrangement.

2. Synthesis of Starting Material

2.1. Overview of Synthesized Quinolines

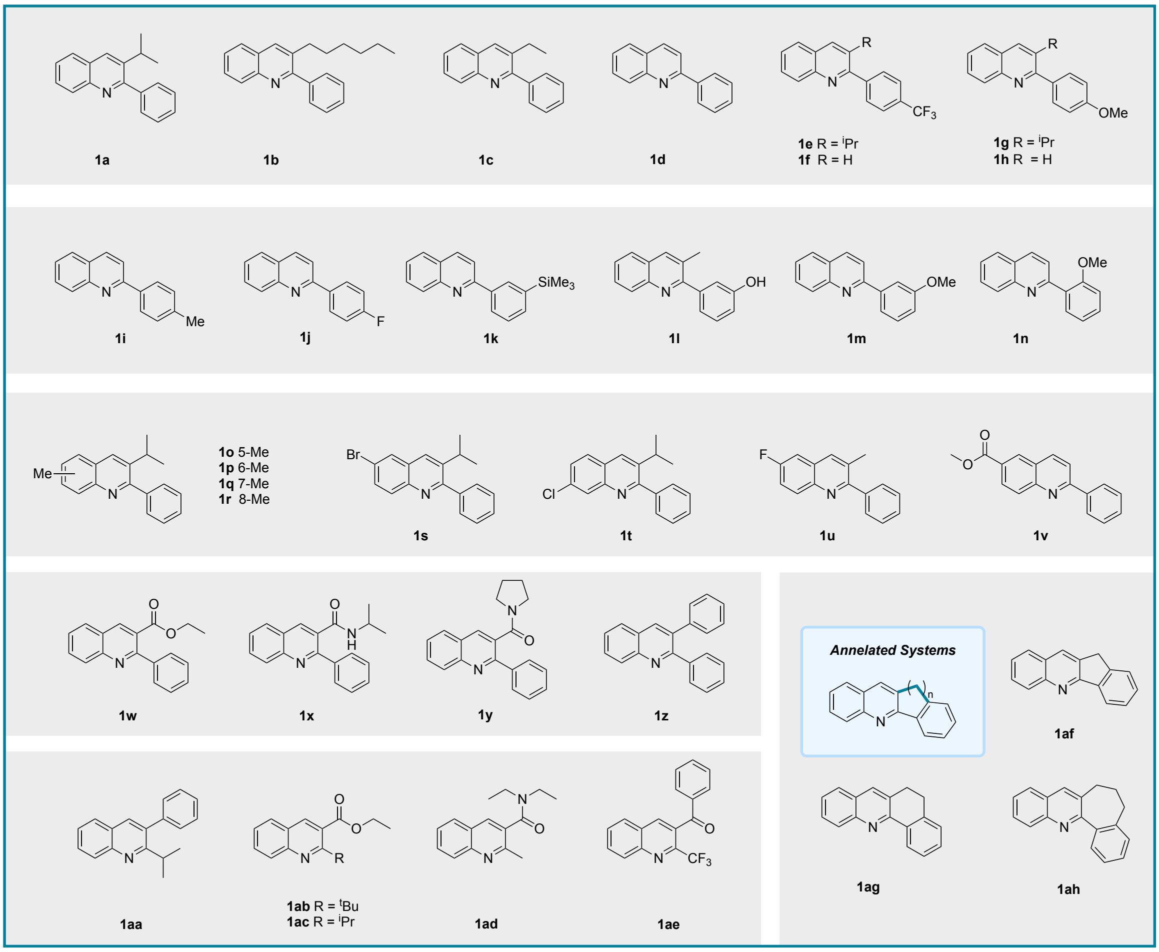

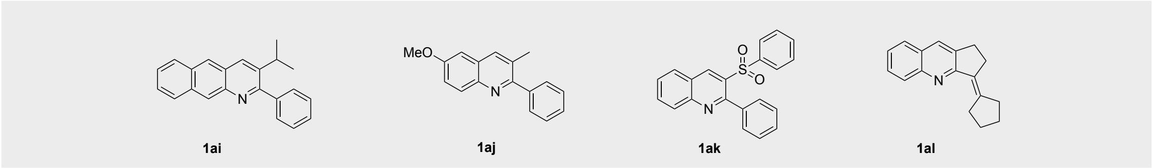

2.2. General Procedures for Starting Material Synthesis

General Procedure 1 (GP1) – Synthesis of Quinolines

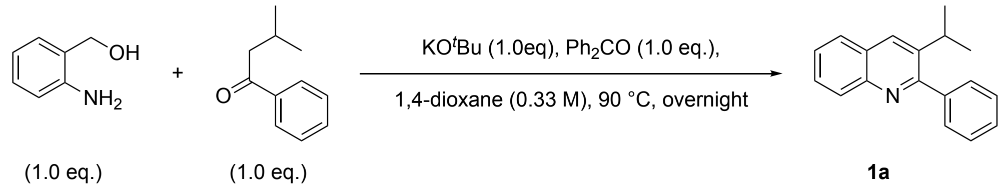

The synthesis of quinolines is performed in accordance to an adjusted literature procedure, shown with **1a** as exemplary quinoline.[2]

2-Aminobenzyl alcohol (1.0 eq.) is charged into a pre-dried Schlenk flask and set under argon atmosphere. The solid is dissolved in 1,4-dioxane (0.33 M) and ketone (1.0 eq.), KO*t*Bu (1.0 eq) as well as Ph_2CO (1.0 eq.) were added. The reaction mixture is heated to 90 °C and stirred at this temperature until complete conversion of starting material is observed – usually overnight. The mixture is filtered over celite and cooled down to room temperature. The organic layer is diluted with EtOAc and extracted with HCl solution (2 M). The combined aqueous layer is basified with NaOH solution (w = 10% in H_2O) and is extracted with DCM upon cool down to room temperature. The combined organic layer was dried over $MgSO_4$ and filtered. The solvents were removed at the rotary evaporator under reduced pressure. The crude product was checked via ^{1}H-NMR whether further purification with column chromatography using SiO_2 was necessary. If so, further details as well as deviations from **GP1** are given in the respective entries.

General Procedure 2 (GP2) – Synthesis of Quinolines

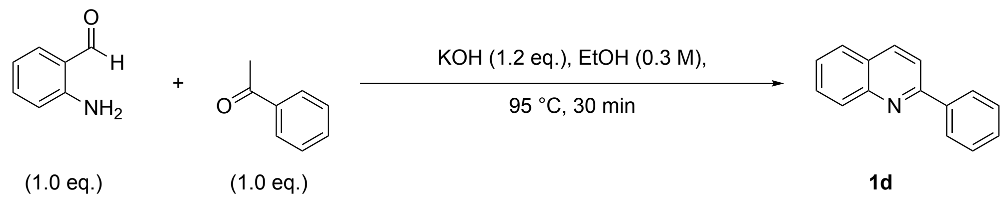

The synthesis of amides is performed in accordance to an adjusted literature procedure, shown with **1d** as exemplary quinoline.[3]

2-aminobenzaldehyd (1.0 eq.) was added into a pre-dried Schlenk-flask under argon. The solid was dissolved in EtOH (0.3 M), the respective ketone (1.0 eq.) and KOH (1.2 eq.) were added to the mixture. The reaction mixture was stirred at 95 °C for 30 min. The mixture was cooled down to r.t., diluted with DCM and filtered over silica. The filtrate was dried over $MgSO_4$ and filtered. The solvents were removed at the rotary evaporator under reduced pressure. The

crude product was purified via column chromatography using SiO_2. Deviations from **GP2** are declared in the respective entries.

General Procedure 3 (GP3) – Synthesis of Ketones

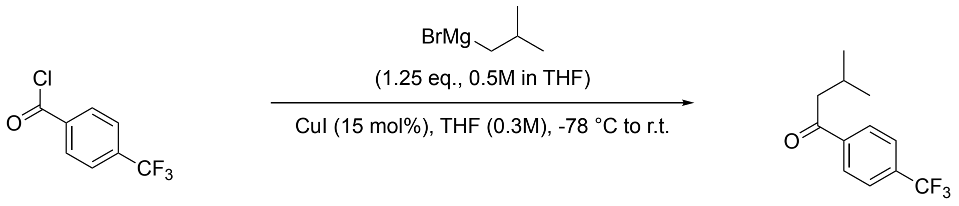

The synthesis of ketones is performed in accordance to an adjusted literature procedure, shown with the 3-methyl-1-(4-(trifluoromethyl)phenyl)butan-1-one as exemplary ketone.[4]
CuI (15 mol%) was charged into a pre-dried Schlenk flask and set under argon atmosphere. THF (0.3 M) and acid chloride (1.0 eq.) were added consecutively. The mixture was cooled down to -78 °C and isobutyl magnesium bromide (1.25 eq., 0.5 M in THF) was added over 30 minutes at this temperature. The reaction mixture was warmed up to r.t. overnight, diluted with EtOAc and quenched by addition of NH_4Cl. The aqueous layer was extracted with EtOAc and the combined organic layers were dried over $MgSO_4$ and filtered. The solvents were removed at the rotary evaporator under reduced pressure. The crude product was purified via column chromatography using SiO_2. Deviations from **GP3** are declared in the respective entries.

General Procedure 4 (GP4) – Synthesis of Quinolines

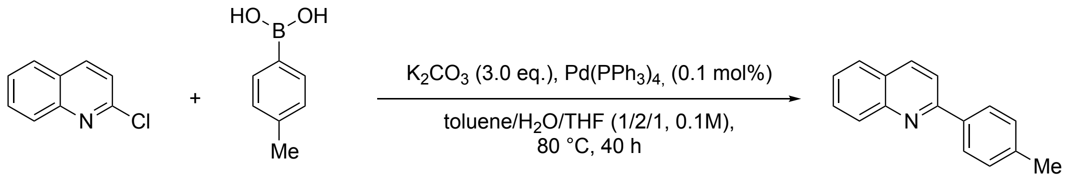

(1.0 eq.) (1.0 eq.) **1i**

The synthesis of quinolines is performed in accordance to an adjusted literature procedure, shown with **1i** as exemplary quinoline.[5]
Quinoline (1.0 eq.), boronic acid (1.2 eq.), K_2CO_3 (3.0 eq.) and $Pd(PPh_3)_4$ (10mol%) were charge into a pre-dried Schlenk-flask and set under argon atmosphere. The solids were dissolved in a toluene/H_2O/THF-mixture (1/2/1, 0.1 M). The reaction mixture was purged with argon for 15 min and the reaction mixture was heated to 80 °C for 40 h. The mixture was cooled down to r.t. and diluted with DCM. The aqueous layer was extracted with DCM and the combined organic layers were dried over $MgSO_4$ and filtered. The solvents were removed at

the rotary evaporator under reduced pressure. The crude product was purified via column chromatography using SiO_2. Deviations from **GP4** are declared in the respective entries.

General Procedure 5 (GP5) – Synthesis of Quinolines

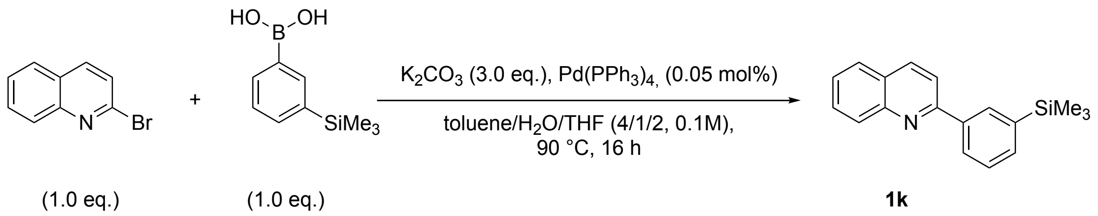

The synthesis of quinolines is performed in accordance to an adjusted literature procedure, shown with **1k** as exemplary quinoline.[6]

Quinoline (1.0 eq.), boronic acid (1.5 eq.), K_2CO_3 (3.0 eq.) and $Pd(PPh_3)_4$ (5mol%) were charge into a pre-dried Schlenk-flask and set under argon atmosphere. The solids were dissolved in a toluene/H_2O/EtOH-mixture (4/1/2, 0.1 M). The reaction mixture was purged with argon for 15 min and the reaction mixture was heated to 90 °C for 16 h. The mixture was cooled down to r.t. and diluted with H_2O and EtOAc. The aqueous layer was extracted with EtOAc and the combined organic layers were dried over $MgSO_4$ and filtered. The solvents were removed at the rotary evaporator under reduced pressure. The crude product was purified via column chromatography using SiO_2. Deviations from **GP5** are declared in the respective entries.

General Procedure 6 (GP6) – Synthesis of Quinolines

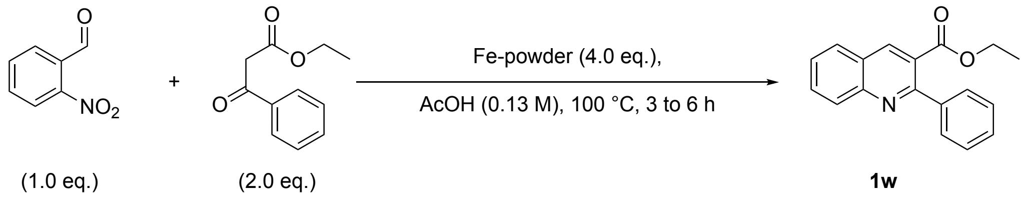

The synthesis of quinolines is performed in accordance to an adjusted literature procedure, shown with **1w** as exemplary quinoline.[7]

2-nitrobenzaldehyde (1.0 eq.) is charged into a pre-dried Schlenk flask and set under argon atmosphere. The solid is dissolved in AcOH (0.13 M) and ketone (2.0 to 3.0 eq) was added. The reaction mixture is heated to 100 °C and stirred at this temperature for 1 minutes. Fe-powder (4.0 eq.) was added and the reaction mixture was stirred at 100 °C until complete consumption of starting material was observed via TLC. The mixture was cooled down to r.t. and filtered over celite and diluted with Et_2O. The organic layer was washed with water, sat.

$NaHCO_3$ and brine. The combined organic layer was dried over $MgSO_4$, filtered and the solvent was removed at the rotary evaporator under reduced pressure. The crude product was purified via column chromatography using SiO_2. Deviations from **GP6** are declared in the respective entries.

General Procedure 7 (GP7) – Synthesis of Amides

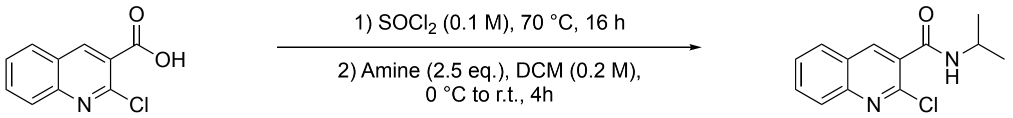

The synthesis of amides is performed in accordance to an adjusted literature procedure, shown with 2-chloro-N-isopropylquinoline-3-carboxamide as exemplary amide.[8,9]

Carboxylic acid (1.0 eq.) was dissolved in $SOCl_2$ (0.1 M) under argon atmosphere and refluxed for 16 h at 70 °C. The excess of $SOCl_2$ was removed under high vacuum and the formed acid chloride was used in the next reaction step. Isopropylamine (2.5 eq.) was dissolved in DCM (1 M) and the acid chloride (1.0 eq.) in DCM (0.4 M) was added at 0 °C. The reaction mixture was warmed up to r.t. and stirred for 4 h. The reaction was quenched by addition of NaOH solution (w = 5% in H_2O) and the aqueous layer was extracted with EtOAc. The combined organic layers were dried over $MgSO_4$ and filtered. The solvents were removed at the rotary evaporator under reduced pressure. The crude product was purified via column chromatography using SiO_2. Deviations from **GP7** are declared in the respective entries.

2.3. Synthesis and Characterization of Starting Material

2-phenyl-3-propan-2-ylquinoline (1a):

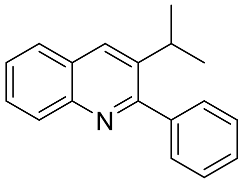

The reaction was performed according to **GP1** with (2-aminophenyl)methanol (0.62 g, 5.0 mmol, 1.0 eq.) and 3-methyl-1-phenylbutan-1-one (0.81 g, 5.0 mmol, 1.0 eq.). After purification via Flash-Chromatography (P/EtOAc – 25/1), the product **1a** was obtained as a yellow solid (0.42 g, 1.7 mmol, 34%).

^{1}H-NMR (300 MHz, $CDCl_3$): δ (ppm) = 8.16 – 8.10 (m, 2H), 7.83 (dd, *J* = 8.2, 1.5 Hz, 1H), 7.67 (ddd, *J* = 8.5, 6.9, 1.5 Hz, 1H), 7.59 – 7.38 (m, 6H), 3.26 (hept, *J* = 6.8 Hz, 1H), 1.26 (d, *J* = 6.8 Hz, 6H).

^{13}C-NMR (76 MHz, $CDCl_3$): δ (ppm) = 160.5, 146.3, 141.1, 140.5, 132.9, 129.4, 129.0, 128.9, 128.4, 128.1, 127.9, 127.2, 126.4, 29.4, 24.3

MS (EI): m/z calculated for $[M]^{\bullet+}$ $C_{18}H_{17}N^{\bullet+}$ 247.1, found 247.1.

The analytical data match those reported in the literature.[10]

3-hexyl-2-phenylquinoline (1b):

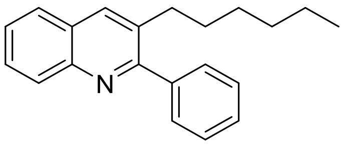

The reaction was performed according to **GP1** with (2-aminophenyl)methanol (0.25 g, 2.0 mmol, 1.0 eq.) and 1-phenyloctan-1-one (0.41 g, 2.0 mmol, 1.0 eq.). After purification via Flash-Chromatography (P/EtOAc – 25/1), the product **1b** was obtained as a yellow oil (0.54 g, 1.8 mmol, 93%).

^{1}H-NMR (300 MHz, $CDCl_3$): δ (ppm) = 8.13 (dd, *J* = 8.5, 1.1 Hz, 1H), 8.03 (s, 1H), 7.81 (dd, *J* = 8.1, 1.4 Hz, 1H), 7.66 (ddd, *J* = 8.4, 6.9, 1.5 Hz, 1H), 7.56 – 7.52 (m, 3H), 7.51 – 7.42 (m, 3H), 2.77 (t, *J* = 7.8 Hz, 2H), 1.57 – 1.50 (m, 2H), 1.29 – 1.14 (m, 6H), 0.83 (t, *J* = 6.6 Hz, 3H).

^{13}C-NMR (76 MHz, $CDCl_3$): δ (ppm) = 160.9, 146.5, 141.1, 135.8, 134.3, 129.4, 128.9, 128.4, 128.2, 127.8, 127.0, 126.5, 33.0, 31.6, 30.7, 29.1, 22.6, 14.2.

MS (EI): m/z calculated for $[M^{\bullet+}]$ $C_{18}H_{17}N^{\bullet+}$ $C_{21}H_{23}N^{\bullet+}$ 289.2, found 289.2.

The analytical data match those reported in the literature.[11]

3-ethyl-2-phenylquinoline (1c):

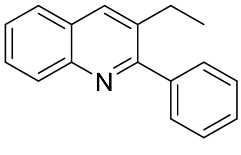

The reaction was performed according to **GP1** with (2-aminophenyl)methanol (0.37 g, 3.0 mmol, 1.0 eq.) and 1-phenylbutan-1-one (0.44 g, 3.0 mmol, 1.0 eq.). After purification via Flash-Chromatography (P/EtOAc – 30/1), the product **1c** was obtained as a yellow oil (0.57 g, 2.5 mmol, 82%).

^{1}**H-NMR** (300 MHz, $CDCl_3$): δ(ppm) = 8.13 (d, J = 8.0 Hz, 1H), 8.05 (s, 1H), 7.82 (dd, J = 8.0, 1.5 Hz, 1H), 7.67 (ddd, J = 8.5, 6.9, 1.5 Hz, 1H), 7.58 – 7.39 (m, 6H), 2.81 (q, J = 7.5 Hz, 2H), 1.20 (t, J = 7.5 Hz, 3H).

^{13}**C-NMR** (76 MHz, $CDCl_3$): δ(ppm) = 160.8, 146.5, 141.1, 135.5, 135.0, 129.5, 128.9, 128.9, 128.4, 128.2, 127.9, 127.1, 126.5, 26.2, 14.9.

MS (EI): m/z calculated for $[M]^{\bullet+}$ $C_{17}H_{15}N^{\bullet+}$ 233.1, found 233.1.

The analytical data match those reported in the literature.[11]

2-phenylquinoline (1d):

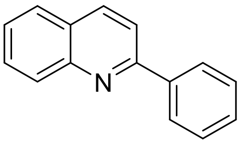

The reaction was performed according to **GP2** with 2-aminobenzaldehyde (0.60 g, 5.0 mmol, 1.0 eq.) and acetophenone (0.60 g, 5.0 mmol, 1.0 eq.). After purification via Flash-Chromatography (P/EtOAc – 20/1), the product XX was obtained as a yellow oil (0.65 g, 3.1 mmol, 64%).

^{1}**H-NMR** (400 MHz, $CDCl_3$): δ(ppm) = 8.23 (d, J = 8.6 Hz, 1H), 8.18 (m, 3H), 7.89 (d, J = 8.6 Hz, 1H), 7.83 (dd, J = 8.1, 1.5 Hz, 1H), 7.74 (ddd, J = 8.5, 6.9, 1.5 Hz, 1H), 7.58 – 7.51 (m, 3H), 7.51 – 7.44 (m, 1H).

^{13}**C-NMR** (101 MHz, $CDCl_3$): δ(ppm) = 157.5, 148.5, 139.9, 136.9, 129.9, 129.8, 129.5, 129.0, 127.7, 127.6, 127.3, 126.4, 119.1.

MS (EI): m/z calculated for $[M^{\bullet+}]$ $C_{15}H_{11}N^{\bullet+}$ 205.1, found 205.0.

The analytical data match those reported in the literature.[3]

3-isopropyl-2-(4-(trifluoromethyl)phenyl)quinoline (1e):

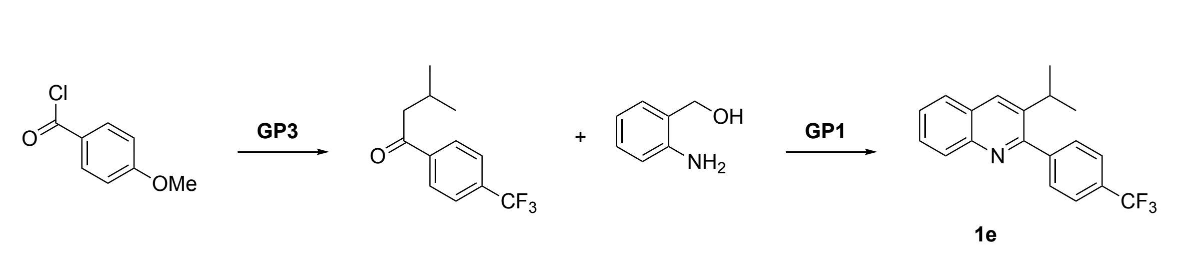

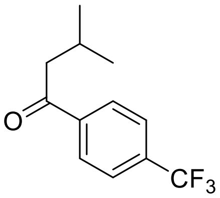

The reaction was performed according to **GP3** with 4-(trifluoromethyl)benzoyl chloride (1.04 g, 5.0 mmol, 1.0 eq.). The crude was filtrated through a SiO_2-pad (P/EtOAc – 10/1) and the ketone 1-(4-trifluoromethylphenyl)-3-methylbutan-1-one was obtained as a colorless oil (0.77 g). The product was confirmed via ^{1}H-NMR and directly used in the next reaction step, albeit still containing impurities.

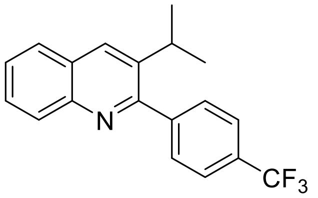

The reaction was performed according to **GP1** with (2-aminophenyl)methanol (0.37 g, 3.0 mmol, 1.0 eq.) and 1-(4-trifluoromethylphenyl)-3-methylbutan-1-one. After purification via Flash-Chromatography (P/EtOAc – 30/1 to 20/1), the product **1e** was obtained as a white solid (96.8 mg, 0.31 mmol, 10%).

^{1}H-NMR (500 MHz, $CDCl_3$): δ (ppm) = 8.16 (s, 1H), 8.10 (dd, J = 8.4, 1.0 Hz, 1H), 7.87 – 7.83 (m, 1H), 7.78 – 7.74 (m, 2H), 7.71 – 7.64 (m, 4H), 7.56 (ddd, J = 8.1, 6.9, 1.2 Hz, 1H), 3.18 (hept, J = 6.8 Hz, 1H), 1.27 (d, J = 6.9 Hz, 6H).

^{13}C-NMR{^{19}F} (126 MHz, $CDCl_3$): δ (ppm) = 159.0, 146.3, 144.8, 140.2, 133.3, 130.4, 129.4, 129.4, 129.3, 128.1, 127.3, 126.9, 125.5, 124.4, 29.5, 24.3.

^{19}F{^{1}H}-NMR (470 MHz, $CDCl_3$): δ (ppm) = -62.6.

HRMS (ESI): m/z calculated for $[M+H]^+$ $C_{19}H_{17}NF_3^+$ 316.1308, found 316.1307.

IR (Solid): $\tilde{\nu}$ (cm^{-1}) = 3049, 2963, 2926, 2872, 1611, 1516, 1490, 1456, 1405, 1317, 1243, 1158, 1104, 1080, 1063, 1018, 996, 911, 876, 856, 842, 797, 758, 748, 709, 621, 576, 477.

Melting point: T (°C) = 75-76.

2-(4-(trifluoromethyl)phenyl)quinoline (1f):

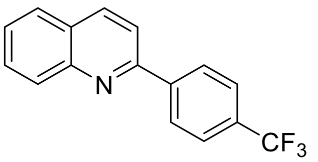

The reaction was performed according to **GP2** with 2-aminobenzaldehyde (0.30 g, 2.5 mmol, 1.0 eq.) and 1-[4-(trifluoromethyl)phenyl]ethanone (0.47 g, 2.5 mmol, 1.0 eq.). After purification via Flash-Chromatography (P/EtOAc – 20/1), the product **1f** was obtained as a white solid (0.16 g, 0.57 mmol, 23%).

^{1}H-NMR (500 MHz, $CDCl_3$): δ (ppm) = 8.32 – 8.24 (m, 3H), 8.22 – 8.16 (m, 1H), 7.90 (d, J = 8.6 Hz, 1H), 7.86 (dd, J = 8.1, 1.5 Hz, 1H), 7.81 – 7.73 (m, 3H), 7.57 (ddd, J = 8.0, 7.0, 1.2 Hz, 1H).

^{13}C{^{19}F}-NMR (126 MHz, $CDCl_3$): δ (ppm) = 155.8, 148.4, 143.1, 137.3, 131.2, 130.1, 130.0, 128.0, 127.7, 127.6, 127.0, 125.9, 124.4, 118.9.

^{19}F{^{1}H}-NMR (470 MHz, $CDCl_3$): δ (ppm) = -62.6.

MS (EI): m/z calculated for $[M^{\bullet+}]$ $C_{16}H_{10}F_3N^{\bullet+}$ 273.1, found 273.0.

The analytical data match those reported in the literature.[12,13]

3-isopropyl-2-(4-methoxyphenyl)quinoline (1g):

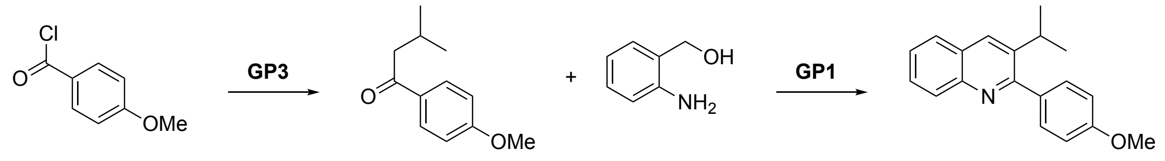

1g

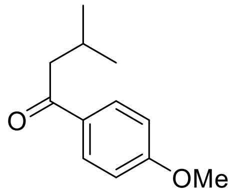

The reaction was performed according to **GP3** with 4-methoxybenzoyl chloride (0.85 g, 5.0 mmol, 1.0 eq.). The crude was filtrated through a SiO_2-pad (P/EtOAc – 10/1) and the ketone 1-(4-methoxyphenyl)-3-methylbutan-1-one was obtained as a colorless oil (0.56 g). The product was confirmed via ^{1}H-NMR and directly used in the next reaction step, albeit still containing impurities.

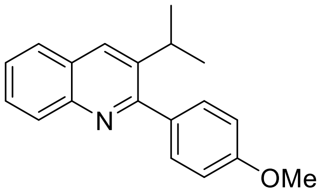

The reaction was performed according to **GP1** with (2-aminophenyl)methanol (0.35 g, 2.8 mmol, 1.0 eq.) and 1-(4-methoxyphenyl)-3-methylbutan-1-one. After purification via Flash-Chromatography (P/EtOAc – 30/1 to 20/1), the product **1g** was obtained as a brown solid (103 mg, 0.371 mmol, 13%).

^{1}H-NMR (599 MHz, $CDCl_3$): δ (ppm) = 8.14 – 8.09 (m, 2H), 7.81 (dd, J = 8.1, 1.4 Hz, 1H), 7.65 (ddd, J = 8.3, 6.9, 1.4 Hz, 1H), 7.54 – 7.44 (m, 3H), 7.06 – 7.00 (m, 2H), 3.88 (s, 3H), 3.31 (hept, J = 6.9 Hz, 1H), 1.26 (d, J = 6.9 Hz, 6H).

^{13}C-NMR (151 MHz, $CDCl_3$): δ (ppm) = 160.2, 159.7, 146.3, 140.7, 133.6, 132.9, 130.3, 129.3, 128.9, 127.8, 127.2, 126.3, 113.9, 55.5, 29.4, 24.3.

HRMS (ESI): m/z calculated for $[M+Na]^+$ $C_{19}H_{19}NONa^+$ 300.1359, found 300.1357.

IR (Solid): $\tilde{\nu}$ (cm^{-1}) = 3042, 2961, 2920, 2871, 2832, 1607, 1577, 1557, 1513, 1488, 1454, 1416, 1364, 1348, 1302, 1284, 1239, 1177, 1148, 1133, 1102, 1078, 1024, 995, 968, 917, 876, 835, 802, 784, 766, 638, 625, 617, 592, 543, 523, 482.

Melting point: T (°C) = 112-113.

2-(4-methoxyphenyl)quinoline (1h):

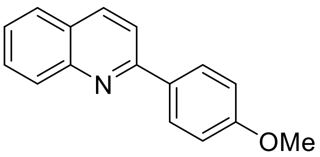

The reaction was performed according to **GP1** with (2-aminophenyl)methanol (0.62 g, 5.0 mmol, 1.0 eq.) and 1-(4-methoxyphenyl)ethanone (0.83 g, 5.5 mmol, 1.1 eq.). After purification via Flash-Chromatography (P/EtOAc – 20/1 to 10/1), the product **1h** was obtained as a white solid (0.94 g, 4.0 mmol, 79%).

^{1}**H-NMR** (400 MHz, $CDCl_3$): δ (ppm) = 8.21 – 8.10 (m, 4H), 7.84 (d, *J* = 8.6 Hz, 1H), 7.80 (dd, *J* = 8.1, 1.5 Hz, 1H), 7.71 (ddd, *J* = 8.4, 6.9, 1.5 Hz, 1H), 7.50 (ddd, *J* = 8.0, 6.9, 1.2 Hz, 1H), 7.09 – 7.01 (m, 2H), 3.89 (s, 3H).

^{13}**C-NMR** (101 MHz, $CDCl_3$): δ (ppm) = 161.0, 157.0, 148.4, 136.7, 132.4, 129.7, 129.7, 129.0, 127.6, 127.0, 126.0, 118.7, 114.4, 55.5.

MS (EI): m/z calculated for $[M^{\bullet+}]$ $C_{16}H_{13}NO^{\bullet+}$ 235.1, found 235.1.

The analytical data match those reported in the literature.[14]

2-(*p*-tolyl)quinoline (1i):

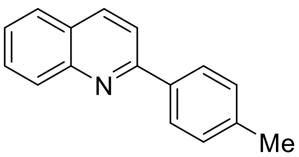

The reaction was performed according to **GP4** with 2-chloroquinoline (0.49 g, 3.0 mmol, 1.0 eq.) and *p*-tolylboronic acid (0.49 g, 3.6 mmol, 1.2 eq.). After purification via Flash-Chromatography (P/EtOAc – 20/1), the product **1i** was obtained as a white solid (0.61 g, 2.8 mmol, 93%).

^{1}**H-NMR** (400 MHz, $CDCl_3$): δ (ppm) = 8.23 – 8.14 (m, 2H), 8.12 – 8.04 (m, 2H), 7.87 (d, *J* = 8.6 Hz, 1H), 7.82 (dd, *J* = 8.1, 1.5 Hz, 1H), 7.72 (ddd, *J* = 8.4, 6.9, 1.5 Hz, 1H), 7.51 (ddd, *J* = 8.1, 6.9, 1.2 Hz, 1H), 7.34 (d, *J* = 7.6 Hz, 2H), 2.44 (s, 3H).

^{13}**C-NMR** (101 MHz, $CDCl_3$): δ (ppm) = 157.5, 148.4, 139.6, 137.0, 136.8, 129.8, 129.7, 127.6, 127.6, 127.2, 126.2, 119.0, 21.5.

HRMS (ESI): m/z calculated for $[M+H]^+$ $C_{16}H_{14}N^+$ 220.1121, found 220.1121.

The analytical data match those reported in the literature.[15]

2-(4-fluorophenyl)quinoline (1j):

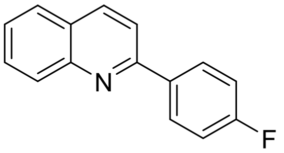

The reaction was performed according to **GP4** with 2-chloroquinoline (0.49 g, 3.0 mmol, 1.0 eq.) and (4-fluorophenyl)boronic acid (0.40 g, 3.6 mmol, 1.2 eq.). After purification via Flash-Chromatography (P/Et_2O – 10/1 to 5/1), the product **1j** was obtained as a white solid (0.61 g, 2.8 mmol, 90%).

^{1}**H-NMR** (300 MHz, $CDCl_3$): δ (ppm) = 8.28 – 8.09 (m, 4H), 7.90 – 7.81 (m, 2H), 7.80 – 7.70 (m, 1H), 7.60 – 7.50 (m, 1H), 7.31 – 7.18 (m, 2H).

^{13}**C-NMR** (76 MHz, $CDCl_3$): δ (ppm) = 163.9 (d, *J* = 249.0 Hz), 156.3, 148.3, 137.0, 135.9 (d, *J* = 3.2 Hz), 129.9, 129.8, 129.5 (d, *J* = 8.4 Hz), 127.6, 127.2, 126.5, 118.7, 115.9 (d, *J* = 21.6 Hz).

^{19}**F-NMR** (282 MHz, $CDCl_3$): δ (ppm) = -112.5.

HRMS (ESI): m/z calculated for $[M+H]^+$ $C_{15}H_{11}FN^+$ 224.0870, found 224.0870.

The analytical data match those reported in the literature.[12]

2-(3-(trimethylsilyl)phenyl)quinoline (1k):

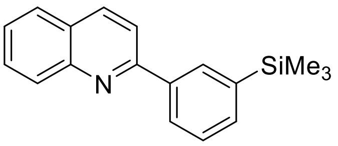

The reaction was performed according to **GP5** with 2-bromoquinoline (0.83 g, 4.0 mmol, 1.0 eq.) and (3-trimethylsilylphenyl)boronic acid (1.16 g, 6.00 mmol, 1.5 eq.). After purification via Flash-Chromatography (P/EtOAc – 60/1 to 30/1), the product **1k** was obtained as a white solid (0.89 g, 3.2 mmol, 81%).

^{1}H-NMR (300 MHz, $CDCl_3$): δ (ppm) = 8.29 – 8.09 (m, 4H), 7.92 – 7.81 (m, 2H), 7.74 (ddt, *J* = 8.1, 6.9, 1.3 Hz, 1H), 7.66 – 7.61 (m, 1H), 7.53 (tdd, *J* = 7.7, 2.3, 1.0 Hz, 1H), 0.36 (s, 9H).

^{13}C-NMR (76 MHz, $CDCl_3$): δ (ppm) = 158.0, 148.5, 141.2, 139.1, 136.8, 134.5, 132.6, 129.9, 129.7, 128.3, 128.3, 127.6, 127.3, 126.4, 119.4, -0.9.

^{29}Si-NMR (80 MHz, $CDCl_3$): δ (ppm) = -3.5.

HRMS (ESI): m/z calculated for $[M+H]^+$ $C_{18}H_{20}NSi^+$ 278.1360, found 278.1360.

IR (Solid): $\tilde{\nu}$ (cm^{-1}) = 3036, 2951, 2898, 1947, 1829, 1617, 1596, 1552, 1501, 1456, 1427, 1392, 1303, 1283, 1244, 1127, 1113, 1066, 973, 942, 877, 829, 798, 788, 756, 697, 667, 622, 480, 461.

Melting point: *T* (°C) = 42-43.

3-(3-methylquinolin-2-yl)phenol (1l):

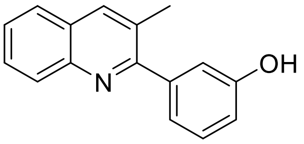

The synthesis of quinoline **1l** is performed in accordance to an adjusted literature procedure.[16] 2-chloro-3-methylquinoline (0.71 g, 4.0 mmol, 1.0 eq.), (3-hydroxyphenyl)boronic acid (0.83 g, 6.0 mmol, 1.5 eq.), K_3PO_4 (1.27 g, 6.00 mmol, 1.5 eq.), $Pd_2(dba)_3$ (45 mg, 50 µmol, 1.25mol%) and SPhos (82 mg, 0.20 mmol, 5mol%) were charge into a pre-dried Schlenk-flask and set under argon atmosphere. The solids were dissolved in 1,4-dioxane (25 mL, 0.1 M). The reaction mixture was purged with argon for 15 min and the reaction mixture was heated to 100 °C for 16 h. The mixture was cooled down to r.t. and diluted with H_2O and EtOAc. The aqueous layer was extracted with EtOAc and the combined organic layers were dried over $MgSO_4$ and filtered. The solvents were removed at the rotary evaporator under reduced pressure. After purification via Flash-Chromatography (P/EtOAc – 5/1 to 2/1), the product **1l** was obtained as an off white solid (0.73 g, 3.1 mmol, 78%).

^{1}H-NMR (599 MHz, $CDCl_3$): δ (ppm) = 9.56 (s, 1H), 8.22 (t, *J* = 1.0 Hz, 1H), 7.97 (dq, *J* = 9.2, 0.9 Hz, 1H), 7.91 (dd, *J* = 8.2, 1.4 Hz, 1H), 7.70 (ddd, *J* = 8.4, 6.8, 1.5 Hz, 1H), 7.57 (ddd, *J* = 8.1, 6.8, 1.2 Hz, 1H), 7.29 (ddd, *J* = 8.1, 7.3, 0.5 Hz, 1H), 7.04 – 6.98 (m, 2H), 6.87 (ddd, *J* = 8.2, 2.4, 1.0 Hz, 1H), 2.43 (d, *J* = 1.0 Hz, 3H).

^{13}C-NMR (151 MHz, $CDCl_3$): δ (ppm) = 159.8, 157.1, 145.9, 141.7, 136.6, 129.0, 128.9, 128.8, 128.6, 127.1, 127.0, 126.4, 119.6, 115.9, 115.1, 20.2.

HRMS (ESI): m/z calculated for $[M+Na]^+$ $C_{16}H_{13}NONa^+$ 258.0889, found 258.0898.

IR (Solid): $\tilde{\nu}$ (cm^{-1}) = 3047, 2919, 2792, 2650, 2608, 2550, 1615, 1600, 1577, 1494, 1475, 1440, 1417, 1357, 1295, 1214, 1159, 1146, 1126, 1033, 998, 923, 908, 868, 854, 802, 778, 752, 718, 698, 615, 552, 530, 499, 476, 463.
Melting point: T (°C) = 201-202.

2-(3-methoxyphenyl)quinoline (1m):

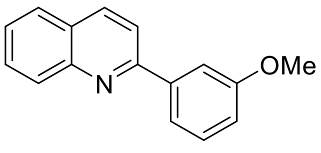

The reaction was performed according to **GP4** with 2-chloroquinoline (0.49 g, 3.0 mmol, 1.0 eq.) and (3-methoxyphenyl)boronic acid (0.55 g, 3.6 mmol, 1.2 eq.). After purification via Flash-Chromatography (P/EtOAc – 20/1), the product **1m** was obtained as a brown solid (0.66 g, 2.8 mmol, 93%).
^{1}H-NMR (400 MHz, $CDCl_3$): δ (ppm) = 8.26 – 8.16 (m, 2H), 7.87 (d, J = 8.5 Hz, 1H), 7.83 (dd, J = 8.1, 1.5 Hz, 1H), 7.78 (dd, J = 2.6, 1.6 Hz, 1H), 7.76 – 7.69 (m, 2H), 7.53 (ddd, J = 8.1, 6.9, 1.2 Hz, 1H), 7.44 (t, J = 7.9 Hz, 1H), 7.02 (ddd, J = 8.2, 2.6, 1.0 Hz, 1H), 3.94 (s, 3H).
^{13}C-NMR (101 MHz, $CDCl_3$): δ (ppm) = 160.3, 157.3, 148.3, 141.3, 136.9, 130.0, 129.9, 129.8, 127.6, 127.4, 126.5, 120.2, 119.3, 115.6, 112.9, 55.6.
HRMS (ESI): m/z calculated for $[M+H]^+$ $C_{16}H_{14}NO^+$ 236.1070, found 236.1068.
The analytical data match those reported in the literature.[15]

2-(2-methoxyphenyl)quinoline (1n):

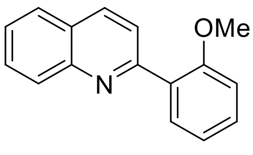

The reaction was performed according to **GP1** with (2-aminophenyl)methanol (0.25 g, 2.0 mmol, 1.0 eq.) and 1-(2-methoxyphenyl)ethanone (0.30 g, 2.0 mmol, 1.0 eq.). The product **1n** was obtained without any further purification necessary as a yellow oil (0.43 g, 1.8 mmol, 92%).
^{1}H-NMR (400 MHz, $CDCl_3$): δ (ppm) = 8.16 (ddd, J = 11.6, 8.5, 0.9 Hz, 2H), 7.89 (d, J = 8.5 Hz, 1H), 7.84 (ddd, J = 9.0, 7.7, 1.7 Hz, 2H), 7.71 (ddd, J = 8.5, 6.9, 1.5 Hz, 1H), 7.53 (ddd, J = 8.1, 6.9, 1.2 Hz, 1H), 7.43 (ddd, J = 8.2, 7.4, 1.8 Hz, 1H), 7.13 (td, J = 7.5, 1.1 Hz, 1H), 7.04 (dd, J = 8.3, 1.0 Hz, 1H), 3.87 (s, 3H).
^{13}C-NMR (101 MHz, $CDCl_3$): δ (ppm) = 157.4, 157.3, 148.5, 135.2, 131.6, 130.4, 129.9, 129.8, 129.3, 127.5, 127.2, 126.3, 123.6, 121.4, 111.6, 55.8.
MS (EI): m/z calculated for $[M^{\bullet+}]$ $C_{16}H_{13}NO^{\bullet+}$ 235.1, found 235.1.
The analytical data match those reported in the literature.[14]

3-isopropyl-5-methyl-2-phenylquinoline (1o):

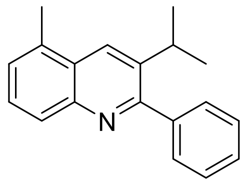

The reaction was performed according to **GP1** with (2-amino-6-methylphenyl)methanol (0.55 g, 4.0 mmol, 1.0 eq.) and 3-methyl-1-phenylbutan-1-one (0.65 g, 4.0 mmol, 1.0 eq.). After purification via Flash-Chromatography (P/EtOAc – 15/1), the product **1o** was obtained as a yellow oil (0.60 g, 2.3 mmol, 58%).

^{1}H-NMR (400 MHz, $CDCl_3$): δ (ppm) = 8.28 (s, 1H), 7.98 (d, *J* = 8.6 Hz, 1H), 7.59 – 7.42 (m, 6H), 7.35 (dt, *J* = 7.0, 1.1 Hz, 1H), 3.28 (hept, *J* = 6.8 Hz, 1H), 2.74 (s, 3H), 1.27 (d, *J* = 6.9 Hz, 6H).

^{13}C-NMR (101 MHz, $CDCl_3$): δ (ppm) = 160.0, 146.5, 141.2, 140.0, 133.9, 129.2, 128.9, 128.7, 128.4, 128.1, 127.8, 127.2, 126.9, 29.6, 24.5, 18.8.

HRMS (ESI): m/z calculated for $[M+Na]^+$ $C_{19}H_{19}NNa^+$ 284.1410, found 284.1411.

IR (Film): $\tilde{\nu}$ (cm^{-1}) = 3058, 3025, 2962, 2868, 1596, 1652, 1474, 1459, 1442, 1398, 1382, 1362, 1340, 1323, 1273, 1209, 1086, 1069, 1029, 989, 907, 814, 784, 727, 702, 662, 597, 555.

3-isopropyl-6-methyl-2-phenylquinoline (1p):

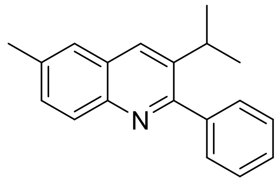

The reaction was performed according to **GP1** with (2-amino-5-methylphenyl)methanol (0.19 g, 1.4 mmol, 1.0 eq.) and 3-methyl-1-phenylbutan-1-one (0.23 g, 1.4 mmol, 1.0 eq.). After purification via Flash-Chromatography (P/EtOAc – 12/1 to 8/1), the product **1p** was obtained as a yellow oil (0.13 g, 0.49 mmol, 35%).

^{1}H-NMR (400 MHz, $CDCl_3$): δ (ppm) = 8.06 – 7.97 (m, 2H), 7.59 (s, 1H), 7.55 – 7.40 (m, 6H), 3.23 (hept, *J* = 6.9 Hz, 1H), 2.55 (s, 3H), 1.24 (d, *J* = 6.8 Hz, 6H).

^{13}C-NMR (101 MHz, $CDCl_3$): δ (ppm) = 159.6, 144.9, 141.3, 140.4, 136.2, 132.2, 131.3, 129.1, 129.0, 128.4, 128.0, 126.0, 29.4, 24.3, 21.8.

HRMS (ESI): m/z calculated for $[M+Na]^+$ $C_{19}H_{19}NNa^+$ 284.1410, found 284.1421.

IR (Solid): $\tilde{\nu}$ (cm^{-1}) = 3055, 3027, 2962, 2922, 2872, 1597, 1557, 1486, 1444, 1387, 1346, 1327, 1263, 1123, 1081, 1035, 997, 913, 821, 790, 761, 731, 700, 660, 625, 577, 536, 522, 480.

3-isopropyl-7-methyl-2-phenylquinoline (1q):

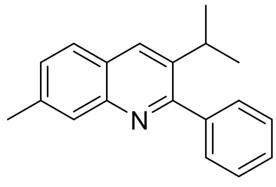

The reaction was performed according to **GP1** with (2-amino-4-methylphenyl)methanol (0.27 g, 2.0 mmol, 1.0 eq.) and 3-methyl-1-phenylbutan-1-one (0.32 g, 2.0 mmol, 1.0 eq.). After purification via Flash-Chromatography (P/EtOAc – 15/1), the product **1q** was obtained as a yellow solid (0.18 g, 0.67 mmol, 34%).

^{1}**H-NMR** (400 MHz, $CDCl_3$): δ (ppm) = 8.08 (s, 1H), 7.92 – 7.89 (m, 1H), 7.72 (d, J = 8.3 Hz, 1H), 7.55 – 7.40 (m, 5H), 7.36 (dd, J = 8.3, 1.7 Hz, 1H), 3.23 (hept, J = 6.8 Hz, 1H), 2.55 (s, 3H), 1.24 (d, J = 6.9 Hz, 6H).

^{13}**C-NMR** (101 MHz, $CDCl_3$): δ (ppm) = 160.4, 146.5, 141.3, 139.6, 139.1, 132.6, 128.9, 128.7, 128.4, 128.4, 128.0, 126.9, 126.0, 29.3, 24.3, 22.0.

HRMS (ESI): m/z calculated for $[M+Na]^+$ $C_{19}H_{19}NNa^+$ 284.1410, found 284.1410.

IR (Solid): $\tilde{\nu}$ (cm^{-1}) = 3039, 2956, 2924, 2869, 1626, 1597, 1552, 1490, 1442, 1419, 1384, 1362, 1343, 1328, 1304, 1266, 1142, 1116, 1080, 1046, 1032, 1003, 906, 885, 807, 795, 773, 749, 732, 698, 654, 603, 590, 580, 558, 524, 475.

Melting point: T (°C) = 80-81.

3-isopropyl-8-methyl-2-phenylquinoline (1r):

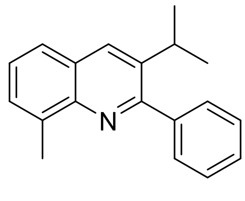

The reaction was performed according to **GP1** with (2-amino-3-methylphenyl)methanol (0.55 g, 4.0 mmol, 1.0 eq.) and 3-methyl-1-phenylbutan-1-one (0.65 g, 4.0 mmol, 1.0 eq.). After purification via Flash-Chromatography (P/Et_2O – 100/1), the product **1r** was obtained as a white solid (0.14 g, 0.54 mmol, 13%).

^{1}**H-NMR** (400 MHz, $CDCl_3$): δ (ppm) = 8.10 (s, 1H), 7.67 (d, J = 8.2 Hz, 1H), 7.63 – 7.58 (m, 2H), 7.55 – 7.37 (m, 5H), 3.34 (hept, J = 6.8 Hz, 1H), 2.80 (s, 3H), 1.27 (d, J = 6.9 Hz, 6H).

^{13}**C-NMR** (101 MHz, $CDCl_3$): δ (ppm) = 158.8, 145.5, 141.6, 140.0, 137.5, 133.1, 129.4, 128.9, 128.2, 128.0, 127.8, 126.2, 125.1, 29.3, 24.4, 18.1.

HRMS (ESI): m/z calculated for $[M+Na]^+$ $C_{19}H_{19}NNa^+$ 284.1410, found 284.1410.

IR (Solid): $\tilde{\nu}$ (cm^{-1}) = 3058, 3033, 2965, 2928, 2871, 1595, 1569, 1473, 1460, 1444, 1412, 1385, 1331, 1267, 1158, 1098, 1087, 1071, 1027, 987, 918, 890, 776, 764, 733, 702, 660, 599, 577, 514.

Melting point: T (°C) = 75-76.

6-bromo-3-isopropyl-2-phenylquinoline (1s):

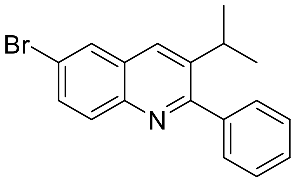

The reaction was performed according to **GP1** with (2-amino-5-bromophenyl)methanol (0.81 g, 4.0 mmol, 1.0 eq.) and 3-methyl-1-phenylbutan-1-one (0.65 g, 4.0 mmol, 1.0 eq.). After purification via Flash-Chromatography (P/EtOAc – 20/1 to 10/1), the product **1s** was obtained as a yellow oil (0.20 g, 0.61 mmol, 15%).

^{1}**H-NMR** (300 MHz, $CDCl_3$): δ (ppm) = 8.17 – 8.08 (m, 2H), 7.83 (dd, J = 8.2, 1.5 Hz, 1H), 7.67 (ddd, J = 8.5, 6.9, 1.5 Hz, 1H), 7.57 – 7.41 (m, 6H), 3.25 (hept, J = 6.8 Hz, 1H), 1.25 (d, J = 6.8 Hz, 6H).

^{13}C-NMR (101 MHz, CDCl$_3$): δ (ppm) = 161.0, 144.9, 141.6, 140.8, 132.4, 131.9, 131.2, 129.3, 129.1, 128.8, 128.5, 128.3, 120.3, 29.5, 24.2.

HRMS (ESI): m/z calculated for [M+Na]$^+$ $C_{18}H_{16}BrNNa^+$ 348.0358, found 348.0358.

IR (Solid): $\tilde{\nu}$ (cm^{-1}) = 3062, 3035, 2963, 2928, 2862, 1586, 1549, 1464, 1440, 1381, 1345, 1321, 1292, 1263, 1176, 1142, 1076, 1058, 1033, 1003, 993, 916, 846, 756, 696, 630, 613, 576, 508, 482.

7-chloro-3-isopropyl-2-phenylquinoline (1t):

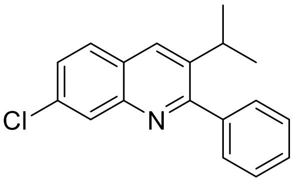

The reaction was performed according to **GP1** with (2-amino-4-chlorophenyl)methanol (0.95 g, 6.0 mmol, 1.0 eq.) and 3-methyl-1-phenylbutan-1-one (0.97 g, 6.0 mmol, 1.0 eq.). After purification via Flash-Chromatography (P/EtOAc – 15/1), the product **1t** was obtained as a pink oil (0.30 g, 1.1 mmol, 18%).

^{1}H-NMR (400 MHz, CDCl$_3$): δ (ppm) = 8.04 (d, J = 2.0 Hz, 1H), 8.02 (s, 1H), 7.68 (d, J = 8.5 Hz, 1H), 7.47 – 7.33 (m, 6H), 3.18 (hept, J = 6.9 Hz, 1H), 1.17 (d, J = 6.9 Hz, 6H).

^{13}C-NMR (101 MHz, CDCl$_3$): δ (ppm) = 161.5, 146.6, 140.9, 140.8, 134.7, 132.8, 128.8, 128.5, 128.5, 128.4, 128.3, 127.5, 126.3, 29.4, 24.2.

HRMS (ESI): m/z calculated for [M+Na]$^+$ $C_{18}H_{16}ClNNa^+$ 304.0863, found 304.0865.

IR (Film): $\tilde{\nu}$ (cm^{-1}) = 3058, 3032, 2963, 2869, 1611, 1591, 1473, 1444, 1412, 1385, 1363, 1338, 1264, 1178, 1142, 1079, 1063, 1003, 995, 923, 911, 876, 809, 792, 763, 725, 707, 697, 594, 571, 472.

6-fluoro-3-methyl-2-phenylquinoline (1u):

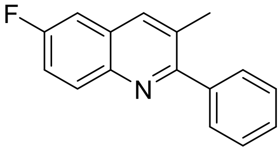

The reaction was performed according to **GP1** with (2-amino-5-fluorophenyl)methanol (0.71 g, 5.0 mmol, 1.0 eq.) and propiophenone (0.67 g, 5.0 mmol, 1.0 eq.). After purification via Flash-Chromatography (P/EtOAc – 20/1 to 10/1), the product **1u** was obtained as a yellow solid (1.14 g, 4.80 mmol, 96%).

^{1}H-NMR (400 MHz, CDCl$_3$): δ (ppm) = 8.13 (dd, J = 9.2, 5.3 Hz, 1H), 7.97 (s, 1H), 7.62 – 7.54 (m, 2H), 7.54 – 7.35 (m, 6H), 2.47 (s, 3H).

^{13}C-NMR (101 MHz, CDCl$_3$): δ (ppm) = 160.7 (d, J = 247.5 Hz), 160.0 (d, J = 2.7 Hz), 143.8, 140.6, 136.3 (d, J = 5.3 Hz), 131.9 (d, J = 9.2 Hz), 130.4, 129.0, 128.5, 128.5, 128.3 (d, J = 10.1 Hz), 119.1 (d, J = 25.8 Hz), 109.8 (d, J = 21.7 Hz), 20.8.

^{19}F-NMR (282 MHz, CDCl$_3$): δ (ppm) = -113.81 (td, J = 8.7, 5.3 Hz).

HRMS (ESI): m/z calculated for [M+H]$^+$ $C_{16}H_{13}FN^+$ 238.1027, found 238.1026.

The analytical data match those reported in the literature.[17]

methyl 2-phenylquinoline-6-carboxylate (1v):

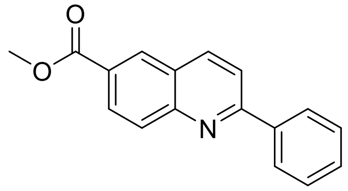

The synthesis of quinolines is performed in accordance to an adjusted literature procedure.[18] Cinnamaldehyde (0.66 g, 5.0 mmol, 1.0 eq.), methyl 4-aminobenzoate (0.76 g, 5.0 mmol, 1.0 eq.), $Pd(OAc)_2$ (0.11 g, 0.50 mmol, 10 mol%) and DMSO (20 mL) were added into a Schlenk flask and the mixture was purged with O_2 for 5 minutes. An oxygen balloon was put onto the reaction mixture. The reaction mixture was heated to 130 °C and stirred for 16 h at this temperature. The mixture was quenched by addition of H_2O (100 mL). The aqueous layer was extracted with EtOAc and the combined organic layers were dried over $MgSO_4$ and filtered. The solvents were removed at the rotary evaporator under reduced pressure. After purification via Flash-Chromatography (P/EtOAc – 40/1 to 10/1), the product **1v** was obtained as an off white solid (0.29 g, 1.1 mmol, 22%).

^{1}H-NMR (300 MHz, $CDCl_3$): δ (ppm) = 8.60 (d, J = 1.9 Hz, 1H), 8.36 – 8.27 (m, 2H), 8.24 – 8.14 (m, 3H), 7.95 (d, J = 8.7 Hz, 1H), 7.59 – 7.46 (m, 3H), 4.00 (s, 3H).

^{13}C-NMR (76 MHz, $CDCl_3$): δ (ppm) = 166.9, 159.5, 150.3, 139.2, 138.2, 130.8, 130.1, 130.0, 129.3, 129.1, 127.8, 127.8, 126.4, 119.8, 52.6.

MS (EI): m/z calculated for $[M^{\bullet+}]$ $C_{17}H_{13}NO_2^{\bullet+}$ 263.1, found 263.1.

The analytical data match those reported in the literature.[19]

ethyl 2-phenylquinoline-3-carboxylate (1w):

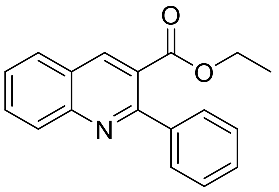

The reaction was performed according to **GP6** with 2-nitrobenzaldehyde (0.76 g, 5.0 mmol, 1.0 eq.) and ethyl 3-oxo-3-phenylpropanoate (1.91 g, 10.0 mmol, 2.0 eq.). After purification via Flash-Chromatography (P/EtOAc – 30/1 to 10/1), the product **1w** was obtained as a white solid (1.17 g, 4.23 mmol, 85%).

^{1}H-NMR (400 MHz, $CDCl_3$): δ (ppm) = 8.65 (s, 1H), 8.22 – 8.15 (m, 1H), 7.96 – 7.88 (m, 1H), 7.81 (ddd, J = 8.4, 6.9, 1.4 Hz, 1H), 7.67 – 7.62 (m, 2H), 7.59 (ddd, J = 8.1, 6.9, 1.2 Hz, 1H), 7.52 – 7.39 (m, 3H), 4.20 (q, J = 7.1 Hz, 2H), 1.08 (t, J = 7.1 Hz, 3H).

^{13}C-NMR (101 MHz, $CDCl_3$): δ (ppm) = 168.1, 158.2, 148.5, 140.9, 139.1, 131.6, 129.7, 128.7, 128.6, 128.3, 128.3, 127.3, 126.0, 125.6, 61.6, 13.8.

MS (EI): m/z calculated for $[M^{\bullet+}]$ $C_{18}H_{15}NO_2^{\bullet+}$ 277.1, found 277.1.

The analytical data match those reported in the literature.[7]

_N_-isopropyl-2-phenylquinoline-3-carboxamide (1x):

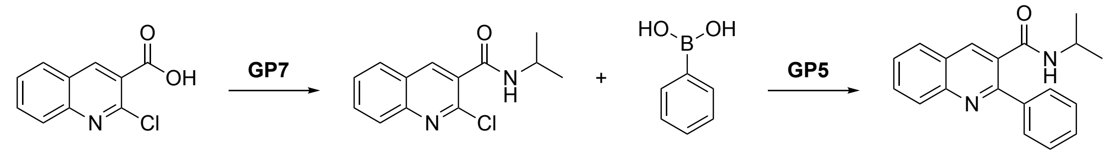

1x

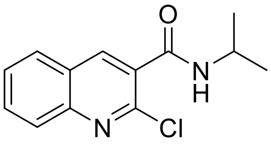

The reaction was performed according to **GP7** with 2-chloroquinoline-3-carboxylic acid (0.17 g, 0.80 mmol, 1.0 eq.) and isopropylamine (0.12 g, 2.0 mmol, 2.5 eq.). After purification via Flash-Chromatography (P/EtOAc – 1/1 to 1/4 to pure EtOAc), the quinoline 2-chloro-*N*-isopropylquinoline-3-carboxamide was obtained as white solid (0.11 g, 0.43 mmol, 54%).

^{1}H-NMR (400 MHz, $CDCl_3$): δ (ppm) = 8.49 (s, 1H), 8.01 (dq, *J* = 8.5, 0.9 Hz, 1H), 7.87 – 7.82 (m, 1H), 7.79 (ddd, *J* = 8.5, 7.0, 1.5 Hz, 1H), 7.60 (ddd, *J* = 8.1, 6.9, 1.2 Hz, 1H), 6.36 – 6.30 (d, *J* = 5.6 Hz, 1H), 4.34 (dp, *J* = 8.0, 6.6 Hz, 1H), 1.32 (d, *J* = 6.6 Hz, 6H).

^{13}C-NMR (101 MHz, $CDCl_3$): δ (ppm) = 164.2, 147.9, 145.9, 140.0, 132.0, 129.4, 128.5, 128.3, 127.9, 126.6, 42.8, 22.7.

HRMS (ESI): m/z calculated for $[M+Na]^+$ $C_{13}H_{13}N_2OClNa^+$ 271.0609, found 271.0607.

IR (Solid): $\tilde{\nu}$ (cm^{-1}) = 3253, 3061, 2972, 2928, 2871, 1644, 1617, 1540, 1486, 1456, 1397, 1365, 1324, 1286, 1207, 1173, 1157, 1135, 1036, 962, 929, 906, 869, 818, 782, 749, 713, 643, 601, 553, 523, 485.

Melting point: *T* (°C) = 166-167.

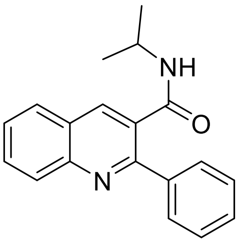

The reaction was performed according to **GP5** with 2-chloro-*N*-isopropylquinoline-3-carboxamide (0.12 g, 0.50 mmol, 1.0 eq.) and phenylboronic acid (91 mg, 0.75 mmol, 1.5 eq.). After purification via Flash-Chromatography (P/EtOAc – 1/1), the product **1x** was obtained as a yellow solid (0.13 g, 0.45 mmol, 91%).

^{1}H-NMR (400 MHz, $CDCl_3$): δ (ppm) = 8.53 (s, 1H), 8.20 – 8.11 (m, 1H), 7.88 (dd, *J* = 8.1, 1.4 Hz, 1H), 7.81 – 7.68 (m, 3H), 7.57 (ddd, *J* = 8.1, 6.9, 1.2 Hz, 1H), 7.52 – 7.45 (m, 3H), 5.22 (d, *J* = 7.7 Hz, 1H), 4.14 – 4.01 (m, 1H), 0.92 (d, *J* = 6.6 Hz, 6H).

^{13}C-NMR (76 MHz, $CDCl_3$): δ (ppm) = 167.2, 156.3, 148.2, 139.7, 138.0, 131.1, 129.8, 129.5, 129.3, 129.1, 128.8, 128.2, 127.3, 126.4, 42.1, 22.2.

HRMS (ESI): m/z calculated for $[M+Na]^+$ $C_{19}H_{18}N_2ONa^+$ 313.1311, found 313.1322.

IR (Solid): $\tilde{\nu}$ (cm^{-1}) = 3276, 3055, 2970, 2927, 2872, 1635, 1539, 1485, 1456, 1415, 1364, 1349, 1297, 1279, 1266, 1159, 1146, 1132, 1076, 1017, 953, 915, 860, 824, 798, 766, 745, 719, 693, 592, 573, 555, 510, 477.

Melting point: *T* (°C) = 176-178.

(2-phenylquinolin-3-yl)(pyrrolidin-1-yl)methanone (1y):

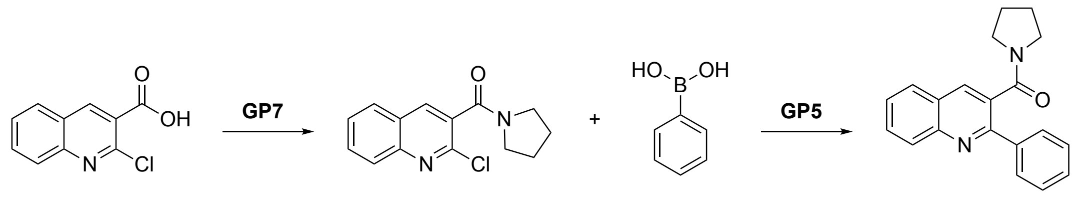

1y

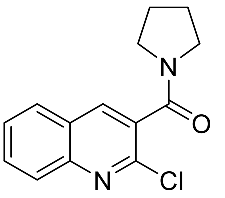

The reaction was performed according to **GP7** with 2-chloroquinoline-3-carboxylic acid (0.25 g, 1.2 mmol, 1.0 eq.) and pyrrolidine (0.21 g, 3.0 mmol, 2.5 eq.). After purification via Flash-Chromatography (P/EtOAc – 1/1 to 1/4 to pure EtOAc), the quinoline (2-chloroquinolin-3-yl)(pyrrolidin-1-yl)methanone was obtained as brown solid (0.18 g, 0.69 mmol, 57%).

^{1}H-NMR (599 MHz, $CDCl_3$): δ (ppm) = 8.14 (s, 1H), 8.06 – 8.01 (m, 1H), 7.83 (m, 1H), 7.78 (ddd, J = 8.5, 7.0, 1.5 Hz, 1H), 7.60 (ddd, J = 8.1, 6.9, 1.2 Hz, 1H), 3.72 (t, J = 7.0 Hz, 2H), 3.32 – 3.24 (m, 2H), 2.04 – 1.98 (m, 2H), 1.93 (m, 2H).

^{13}C-NMR (151 MHz, $CDCl_3$): δ (ppm) = 165.1, 147.7, 146.2, 136.6, 131.5, 131.4, 128.7, 127.9, 127.8, 126.7, 48.3, 46.0, 26.1, 24.6.

HRMS (ESI): m/z calculated for $[M+Na]^+$ $C_{14}H_{13}N_2OClNa^+$ 283.0609, found 283.0606.

IR (Solid): $\tilde{\nu}$ (cm^{-1}) = 3060, 2972, 2955, 2928, 2875, 1622, 1590, 1561, 1489, 1458, 1432, 1370, 1330, 1305, 1255, 1231, 1193, 1164, 1136, 1115, 1049, 1026, 921, 907, 862, 838, 778, 751, 732, 639, 597, 562, 523, 480, 465.

Melting point: T (°C) = 128-129.

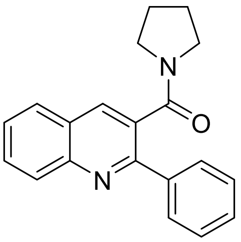

The reaction was performed according to **GP5** with (2-chloroquinolin-3-yl)(pyrrolidin-1-yl)methanone (0.16 g, 0.60 mmol, 1.0 eq.) and phenylboronic acid (0.11 g, 0.90 mmol, 1.5 eq.). No further purification was necessary and the product **1y** was obtained as a yellow solid (0.17 g, 0.55 mmol, 92%).

^{1}H-NMR (599 MHz, $CDCl_3$): δ (ppm) = 8.29 (s, 1H), 8.21 (d, J = 8.5 Hz, 1H), 7.92 – 7.90 (m, 2H), 7.87 (m, 2H), 7.78 (ddd, J = 8.4, 6.9, 1.4 Hz, 1H), 7.58 (ddd, J = 8.1, 6.9, 1.2 Hz, 1H), 7.49 – 7.42 (m, 3H), 3.49 (s, 2H), 2.76 (s, 2H), 1.68 (s, 2H), 1.49 (s, 2H).

^{13}C-NMR (151 MHz, $CDCl_3$): δ (ppm) = 168.3, 155.1, 136.1, 131.1, 130.8, 129.6, 129.4, 129.0, 128.8, 128.7, 127.9, 127.3, 126.6, 126.2, 47.8, 45.9, 25.7, 24.3.

HRMS (ESI): m/z calculated for $[M+Na]^+$ $C_{20}H_{18}N_2ONa^+$ 325.1311, found 325.1311.

IR (Solid): $\tilde{\nu}$ (cm^{-1}) = 3055, 3000, 2946, 2928, 2868, 1615, 1594, 1554, 1483, 1466, 1442, 1402, 1373, 1334, 1315, 1259, 1152, 1074, 1015, 964, 925, 880, 867, 834, 804, 770, 727, 718, 695, 617, 581, 563, 523, 481.

Melting point: T (°C) = 106-107.

2,3-diphenylquinoline (1z):

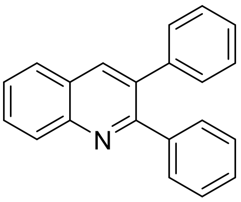

The reaction was performed according to **GP4** with 3-bromo-2-chloroquinoline (1.21 g, 5.00 mmol, 1.7 eq.) and phenylboronic acid (0.73 g, 6.0 mmol, 1.0 eq.). After purification via Flash-Chromatography (P/EtOAc – 10/1), the product **1z** was obtained as a off white solid (0.44 g, 1.6 mmol, 52%).

^{1}H-NMR (400 MHz, $CDCl_3$): δ(ppm) = 8.19 (d, J = 8.8 Hz, 1H), 8.15 (s, 1H), 7.84 (d, J = 8.2 Hz, 1H), 7.71 (ddd, J = 8.4, 6.9, 1.5 Hz, 1H), 7.54 (ddd, J = 8.1, 6.8, 1.2 Hz, 1H), 7.46 – 7.41 (m, 2H), 7.29 – 7.20 (m, 8H).

^{13}C-NMR (101 MHz, $CDCl_3$): δ(ppm) = 158.5, 147.4, 140.6, 140.1, 137.7, 134.7, 130.2, 129.9, 129.7, 129.6, 128.4, 128.1, 128.1, 127.6, 127.4, 127.3, 126.9.

HRMS (ESI): m/z calculated for $[M+H]^+$ $C_{21}H_{16}N^+$ 282.1277, found 282.1279.

The analytical data match those reported in the literature.[20]

2-isopropyl-3-phenylquinoline (1aa):

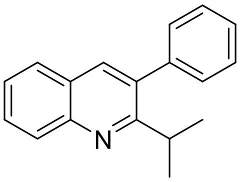

The reaction was performed according to **GP1** with (2-aminophenyl)methanol (0.49 g, 4.0 mmol, 1.0 eq.) and 3-methyl-1-phenylbutan-2-one (0.65 g, 4.0 mmol, 1.0 eq.). After purification via Flash-Chromatography (P/Et_2O – 20/1), the product **1aa** was obtained as a white solid (0.47 g, 1.9 mmol, 47%).

^{1}H-NMR (400 MHz, $CDCl_3$): δ(ppm) = 8.12 (dq, J = 8.5, 0.8 Hz, 1H), 7.92 (s, 1H), 7.77 (dd, J = 8.1, 1.4 Hz, 1H), 7.69 (ddd, J = 8.4, 6.9, 1.5 Hz, 1H), 7.54 – 7.36 (m, 6H), 3.39 (hept, J = 6.7 Hz, 1H), 1.31 (d, J = 6.7 Hz, 6H).

^{13}C-NMR (101 MHz, $CDCl_3$): δ(ppm) = 165.6, 147.7, 140.4, 136.4, 135.2, 129.5, 129.1, 129.1, 128.4, 127.5, 127.5, 126.6, 126.0, 32.3, 22.6.

MS (EI): m/z calculated for $[M^{\bullet+}]$ $C_{18}H_{17}N^{\bullet+}$ 247.1, found 247.1.

The analytical data match those reported in the literature.[21]

ethyl 2-(*tert*-butyl)quinoline-3-carboxylate (1ab):

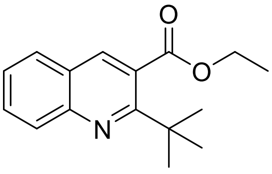

The reaction was performed according to **GP6** with 2-nitrobenzaldehyde (0.30 g, 2.0 mmol, 1.0 eq.) and ethyl 4,4-dimethyl-3-oxopentanoate (1.03 g, 6.00 mmol, 3.0 eq.). After purification via Flash-Chromatography (P/EtOAc – 25/1 to 20/1), the product **1ab** was obtained as a white solid (0.25 g, 0.99 mmol, 49%).

^{1}H-NMR (599 MHz, $CDCl_3$): δ (ppm) = 8.15 (s, 1H), 8.05 (dd, J = 8.5, 1.0 Hz, 1H), 7.80 – 7.75 (m, 1H), 7.72 (ddd, J = 8.4, 6.9, 1.4 Hz, 1H), 7.51 (ddd, J = 8.0, 6.9, 1.2 Hz, 1H), 4.44 (q, J = 7.1 Hz, 2H), 1.54 (d, J = 0.5 Hz, 9H), 1.44 (t, J = 7.2 Hz, 3H).
^{13}C-NMR (151 MHz, $CDCl_3$): δ (ppm) = 170.5, 164.6, 147.2, 137.3, 130.5, 129.5, 127.5, 127.0, 126.7, 125.0, 61.9, 40.1, 30.2, 14.2.
HRMS (ESI): m/z calculated for $[M+Na]^+$ $C_{16}H_{19}NO_2Na^+$ 280.1308, found 280.1308.
IR (Solid): $\tilde{\nu}$ (cm^{-1}) = 2958, 2908, 2866, 1714, 1618, 1590, 1555, 1483, 1450, 1415, 1391, 1364, 1318, 1283, 1268, 1228, 1202, 1154, 1121, 1047, 1015, 962, 933, 865, 796, 755, 732, 616, 594, 569, 481.
Melting point: T (°C) = 59-60.

ethyl 2-isopropylquinoline-3-carboxylate (1ac):

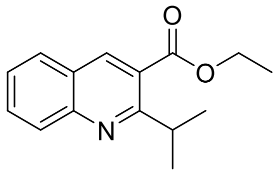

The reaction was performed according to **GP6** with 2-nitrobenzaldehyde (0.30 g, 2.0 mmol, 1.0 eq.) and ethyl 4-methyl-3-oxopentanoate (0.95 g, 6.0 mmol, 3.0 eq.). After purification via Flash-Chromatography (P/Et_2O – 20/1), the product **1ac** was obtained as a white solid (0.26 g, 1.1 mmol, 53%).
^{1}H-NMR (300 MHz, $CDCl_3$): δ (ppm) = 8.57 (s, 1H), 8.07 (d, J = 8.4 Hz, 1H), 7.83 (dd, J = 8.1, 1.5 Hz, 1H), 7.75 (ddd, J = 8.5, 6.9, 1.5 Hz, 1H), 7.51 (ddd, J = 8.1, 6.9, 1.2 Hz, 1H), 4.44 (q, J = 7.2 Hz, 2H), 3.97 (hept, J = 6.7 Hz, 1H), 1.45 (t, J = 7.2 Hz, 3H), 1.40 (d, J = 6.7 Hz, 6H).
^{13}C-NMR (76 MHz, $CDCl_3$): δ (ppm) = 167.5, 165.9, 148.8, 139.2, 131.2, 129.2, 128.3, 126.5, 125.6, 124.3, 61.6, 33.1, 22.5, 14.4.
MS (EI): m/z calculated for $[M^{\bullet+}]$ $C_{15}H_{17}NO_2^{\bullet+}$ 243.1, found 243.1.
The analytical data match those reported in the literature.[22]

***N,N*-diethyl-2-methylquinoline-3-carboxamide (1ad):**

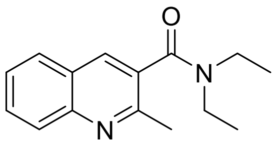

The reaction was performed according to **GP6** with 2-nitrobenzaldehyde (0.30 g, 2.0 mmol, 1.0 eq.) and *N,N*-diethyl-3-oxobutanamide (0.94 g, 6.0 mmol, 3.0 eq.). After purification via Flash-Chromatography (P/EtOAc – 1/1 to 1/2), the product **1ad** was obtained as a yellow oil (0.11 g, 0.47 mmol, 23%).
^{1}H-NMR (400 MHz, $CDCl_3$): δ (ppm) = 8.03 (d, J = 8.5 Hz, 1H), 7.95 (s, 1H), 7.78 (d, J = 8.2 Hz, 1H), 7.72 (ddd, J = 8.4, 6.9, 1.5 Hz, 1H), 7.52 (ddd, J = 8.1, 6.9, 1.2 Hz, 1H), 3.56 (s, 2H), 3.17 (d, J = 7.4 Hz, 2H), 2.70 (s, 3H), 1.32 (t, J = 7.1 Hz, 2H), 1.06 (t, J = 7.1 Hz, 2H).
^{13}C-NMR (101 MHz, $CDCl_3$): δ (ppm) = 169.2, 155.2, 147.7, 132.9, 131.0, 130.2, 128.8, 127.7, 126.6, 126.0, 43.0, 39.3, 23.1, 14.2, 12.9.
HRMS (ESI): m/z calculated for $[M+Na]^+$ $C_{15}H_{18}N_2ONa^+$ 265.1311, found 265.1319.

IR (Film): $\tilde{\nu}$ (cm^{-1}) = 3444, 3257, 3054, 2974, 2934, 2871, 1618, 1567, 1480, 1459, 1434, 1407, 1381, 1364, 1320, 1280, 1217, 1169, 1125, 1059, 943, 921, 858, 808, 786, 753, 657, 621, 574, 493, 481.

phenyl(2-(trifluoromethyl)quinolin-3-yl)methanone (1ae):

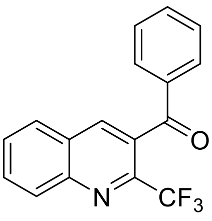

The reaction was performed according to **GP1** with (2-aminophenyl)methanol (0.12 g, 1.0 mmol, 1.0 eq.) and 4,4,4-trifluoro-1-phenylbutane-1,3-dione (0.42 g, 2.0 mmol, 2.0 eq.). After purification via Flash-Chromatography (P/EtOAc – 5/1), the product **1ae** was obtained as a yellow solid (0.11 g, 0.37 mmol, 37%).

^{1}H-NMR (300 MHz, $CDCl_3$): δ (ppm) = 8.35 – 8.29 (m, 1H), 8.27 (s, 1H), 7.98 – 7.89 (m, 2H), 7.88 – 7.81 (m, 2H), 7.75 (ddd, *J* = 7.9, 6.9, 1.2 Hz, 1H), 7.70 – 7.61 (m, 1H), 7.50 (td, *J* = 7.8, 1.7 Hz, 2H).

^{13}C-NMR (76 MHz, $CDCl_3$): δ (ppm) = 193.7, 147.0, 137.5, 136.5, 134.3, 132.1, 130.8, 130.4, 130.3, 129.8, 128.9, 128.1, 127.4, 119.7.

^{19}F{^{1}H} NMR (282 MHz, $CDCl_3$) δ (ppm) = -62.8.

MS (EI): m/z calculated for [$M^{\bullet+}$] $C_{17}H_{10}F_3NO^{\bullet+}$ 301.1, found 301.1.

The analytical data match those reported in the literature.[22]

11*H*-indeno[1,2-*b*]quinoline (1af):

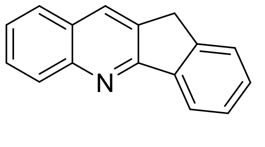

The reaction was performed according to **GP2** with 2-aminobenzaldehyde (0.49 g, 4.0 mmol, 1.0 eq.) and 2,3-dihydroinden-1-one (0.53 g, 4.0 mmol, 1.0 eq.). After purification via Flash-Chromatography (P/EtOAc – 10/1 to 5/1), the product **1af** was obtained as a white solid (0.73 g, 3.4 mmol, 84%).

^{1}H-NMR (400 MHz, $CDCl_3$): δ (ppm) = 8.33 – 8.29 (m, 1H), 8.24 – 8.16 (m, 2H), 7.86 – 7.78 (m, 1H), 7.75 – 7.66 (m, 1H), 7.64 – 7.58 (m, 1H), 7.51 (m, 3H), 4.04 (s, 2H).

^{13}C-NMR (101 MHz, $CDCl_3$): δ (ppm) = 161.9, 148.3, 145.2, 140.6, 134.7, 131.3, 130.1, 129.3, 128.9, 127.9, 127.7, 127.5, 125.8, 125.6, 122.2, 34.2.

MS (EI): m/z calculated for [$M^{\bullet+}$] 217.1, found 217.1.

The analytical data match those reported in the literature.[3]

5,6-dihydrobenzo[*c*]acridine (1ag):

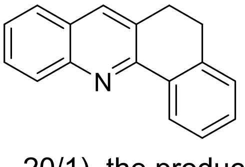

The reaction was performed according to **GP2** with 2-aminobenzaldehyde (0.36 g, 3.0 mmol, 1.0 eq.) and 3,4-dihydro-2H-naphthalen-1-one (0.44 g, 3.0 mmol, 1.0 eq.). After purification via Flash-Chromatography (P/EtOAc – 20/1), the product **1ag** was obtained as an off-white solid (0.48 g, 2.1 mmol, 70%).

^{1}H-NMR (400 MHz, $CDCl_3$): δ (ppm) = 8.59 (dd, J = 7.7, 1.6 Hz, 1H), 8.14 (d, J = 8.5 Hz, 1H), 7.92 (s, 1H), 7.75 (d, J = 8.2 Hz, 1H), 7.65 (ddd, J = 8.4, 6.8, 1.5 Hz, 1H), 7.52 – 7.33 (m, 3H), 7.28 (dd, J = 7.1, 1.4 Hz, 1H), 3.18 – 3.09 (m, 2H), 3.06 – 2.98 (m, 2H).

^{13}C-NMR (101 MHz, $CDCl_3$): δ (ppm) = 153.5, 147.8, 139.5, 134.9, 133.8, 130.7, 129.8, 129.6, 128.8, 128.1, 128.0, 127.5, 127.1, 126.2, 126.2, 29.0, 28.6.

MS (EI): m/z calculated for [$M^{\bullet+}$] $C_{16}H_{13}NO^{\bullet+}$ 231.1, found 231.1.

The analytical data match those reported in the literature.[23]

19-azatetracyclo[9.8.0.02,7.013,18]nonadeca-1(19),2,4,6,11,13,15,17-octaene (1ah):

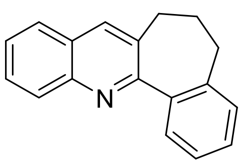

The reaction was performed according to **GP1** with (2-aminophenyl)methanol (0.25 g, 2.0 mmol, 1.0 eq.) and 6,7,8,9-tetrahydrobenzo[7]annulen-5-one (0.32 g, 2.0 mmol, 1.0 eq.). After purification via Flash-Chromatography (P/Et_2O – 20/1), the product **1ah** was obtained as a colorless resin (0.41 g, 1.7 mmol, 83%).

^{1}H-NMR (400 MHz, $CDCl_3$): δ (ppm) = 8.18 (d, J = 8.4 Hz, 1H), 7.97 (s, 1H), 7.86 (dd, J = 7.3, 1.7 Hz, 1H), 7.81 (dd, J = 8.1, 1.5 Hz, 1H), 7.69 (ddd, J = 8.4, 6.9, 1.5 Hz, 1H), 7.53 (ddd, J = 8.1, 6.8, 1.3 Hz, 1H), 7.47 – 7.37 (m, 2H), 2.70 (t, J = 7.0 Hz, 2H), 2.61 (t, J = 7.1 Hz, 2H), 2.24 (p, J = 7.0 Hz, 2H).

^{13}C-NMR (76 MHz, $CDCl_3$): δ (ppm) = 160.5, 147.5, 140.4, 139.3, 134.7, 133.4, 129.6, 129.3, 129.1, 128.9, 128.5, 127.9, 127.2, 127.0, 126.3, 31.9, 31.0, 30.7.

MS (EI): m/z calculated for [$M^{\bullet+}$] $C_{18}H_{15}N^{\bullet+}$ 245.1, found 245.1.

The analytical data match those reported in the literature.[24]

3-isopropyl-2-phenylbenzo[*g*]quinoline (1ai):

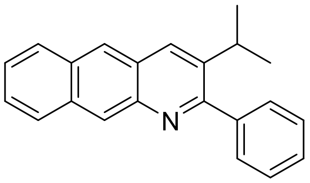

The reaction was performed according to **GP1** with (3-aminonaphthalen-2-yl)methanol (0.31 g, 1.8 mmol, 1.0 eq.) and 3-methyl-1-phenylbutan-1-one (0.29 g, 1.8 mmol, 1.0 eq.). After purification via Flash-Chromatography (P/Et_2O – 12/1 to 8/1), the product **1ai** was obtained as a yellow solid (0.26 g, 0.86 mmol, 48%).

^{1}H-NMR (400 MHz, $CDCl_3$): δ (ppm) = 8.72 (s, 1H), 8.39 (s, 1H), 8.27 (s, 1H), 8.10 – 7.99 (m, 2H), 7.62 – 7.57 (m, 2H), 7.55 – 7.43 (m, 5H), 3.29 (hept, J = 6.8 Hz, 1H), 1.28 (d, J = 6.8 Hz, 6H).

^{13}C-NMR (101 MHz, $CDCl_3$): δ (ppm) = 162.1, 143.2, 141.2, 140.3, 133.8, 132.6, 132.0, 128.8, 128.7, 128.4, 128.3, 128.1, 127.3, 126.5, 126.0, 125.9, 125.7, 29.6, 24.2.

HRMS (APCI): m/z calculated for $[M+H]^+$ $C_{22}H_{20}N^+$ 298.1590, found 298.1584.

IR (Solid): $\tilde{\nu}$ (cm^{-1}) = 3055, 3018, 2960, 2925, 2871, 1611, 1585, 1554, 1517, 1441, 1402, 1361, 1331, 1317, 1290, 1274, 1242, 1196, 1179, 1072, 1002, 993, 957, 907, 885, 861, 793, 769, 743, 698, 590, 572, 541, 481, 465.
Melting point: T (°C) = 93-95.

6-methoxy-3-methyl-2-phenylquinoline (1aj):

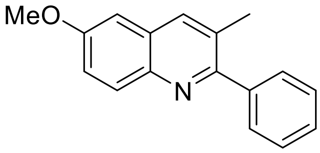

The reaction was performed according to **GP1** with (2-amino-5-methoxyphenyl)methanol (0.77 g, 5.0 mmol, 1.0 eq.) and propiophenone (0.67 g, 5.0 mmol, 1.0 eq.). After purification via Flash-Chromatography (P/Et_2O – 20/1), the product **1aj** was obtained as a yellow oil (1.21 g, 4.86 mmol, 97%).
^{1}H-NMR (400 MHz, $CDCl_3$): δ (ppm) = 8.02 (d, J = 9.2 Hz, 1H), 7.90 (s, 1H), 7.62 – 7.54 (m, 2H), 7.52 – 7.37 (m, 3H), 7.32 (dd, J = 9.2, 2.8 Hz, 1H), 7.04 (d, J = 2.8 Hz, 1H), 3.93 (s, 3H), 2.44 (s, 3H).
^{13}C-NMR (101 MHz, $CDCl_3$): δ (ppm) = 158.1, 157.9, 142.9, 141.1, 135.7, 130.9, 129.5, 129.0, 128.6, 128.4, 128.1, 121.5, 104.3, 55.6, 20.7.
HRMS (ESI): m/z calculated for $[M+H]^+$ $C_{17}H_{16}NO^+$ 250.1226, found 250.1227.
The analytical data match those reported in the literature.[17]

3-(benzenesulfonyl)-2-phenylquinoline (1ak):

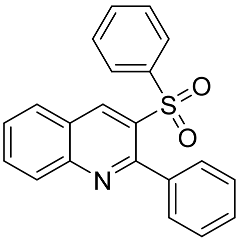

The reaction was performed according to **GP6** with 2-nitrobenzaldehyde (0.11 g, 0.70 mmol, 1.0 eq.) and 2-(benzenesulfonyl)-1-phenylethanone (0.36 g, 1.4 mmol, 2.0 eq.). After purification via Flash-Chromatography (P/Et_2O – 4/1 to 2/1), the product **1ak** was obtained as a yellow solid (85 mg, 0.25 mmol, 35%).
^{1}H-NMR (400 MHz, $CDCl_3$): δ (ppm) = 9.32 (s, 1H), 8.17 (dt, J = 8.5, 0.9 Hz, 1H), 8.10 (dd, J = 8.1, 1.5 Hz, 1H), 7.90 (ddd, J = 8.5, 6.9, 1.5 Hz, 1H), 7.73 (ddd, J = 8.1, 6.9, 1.1 Hz, 1H), 7.46 – 7.34 (m, 2H), 7.32 – 7.14 (m, 8H).
^{13}C-NMR (101 MHz, $CDCl_3$): δ (ppm) = 157.3, 148.9, 140.0, 139.3, 138.3, 134.8, 133.1, 129.8, 129.6, 129.2, 128.8, 128.7, 128.3, 128.1, 127.7, 125.9.
MS (EI): m/z calculated for $[M^{\bullet +}]$ 345.1, found 345.1.
The analytical data match those reported in the literature.[25]

3-cyclopentylidene-2,3-dihydro-1*H*-cyclopenta[*b*]quinoline (1al):

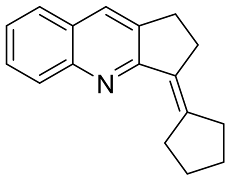

The reaction was performed according to **GP1** with (2-aminophenyl)methanol (0.49 g, 4.0 mmol, 1.0 eq.) and cyclopentanone (0.34 g, 4.0 mmol, 1.0 eq.). After purification via Flash-Chromatography (P/Et_2O – 50/1), the product **1al** was obtained as a yellow solid (0.15 g, 0.63 mmol, 32%).

^{1}H-NMR (599 MHz, $CDCl_3$): δ (ppm) = 8.03 (d, *J* = 8.4 Hz, 1H), 7.82 (d, *J* = 1.1 Hz, 1H), 7.67 (dd, *J* = 8.0, 1.5 Hz, 1H), 7.57 (ddd, *J* = 8.4, 6.8, 1.5 Hz, 1H), 7.39 (ddd, *J* = 8.0, 6.8, 1.2 Hz, 1H), 3.21 – 3.14 (m, 2H), 3.13 – 3.09 (m, 2H), 2.82 – 2.75 (m, 2H), 2.46 – 2.39 (m, 2H), 1.87 – 1.81 (m, 2H), 1.80 – 1.74 (m, 2H).

^{13}C-NMR (151 MHz, $CDCl_3$): δ (ppm) = 163.3, 148.5, 146.7, 138.5, 130.1, 129.9, 129.6, 128.1, 127.3, 126.9, 125.3, 34.1, 32.8, 29.0, 27.6, 27.5, 25.9.

HRMS (ESI): m/z calculated for $[M+Na]^+$ $C_{17}H_{17}NaN^+$ 258.1253, found 258.1253.

IR (Solid): $\tilde{\nu}$ (cm^{-1}) = 3052, 2955, 2930, 2866, 2818, 1607, 1557, 1495, 1442, 1395, 1318, 1275, 1145, 1073, 1003, 907, 885, 861, 794, 769, 741, 697, 612, 572, 466.

Melting point: *T* (°C) = 114-115.

3. Reaction Optimization

The reaction was initially performed with the HCl salt of quinoline **1a HCl**, according to our previous work regarding the hydrogenation of quinolines to tetrahydroquinolines.[26] Herein was observed that for quinoline **1a** indole formation took part as the major product in contrast to the expected tetrahydroquinoline formation – see below. This finding let us to believe that it is possible to access a wider range of disubstituted indoles from quinoline starting material in comparison to the non-protonated substrates, where only a few examples could be realized.[5,26]

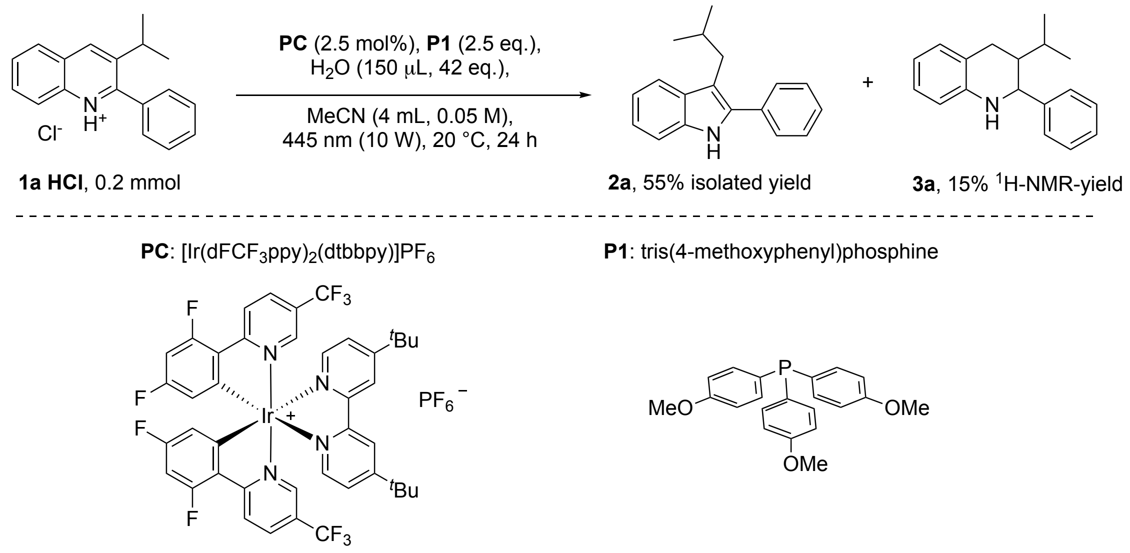

We also went back and subjected quinoline **1a** to the previously found non-acidic reaction conditions from our initial work regarding the water activation.[5] As can be seen below the reaction only proceed towards a conversion of 28% and furthermore the undesired tetrahydroquinoline product was predominantly formed. Therefore we chose to keep the acidic reaction conditions.

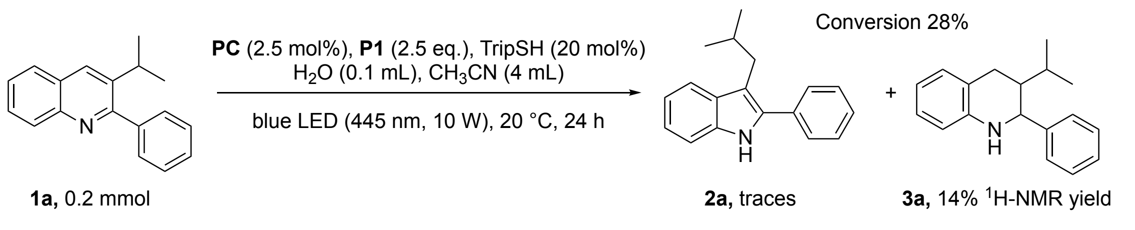

The used quinoline HCl salt was synthesized according to our previously reported method:[26]

Quinoline was dissolved in THF (0.2 M). Then, 3.0 equivalents of HCl (2.0 M in Et_2O) were slowly added to the solution at 0 °C (ice bath). The formed precipitate was filtered and washed with THF and Et_2O to afford the respective quinoline hydrochloride.

The reaction was performed after our previously reported method:[26]

1a HCl (56.8 mg, 0.200 mmol, 1.0 eq.), **PC** (5.6 mg; 5.0 µmol; 2.5 mol%) and **P1** (176 mg, 0.500 mmol, 2.5 eq.) were added into the reaction vessel. The mixture was put under a positive argon flow. Dry MeCN (4.0 ml; 0.05 mol/L) and water (150 µL; 42 eq.) were added and the mixture was purged with argon for 5 min. The reaction mixture was stirred for 24 h at 20 °C and irradiated with blue light (LED; 445 nm, 10 W). The reaction was washed into a round bottom flask with DCM and quenched by addition of sat. aq. Na_2CO_3 solution. The aq. layer was extracted with DCM and the org. layer was dried over $MgSO_4$. The solvents were removed at the rotary evaporator under reduced pressure. The reaction showed an ^{1}H-NMR-yield of 68% for **2a** and 15% for **3a** against CH_2Br_2 as internal standard. After purification via Flash-Chromatography (P/EtOAc – 30/1), the product **2a** was obtained as a white solid (27.4 mg, 0.110 mmol, 55%).

Initial screening of the reaction conditions with **1a HCl** revealed **P2** as phosphine to form selectively **2a**, shown below. Unfortunately, these conditions proved to be not as general as we hoped and still had flaws, we wanted to address for a more convenient handling of the reaction:

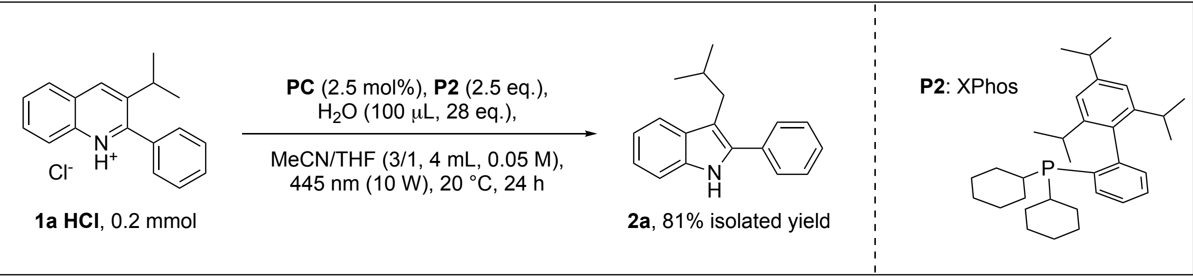

1. Preparation of the HCl-salt from the quinoline, which means a further precipitation step and pre-functionalization in the synthesis of the indoles from quinolines.
2. The usage of XPhos as selective parameter in our reaction in comparison to other readily available phosphines.
    - The usage of XPhos proved to work best for **1a HCl** with a solvent mixture of MeCN and THF due to insolubility in pure MeCN, which results in a more inconvenient reaction preparation.
    - XPhos has in comparison to other viable phosphines in our water activation system a high molecular weight resulting in a higher waist production.

Addressing our first concern, we tried out other acids. For this we used quinoline **1w**, which also initially showed indole formation under acidic conditions employed as the HCl salt – **1w HCl**.

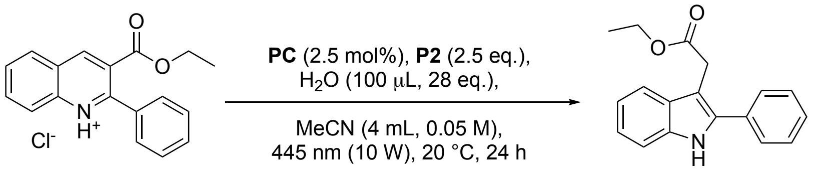

1w HCl, 0.2 mmol — **2w**, 49% ^{1}H-NMR yield

We thought quinoline **1w** to be an ideal substrate to test other acids due to possible acid catalyzed hydrolysis of the ethyl ester as side reaction. Therefore, the best reaction conditions may prove to be also applicable for acid sensitive substrates. As can be seen below, the usage of pTsOH monohydrate proved to be the best. It was also no difference between the phosphines **P2** and **P3** observed. **P3** was tested in this case, since previous investigations showed an improved yield when employing this phosphine for this specific substrate.

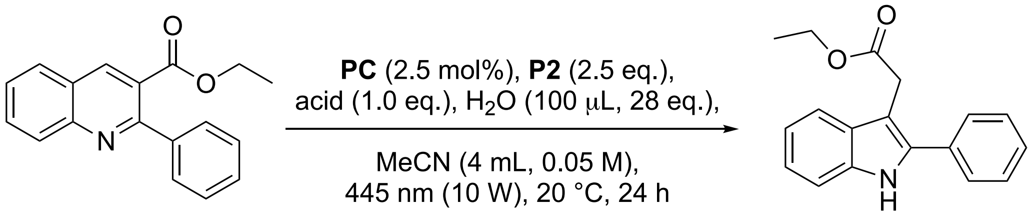

1w, 0.2 mmol — **2w**

Entry	Acid	**2a** Yield[a] / %
1	Benzoic Acid	<5
2	TFA	33
3	pTsOH monohydrate	60
3a	**P3** (p-$CF_3C_6H_4$)$_3$P for **P2**	60
4	Tf_2NH	32
5	MsOH	58
6	TfOH	33
7	$(PhO)_2PO_2H$	29

[a] Determined by ^{1}H-NMR with CH_2Br_2 as internal standard @ 0.2 mmol, isolated yield in parentheses.

When switching back to our standard substrate **1a** not a full conversion was observed. Addressing the second point of concern regarding the reaction design we retried other phosphines in the reaction. It was found that phosphine **P1** worked best with pTsOH monohydrate as acid.

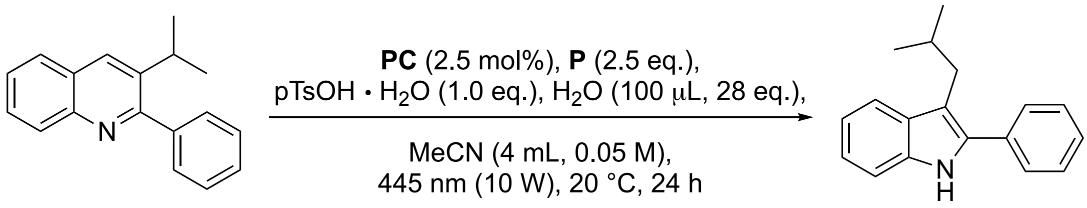

1a, 0.2 mmol **2a**

Entry	Phosphine **P**	**2a** Yield[a] / %	Conversion %
1	**P2** XPhos	45	50
2	**P1** $(p\text{-}MeOC_6H_4)_3P$	>99 (86)	Full conversion
3	$PMePh_2$	44	90
4[b]	BrettPhos	–	Full conversion
5	RuPhos	Traces	46
6	SPhos	Traces	31

[a] Determined by ^{1}H-NMR with CH_2Br_2 as internal standard @ 0.2 mmol, isolated yield in parentheses.

[b] Formation of the dimer side product (rearranged indole, followed by C-H activation in 4-position of the quinoline).

For the further optimization dried pTsOH was used to create accurate reaction conditions regarding the acid and water amount employed in the reaction. It was found that the usage of 1.0 eq. of acid proved to be best. It can be stated that the reaction conditions are stable in regard of an acid amount ±0.25 eq. of pTsOH.

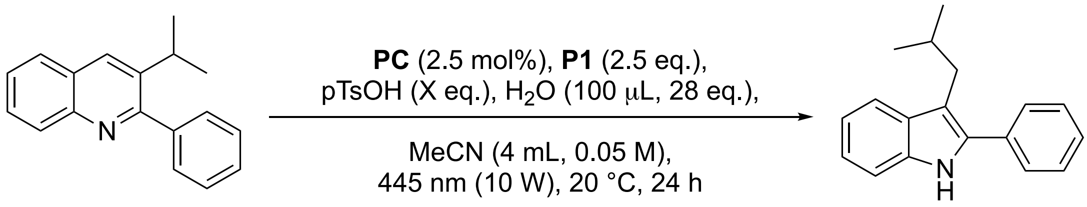

1a, 0.2 mmol **2a**

Entry	n(pTsOH)	**2a** Yield[a] / %	Conversion %
1	Standard conditions 0.20 mmol, 1.00 eq	>99 (86)	Full conversion
2	0.05 mmol, 0.25	16	35
3	0.10 mmol, 0.50	46	53
4	0.15 mmol, 0.75	91	Full conversion
5	0.25 mmol, 1.25	88	Full conversion
6	0.30 mmol, 1.50	85	Full conversion
7	0.40 mmol, 2.00	82	Full conversion

[a] Determined by ^{1}H-NMR with CH_2Br_2 as internal standard @ 0.2 mmol, isolated yield in parentheses.

For further optimization the amount of phosphine **P1** was investigated. The usage of 2.5 eq. proved to work best.

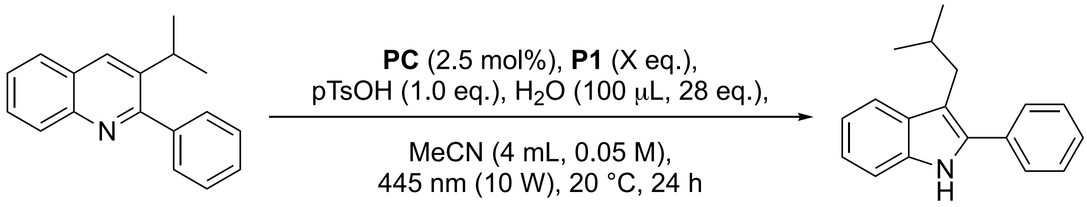

1a, 0.2 mmol **2a**

Entry	n(**P**) / mmol; eq.	**2a** Yield[a] / %	Conversion %
1	Standard conditions 0.5 mmol, 2.5 eq.	>99 (86)	Full conversion
2	0.3 mmol, 1.5 eq.	78	Full conversion
3	0.4 mmol, 2.0 eq.	90	Full conversion
4	0.6 mmol, 3.0 eq.	93	Full conversion

[a] Determined by ^{1}H-NMR with CH_2Br_2 as internal standard @ 0.2 mmol, isolated yield in parentheses.

The used amount of water proves to be quite stable within the reaction conditions, giving high yields for 75 to 125 µL.

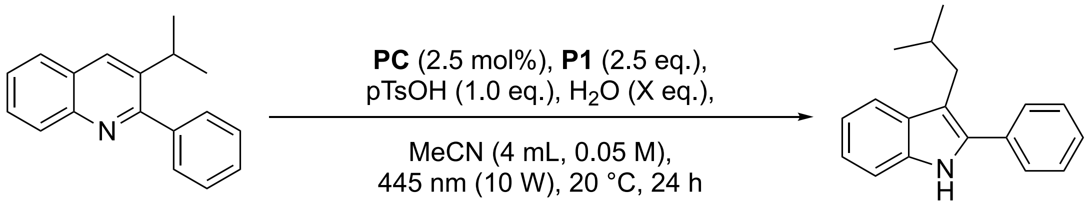

1a, 0.2 mmol

2a

Entry	V(H_2O)	**2a** Yield[a] / %	Conversion %
1	Standard conditions 100 µL, 28 eq.	>99 (86)	Full conversion
2	25 µL, 7 eq.	86	Full conversion
3	50 µL, 14 eq.	88	Full conversion
4	75 µL, 21 eq.	>99	Full conversion
5	125 µL, 35 eq.	>99	Full conversion
6	150 µL, 42 eq.	92	Full conversion

[a] Determined by ^{1}H-NMR with CH_2Br_2 as internal standard @ 0.2 mmol, isolated yield in parentheses.

The investigation of the reaction time showed that a shorter reaction time with 16 h is viable for the reaction and even showed an improved isolated yield.

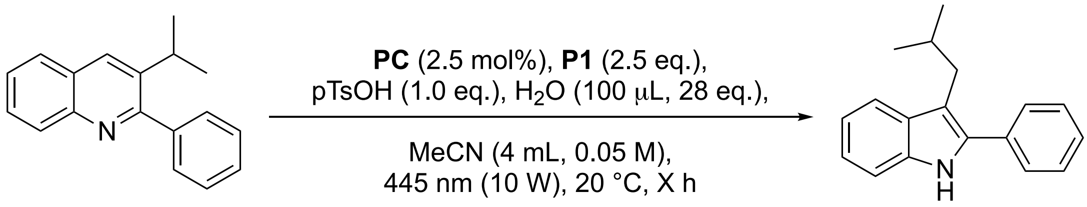

1a, 0.2 mmol

2a

Entry	Reaction time	**2a** Yield[a] / %	Conversion %
1	Standard conditions 24 h	>99 (86)	Full conversion
2	18 h	>99	Full conversion
3	16 h	>99 (92)	Full conversion
4	6 h	89	93

[a] Determined by ^{1}H-NMR with CH_2Br_2 as internal standard @ 0.2 mmol, isolated yield in parentheses.

Further PCs were analyzed under our reaction conditions to see whether the iridium-based **PC** can be replaced and a non-transition metal **PC** can be employed. The used organic dyes were submitted to the reaction with a higher catalyst loading of 5.0 mol%, but still no sufficient reactivity with a same yield as **PC** was observed.

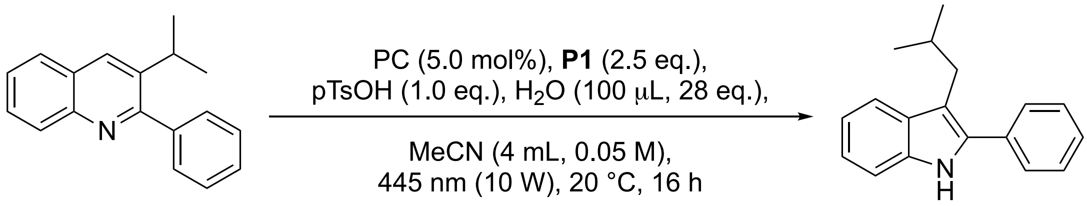

1a, 0.2 mmol **2a**

Entry	Photocatalyst PC	**2a** Yield[a] / %	Conversion %
1[b]	Standard conditions **PC** (2.5 mol%)	>99 (92)	Full conversion
2	Rhodamine 6G	19	21
3	Methylene blue	–	No conversion
4	Eosin Y	30	34
5	[Mes-Acr-Ph][BF_4]	–	No conversion
6	4CzIPN	34	39

[a] Determined by ^{1}H-NMR with CH_2Br_2 as internal standard @ 0.2 mmol, isolated yield in parentheses.
[b] The marked Ir-PC was used with 2.5 mol%, whereas the organic dyes were used with 5 mol%.

The controls of the different reagent proved to be ok and it can be stated that the reaction only proceeds with every part of the reaction in place. It was also observed that the product is not completely stable in the chosen reaction conditions, proven by the decomposition of indole, when submitting the product to the reaction conditions for 24 h.

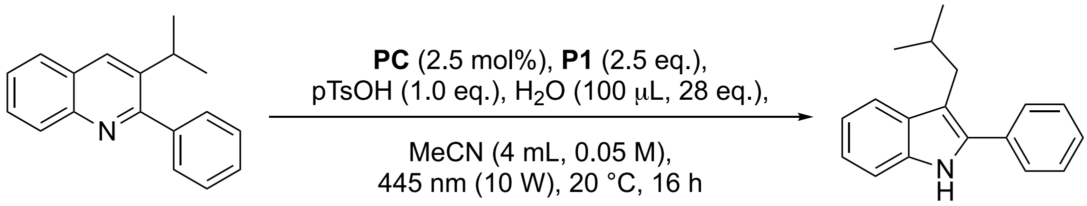

1a, 0.2 mmol **2a**

Entry	Controls	**2a** Yield[a] / %	Conversion %
1	Standard conditions	>99 (92)	Full conversion
2	No light	–	No conversion
3	No Photocatalysator	–	No conversion
4	No Phosphine	–	No conversion
5	No H_2O[b]	36	43
5a	+ molsieves in reaction	28	91
6	No Acid	–	21
7[c]	As HCl-salt	56	Full conversion
8[c]	As HCl-salt without H_2O	24	Full conversion
9	Submit indole **2a** to reaction for 24 h	94	–

[a] Determined by ^{1}H-NMR with CH_2Br_2 as internal standard @ 0.2 mmol, isolated yield in parentheses.

[b] pTsOH dried at 100 °C under high vacuum.

[c] Reactions performed without pTsOH.

4. Synthesis of Products

4.1. Overview of Synthesized Indoles

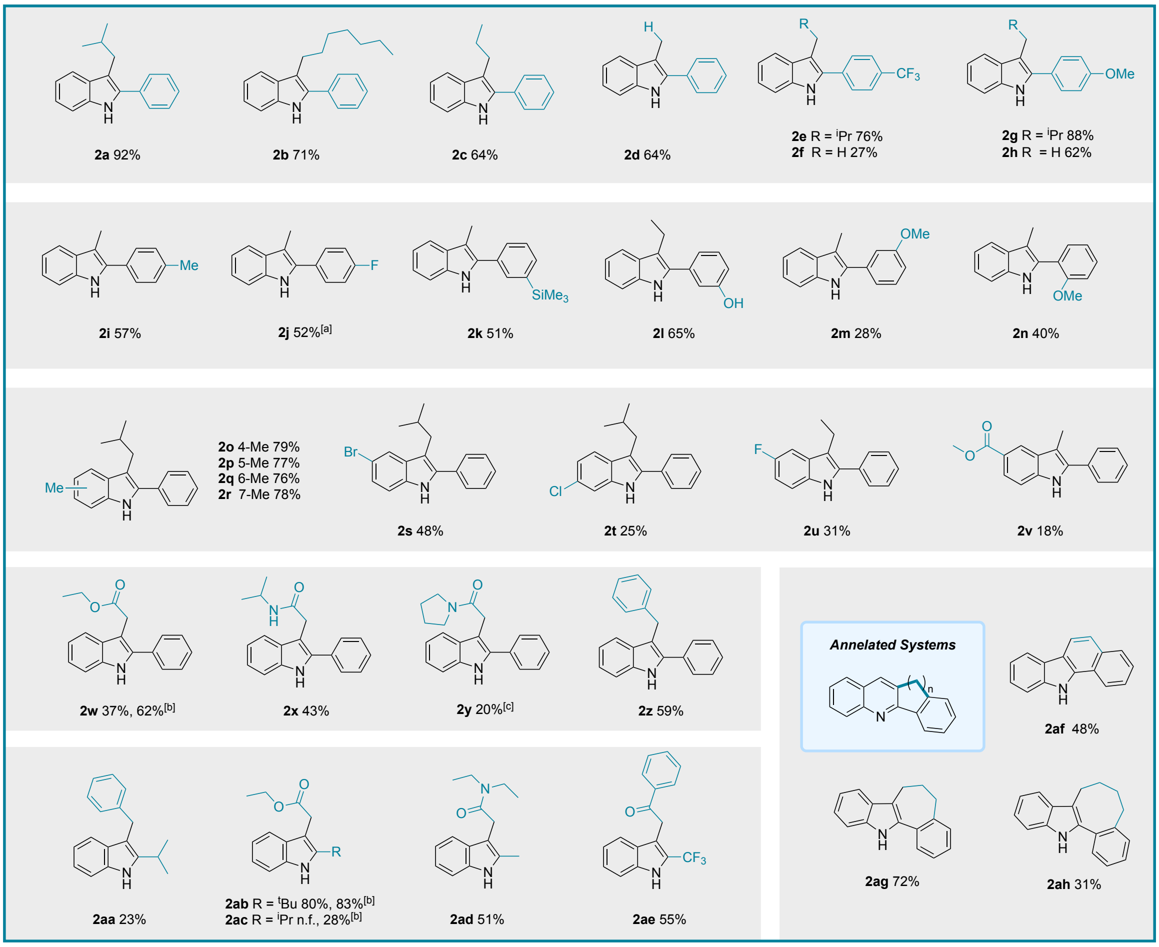

4.2. General Procedure for Product Synthesis

General Procedure 8 (GP8) – shown with the standard substrate **1a** as example

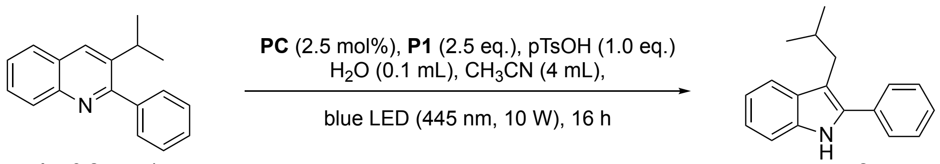

1a, 0.2 mmol **2a**

Quinoline (0.2 mmol, 1.0 eq.), **PC** (5.6 mg; 5.0 µmol; 2.5 mol%), **P1** (176 mg, 0.500 mmol, 2.5 eq.) and dried pTsOH (34.4 mg; 0.100 mmol, 1.0 eq.) were added into the reaction vessel. The mixture was put under argon atmosphere. Dry MeCN (4.0 ml; 0.05 mol/L) and water (100 µL; 28 eq.) were added and the mixture was purged with argon for 5 min. The reaction mixture was stirred for 16 h at 20 °C and irradiated with blue light (LED; 445 nm, 10 W). The reaction was washed into a round bottom flask with DCM and quenched by addition of sat. aq. Na_2CO_3 solution. The aqueous layer was extracted with DCM, the combined organic layers were dried over $MgSO_4$ and filtrated. The solvents were removed at the rotary evaporator under reduced pressure.

The crude product was checked via TLC for the retention of the product and the phosphine before quenching. If the phosphine runs near the product, H_2O_2 was added to oxidize phosphine to the phosphine oxide for an easier isolation. The products were isolated via column chromatography using SiO_2 and the declared solvent in the respective entry. Deviations from **GP8** are declared in the respective entries.

4.3. Synthesis and Characterization of Products

3-isobutyl-2-phenyl-1*H*-indole (2a):

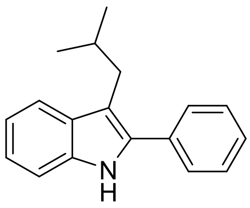

The reaction was performed according to **GP8** with 2-phenyl-3-propan-2-ylquinoline **1a** (49.5 mg, 0.200 mmol, 1.0 eq.). After purification via Flash-Chromatography (P/EtOAc – 30/1), the product **2a** was obtained as a white solid (45.8 mg, 0.184 mmol, 92%).

^{1}H-NMR (599 MHz, $CDCl_3$): δ (ppm) = 7.99 (s, 1H), 7.66 – 7.64 (m, 1H), 7.59 – 7.57 (m, 2H). 7.49 – 7.46 (m, 2H), 7.38 – 7.36 (m, 2H), 7.21 (ddd, *J* = 8.1, 7.1, 1.2 Hz, 1H), 7.14 (ddd, *J* = 8.0, 7.0, 1.0 Hz, 1H), 2.79 (d, *J* = 7.3 Hz, 2H), 2.07 (hept, *J* = 6.7 Hz, 1H), 0.91 (d, *J* = 6.6 Hz, 6H).
^{13}C-NMR (151 MHz, $CDCl_3$): δ (ppm) = 136.0, 134.9, 133.9, 129.8, 128.9, 128.3, 127.6, 122.2, 119.8, 119.5, 113.4, 110.8, 33.8, 30.0, 23.0.
HRMS (ESI): m/z calculated for $[M-H]^-$ $C_{18}H_{18}N^-$ 248.1445, found 248.1442.
IR (Film): $\tilde{\nu}$ (cm^{-1}) = 3396, 3378, 2051, 2952, 2925, 2865, 1884, 1602, 1484, 1457, 1446, 1425, 1329, 1244, 1221, 1011, 740, 697, 555, 501.
Melting point: *T* (°C) = 59-60.

3-heptyl-2-phenyl-1*H*-indole (2b):

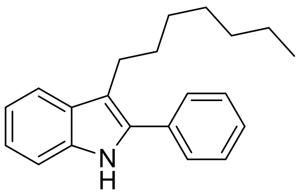

The reaction was performed according to **GP8** with 3-hexyl-2-phenylquinoline **1b** (57.9 mg, 0.200 mmol, 1.0 eq.). After purification via Flash-Chromatography (P/EtOAc – 25/1 to 20/1), the product **2b** was obtained as a colorless oil (41.2 mg, 0.141 mmol, 71%).
^{1}H-NMR (300 MHz, $CDCl_3$): δ (ppm) = 7.97 (s, 1H), 7.68 (d, *J* = 7.7Hz, 1H), 7.61 – 7.54 (m, 2H), 7.54 – 7.45 (m, 2H), 7.42 – 7.35 (m, 2H), 7.23 (ddd, *J* = 8.0, 7.0, 1.4 Hz, 1H), 7.16 (ddd, *J* = 8.2, 7.1, 1.3 Hz, 1H), 2.94 – 2.86 (m, 2H), 1.82 – 1.69 (m, 2H), 1.47 – 1.21 (m, 8H), 0.90 (t, *J* = 6.7 Hz, 3H).
^{13}C-NMR (101 MHz, $CDCl_3$): δ (ppm) = 136.1, 134.1, 133.7, 129.5, 128.9, 128.1, 127.6, 122.3, 119.6, 119.5, 114.3, 110.9, 32.0, 31.2, 30.0, 29.3, 24.7, 22.8, 14.3.
HRMS (ESI): m/z calculated for $[M-H]^-$ $C_{21}H_{24}N^-$ 290.1914, found 290.1913.
IR (Film): $\tilde{\nu}$ (cm^{-1}) = 3405, 3320, 3058, 2953, 2925, 2855, 2360, 2338, 1539, 1490, 1457, 1448, 1340, 1306, 1262, 1074, 755, 741, 696.

2-phenyl-3-propyl-1*H*-indole (2c):

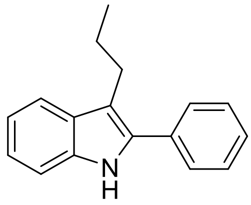

The reaction was performed according to **GP8** with 3-ethyl-2-phenylquinoline **1c** (46.7 mg, 0.200 mmol, 1.0 eq.). After purification via Flash-Chromatography (P/EtOAc – 25/1 to 20/1), the product **2c** was obtained as a white solid (30.3 mg, 0.129 mmol, 64%).

^{1}H-NMR (400 MHz, $CDCl_3$): δ (ppm) = 7.99 (s, 1H), 7.64 (d, *J* = 7.9 Hz, 1H), 7.59 – 7.54 (m, 2H), 7.50 – 7.45 (m, 2H), 7.37 (m, 2H), 7.20 (ddd, *J* = 8.1, 7.1, 1.2 Hz, 1H), 7.14 (ddd, *J* = 8.1, 7.0, 1.1 Hz, 1H), 2.90 – 2.83 (m, 2H), 1.76 (dq, *J* = 14.9, 7.5 Hz, 2H), 1.00 (t, *J* = 7.3 Hz, 3H).
^{13}C-NMR (101 MHz, $CDCl_3$): δ (ppm) = 136.0, 134.3, 133.6, 129.5, 128.9, 128.1, 127.6, 122.3, 119.6, 119.5, 114.1, 110.9, 26.9, 24.4, 14.6.
HRMS (ESI): m/z calculated for $[M-H]^-$ $C_{17}H_{16}N^-$ 234.1288, found 234.1287.
The analytical data match those reported in the literature.[27]

3-methyl-2-phenyl-1*H*-indole (2d):

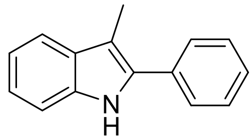

The reaction was performed according to **GP8** with 2-phenylquinoline **1d** (41.1 mg, 0.200 mmol, 1.0 eq.). After purification via Flash-Chromatography (P/EtOAc – 30/1 to 25/1), the product **2d** was obtained as a white solid (26.4 mg, 0.127 mmol, 64%).
^{1}H-NMR (400 MHz, $CDCl_3$): δ (ppm) = 8.01 (s, 1H), 7.65 – 7.57 (m, 3H), 7.49 (m, 2H), 7.41 – 7.34 (m, 2H), 7.22 (ddd, *J* = 8.1, 7.6, 1.4 Hz, 1H), 7.16 (ddd, *J* = 8.1, 7.1, 1.2 Hz, 1H), 2.48 (s, 3H).
^{13}C-NMR (101 MHz, $CDCl_3$): δ (ppm) = 135.9, 134.1, 133.5, 130.2, 129.0, 127.9, 127.5, 122.5, 119.7, 119.1, 110.8, 108.9, 9.8.
HRMS (ESI): m/z calculated for $[M-H]^-$ $C_{15}H_{12}N^-$ 206.0975, found 206.0974.
The analytical data match those reported in the literature.[27]

3-isobutyl-2-(4-(trifluoromethyl)phenyl)-1*H*-indole (2e):

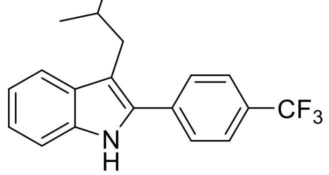

The reaction was performed according to **GP8** with 2-(4-(trifluoromethyl)phenyl)-3-propan-2-ylquinoline **1e** (63.1 mg, 0.200 mmol, 1.0 eq.). After purification via Flash-Chromatography (P/EtOAc – 20/1), the product **2e** was obtained as a white solid (48.3 mg, 0.152 mmol, 76%).
^{1}H-NMR (500 MHz, $CDCl_3$): δ (ppm) = 8.01 (s, 1H), 7.74 – 7.68 (m, 4H), 7.66 (dd, *J* = 8.0, 1.0 Hz, 1H), 7.39 (dt, *J* = 8.1, 0.9 Hz, 1H), 7.24 (ddd, *J* = 8.2, 7.1, 1.2 Hz, 1H), 7.15 (ddd, *J* = 8.0, 7.0, 1.0 Hz, 1H), 2.80 (d, *J* = 7.3 Hz, 2H), 2.05 (hept, *J* = 6.8 Hz, 1H), 0.91 (d, *J* = 6.6 Hz, 6H).

^{13}C-NMR{^{19}F} (126 MHz, CDCl$_3$): δ(ppm) = 137.4, 136.3, 133.1, 129.7, 129.4, 128.3, 125.9, 124.3, 123.0, 120.1, 119.9, 114.9, 111.0, 33.8, 30.1, 23.0.

^{19}F{^{1}H} NMR (470 MHz, CDCl$_3$) δ(ppm) = -62.5.

HRMS (ESI): m/z calculated for $[M-H]^-$ $C_{19}H_{17}F_3N^-$ 316.1319, found 316.1316.

IR (Solid): $\tilde{\nu}$ (cm^{-1}) = 3462, 3376, 3054, 2962, 2953, 2925, 2905, 2868, 2845, 1615, 1456, 1436, 1321, 1165, 1123, 1106, 1068, 1013, 846, 822, 739, 690, 605, 583, 555, 502.

Melting point: T (°C) = 56-60.

3-methyl-2-(4-(trifluoromethyl)phenyl)-1*H*-indole (2f):

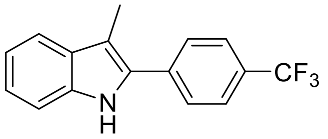

The reaction was performed according to **GP8** with 2-(4-(trifluoromethyl)phenyl)quinoline **1f** (54.7 mg, 0.200 mmol, 1.0 eq.). After purification via Flash-Chromatography (P/EtOAc – 25/1 to 20/1), the product **2f** was obtained as a white solid (15.0 mg, 54.5 µmol, 27%).

^{1}H-NMR (400 MHz, DMSO-d_6): δ(ppm) = 11.33 (s, 1H), 7.93 – 7.82 (m, 4H), 7.58 (d, J = 7.9 Hz, 1H), 7.39 (dt, J = 8.2, 0.9 Hz, 1H), 7.15 (ddd, J = 8.1, 7.0, 1.2 Hz, 1H), 7.04 (ddd, J = 8.0, 7.0, 1.0 Hz, 1H), 2.46 (s, 3H).

^{13}C-NMR (101 MHz, CDCl$_3$): δ(ppm) = 137.0, 136.2, 132.0, 129.2, 127.8, 126.9 (q, J = 32.2 Hz), 125.6(q, J = 3.8 Hz)., 124.4 (q, J = 270.0 Hz), 122.3, 118.9, 118.8, 111.2, 108.8, 9.9.

^{19}F{^{1}H}-NMR (376 MHz, CDCl$_3$): δ(ppm) = -60.81.

HRMS (ESI): m/z calculated for $[M-H]^-$ $C_{16}H_{11}F_3N^-$ 274.0849, found 274.0847.

The analytical data match those reported in the literature.[28]

3-(2-methylpropyl)-2-(4-methoxyphenyl)-1*H*-indole (2g):

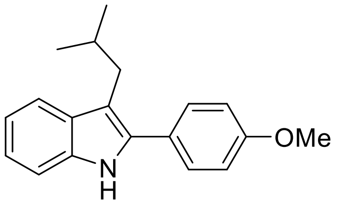

The reaction was performed according to **GP8** with 2-(4-methoxyphenyl)-3-propan-2-ylquinoline **1g** (55.5 mg, 0.200 mmol, 1.0 eq.). After purification via Flash-Chromatography (P/EtOAc – 25/1 to 15/1), the product **2g** was obtained as a white resin (49.1 mg, 0.176 mmol, 88%).

^{1}H-NMR (599 MHz, CDCl$_3$): δ(ppm) = 7.94 (s, 1H), 7.65 – 7.61 (m, 1H), 7.53 – 7.47 (m, 2H), 7.35 (dt, J = 8.0, 0.9 Hz, 1H), 7.19 (ddd, J = 8.1, 7.0, 1.2 Hz, 1H), 7.13 (ddd, J = 8.0, 7.1, 1.1 Hz, 1H), 7.04 – 6.97 (m, 2H) 3.88 (s, 3H), 2.76 (d, J = 7.3 Hz, 2H), 2.06 (hept, J = 6.8 Hz, 1H), 0.91 (d, J = 6.6 Hz, 1H).

^{13}C-NMR (151 MHz, CDCl$_3$): δ(ppm) = 159.2, 135.8, 134.8, 129.8, 129.6, 126.4, 121.9, 119.6, 119.4, 114.3, 112.6, 110.7, 55.5, 33.8, 30.0, 23.0.

HRMS (ESI): m/z calculated for $[M+H]^+$ $C_{19}H_{22}NO^+$ 280.1696, found 280.1696.

IR (Film): $\tilde{\nu}$ (cm^{-1}) = 3403, 2952, 2933, 2867, 2837, 1604, 1507, 1458, 1439, 1305, 1282, 1249, 1176, 1029, 835, 763, 741, 548.

2-(4-methoxyphenyl)-3-methyl-1*H*-indole (2h):

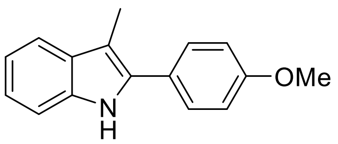

The reaction was performed according to **GP8** with 2-(4-methoxyphenyl)quinoline **1h** (47.1 mg, 0.200 mmol, 1.0 eq.). After purification via Flash-Chromatography (P/EtOAc – 25/1 to 10/1), the product **2h** was obtained as a white solid (29.4 mg, 0.124 mmol, 62%).

^{1}H-NMR (400 MHz, $CDCl_3$): δ (ppm) = 7.95 (s, 1H), 7.60 (d, *J* = 7.7 Hz, 1H), 7.55 – 7.42 (m, 2H), 7.35 (d, *J* = 7.9 Hz, 1H), 7.23 – 7.11 (m, 2H), 7.07 – 6.99 (m, 2H), 3.88 (s, 3H), 2.45 (s, 3H).

^{13}C-NMR (101 MHz, $CDCl_3$): δ (ppm) = 159.1, 135.8, 134.1, 130.2, 129.1, 126.0, 122.1, 119.6, 118.9, 114.4, 110.7, 107.9, 55.5, 9.7.

HRMS (ESI): m/z calculated for $[M-H]^-$ $C_{16}H_{14}NO^-$ 236.1081, found 236.1079.

The analytical data match those reported in the literature.[29]

3-methyl-2-(4-methylphenyl)-1*H*-indole (2i):

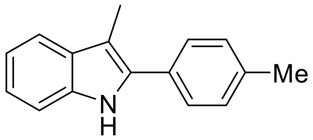

The reaction was performed according to **GP8** with 2-(4-methylphenyl)quinoline **1i** (43.9 mg, 0.200 mmol, 1.0 eq.). After purification via Flash-Chromatography (P/EtOAc – 25/1), the product **2i** was obtained as a white solid (25.1 mg, 0.113 mmol, 57%).

^{1}H-NMR (400 MHz, $CDCl_3$): δ (ppm) = 7.98 (s, 1H), 7.62 (d, *J* = 7.8 Hz, 1H), 7.54 – 7.45 (m, 2H), 7.37 (d, *J* = 7.8 Hz, 1H), 7.31 (d, *J* = 7.8 Hz, 2H), 7.25 – 7.13 (m, 2H), 2.47 (s, 3H), 2.44 (s, 3H).

^{13}C-NMR (101 MHz, $CDCl_3$): δ (ppm) = 137.3, 135.8, 134.2, 130.6, 130.2, 129.7, 127.7, 122.2, 119.6, 119.0, 110.7, 108.4, 21.4, 9.8.

HRMS (ESI): m/z calculated for $[M-H]^-$ $C_{16}H_{14}N^-$ 220.1132, found 220.1131.

The analytical data match those reported in the literature.[30]

2-(4-fluorophenyl)-3-methyl-1*H*-indole (2j):

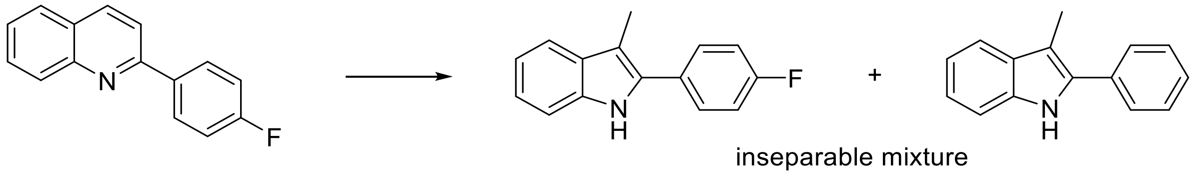

The reaction was performed according to **GP8** with 2-(4-fluorophenyl)quinoline **1j** (44.7 mg, 0.200 mmol, 1.0 eq.). After purification via Flash-Chromatography (P/EtOAc – 30/1), the product **2j** and defluorinated side product were obtained as a white solid (26.5 mg, 0.118 mmol,

59% combined, 52% **2j**). The mixture of both was inseparable. The NMR-analytics are given below for the major product **2j** still inheriting the Fluor-atom. The product-ratio was determined via ^{1}H-NMR and both products could be found in the HRMS-analysis.

^{1}H-NMR (400 MHz, $CDCl_3$): δ (ppm) = 7.94 (s, 1H), 7.66 – 7.57 (m, 1H), 7.57 – 7.47 (m, 2H), 7.41 – 7.33 (m, 1H), 7.26 – 7.13 (m, 4H), 2.44 (s, 3H).

^{13}C-NMR (101 MHz, $CDCl_3$): δ (ppm) = 162.3 (d, *J* = 247.4 Hz), 135.9, 133.3, 130.0, 129.6 (d, *J* = 8.0 Hz), 122.5, 119.8, 119.1, 116.0 (d, *J* = 21.5 Hz), 110.8, 108.8, 9.7.

^{19}F{^{1}H}-NMR (376 MHz, $CDCl_3$): δ (ppm) = -114.3.

HRMS (ESI): m/z calculated for $[M-H]^-$ $C_{15}H_{11}FN^-$ 224.0881, found 224.0880.
m/z calculated for $[M-H]^-$ $C_{15}H_{12}N^-$ 206.0975, found 206.0974.

The analytical data match those reported in the literature.[31]

3-methyl-2-(3-(trimethylsilyl)phenyl)-1*H*-indole (2k):

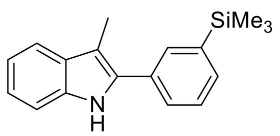

The reaction was performed according to **GP8** with 2-(3-(trimethylsilyl)phenyl)quinoline **1k** (55.5 mg, 0.200 mmol, 1.0 eq.). After purification via Flash-Chromatography (P/EtOAc – 50/1 to 30/1), the product **2k** was obtained as a white solid (28.6 mg, 0.102 mmol, 51%).

^{1}H-NMR (599 MHz, $CDCl_3$): δ (ppm) = 8.03 (s, 1H), 7.75 (s, 1H), 7.63 (d, *J* = 7.8 Hz, 1H), 7.58 (dt, *J* = 7.5, 1.7 Hz, 1H), 7.54 (dt, *J* = 7.5, 1.3 Hz, 1H), 7.49 (t, *J* = 7.4 Hz, 1H), 7.40 (dt, *J* = 8.1, 1.0 Hz, 1H), 7.23 (ddd, *J* = 8.2, 7.1, 1.3 Hz, 1H), 7.17 (ddd, *J* = 8.0, 7.0, 1.1 Hz, 1H), 2.49 (s, 3H), 0.35 (s, 9H).

^{13}C-NMR (151 MHz, $CDCl_3$): δ (ppm) = 141.4, 136.0, 134.5, 132.8, 132.8, 132.4, 130.2, 128.4, 128.3, 122.4, 119.7, 119.1, 110.8, 108.8, 9.8, -1.0.

^{29}Si-NMR (119 MHz, $CDCl_3$): δ (ppm) = -3.6.

HRMS (ESI): m/z calculated for $[M-H]^-$ $C_{18}H_{20}NSi^-$ 278.1371, found 278.1369.

IR (Solid): $\tilde{\nu}$ (cm^{-1}) = 3422, 3036, 2952, 1593, 1460, 1445, 1394, 1360, 1335, 1304, 1246, 1121, 855, 833, 819, 794, 742, 703, 691, 680, 661, 619, 579, 460.

Melting point: *T* (°C) = 71-72.

3-(3-ethyl-1*H*-indol-2-yl)phenol (2l):

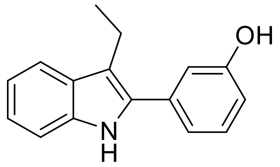

The reaction was performed according to **GP8** with 3-(3-methylquinolin-2-yl)phenol **1l** (47.1 mg, 0.200 mmol, 1.0 eq.). After purification via Flash-Chromatography (P/EtOAc – 12/1 to 5/1), the product **2l** was obtained as a white oil (30.9 mg, 0.119 mmol, 65%).

^{1}H-NMR (300 MHz, $CDCl_3$): δ (ppm) = 7.94 (s, 1H), 7.66 (d, *J* = 7.7 Hz, 1H), 7.34 (td, *J* = 8.6, 1.8 Hz, 2H), 7.25 – 7.10 (m, 3H), 7.00 (t, *J* = 2.1 Hz, 1H), 6.83 (dd, *J* = 8.2, 2.5 Hz, 1H), 5.31 (s, 1H), 2.92 (q, *J* = 7.6 Hz, 2H), 1.34 (t, *J* = 7.5 Hz, 3H).

13C-NMR (101 MHz, $CDCl_3$): δ (ppm) = 156.0, 136.0, 135.1, 133.3, 130.2, 129.1, 122.5, 120.5, 119.6, 119.4, 115.8, 114.8, 114.6, 111.0, 17.9, 15.7.

HRMS (ESI): m/z calculated for $[M-H]^-$ $C_{16}H_{14}NO^-$ 236.1081, found 236.1080.

IR (Film): $\tilde{\nu}$ (cm^{-1}) = 3401, 3054, 2962, 2926, 2872, 1611, 1586, 1485, 1454, 1338, 1309, 1262, 1199, 1155, 1083, 1011, 916, 870, 785, 738, 685, 467.

2-(3-methoxyphenyl)-3-methyl-1*H*-indole (2m):

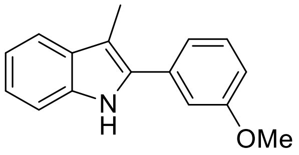

The reaction was performed according to **GP8** with 2-(3-methoxyphenyl)quinoline **1m** (47.1 mg, 0.200 mmol, 1.0 eq.). After purification via Flash-Chromatography (P/EtOAc – 20/1 to 15/1), the product **2m** was obtained as a white resin (13.2 mg, 55.6μmol, 28%).

1H-NMR (400 MHz, $CDCl_3$): δ (ppm) = 8.01 (s, 1H), 7.63 – 7.58 (m, 1H), 7.44 – 7.32 (m, 2H), 7.25 – 7.11 (m, 4H), 6.91 (ddd, *J* = 8.3, 2.6, 1.0 Hz, 1H), 3.88 (s, 3H), 2.48 (s, 3H).

13C-NMR (101 MHz, $CDCl_3$): δ (ppm) = 160.0, 135.9, 134.8, 134.0, 130.1, 130.0, 122.5, 120.3, 119.7, 119.1, 113.6, 112.9, 110.8, 109.0, 55.5, 9.8.

HRMS (ESI): m/z calculated for $[M-H]^-$ $C_{16}H_{14}NO^-$ 236.1081, found 236.1079.

IR (Film): $\tilde{\nu}$ (cm^{-1}) = 3313, 3049, 2957, 2925, 2857, 1650, 1599, 1580, 1535, 1487, 1450, 1314, 1269, 1224, 1180, 1092, 1043, 875, 754, 736, 685, 609, 581, 479.

2-(2-methoxyphenyl)-3-methyl-1*H*-indole (2n):

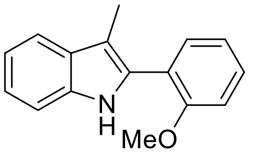

The reaction was performed according to **GP8** with 2-(2-methoxyphenyl)quinoline **1n** (47.1 mg, 0.200 mmol, 1.0 eq.). After purification via Flash-Chromatography (P/EtOAc – 20/1 to 15/1), the product **2n** was obtained as a white solid (19.0 mg, 80.1 μmol, 40%).

1H-NMR (400 MHz, $CDCl_3$): δ (ppm) = 8.69 (s, 1H), 7.65 – 7.58 (m, 1H), 7.55 (dd, *J* = 7.6, 1.8 Hz, 2H), 7.40 – 7.32 (m, 2H), 7.22 – 7.17 (m, 1H), 7.16 – 7.03 (m, 3H), 3.90 (s, 3H), 2.44 (s, 3H).

13C-NMR (101 MHz, $CDCl_3$): δ (ppm) = 156.8, 135.6, 131.4, 131.3, 129.3, 128.9, 122.1, 121.8, 121.1, 119.1, 118.9, 111.6, 110.7, 109.5, 55.9, 10.3.

HRMS (ESI): m/z calculated for $[M-H]^-$ $C_{16}H_{14}NO^-$ 236.1081, found 236.1079.[5]

The analytical data match those reported in the literature.[31]

3-isobutyl-4-methyl-2-phenyl-1*H*-indole (2o):

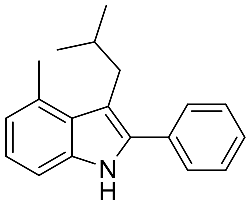

The reaction was performed according to **GP8** with 3-isopropyl-5-methyl-2-phenylquinoline **1o** (52.3 mg, 0.200 mmol, 1.0 eq.). After purification via Flash-Chromatography (P/DCM – 10/1 to 1/1), the product **2o** was obtained as a white resin (41.7 mg, 0.158 mmol, 79%).

^{1}H-NMR (400 MHz, $CDCl_3$): δ (ppm) = 7.95 (s, 1H), 7.56 – 7.52 (m, 2H), 7.49 – 7.43 (m, 2H), 7.40 – 7.34 (m, 1H), 7.21 (d, *J* = 8.1 Hz, 1H), 7.08 (dd, *J* = 8.1, 7.1 Hz, 1H), 6.88 (d, *J* = 7.1 Hz, 1H), 2.89 (d, *J* = 7.0 Hz, 2H), 2.74 (s, 3H), 1.84 (hept, *J* = 6.7 Hz, 1H), 0.75 (d, *J* = 6.6 Hz, 6H).
^{13}C-NMR (101 MHz, $CDCl_3$): δ (ppm) = 136.4, 135.6, 134.4, 131.2, 129.2, 128.8, 127.6, 127.6, 122.1, 121.8, 113.9, 108.8, 34.5, 31.7, 22.4, 20.5.
HRMS (ESI): m/z calculated for $[M-H]^-$ $C_{19}H_{20}N^-$ 262.1601, found 262.1599.
IR (Film): $\tilde{\nu}$ (cm^{-1}) = 3363, 3054, 2956, 2925, 2867, 1604, 1489, 1464, 1446, 1379, 1362, 1329, 1303, 1236, 1166, 1074, 1021, 771, 747, 700, 582, 539, 521.

3-isobutyl-5-methyl-2-phenyl-1*H*-indole (2p):

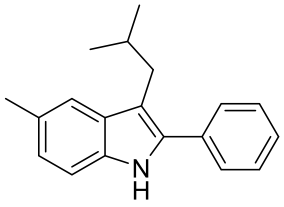

The reaction was performed according to **GP8** with 3-isopropyl-6-methyl-2-phenylquinoline **1p** (52.3 mg, 0.200 mmol, 1.0 eq.). After purification via Flash-Chromatography (P/EtOAc – 30/1), the product **2p** was obtained as a yellow solid (40.5 mg, 0.155 mmol, 77%).

^{1}H-NMR (400 MHz, $CDCl_3$): δ (ppm) = 7.89 (s, 1H), 7.61 – 7.54 (m, 2H), 7.49 – 7.44 (m, 2H), 7.43 – 7.41 (m, 1H), 7.38 – 7.33 (m, 1H), 7.26 (d, *J* = 8.2 Hz, 1H), 7.03 (dd, *J* = 8.3, 1.6 Hz, 1H), 2.77 (d, *J* = 7.3 Hz, 2H), 2.49 (s, 3H), 2.07 (hept, *J* = 6.9 Hz, 1H), 0.91 (d, *J* = 6.6 Hz, 6H).
^{13}C-NMR (101 MHz, $CDCl_3$): δ (ppm) = 135.0, 134.4, 134.1, 130.0, 128.8, 128.7, 128.3, 127.5, 123.8, 119.5, 112.9, 110.5, 33.8, 29.9, 23.0, 21.8.
HRMS (ESI): m/z calculated for $[M-H]^-$ $C_{19}H_{20}N^-$ 262.1601, found 262.1600.
IR (Solid): $\tilde{\nu}$ (cm^{-1}) = 3398, 3373, 3054, 2945, 2864, 1602, 1446, 1381, 1363, 1308, 1258, 1220, 1167, 1074, 1022, 870, 800, 766, 723, 698, 607, 589, 570, 526, 507, 487.
Melting point: *T* (°C) = 116-117.

3-isobutyl-6-methyl-2-phenyl-1*H*-indole (2q):

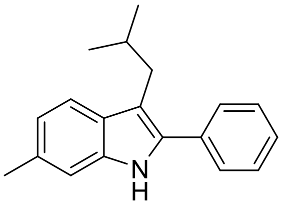

The reaction was performed according to **GP8** with 3-isopropyl-7-methyl-2-phenylquinoline **1q** (52.3 mg, 0.200 mmol, 1.0 eq.). After purification via Flash-Chromatography (P/DCM – 20/1 to 10/1), the product **2q** was obtained as a white resin (40.2 mg, 0.153 mmol, 76%).

^{1}H-NMR (400 MHz, $CDCl_3$): δ (ppm) = 7.87 (s, 1H), 7.62 – 7.55 (m, 2H), 7.54 (d, *J* = 8.1 Hz, 1H), 7.50 – 7.42 (m, 2H), 7.40 – 7.32 (m, 1H), 7.19 – 7.14 (m, 1H), 6.98 (dd, *J* = 8.1, 1.5 Hz, 1H), 2.78 (d, *J* = 7.3 Hz, 2H), 2.49 (s, 3H), 2.07 (hept, *J* = 6.8 Hz, 1H), 0.92 (d, *J* = 6.4 Hz, 6H).
^{13}C-NMR (101 MHz, $CDCl_3$): δ (ppm) = 136.4, 134.2, 134.1, 132.1, 128.9, 128.2, 127.7, 127.4, 121.3, 119.5, 113.2, 110.8, 33.9, 30.0, 23.0, 21.9.

HRMS (ESI): m/z calculated for [M-H]$^-$ $C_{19}H_{20}N^-$ 262.1601, found 262.1599.

IR (Film): $\tilde{\nu}$ (cm^{-1}) = 3206, 2950, 2864, 1620, 1575, 1530, 1445, 1364, 1261, 1207, 1038, 938, 885, 817, 780, 755, 692, 599, 535.

3-isobutyl-7-methyl-2-phenyl-1*H*-indole (2r):

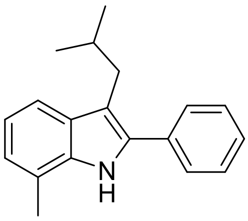

The reaction was performed according to **GP8** with 3-isopropyl-8-methyl-2-phenylquinoline **1r** (52.3 mg, 0.200 mmol, 1.0 eq.). After purification via Flash-Chromatography (P/DCM – 10/1), the product **2r** was obtained as a white resin (40.9 mg, 0.155 mmol, 78%).

^{1}H-NMR (400 MHz, $CDCl_3$): δ (ppm) = 7.90 (s, 1H), 7.66 – 7.60 (m, 2H), 7.55 – 7.46 (m, 3H), 7.42 – 7.36 (m, 1H), 7.09 (t, *J* = 7.5 Hz, 1H), 7.03 (d, *J* = 7.0 Hz, 1H), 2.80 (d, *J* = 7.3 Hz, 2H), 2.54 (s, 3H), 2.09 (hept, *J* = 6.7 Hz, 1H), 0.93 (d, *J* = 6.6 Hz, 6H).

^{13}C-NMR (101 MHz, $CDCl_3$): δ (ppm) = 135.5, 134.7, 134.1, 129.3, 128.9, 128.4, 127.6, 122.8, 120.0, 119.8, 117.6, 113.9, 33.9, 30.0, 23.0, 16.8.

HRMS (ESI): m/z calculated for [M-H]$^-$ $C_{19}H_{20}N^-$ 262.1601, found 262.1600.

IR (Film): $\tilde{\nu}$ (cm^{-1}) = 3309, 3054, 2954, 2867, 1666, 1601, 1534, 1491, 1445, 1365, 1303, 1240, 1173, 1050, 958, 926, 886, 850, 765, 744, 711, 689, 649, 583, 510, 484.

5-bromo-3-isobutyl-2-phenyl-1*H*-indole (2s):

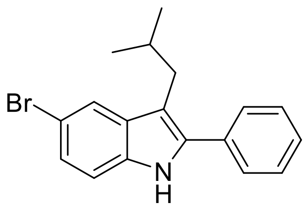

The reaction was performed according to **GP8** with 6-bromo-3-isopropyl-2-phenylquinoline **1s** (65.2 mg, 0.200 mmol, 1.0 eq.). After purification via Flash-Chromatography (P/EtOAc – 25/1 to 20/1), the product **2s** was obtained as a yellow solid (31.2 mg, 95.1 µmol, 48%).

^{1}H-NMR (599 MHz, $CDCl_3$): δ (ppm) = 8.01 (s, 1H), 7.74 (d, *J* = 1.9 Hz, 1H), 7.58 – 7.53 (m, 2H), 7.50 – 7.44 (m, 2H), 7.41 – 7.35 (m, 1H), 7.27 (dd, *J* = 8.5, 1.8 Hz, 1H), 7.23 (dd, *J* = 8.5, 0.6 Hz, 1H), 2.72 (d, *J* = 7.4 Hz, 2H), 2.02 (hept, *J* = 6.9 Hz, 1H), 0.89 (d, *J* = 6.6Hz, 6H).

^{13}C-NMR (151 MHz, $CDCl_3$): δ (ppm) = 136.2, 134.5, 133.3, 131.6, 129.0, 128.3, 128.0, 124.9, 122.3, 113.0, 112.8, 112.2, 33.6, 29.9, 22.9.

HRMS (ESI): m/z calculated for [M-H]$^-$ $C_{18}H_{17}BrN^-$ 326.0550, found 326.0547.

IR (Solid): $\tilde{\nu}$ (cm^{-1}) = 3403, 3382, 3054, 2948, 2925, 2866, 1602, 1448, 1421, 1364, 1316, 1287, 1214, 1074, 1058, 1021, 921, 874, 796, 777, 766, 756, 699, 678, 644, 594, 569, 504, 486.

Melting point: *T* (°C) = 80-81.

6-chloro-3-isobutyl-2-phenyl-1*H*-indole (2t):

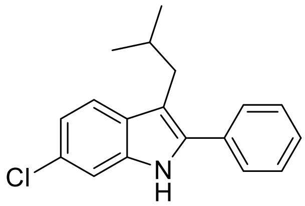

The reaction was performed according to **GP8** with 7-chloro-3-isopropyl-2-phenylquinoline **1t** (56.4 mg, 0.200 mmol, 1.0 eq.). After purification via Flash-Chromatography (P/DCM – 10/1), the product **2t** was obtained as a white resin (14.4 mg, 50.7 µmol, 25%).

^{1}H-NMR (400 MHz, $CDCl_3$): δ (ppm) = 7.99 (s, 1H), 7.59 – 7.51 (m, 3H), 7.50 – 7.44 (m, 2H), 7.43 – 7.33 (m, 2H), 7.09 (dd, *J* = 8.4, 1.9 Hz, 1H), 2.75 (d, *J* = 7.3 Hz, 2H), 2.01 (hept, *J* = 6.8 Hz, 1H), 0.88 (d, *J* = 6.6 Hz, 6H).

^{13}C-NMR (101 MHz, $CDCl_3$): δ (ppm) = 136.2, 135.5, 133.4, 129.0, 128.4, 128.2, 128.0, 127.9, 120.7, 120.2, 113.4, 110.7, 33.6, 30.0, 22.9.

HRMS (ESI): m/z calculated for $[M-H]^-$ $C_{18}H_{17}ClN^-$ 282.1055, found 282.1053.

IR (Film): $\tilde{\nu}$ (cm^{-1}) = 3421, 3315, 3056, 2953, 2925, 2867, 2360, 2342, 1604, 1458, 1446, 1335, 1224, 1064, 927, 800, 767, 698, 564.

3-ethyl-5-fluoro-2-phenyl-1*H*-indole (2u):

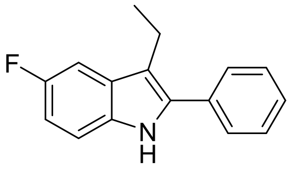

The reaction was performed according to **GP8** with 6-fluoro-3-methyl-2-phenylquinoline **1u** (47.5 mg, 0.200 mmol, 1.0 eq.). After purification via Flash-Chromatography (P/EtOAc – 20/1 to 10/1), the product **2u** was obtained as a white solid (14.7 mg, 61.4 µmol, 31%).

^{1}H-NMR (400 MHz, $CDCl_3$): δ (ppm) = 7.96 (s, 1H), 7.57 – 7.53 (m, 2H), 7.51 – 7.45 (m, 2H), 7.42 – 7.36 (m, 1H), 7.31 – 7.26 (m, 2H), 6.95 (td, *J* = 9.1, 2.4 Hz, 1H), 2.87 (q, *J* = 7.5 Hz, 2H), 1.33 (t, *J* = 7.6 Hz, 1H).

^{13}C-NMR (101 MHz, $CDCl_3$): δ (ppm) = 157.9 (d, *J* = 234.4 Hz), 135.8, 133.2, 132.6, 129.6 (d, *J* = 9.5 Hz), 129.0, 128.0, 127.9, 115.7 (d, *J* = 4.8 Hz), 111.5 (d, *J* = 9.6 Hz), 110.6 (d, *J* = 26.3 Hz), 104.2 (d, *J* = 23.3 Hz), 17.9, 15.6.

^{19}F{^{1}H}-NMR (376 MHz, $CDCl_3$) δ (ppm) = -124.5.

HRMS (ESI): m/z calculated for $[M-H]^-$ $C_{16}H_{13}FN^-$ 238.1038, found 238.1036.

IR (Solid): $\tilde{\nu}$ (cm^{-1}) = 3410, 3053, 2965, 2929, 2856, 1598, 1579, 1481, 1450, 1432, 1302, 1254, 1180, 1159, 1116, 1071, 953, 852, 794, 766, 745, 688, 607, 574, 504, 470.

Melting point: *T* (°C) = 75-76.

methyl 3-methyl-2-phenyl-1*H*-indole-5-carboxylate (2v):

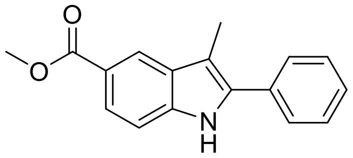

The reaction was performed according to **GP8** with methyl 2-phenylquinoline-6-carboxylate **1v** (52.7 mg, 0.200 mmol, 1.0 eq.). After purification via Flash-Chromatography (P/EtOAc –

25/1 to 10/1), the product **2v** was obtained as a white solid (9.8 mg, 37 µmol, 18%).

^{1}H-NMR (400 MHz, $CDCl_3$): δ (ppm) = 8.38 (m, 1H), 8.22 (s, 1H), 7.92 (dd, *J* = 8.6, 1.6 Hz, 1H), 7.62 – 7.55 (m, 2H), 7.54 – 7.45 (m, 2H), 7.43 – 7.29 (m, 2H), 3.95 (s, 3H), 2.50 (s, 3H).

^{13}C-NMR (101 MHz, $CDCl_3$): δ (ppm) = 168.4, 138.5, 135.4, 132.8, 129.9, 129.1, 127.9, 127.9, 123.9, 122.2, 121.7, 110.4, 110.1, 52.0, 9.7.

HRMS (ESI): m/z calculated for $[M+Na]^+$ $C_{17}H_{15}NO_2Na^+$ 288.0995, found 288.0995.

The analytical data match those reported in the literature.[26]

ethyl 2-(2-phenyl-1*H*-indol-3-yl)acetate (2w):

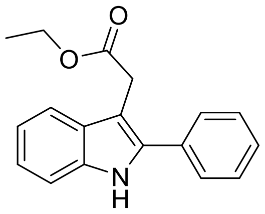

The reaction was performed according to **GP8** with ethyl 2-phenylquinoline-3-carboxylate **1w** (55.5 mg, 0.200 mmol, 1.0 eq.). After purification via Flash-Chromatography (P/EtOAc – 25/1 to 10/1), the product **2w** was obtained as a white solid (20.4 mg, 73.0 µmol, 37%). Adjusting the reaction conditions by employing **P3** (233 mg, 0.500 mmol, 2.5 eq.) as phosphine and a reduced amount of water (50 µL; 21 eq.) resulted after purification in a higher product formation (34.8 mg, 0.125 mmol, 62%).

^{1}H-NMR (300 MHz, $CDCl_3$): δ (ppm) = 8.17 (s, 1H), 7.74 – 7.62 (m, 3H), 7.53 – 7.45 (m, 2H), 7.44 – 7.35 (m, 2H), 7.26 – 7.11 (m, 2H), 4.18 (q, *J* = 7.2 Hz, 2H), 3.84 (s, 2H), 1.27 (t, *J* = 7.1 Hz, 3H).

^{13}C-NMR (76 MHz, $CDCl_3$): δ (ppm) = 172.4, 136.3, 135.9, 132.5, 129.2, 129.1, 128.4, 128.2, 122.7, 120.2, 119.5, 111.0, 105.9, 61.0, 31.3, 14.4.

HRMS (ESI): m/z calculated for $[M+Na]^+$ $C_{18}H_{17}NO_2Na^+$ 302.1151, found 302.1151.

The analytical data match those reported in the literature.[32]

***N*-isopropyl-2-(2-phenyl-1*H*-indol-3-yl)acetamide (2x):**

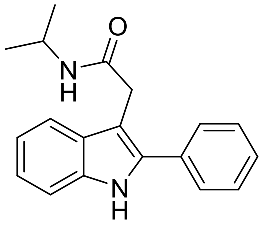

The reaction was performed according to **GP8** with *N*-isopropyl-2-phenylquinoline-3-carboxamide **1x** (58.1 mg, 0.200 mmol, 1.0 eq.). After purification via Flash-Chromatography (P/Et_2O – 1/1), the product **2x** was obtained as a white foam (25.4 mg, 87.9 µmol, 43%).

^{1}H-NMR (599 MHz, $CDCl_3$): δ (ppm) = 8.50 (s, 1H), 7.59 – 7.51 (m, 3H), 7.50 – 7.46 (m, 2H), 7.44 (m, 1H), 7.42 – 7.36 (m, 1H), 7.29 – 7.23 (m, 1H), 7.19 (m, 1H), 5.58 (d, *J* = 8.2 Hz, 1H), 4.15 – 4.04 (heptd, *J* = 8.2, 6.6 Hz, 1H), 3.82 (s, 2H), 0.97 (d, *J* = 6.6, 6H).

^{13}C-NMR (151 MHz, $CDCl_3$): δ (ppm) = 170.5, 136.6, 136.1, 132.2, 129.3, 129.1, 128.4, 127.8, 123.1, 120.6, 118.9, 111.2, 105.8, 41.5, 33.3, 22.7.

HRMS (ESI): m/z calculated for $[M-H]^-$ $C_{19}H_{19}N_2O^-$ 291.1503, found 291.1502.

IR (Solid): $\tilde{\nu}$ (cm^{-1}) = 3391, 3260, 3058, 2969, 2927, 1641, 1516, 1454, 1366, 1341, 1308, 1241, 1171, 768, 740, 697, 607, 560, 537, 493.
Melting point: T (°C) = 130-131.

2-(2-phenyl-1*H*-indol-3-yl)-1-(pyrrolidin-1-yl)ethan-1-one (2y):

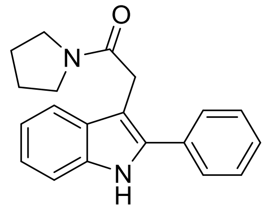

The reaction was performed according to **GP8** with (2-phenylquinolin-3-yl)(pyrrolidin-1-yl)methanone **1y** (60.5 mg, 0.200 mmol, 1.0 eq.). After purification via Flash-Chromatography (P/EtOAc – 25/1 to 1/1), the product **2y** was obtained as a yellow oil (12.1 mg, 39.8 µmol, 20%).

^{1}H-NMR (599 MHz, $CDCl_3$): δ (ppm) = 8.21 (s, 1H), 7.69 (ddt, = 7.9, 1.3, 0.8 Hz, 1H), 7.61 – 7.55 (m, 2H), 7.49 – 7.43 (m, 2H), 7.41 – 7.31 (m, 2H), 7.19 (ddd, J = 8.2, 7.1, 1.2 Hz, 1H), 7.12 (ddd, J = 8.1, 7.1, 1.1 Hz, 1H), 3.86 (s, 2H), 3.46 (t, J = 6.8 Hz, 2H), 3.24 (t, J = 6.7 Hz, 2H), 1.85 – 1.80 (m, 2H), 1.80 – 174 (m, 2H).
^{13}C-NMR (151 MHz, $CDCl_3$): δ (ppm) = 169.9, 136.1, 136.0, 133.0, 129.3, 129.0, 128.5, 128.1, 122.6, 120.1, 119.8, 110.9, 106.7, 46.8, 46.1, 32.3, 26.4, 24.4.
HRMS (ESI): m/z calculated for $[M-H]^-$ $C_{20}H_{19}N_2O^-$ 303.1503, found 303.1501.
The analytical data match those reported in the literature.[33]

3-benzyl-2-phenyl-1*H*-indole (2z):

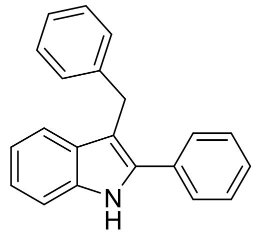

The reaction was performed according to **GP8** with 2,3-diphenylquinoline **1z** (56.3 mg, 0.200 mmol, 1.0 eq.). After purification via Flash-Chromatography (P/EtOAc – 10/1 to 8/1), the product **2z** was obtained as a white solid (33.3 mg, 0.118 mmol, 59%).

^{1}H-NMR (400 MHz, $CDCl_3$): δ (ppm) = 8.13 (s, 1H), 7.59 – 7.50 (m, 2H), 7.47 – 7.40 (m, 4H), 7.40 – 7.33 (m, 1H), 7.30 – 7.17 (m, 6H), 7.09 (ddd, J = 8.0, 7.0, 1.0 Hz, 1H), 4.30 (s, 2H).
^{13}C-NMR (101 MHz, $CDCl_3$): δ (ppm) = 141.6, 136.2, 135.6, 133.1, 129.7, 129.0, 128.5, 128.4, 128.0, 127.9, 125.9, 122.5, 119.9, 119.8, 111.3, 110.9, 30.6.
HRMS (ESI): m/z calculated for $[M-H]^-$ $C_{21}H_{16}N^-$ 282.1288, found 282.1286.
The analytical data match those reported in the literature.[34]

3-benzyl-2-isopropyl-1*H*-indole (2aa):

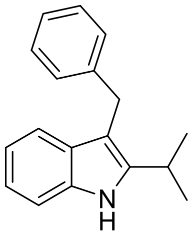

The reaction was performed according to **GP8** with 3-phenyl-2-propan-2-ylquinoline **1aa** (49.5 mg, 0.200 mmol, 1.0 eq.). After purification via Flash-Chromatography (P/Et_2O – 50/1 to 10/1), the product **2aa** was obtained as a yellow oil (11.4 mg, 45.7 µmol, 23%).

^{1}H-NMR (599 MHz, $CDCl_3$): δ(ppm) = 7.85 (s, 1H), 7.38 (d, *J* = 7.9 Hz, 1H), 7.32 (dq, *J* = 8.0, 0.9 Hz, 1H), 7.25 – 7.20 (m, 4H), 7.16 – 7.10 (m, 2H), 7.04 – 7.00 (m, 1H), 4.11 (s, 2H), 3.28 (hept, *J* = 7.0 Hz, 1H), 1.30 (d, *J* = 7.0 Hz, 6H).

^{13}C-NMR (151 MHz, $CDCl_3$): δ(ppm) = 141.9, 141.5, 135.3, 129.1, 128.4, 128.4, 125.8, 121.2, 119.4, 118.8, 110.5, 108.8, 30.1, 25.8, 22.8.

HRMS (ESI): m/z calculated for $[M-H]^-$ $C_{18}H_{18}N^-$ 248.1445, found 248.1443.

IR (Film): $\tilde{\nu}$ (cm^{-1}) = 3347, 3059, 3025, 2963, 2925, 1670, 1605, 1584, 1518, 1487, 1451, 1296, 1195, 1148, 1097, 745, 696, 493.

ethyl 2-(2-(tert-butyl)-1*H*-indol-3-yl)acetate (2ab):

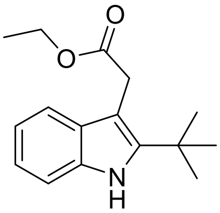

The reaction was performed according to **GP8** with ethyl 2-(tert-butyl)quinoline-3-carboxylate **1ab** (51.5 mg, 0.200 mmol, 1.0 eq.). After purification via Flash-Chromatography (P/EtOAc – 20/1 to 10/1), the product **2ab** was obtained as a white solid 41.5 mg, 0.160 mmol, 80%).

Adjusting the reaction conditions by employing **P3** (233 mg, 0.500 mmol, 2.5 eq.) as phosphine and a reduced amount of water (50 µL; 21 eq.) resulted after purification in a higher product formation (43.3 mg, 0.167 mmol, 83%).

^{1}H-NMR (599 MHz, $CDCl_3$): δ(ppm) = 7.94 (s, 1H), 7.57 – 7.52 (m, 1H), 7.29 (dt, *J* = 7.8, 0.8 Hz, 1H), 7.13 (ddd, *J* = 8.1, 7.1, 1.3 Hz, 1H), 7.09 (ddd, *J* = 8.1, 7.0, 1.1 Hz, 1H), 4.14 (q, *J* = 7.1 Hz, 2H), 3.90 (s, 2H), 1.48 (s, 9H), 1.23 (t, *J* = 7.1 Hz, 3H).

^{13}C-NMR (151 MHz, $CDCl_3$): δ(ppm) = 172.3, 143.3, 134.0, 129.9, 121.5, 119.7, 118.4, 110.4, 103.2, 60.8, 33.1, 31.6, 30.6, 14.4.

HRMS (ESI): m/z calculated for $[M-H]^-$ $C_{16}H_{20}NO_2^-$ 258.1500, found 258.1498.

IR (Solid): $\tilde{\nu}$ (cm^{-1}) = 3392, 2958, 2922, 1718, 1484, 1460, 1416, 1370, 1345, 1334, 1307, 1256, 1203, 1169, 1026, 797, 783, 739, 698, 600, 571.

Melting point: *T* (°C) = 74-75.

ethyl 2-(2-isopropyl-1*H*-indol-3-yl)acetate (2ac):

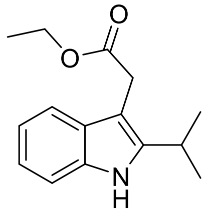

The reaction was performed according to **GP8** by with ethyl 2-isopropylquinoline-3-carboxylate **1ac** (48.7 mg, 0.200 mmol, 1.0 eq.). No product formation could be observed.

Adjusting the reaction conditions by employing **P3** (233 mg, 0.500 mmol, 2.5 eq.) as phosphine and a reduced amount of water (50 µL; 21 eq.) resulted in product formation. After purification via Flash-Chromatography (P/Et_2O – 10/1), the product **2ac** was obtained as a yellow oil (13.6 mg, 55.4 µmol, 28%).

^{1}H-NMR (400 MHz, $CDCl_3$): δ (ppm) = 7.90 (s, 1H), 7.61 – 7.51 (m, 1H), 7.30 (dd, *J* = 7.1, 1.7 Hz, 1H), 7.12 (pd, *J* = 7.1, 1.4 Hz, 2H), 4.12 (q, *J* = 7.2 Hz, 2H), 3.71 (s, 2H), 3.32 (hept, *J* = 6.9 Hz, 1H), 1.34 (d, *J* = 7.0 Hz, 6H), 1.23 (t, *J* = 7.1 Hz, 3H).

^{13}C-NMR (101 MHz, $CDCl_3$): δ (ppm) = 172.2, 142.2, 135.0, 128.6, 121.4, 119.7, 118.5, 110.6, 103.1, 60.8, 30.6, 25.7, 22.7, 14.3.

HRMS (ESI): m/z calculated for $[M-H]^-$ $C_{15}H_{18}NO_2^-$ 244.1343, found 244.1341.

IR (Film): $\tilde{\nu}$ (cm^{-1}) = 3393, 2963, 2922, 2871, 1725, 1462, 1367, 1302, 1262, 1244, 1177, 1154, 1033, 742.

***N,N*-diethyl-2-(2-methyl-1*H*-indol-3-yl)acetamide (2ad):**

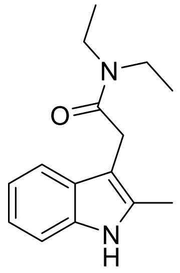

The reaction was performed according to **GP8** with *N,N*-diethyl-2-methylquinoline-3-carboxamide **1ad** (48.5 mg, 0.200 mmol, 1.0 eq.). After purification via Flash-Chromatography (P/EtOAc – 1/1 to 1/2), the product **2ad** was obtained as a yellow solid (24.9 mg, 0.102 mmol, 51%).

^{1}H-NMR (400 MHz, $CDCl_3$): δ (ppm) = 7.95 (s, 1H), 7.57 – 7.50 (m, 1H), 7.25 – 7.22 (m, 1H), 7.08 (pd, *J* = 7.1, 1.4 Hz, 2H), 3.74 (s, 2H), 3.42 – 3.28 (m, 4H), 2.38 (s, 3H), 1.11 (t, *J* = 7.1 Hz, 1H), 1.04 (t, *J* = 7.1 Hz, 1H).

^{13}C-NMR (101 MHz, $CDCl_3$): δ (ppm) = 170.8, 135.3, 132.2, 128.7, 121.2, 119.6, 118.2, 110.3, 105.6, 42.4, 40.5, 31.0, 14.2, 13.2, 12.0.

HRMS (ESI): m/z calculated for $[M-H]^-$ $C_{15}H_{19}N_2O^-$ 243.1503, found 243.1502.

IR (Solid): $\tilde{\nu}$ (cm^{-1}) = 3230, 3195, 2970, 2929, 1631, 1607, 1462, 1432, 1361, 1258, 1243, 1141, 1097, 1071, 1006, 731, 683, 591.

Melting point: *T* (°C) = 133-134.

1-phenyl-2-(2-(trifluoromethyl)-1*H*-indol-3-yl)ethan-1-one (2ae):

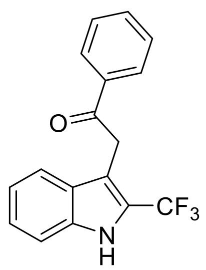

The reaction was performed according to **GP8** with 2,2,2-trifluoro-1-(2-phenylquinolin-3-yl)ethan-1-one **1ae** (60.2 mg, 0.200 mmol, 1.0 eq.). After purification via Flash-Chromatography (P/EtOAc – 15/1), the product **2ae** was obtained as a white solid (33.3 mg, 0.106 mmol, 55%).

^{1}H-NMR (500 MHz, $CDCl_3$): δ(ppm) = 8.19 (dd, *J* = 8.5, 1.0 Hz, 1H), 7.88 (s, 1H), 7.78 – 7.70 (m, 2H), 7.60 (ddd, *J* = 8.2, 6.8, 1.2 Hz, 1H), 7.39 – 7.33 (m, 2H), 7.33 – 7.28 (m, 1H), 7.24 – 7.20 (m, 2H), 4.35 (s, 2H).

^{13}C-NMR{^{19}F} (126 MHz, $CDCl_3$): δ(ppm) = 146.3, 145.2, 139.3, 138.8, 131.7, 130.2, 130.0, 129.5, 129.1, 129.0, 128.8, 127.3, 126.9, 122.3, 36.7.

^{19}F NMR (470 MHz, $CDCl_3$) δ(ppm) = -64.1.

HRMS (ESI): m/z calculated for $[M-H]^-$ $C_{17}H_{11}F_3NO^-$ 302.0798, found 302.0801.

11*H*-benzo[*a*]carbazole (2af):

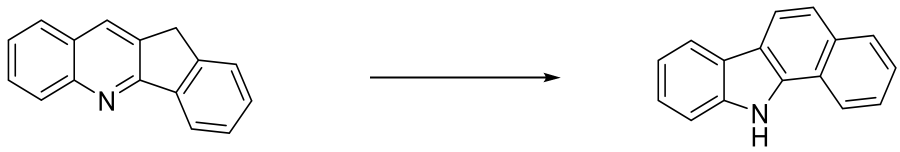

The reaction was performed according to **GP8** with 11*H*-indeno[1,2-*b*]quinoline **1af** (43.5 mg, 0.200 mmol, 1.0 eq.). After purification via Flash-Chromatography (P/DCM – 1/1), the side product **2af** was obtained as a white solid (20.9 mg, 96.2 µmol, 48%).

^{1}H-NMR (300 MHz, $CDCl_3$): δ(ppm) = 8.78 (s, 1H), 8.20 – 8.09 (m, 3H), 8.02 (dd, *J* = 8.2, 1.3 Hz, 1H), 7.67 (d, *J* = 8.6 Hz, 1H), 7.64 – 7.51 (m, 3H), 7.45 (ddd, *J* = 8.2, 7.1, 1.2 Hz, 1H), 7.32 (ddd, *J* = 8.0, 7.1, 1.0 Hz, 1H).

^{13}C-NMR (101 MHz, $CDCl_3$): δ(ppm) = 138.6, 135.0, 132.6, 129.2, 125.7, 125.4, 125.0, 124.3, 121.2, 120.6, 120.4, 120.1, 120.1, 119.5, 118.6, 111.2.

HRMS (ESI): m/z calculated for $[M-H]^-$ $C_{15}H_{12}N^-$ 206.0975, found 206.0974.

The analytical data match those reported in the literature.[35]

18-azatetracyclo[9.7.0.02,7.012,17]octadeca-1(11),2,4,6,12,14,16-heptaene (2ag):

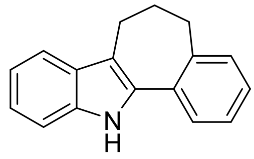

The reaction was performed according to **GP8** with 5,6-dihydrobenzo[c]acridine **1ag** (46.3 mg, 0.200 mmol, 1.0 eq.). After purification via Flash-Chromatography (P/EtOAc – 10/1 to 8/1), the product **2ag** was obtained as a white solid (33.4 mg, 0.143 mmol, 72%).

^{1}H-NMR (400 MHz, $CDCl_3$): δ (ppm) = 8.07 (s, 1H), 7.60 (t, J = 7.3 Hz, 2H), 7.41 (d, J = 8.1 Hz, 1H), 7.34 (td, J = 7.1, 2.3 Hz, 1H), 7.29 – 7.22 (m, 3H), 7.17 (t, J = 7.4 Hz, 1H), 3.15 (t, J = 6.9 Hz, 2H), 2.98 – 2.91 (m, 2H), 2.20 (dt, J = 10.8, 6.7 Hz, 2H).

^{13}C-NMR (101 MHz, $CDCl_3$): δ (ppm) = 142.5, 136.3, 132.8, 131.9, 130.1, 129.9, 127.1, 126.6, 125.3, 122.9, 119.6, 118.8, 114.8, 110.7, 35.4, 26.9, 26.2.

HRMS (ESI): m/z calculated for $[M-H]^-$ $C_{17}H_{14}N^-$ 232.1132, found 232.1131.

The analytical data match those reported in the literature.[36]

6,7,8,13-tetrahydro-5*H*-benzo[7,8]cycloocta[1,2-*b*]indole (2ah):

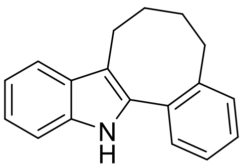

The reaction was performed according to **GP8** with 6,7-dihydro-5*H*-benzo[6,7]cyclohepta[1,2-*b*]quinoline **1ah** (49.1 mg, 0.200 mmol, 1.0 eq.). After purification via Flash-Chromatography (P/Et_2O – 20/1 to 10/1), the product 2ah was obtained as a yellow solid (15.1 mg, 61.1 µmol, 31%).

^{1}H-NMR (400 MHz, $CDCl_3$): δ (ppm) = 7.80 (s, 1H), 7.58 (d, J = 7.8 Hz, 0H), 7.48 (dd, J = 7.0, 2.0 Hz, 1H), 7.39 – 7.26 (m, 4H), 7.22 (ddd, J = 8.1, 7.1, 1.3 Hz, 1H), 7.15 (ddd, J = 8.0, 7.0, 1.1 Hz, 1H), 3.02 – 2.95 (m, 2H), 2.85 (m, 2H), 1.92 – 1.74 (m, 4H).

^{13}C-NMR (101 MHz, $CDCl_3$): δ (ppm) = 141.1, 136.2, 134.0, 132.4, 129.9, 129.5, 128.3, 127.8, 126.3, 122.3, 119.5, 118.8, 115.1, 110.6, 32.4, 31.5, 25.3, 22.2.

HRMS (ESI): m/z calculated for $[M-H]^-$ $C_{18}H_{16}N^-$ 246.1288, found 246.1287.

IR (Solid): $\tilde{\nu}$ (cm^{-1}) = 3389, 3053, 3016, 2924, 2898, 2869, 2843, 1486, 1454, 1441, 1428, 1356, 1301, 1228, 1151, 1101, 943, 743, 704, 675, 583, 518, 489, 458.

Melting point: T (°C) = 147-149.

4.4. Synthesis and Characterization of Side Products

The following reactions resulted in the formation of side products:

2-phenyl-1*H*-indole (3y):

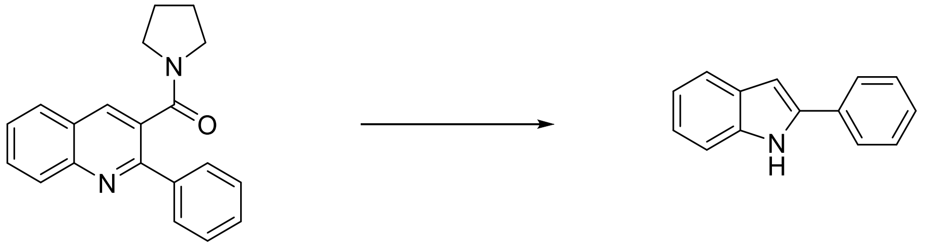

The reaction was performed according to **GP8** with (2-phenylquinolin-3-yl)(pyrrolidin-1-yl)methanone **1y** (60.5 mg, 0.200 mmol, 1.0 eq.). After purification via Flash-Chromatography (P/EtOAc – 25/1 to 1/1), the byproduct **3y** was obtained as a white solid (6.9 mg, 36 µmol, 18%).

^{1}H-NMR (599 MHz, $CDCl_3$): δ (ppm) = 8.33 (s, 1H), 7.69 – 7.66 (m, 2H), 7.64 (dq, *J* = 7.8, 0.9 Hz, 1H), 7.47 – 7.43 (m, 2H), 7.41 (dq, *J* = 8.1, 0.9 Hz, 1H), 7.35 – 7.32 (m, 1H), 7.21 (ddd, *J* = 8.1, 7.1, 1.2 Hz, 1H), 7.13 (ddd, *J* = 7.9, 7.1, 1.0 Hz, 1H), 6.84 (dd, *J* = 2.2, 1.0 Hz, 1H).

^{13}C-NMR (151 MHz, $CDCl_3$): δ (ppm) = 138.0, 137.0, 132.5, 129.4, 129.2, 127.9, 125.3, 122.5, 120.8, 120.4, 111.0, 100.2.

HRMS (ESI): m/z calculated for $[M-H]^-$ $C_{14}H_{10}N^-$ 192.0819, found 192.0817.

The analytical data match those reported in the literature.[37]

3-isopropyl-2-phenyl-5,10-dihydrobenzo[*g*]quinoline (3ai):

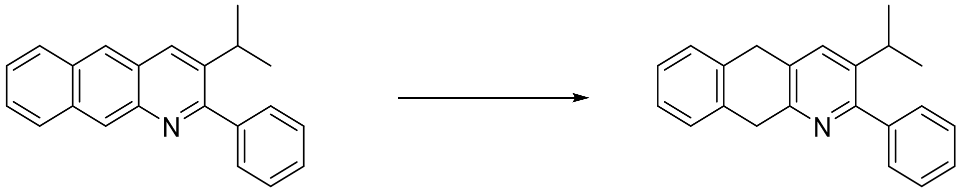

The reaction was performed according to **GP8** with 3-isopropyl-2-phenylbenzo[*g*]quinoline **1ai** (59.5 mg, 0.200 mmol, 1.0 eq.). After purification via Flash-Chromatography (P/EtOAc – 50/1 to 10/1), the side product **3ai** was obtained as a red solid (31.5 mg, 0.105 mmol, 53%).

^{1}H-NMR (500 MHz, $CDCl_3$): δ (ppm) = 7.59 (s, 1H), 7.47 – 7.42 (m, 4H), 7.41 – 7.37 (m, 1H), 7.36 – 7.31 (m, 2H), 7.25 – 7.21 (m, 2H), 4.18 (s, 2H), 4.06 (s, 2H), 3.11 (hept, *J* = 6.9 Hz, 1H), 1.19 (d, *J* = 6.9 Hz, 6H).

^{13}C-NMR (126 MHz, $CDCl_3$): δ (ppm) = 155.8, 153.7, 141.1, 139.5, 136.3, 135.4, 133.1, 129.8, 129.1, 128.3, 128.2, 127.7, 127.5, 126.5, 126.4, 38.7, 35.3, 28.9, 24.3.

HRMS (ESI): m/z calculated for $[M+H]^+$ $C_{22}H_{22}N^+$ 300.1747, found 300.1747.

IR (Solid): $\tilde{\nu}$ (cm^{-1}) = 3060, 2965, 2926, 2869, 2813, 1554, 1456, 1432, 1362, 1336, 1146, 1072, 1029, 981, 957, 928, 750, 739, 700, 612, 587, 575.

Melting point: *T* (°C) = 110-111.

***cis*-6-methoxy-3-methyl-2-phenyl-1,2,3,4-tetrahydroquinoline (3aj):**

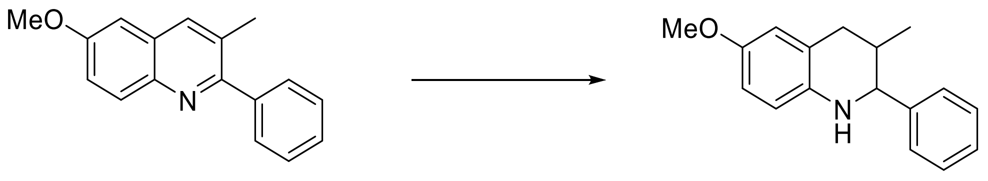

The reaction was performed according to **GP8** with 6-methoxy-3-methyl-2-phenylquinoline **1aj** (49.9 mg, 0.200 mmol, 1.0 eq.). After purification via Flash-Chromatography (P/Et_2O – 50/1 to 10/1), the side product **3aj** was obtained as a yellow solid (20.6 mg, 81.3 µmol, 41%).

^{1}H-NMR (400 MHz, $CDCl_3$): δ(ppm) = 7.39 – 7.22 (m, 5H), 6.68 – 6.60 (m, 2H), 6.53 (d, *J* = 8.5 Hz, 1H), 4.48 (d, *J* = 3.3 Hz, 1H), 3.91 – 3.83 (m, 1H), 3.76 (s, 3H), 3.02 (dd, *J* = 16.3, 5.2 Hz, 1H), 2.50 (dd, *J* = 16.3, 6.0 Hz, 1H), 2.36 – 2.23 (m, 1H), 0.83 (d, *J* = 6.9 Hz, 3H).

^{13}C-NMR (101 MHz, $CDCl_3$): δ(ppm) = 151.9, 143.3, 138.4, 128.3, 127.2, 127.2, 121.4, 115.2, 114.9, 113.1, 59.7, 55.9, 34.1, 32.3, 14.9.

HRMS (ESI): m/z calculated for $[M+H]^+$ $C_{17}H_{20}NO^+$ 254.1539, found 254.1539.

The analytical data match those reported in the literature.[26]

3-methyl-2-phenyl-1*H*-indole (3ak):

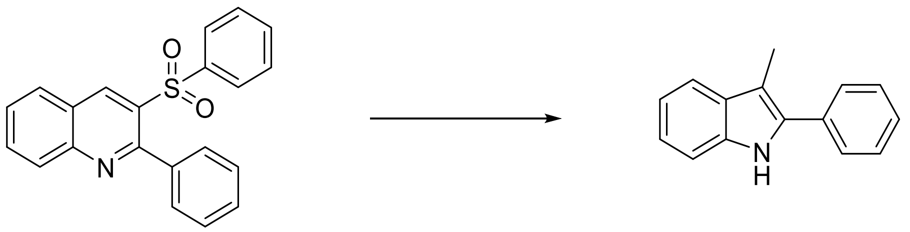

The reaction was performed according to **GP8** with 2-phenyl-3-(phenylsulfonyl)quinoline **1ak** (69.1 mg, 0.200 mmol, 1.0 eq.). After purification via Flash-Chromatography (P/Et_2O – 10/1), the side product **3ak** was obtained as a white solid (6.2 mg, 30 µmol, 15%).

^{1}H-NMR (400 MHz, $CDCl_3$): δ(ppm) = 8.01 (s, 1H), 7.65 – 7.54 (m, 3H), 7.48 (dd, *J* = 8.5, 7.0 Hz, 2H), 7.40 – 7.33 (m, 2H), 7.22 (ddd, *J* = 8.1, 7.0, 1.3 Hz, 1H), 7.16 (ddd, *J* = 8.0, 7.0, 1.1 Hz, 1H), 2.48 (s, 3H).

^{13}C-NMR (101 MHz, $CDCl_3$): δ(ppm) = 136.0, 134.2, 133.5, 130.2, 129.0, 127.9, 127.5, 122.5, 119.7, 119.1, 110.8, 108.9, 9.8.

HRMS (ESI): m/z calculated for $[M-H]^-$ $C_{15}H_{12}N^-$ 206.0975, found 206.0974.

The analytical data match those reported in the literature.[27]

3-cyclopentyl-2,3-dihydro-1*H*-cyclopenta[*b*]quinoline (3ak):

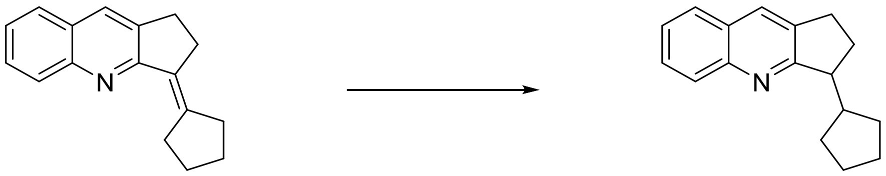

The reaction was performed according to **GP8** with 3-cyclopentylidene-2,3-dihydro-1*H*-cyclopenta[*b*]quinoline **1ak** (47.1 mg, 0.200 mmol, 1.0 eq.). After purification via Flash-Chromatography (P/Et_2O – 10/1), the side product **3ak** was obtained as a yellow oil (21.2 mg, 89.3 µmol, 45%).

^{1}H-NMR (599 MHz, $CDCl_3$): δ (ppm) = 8.04 (d, *J* = 8.4 Hz, 1H), 7.85 (s, 1H), 7.72 (dd, *J* = 8.1, 1.5 Hz, 1H), 7.60 (ddd, *J* = 8.4, 6.8, 1.5 Hz, 1H), 7.44 (ddd, *J* = 8.1, 6.8, 1.2 Hz, 1H), 3.30 (dt, *J* = 8.0, 6.2 Hz, 1H), 3.12 – 3.05 (m, 1H), 3.02 – 2.94 (m, 1H), 2.46 – 2.36 (m, 1H), 2.34 – 2.27 (m, 1H), 2.03 – 1.94 (m, 2H), 1.73 – 1.48 (m, 6H), 1.35 – 1.22 (m, 1H).

^{13}C-NMR (151 MHz, $CDCl_3$): δ (ppm) = 170.3, 147.9, 136.1, 130.3, 129.2, 128.2, 127.7, 127.5, 125.5, 49.7, 43.5, 31.1, 29.5, 29.1, 27.7, 25.5, 25.3.

HRMS (ESI): m/z calculated for $[M+H]^+$ $C_{17}H_{20}N^+$ 238.1590, found 238.1590.

IR (Film): $\tilde{\nu}$ (cm^{-1}) = 3059, 2943, 2862, 1618, 1566, 1496, 1445, 1401, 1303, 1202, 1146, 1127, 1016, 948, 899, 857, 782, 752, 742, 703, 615, 565, 476.

4.5. Unsuccessful Substrates

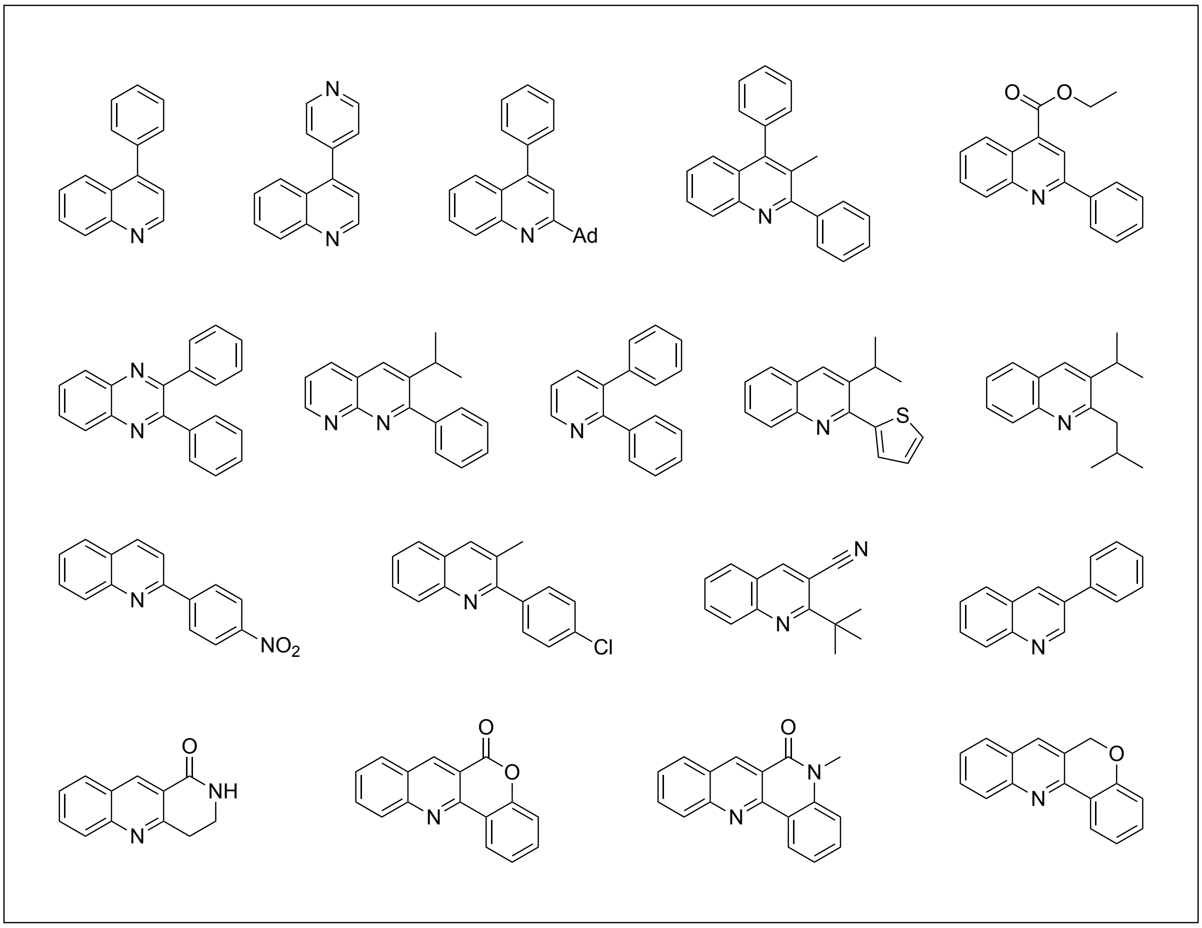

5. Scale-Up Experiments

For the scale-up experiments with our standard substrate **1a**, we wanted to use a more convenient reaction set-up, because ours with the photoboxes, doesn't apply due to the restricted solvent volume. Therefore, we used a Kessil lamp (456 nm, 30 W) and set up the reaction in a Schlenk flask. We found that after 16 h only 27% of the starting material was converted to the indole product. We checked a longer reaction time of 48 h and found a full consumption of the starting material with an 89% ^{1}H-NMR-yield of the indole against CH_2Br_2 as internal standard.

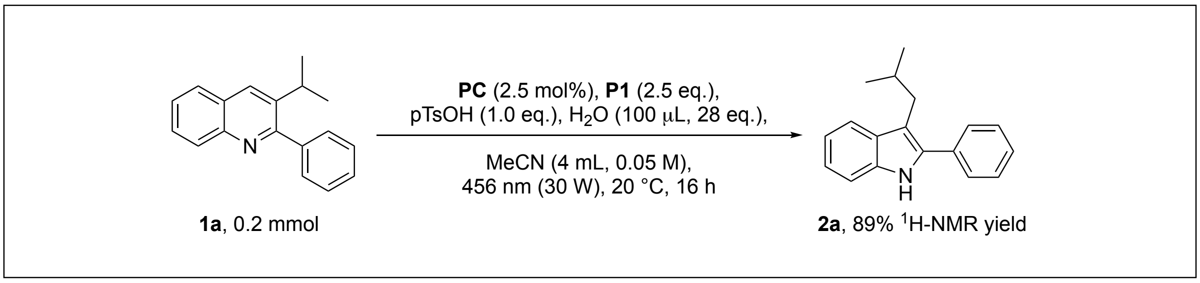

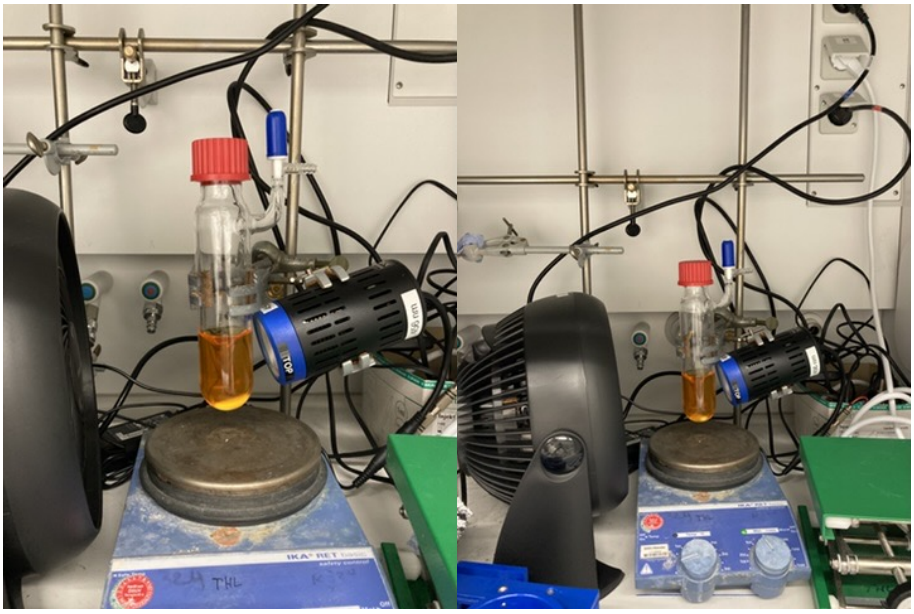

Figure 2 Set-up with Kessil-lamp (456 nm, 30 W) and fan for the scale-up.

Scale-up to 2 mmol with 2-phenylquinoline 1d:

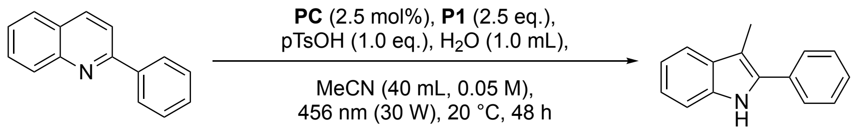

1d, 2.0 mmol

2d, 0.94 mmol, 47%

2-phenylquinoline **1d** (0.41 g, 2.0 mmol, 1.0 eq.), **PC** (56 mg; 50 µmol; 2.5 mol%), **P1** (1.76 g, 5.00 mmol, 2.5 eq.) and dried pTsOH (0.34 g; 2.0 mmol, 1.0 eq.) were added into a Schlenk-flask. The mixture was put under argon atmosphere. Dry MeCN (40 ml; 0.05 mol/L) and water (1.0 mL; 28 eq.) were added and the mixture was purged with argon for 10 min. The reaction mixture was stirred for 48 h at 20 °C and irradiated with blue light (Kessil lamp; 456 nm, 30 W). The reaction was washed into a round bottom flask with DCM and quenched by addition of sat. aq. Na_2CO_3 solution. The aqueous layer was extracted with DCM, the combined organic layer was dried over $MgSO_4$ and filtrated. The solvents were removed at the rotary evaporator under reduced pressure. After purification via Flash-Chromatography (P/EtOAc – 30/1 to 25/1), the product **2d** was obtained as a yellow solid (0.19 g, 0.94 mmol, 47%). The analytical data matches the above noted and is in accordance with the literature.[27]

Scale-Up to 4 mmol with 2-phenyl-3-propan-2-ylquinoline 1a:

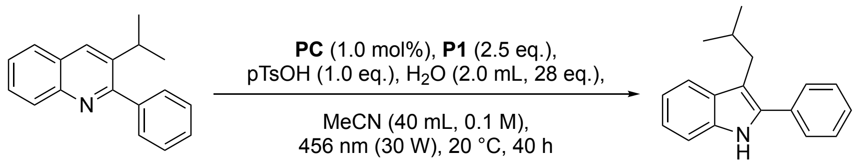

1a, 4.0 mmol

2a, 2.4 mmol, 60%
(70% rsm)

2-phenyl-3-propan-2-ylquinoline **1a** (0.99 g, 4.0 mmol, 1.0 eq.), **PC** (45 mg; 40 µmol; 1.0 mol%), **P1** (3.52 g, 10.0 mmol, 2.5 eq.) and dried pTsOH (0.69 g; 4.0 mmol, 1.0 eq.) were added into a Schlenk-flask. The mixture was put under argon atmosphere. Dry MeCN (40 ml; 0.10 mol/L) and water (2.0 mL; 28 eq.) were added and the mixture was purged with argon for 10 min. The reaction mixture was stirred for 40 h at 20 °C and irradiated with blue light (Kessil lamp; 456 nm, 30 W). The reaction was washed into a round bottom flask with DCM and quenched by addition of sat. aq. Na_2CO_3 solution. The aqueous layer was extracted with DCM, the combined organic layer was dried over $MgSO_4$ and filtrated. The solvents were removed at the rotary evaporator under reduced pressure. After purification via Flash-Chromatography (P/EtOAc – 30/1), the product **2a** was obtained as an off-white solid (0.60 g, 2.4 mmol, 60%; 71% yield in regard of remaining starting material – 150 mg, 0.607 mmol). The analytical data matches the above noted.

In this case the reaction mixture was a suspension due to the limited solubility of the phosphine. This also resulted in a loss of reactivity, which turned out to be problematic due to the slight decomposition of the resulting product during the reaction. Therefore, the reaction was quenched before a full conversion of the starting material was observed. Also, the isolation via column chromatography proved to be more difficult due to decomposition of the product on the column, during the isolation.

6. Mechanistic Investigations

6.1. Deuteration Experiments – Hydrogen Source

The deuteration experiments show that the H-atoms, which are incorporated within the molecule during the reductive rearrangement, are originating from the used water and not the solvent.

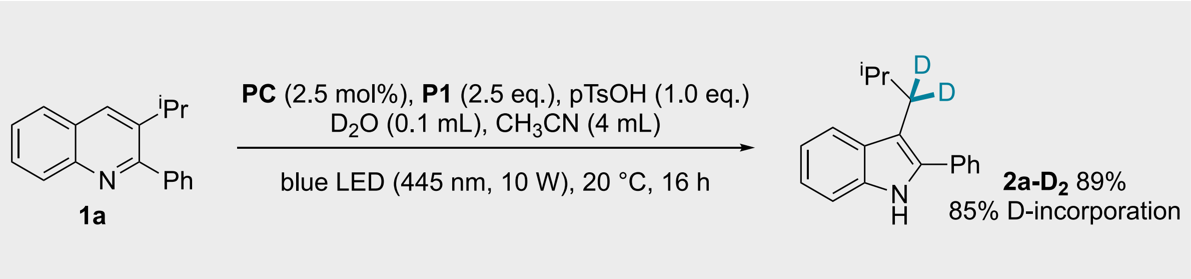

3-isobutyl-2-phenyl-1*H*-indole(2a) synthesized with CD_3CN:

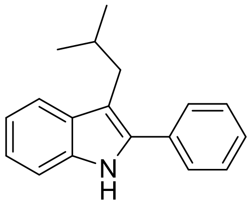

The reaction was performed according to **GP8** with 2-phenyl-3-propan-2-ylquinoline **1a** (49.5 mg, 0.200 mmol, 1.0 eq.). After purification via Flash-Chromatography (P/EtOAc – 30/1), the product **2a** was obtained as a white solid (41.5 mg, 0.184 mmol, 83%).

^{1}H-NMR (400 MHz, $CDCl_3$): δ (ppm) = 7.98 (s, 1H), 7.68 – 7.64 (m, 1H), 7.62 – 7.56 (m, 2H), 7.51 – 7.45 (m, 2H), 7.42 – 7.33 (m, 2H), 7.21 (ddd, *J* = 8.1, 7.0, 1.2 Hz, 1H), 7.15 (ddd, *J* = 8.1, 7.1, 1.1 Hz, 1H), 2.80 (d, *J* = 7.3 Hz, 2H), 2.08 (hept, *J* = 6.8 Hz, 1H), 0.92 (d, *J* = 6.6 Hz, 1H).
HRMS (ESI): m/z calculated for $[M\text{-}H]^-$ $C_{18}H_{18}N^-$ 248.1445, found 248.1442.
The here reported data matches the above shown.

D_2-3-isobutyl-2-phenyl-1*H*-indole (2a-D_2) synthesized with D_2O:

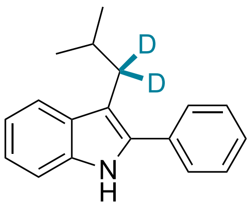

The reaction was performed according to **GP8** with 2-phenyl-3-propan-2-ylquinoline **1a** (49.5 mg, 0.200 mmol, 1.0 eq.) and 100 µL D_2O. After purification via Flash-Chromatography (P/EtOAc – 30/1 to 10/1), the product **2a-D_2** was obtained as a white solid (44.8 mg, 0.178 mmol, 89%).
^{1}H-NMR (400 MHz, $CDCl_3$): δ (ppm) = 7.99 (s, 1H), 7.68 (d, *J* = 7.8 Hz, 1H), 7.63 – 7.57 (m, 2H), 7.49 (t, *J* = 7.7 Hz, 2H), 7.43 – 7.35 (m, 2H), 7.30 – 7.13 (m, 2H), 2.80 (d, J = 7.3 Hz, 0.23H), 2.08 (hept, *J* = 6.7 Hz, 1H), 0.94 (d, J = 6.7 Hz, 6H).
HRMS (ESI): m/z calculated for $[M\text{-}H]^-$ $C_{18}H_{16}D_2N^-$ 250.1570, found 250.1567.
The molecule has an 85% D-incorporation in regard of the CH_2-group.

D_2-3-isobutyl-2-phenyl-1*H*-indole (2a-D_2):

^{1}H-NMR (400 MHz, $CDCl_3$)

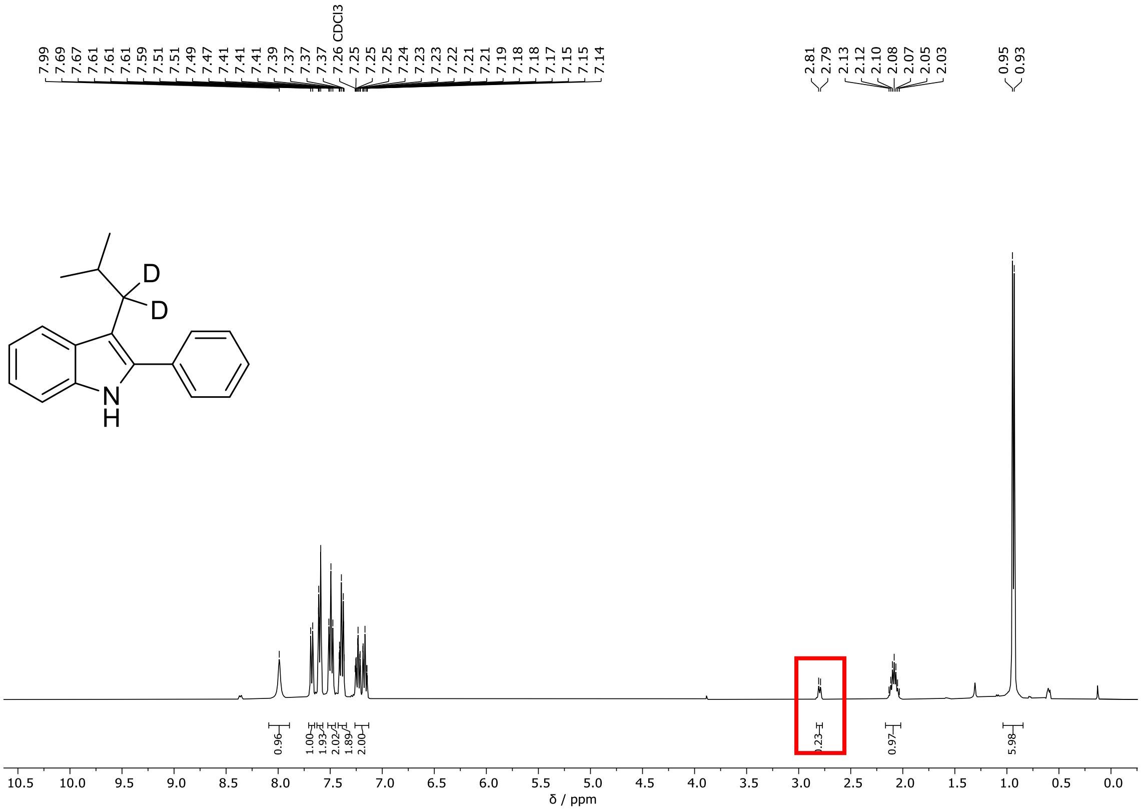

LabelChecker Results

Formula:	C18 H18 N
Mass (monoisotopic):	248,14
Difference Value:	0,001759
Error Sum:	0,042
Error (%):	0,568

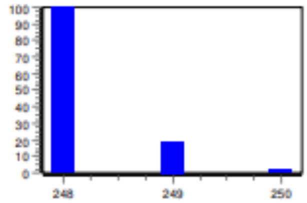

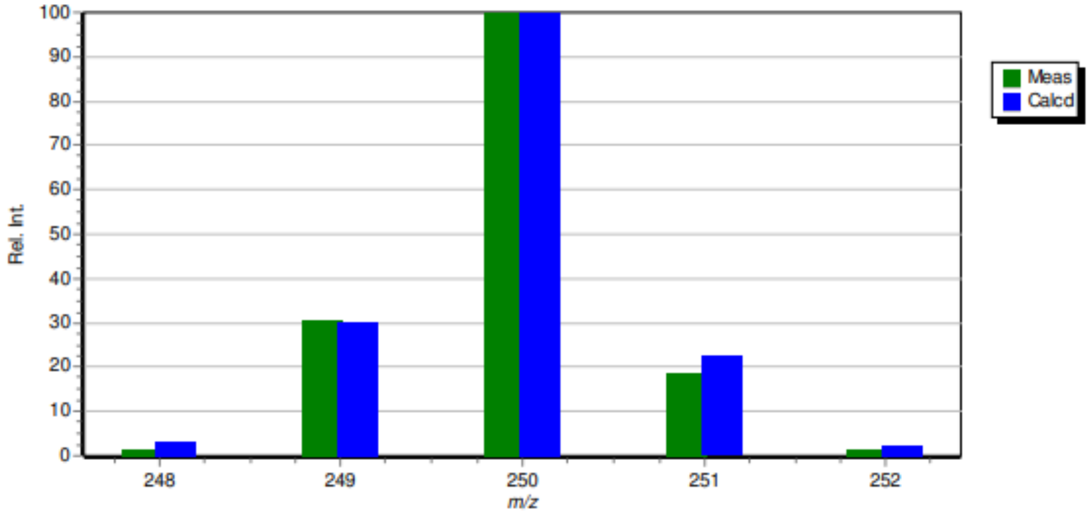

Deuterium: 0-fold (%): 1,20 2,32
Deuterium: 1-fold (%): 31,49 22,80
Deuterium: 2-fold (%): 100,00 72,39
Deuterium: 3-fold (%): 1,19 2,31
Deuterium: 4-fold (%): 0,00 0,00
Deuterium: 5-fold (%): 0,25 0,18
Deuterium: 6-fold (%): 0,00 0,00
Label Atom Sum: 1,75 (9,75%)

Isotope List used for fitting data:

m/z	intensity
248,14	38302172
249,14	781101995
250,05	208675
250,11	76048
250,14	2539486563
250,26	289919
250,31	45895
251,16	472273344
251,84	43429
252,16	40993700
253,05	45867
253,17	2552121
253,22	2400668

6.2. Protonation Studies

To showcase the proposed protonation of the quinoline in the reaction mixture, we did protonation studies of the reagents. Therefore, we set up all reagent combination between the quinoline **1a** (0.05 mmol, 1.0 eq.), phosphine **P1** (0.05 mmol, 1.0 eq.) and pTsOH (0.05 mmol, 1.0 eq.) in a mixture of CD_3CN/H_2O (2 mL, 3/1-mixture, 0.025 M), as described below. The used reagents are displayed in the respective ^{1}H-NMR/^{31}P-NMR spectra. For a better comparison of the chemical shifts the spectra are shown in an overlay.

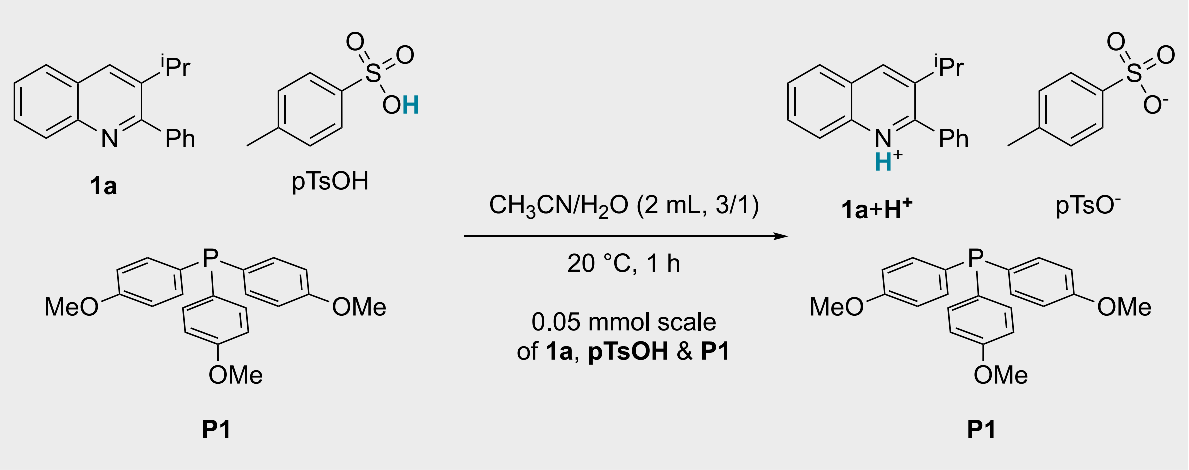

Investigation of the quinoline 1a:

^{1}H-NMR (300 MHz, CD_3CN/H_2O – 3/1)

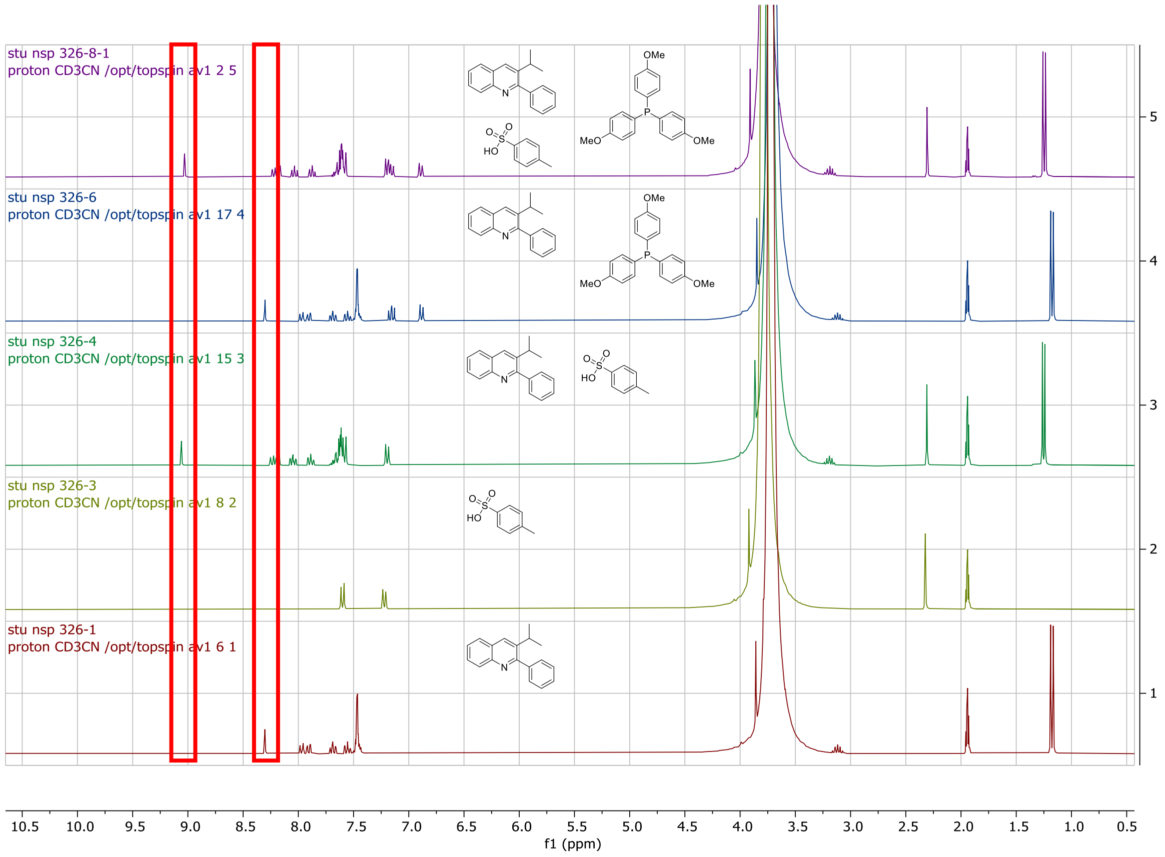

It can be observed that the ^{1}H-NMR signals of quinoline **1a** are shifted towards low field in combination with solely pTsOH and in combination with pTsOH and phosphine **P1**. This indicates that a protonation of the quinoline **1a** by pTsOH is viable (sample stu nsp 326-4), as well as a protonation is also possible in the reaction mixture when phosphine **P1** is present (sample stu nsp 326-8-1). This can be observed most prominent at the marked signal.

Investigation of the phosphine P1:

^{1}H-NMR (300 MHz, CD_3CN/H_2O – 3/1)

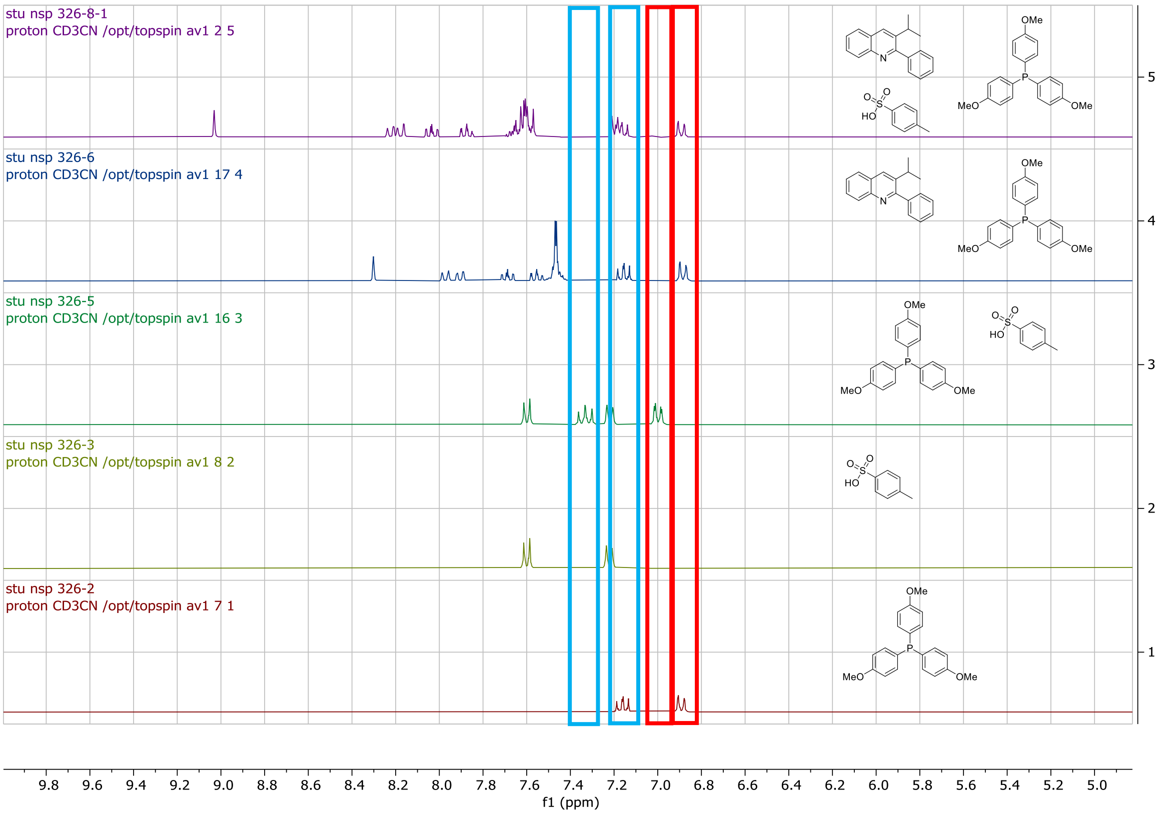

It can be observed that the ^{1}H-NMR signals of phosphine **P1** are shifted towards low field in combination with solely pTsOH. This indicates that a protonation of the phosphine **P1** by pTsOH is generally possible (sample stu nsp 326-5). In combination with the quinoline **1a** and pTsOH no shift of the signals is observed, showcasing that in this combination the phosphine **P1** doesn't get protonated in the mixture by the pTsOH (sample stu nsp 326-4). This is also supported by the below shown ^{31}P-NMR spectra, where only a shift of the phosphorus signal towards low field is observed in combination with pTsOH, whereas no shift is observed when also the quinoline **1a** is added to the mixture (samples stu nsp 326-5 and stu nsp 326-8-1). This can be observed most prominent at the marked signal in the spectra overlays.

^{31}P-NMR (122 MHz, CD_3CN/H_2O – 3/1)3

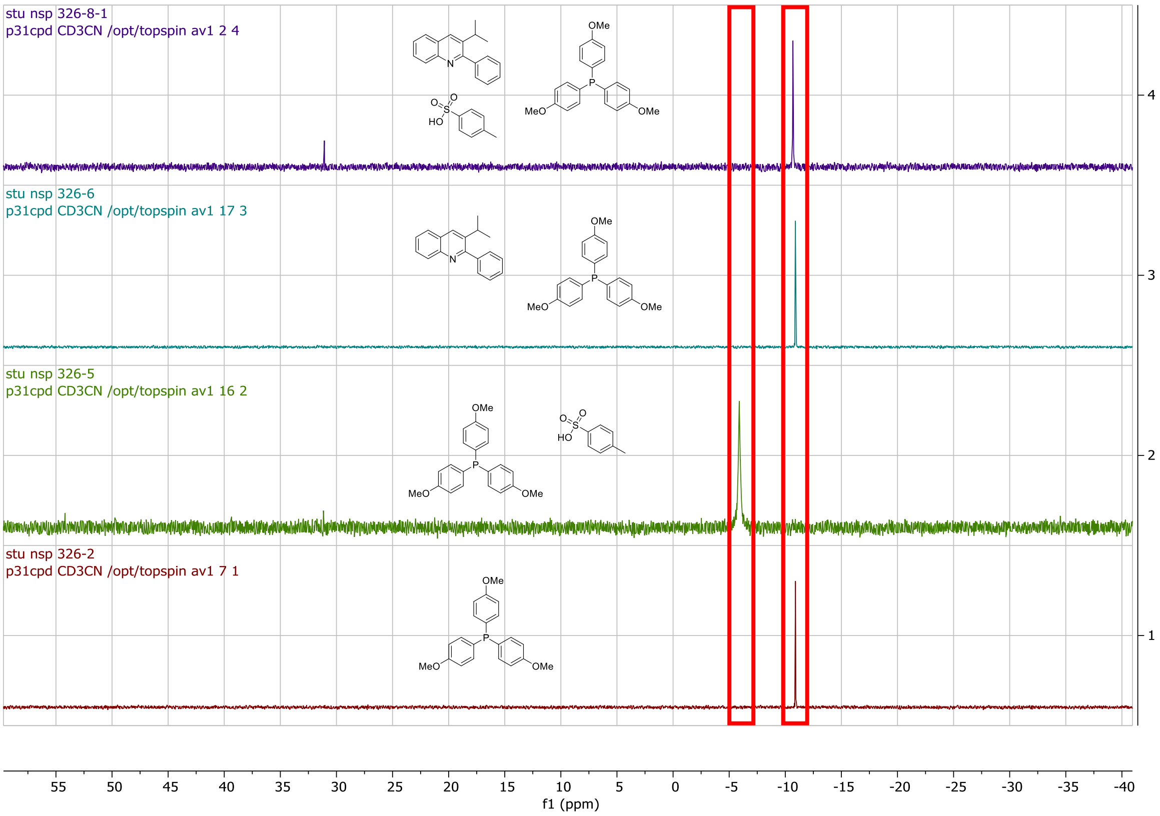

6.3. Cyclic Voltammetry

The cyclic voltammetry (CV) measurements were performed in an argon-filled glove box. A solution of the corresponding substrate in a 0.1 M NBu_4PF_6 solution in MeCN was used. The MeCN was dried over molecular sieve (3Å) prior to use and stored in the glove box. The conducting salt NBu_4PF_6 used was dried at 75 °C under vacuum and stored in the glove box. The measurement was performed at a scan rate of 200 mV/s. A three-electrode setup with a Pt working electrode, a Pt counter electrode, and an Ag/Ag^+ reference electrode was used. The recorded CVs were referenced to Fc/Fc^+ as an internal standard and the redox potentials were determined according to peak potentials (E_p). The measurements show a reduction for **1a** at E_p = -2.61 V vs. Fc/Fc^+ (E = -2.21 V vs. SCE) and for **1a HCl** at E_p = -0.95 V vs. Fc/Fc^+ (E = -0.55 V vs. SCE) and E_p = -2.52 V vs. Fc/Fc^+ (E = -2.12 V vs. SCE) – values recalculated for SCE.[38] For **1a HCl**, a new reduction potential has been found, which makes reduction easier compared to **1a**. The CV diagrams show virtually reversible reductions for **1a** and **1a HCl**.

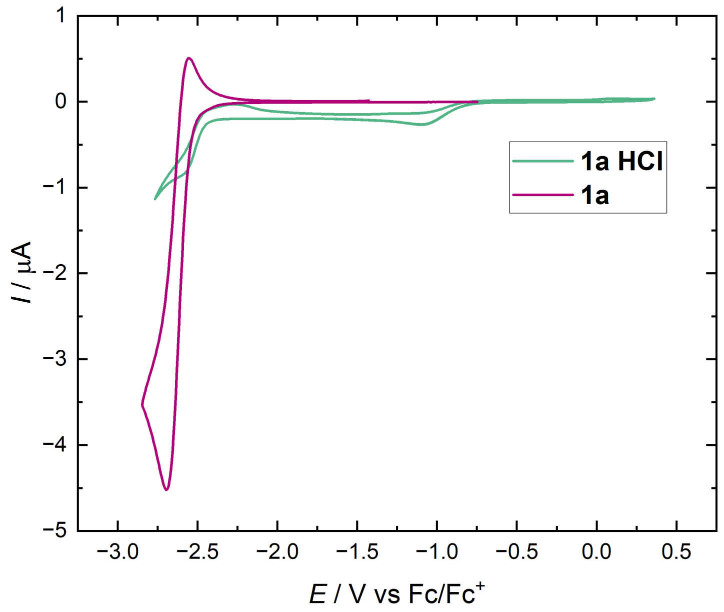

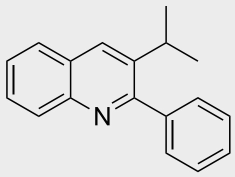

1a

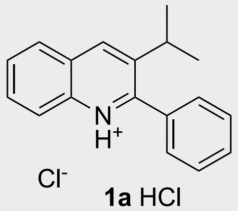

Cl⁻ **1a** HCl

E(**1aH⁺**/**1aH**) = -0.55 V vs. SCE

E(**1**/**1⁻**) = -2.21 V vs. SCE

$E(Ir^{4+}/{}^*Ir^{3+})$ = -0.89 V vs. SCE

6.4. Stern–Volmer Quenching

The Stern–Volmer fluorescence quenching experiments were recorded using the Jasco FP8550 spectrofluorometer at 20 °C. The following measurement parameters were set: excitation bandwidth = 5 nm, data interval = 0.2 nm, scan speed = 500 nm/min, response time = 2 s. The fluorescence spectra were recorded in a wavelength range from λ_{Flu} = 395 to 800 nm at an excitation wavelength of λ_{ex} = 380 nm. The measurement was performed in a QS *High Precision Cell* quartz cuvette (V = 1.4 mL, H x W x D = 46 mm x 12.5 mm x 12.5 mm) from *HellmaAnalytics* for fluorescence measurements, which was sealed with a PTFE stopper. To prepare the measurement solution, stock solutions of the photocatalyst **PC** (c = 1.5 $\cdot$ 10^{-3} mmol/L), substrate **1a HCl**, and the phosphines **P1** and **P3** (c = 0.25 mmol/L) used were prepared. For each measurement, the measuring solution was prepared from the stock solutions and degassed in the cuvette for 30 seconds by introducing argon. Multiple determinations were carried out, with the solution being replaced and degassed again between measurements. The measuring solution has a concentration of c = 4.5 $\cdot$ 10^{-4} mmol/L for the photocatalyst. The concentrations of the quenchers are listed in the corresponding tables.

c(**P1**)	0.000	0. 013	0.026	0.040	0.053	0.066	0.079
I	7168	6676	6276	5867	5508	5168	4863
I_0/I	1.000	1.074	1.142	1.222	1.301	1.387	1.474

c(**P3**)	0.000	0. 013	0.026	0.039	0.052	0.078
I	7294	7076	6960	6835	6670	6434
I_0/I	1.000	1.031	1.049	1.067	1.093	1.134

c(**1a HCl**)	0.000	0. 013	0.025	0.038	0.050	0.063	0.075
I	7560	7316	6928	6718	6396	6186	5877
I_0/I	1.000	1.033	1.091	1.125	1.182	1.222	1.286

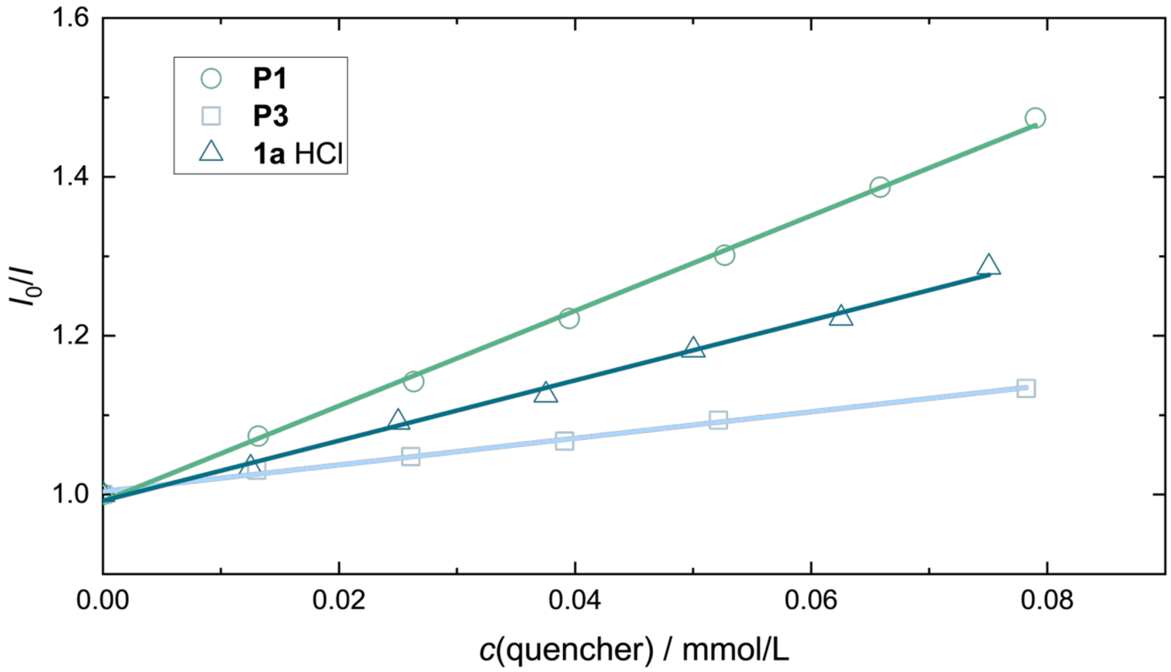

Equation	I0/I = KSV · c +1		
Plot	P3	P1	1a HCl
Weight	No Weighting		
Intercept	1.00396 ± 0.00249	0.992 ± 0.00521	0.99216 ± 0.00563
Slope	1.67317 ± 0.05763	5.98854 ± 0.10968	3.79009 ± 0.1249
Residual Sum of Squares	5.27053E-5	2.92029E-4	3.41749E-4
Pearson's r	0.99764	0.99916	0.9973
R-Square (COD)	0.99528	0.99833	0.9946
Adj. R-Square	0.9941	0.99799	0.99352

According to the Stern-Volmer Quenching experiments it can be stated that none of the used components – phosphines **P1** and **P3** as well as quinoline **1a HCl** are quenching with a significant higher rate. Therefore, an initial oxidative quenching of the **PC** via the phosphines as well as reductive quenching via the protonated quinoline in the reaction can't be ruled out.

6.5. Deuteration Experiments — Investigations for 2-monosubstituted Quinolines

To confirm the proposed reaction mechanism - also for the 2-monosubstituted quinolines - in contrast to the previously reported neophile-like mechanism[5], we did label experiments. Therefore, we subjected 2-phenylquinoline, 2-phenylquinoline-3-D and 2-phenylquinoline-4-D to the reaction conditions. In case of 2-phenylquinoline we employed D_2O to check on the general D-incorporation for 2-monosubstituted quinolines. We observed an overall 78% D-incorporation, which is higher than the expected 66%. We suspected a potential H/D-exchange in 3-position due to reversibility between the intermediates **I-1** and **I-2**. To confirm, we subjected quinoline **1d** to the reaction conditions employing D_2O and TripSH instead of the phosphine to prevent the rearrangement to see whether we would observe a D-incorporation in the starting material due to H/D-exchange and HAT-processes. This was the case, which can be seen by a 33% D incorporation over 3- and 4-positions (see below for further information). In the other cases we used H_2O to see whether we have in a remaining D-incorporation within one of the products. It could be observed that for 1d-3-D as starting material a remaining 18% D-incorporation was observed and for 1d-4-D a negligible 2%. Thus, we conclude that for the monosubstituted quinolines the proposed mechanism is also proceeding. The results are shown below. The synthesis of the deuterated quinolines is described at the end of this section.

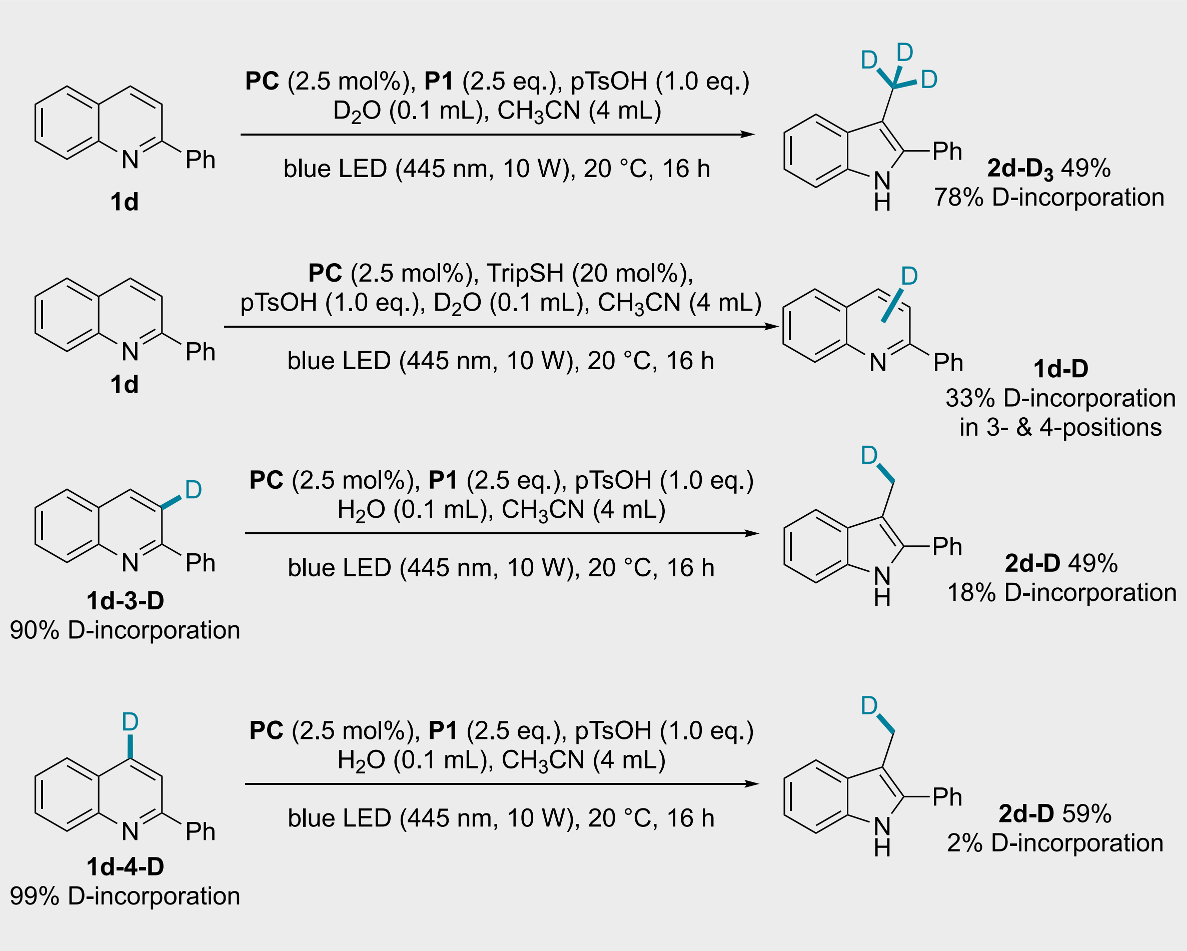

D_3-3-methyl-2-phenyl-1*H*-indole (2d-D_3) synthesized with D_2O:

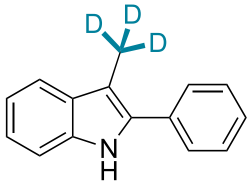

The reaction was performed according to **GP8** with 2-phenylquinoline **1d** (41.1 mg, 0.200 mmol, 1.0 eq.) and 100 µL D_2O. After purification via Flash-Chromatography (P/EtOAc – 30/1 to 25/1), the product **2d-D_3** was obtained as an off-white solid (20.5 mg, 97.5 µmol, 49%).

^{1}H-NMR (400 MHz, $CDCl_3$): δ (ppm) = 8.01 (s, 1H), 7.65 – 7.56 (m, 3H), 7.53 – 7.46 (m, 2H), 7.43 – 7.32 (m, 2H), 7.30 – 7.12 (m, 2H), 2.51 – 2.42 (m, 0.60H).

HRMS (ESI): m/z calculated for $[M-H]^-$ $C_{15}H_9D_3N^-$ 206.1164, found 209.1160.

The molecule has an 78% D-incorporation in regard of the CH_3-group.

D_3-3-methyl-2-phenyl-1*H*-indole (2d-D_3):

^{1}H-NMR (400 MHz, $CDCl_3$)

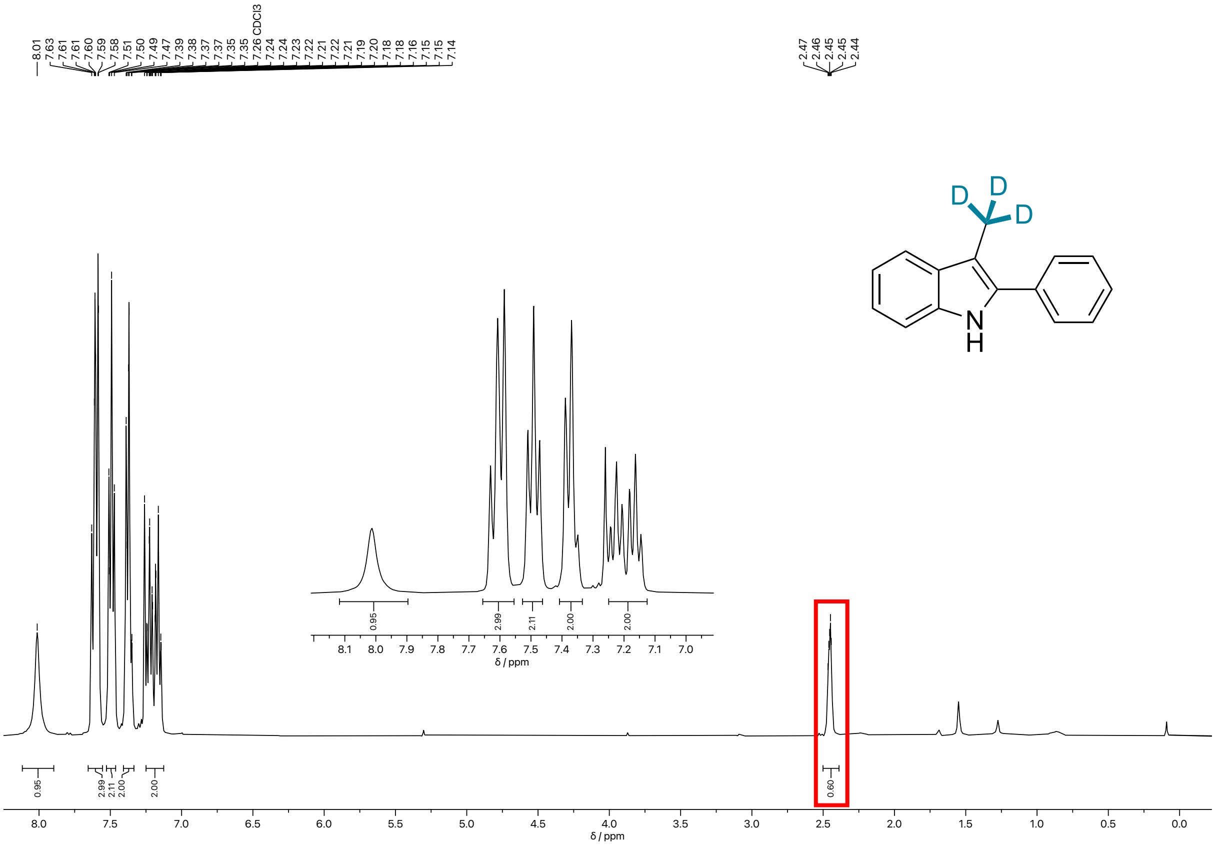

LabelChecker Results

Formula:	C15 H12 N
Mass (monoisotopic):	206,10
Difference Value:	0,000006
Error Sum:	0,003
Error (%):	0,061

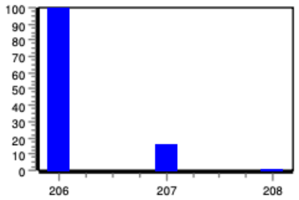

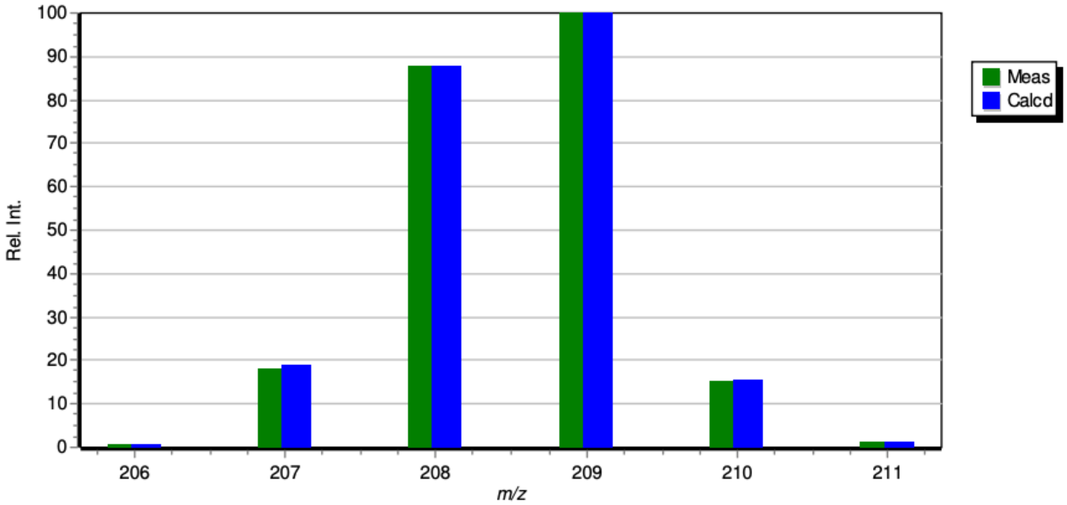

Deuterium: 0-fold (%): 1,08 0,49
Deuterium: 1-fold (%): 22,08 9,93
Deuterium: 2-fold (%): 99,19 44,61
Deuterium: 3-fold (%): 100,00 44,97
Label Atom Sum: 2,34 (19,51%)

Isotope List used for fitting data:

m/z	intensity
206,10	4456527
207,07	3560439
207,10	87904928
208,11	422748192
209,12	480167808
210,12	72904864
211,12	5125306

2-phenylquinoline-D (1d-D):

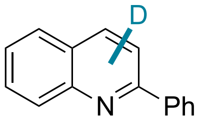

The reaction was performed according to **GP8** with 2-phenylquinoline **1d** (41.1 mg, 0.200 mmol, 1.0 eq.), 100 µL D_2O & TripSH (9.5 mg, 40 µmol, 20 mol%,) instead of **P1**. After purification via Flash-Chromatography (P/EtOAc – 30/1 to 25/1), **1d-D** was reisolated.

^{1}H-NMR (400 MHz, $CDCl_3$): δ (ppm) = 8.26 – 8.22 (m, 0.92H), 8.20 – 8.14 (m, 3H), 7.89 (m, 0.75H), 7.84 (d, J = 8.1 Hz, 1H), 7.74 (ddd, J = 8.5, 6.9, 1.5 Hz, 1H), 7.57 – 7.51 (m, 3H), 7.50 – 7.44 (m, 1H).

HRMS (ESI): m/z calculated for $[M-H]^-$ $C_{15}H_{11}DN^+$ 207.1027, found 207.1029.

The molecule has an overall 33% D-incorporation in regard of the CH-groups in 3- and 4-position. Herein 25% D-incorporation are at 3-position and 8% D-incorporation are at 4-position. Furthermore, 27% of the molecules have a mono-D-incorporation whereas 3% have a di-D-incorporation.

2-phenylquinoline-D (1d-D):

^{1}H-NMR (400 MHz, $CDCl_3$)

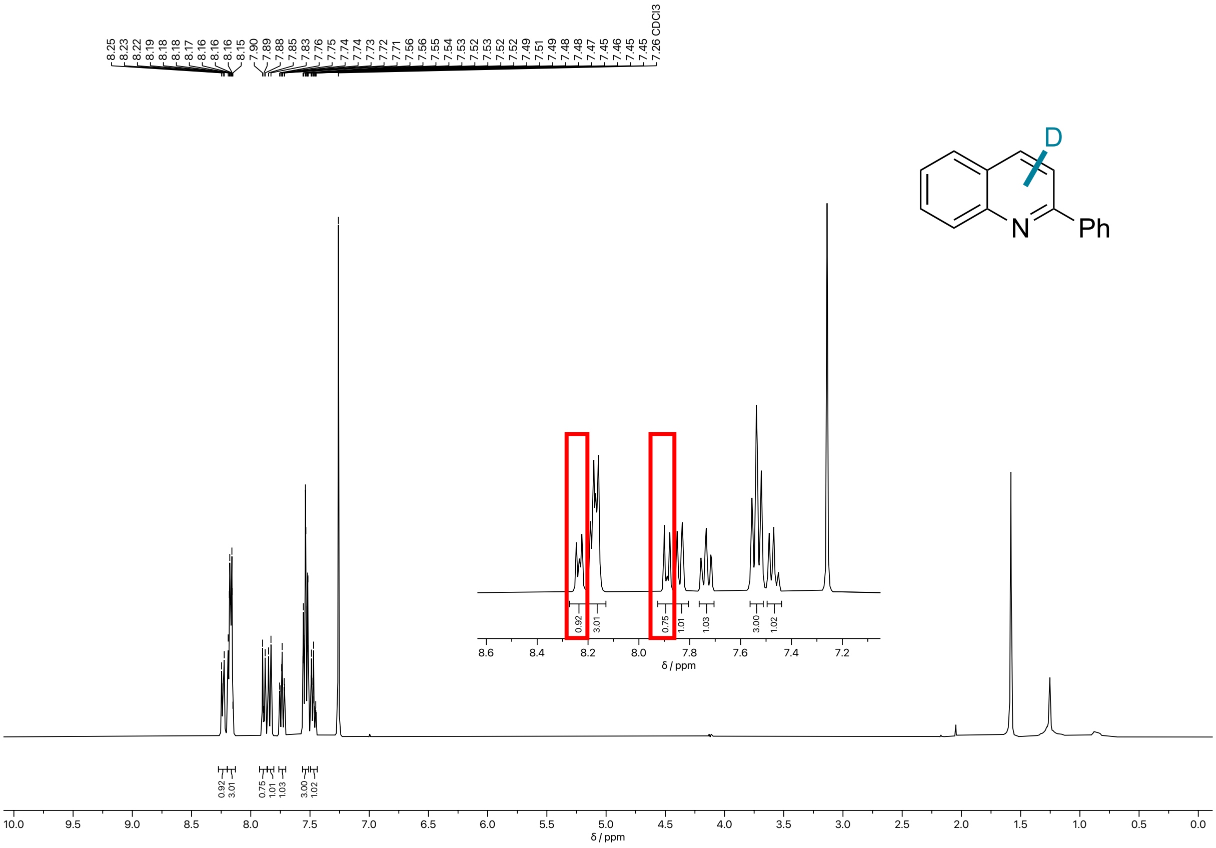

LabelChecker Results

Formula:	C15 H12 N
Mass (monoisotopic):	206.10
Difference Value:	0.000018
Error Sum:	0.004
Error (%):	0.092

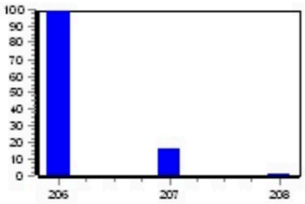

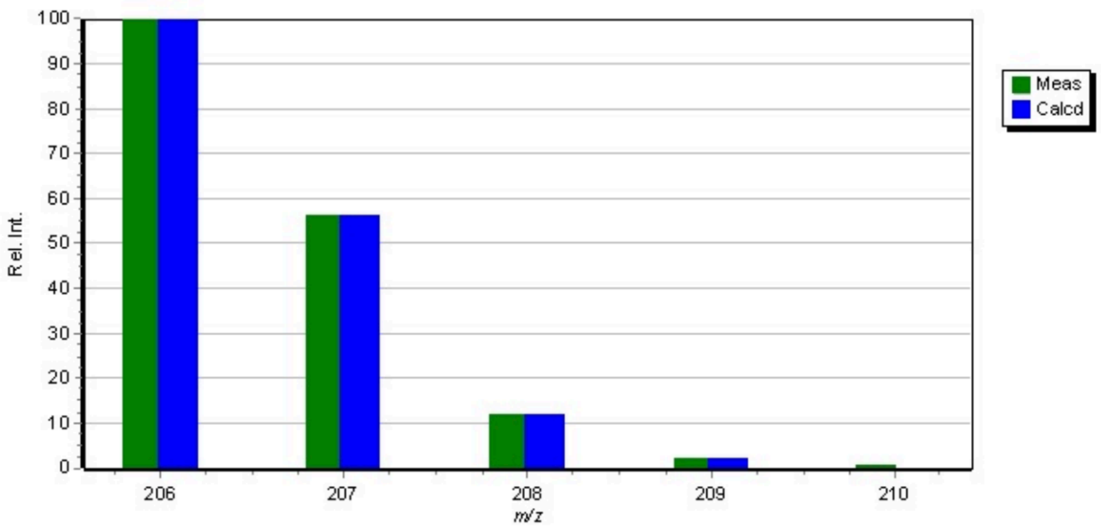

Deuterium: 0-fold (%): 100.00 69.23
Deuterium: 1-fold (%): 39.30 27.21
Deuterium: 2-fold (%): 4.17 2.88
Deuterium: 3-fold (%): 0.98 0.68
Label Atom Sum: 0.35 (2.92%)

Isotope List used for fitting data:

m/z	intensity
206.10	1441774592
207.10	807844096
208.11	173784672
209.11	31543979
210.12	9148663
211.13	2572977

D-3-methyl-2-phenyl-1*H*-indole (2d-D) synthesized from 1d-3-D:

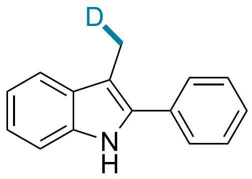

The reaction was performed according to **GP8** with 2-phenylquinoline-3-D **1d-3-D** (41.3 mg, 0.200 mmol, 1.0 eq.). After purification via Flash-Chromatography (P/EtOAc – 30/1 to 25/1), the product **2d-D** was obtained as an off-white solid (20.6 mg, 98.9 µmol, 49%).

^{1}H-NMR (400 MHz, $CDCl_3$): δ (ppm) = 8.02 (s, 1H), 7.65 – 7.56 (m, 3H), 7.55 – 7.44 (m, 2H), 7.41 – 7.32 (m, 2H), 7.24 – 7.12 (m, 2H), 2.50 – 2.43 (m, 2.54H).

HRMS (ESI): m/z calculated for $[M\text{-}H]^-$ $C_{15}H_{11}DN^-$ 207.1038, found 207.1034.

The molecule has a remaining 18% D-incorporation in regard of the methyl group (0.18D in regard of 0.90D from **1d-3-D** starting material). In this case the ^{1}H-NMR shows an even higher remaining D-incorporation within the molecule than the stated mass analysis.

D-3-methyl-2-phenyl-1*H*-indole (2d-D):

^{1}H-NMR (400 MHz, $CDCl_3$)

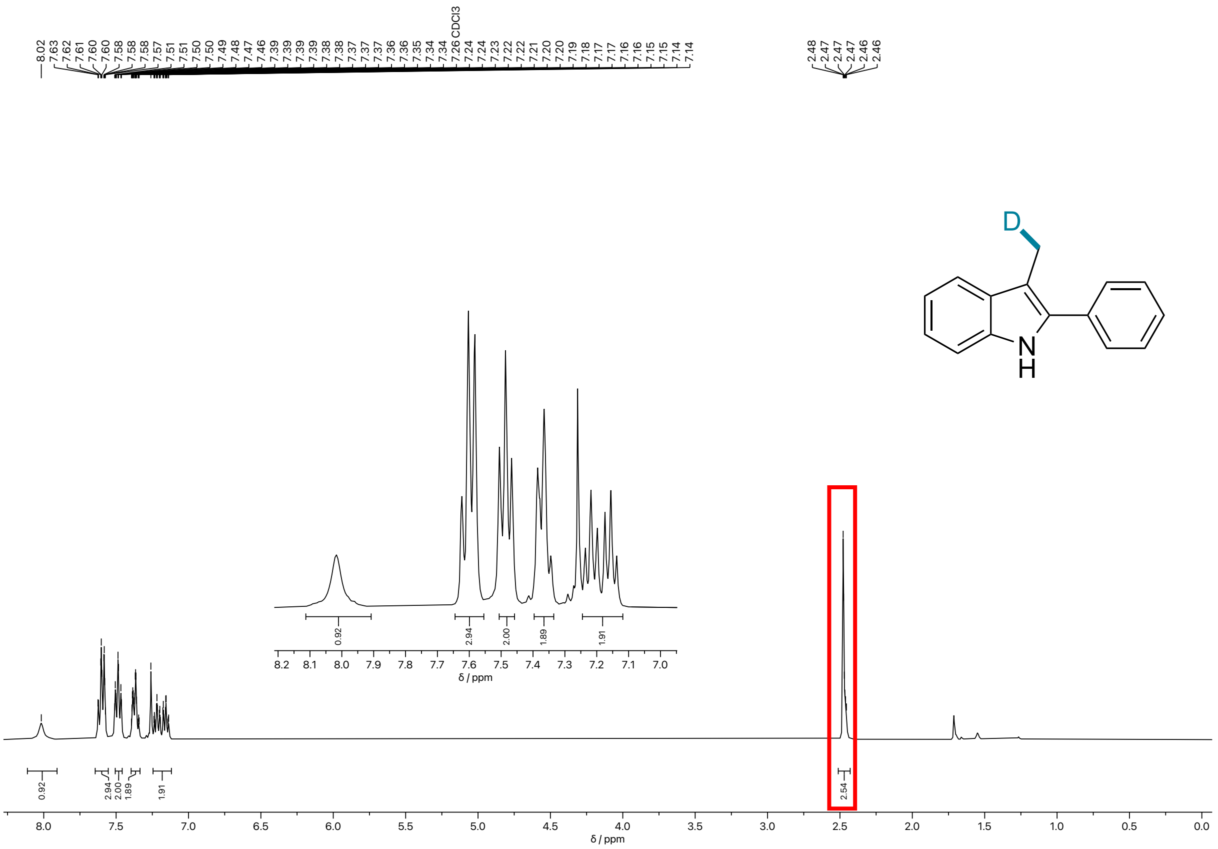

LabelChecker Results

Formula:	C15 H12 N
Mass (monoisotopic):	206,10
Difference Value:	0,000023
Error Sum:	0,005
Error (%):	0,088

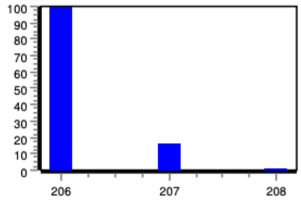

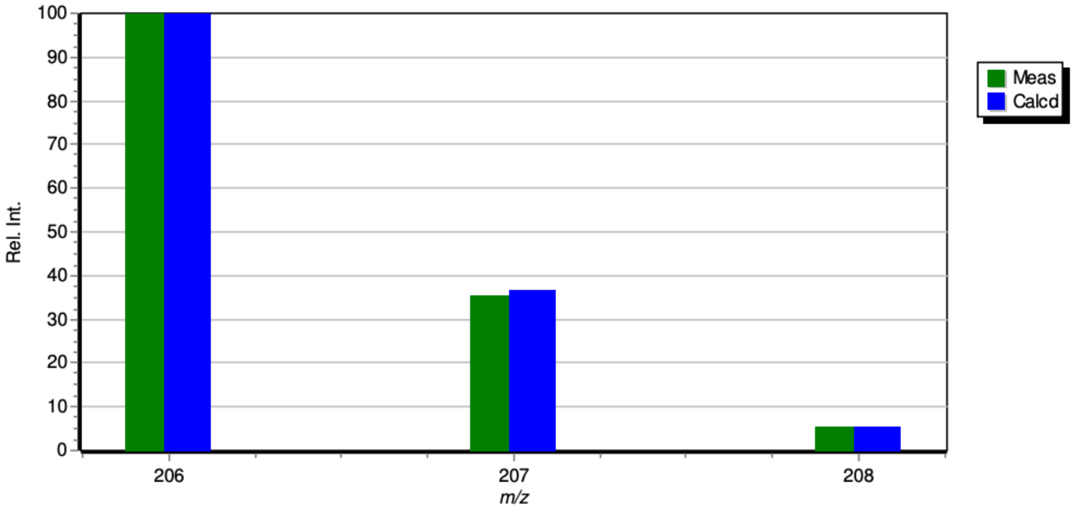

Deuterium: 0-fold (%): 100,00 82,74
Deuterium: 1-fold (%): 19,98 16,53
Deuterium: 2-fold (%): 0,88 0,73
Deuterium: 3-fold (%): 0,00 0,00
Deuterium: 4-fold (%): 0,00 0,00
Label Atom Sum: 0,18 (1,50%)

Isotope List used for fitting data:

m/z	intensity
206,02	156362
206,09	892417793
206,17	129374
207,07	9678677
207,10	317859360
208,07	1500784
208,11	48606148

D-3-methyl-2-phenyl-1*H*-indole (2d-D) synthesized from 1d-4-D:

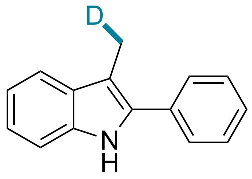

The reaction was performed according to **GP8** with 2-phenylquinoline-4-D **1d-4-D** (41.3 mg, 0.200 mmol, 1.0 eq.). After purification via Flash-Chromatography (P/EtOAc – 30/1 to 25/1), the product **2d-D** was obtained as an off-white solid (24.6 mg, 0.118 mmol, 59%).

^{1}H-NMR (400 MHz, $CDCl_3$): δ (ppm) = 8.02 (s, 1H), 7.64 – 7.57 (m, 3H), 7.55 – 7.43 (m, 2H), 7.40 – 7.33 (m, 2H), 7.22 (td, J = 8.0, 7.6, 1.3 Hz, 1H), 7.16 (td, J = 7.5, 7.0, 1.1 Hz, 1H), 2.48 (s, 2.96H).

HRMS (ESI): m/z calculated for $[M-H]^-$ $C_{15}H_{11}DN^-$ 207.1038, found 207.1028.

The molecule has a remaining 2% D-incorporation in regard of the methyl group (0.02D in regard of 1.0D from **1d-4-D** starting material).

D-3-methyl-2-phenyl-1*H*-indole (2d-D):

^{1}H-NMR (400 MHz, $CDCl_3$)

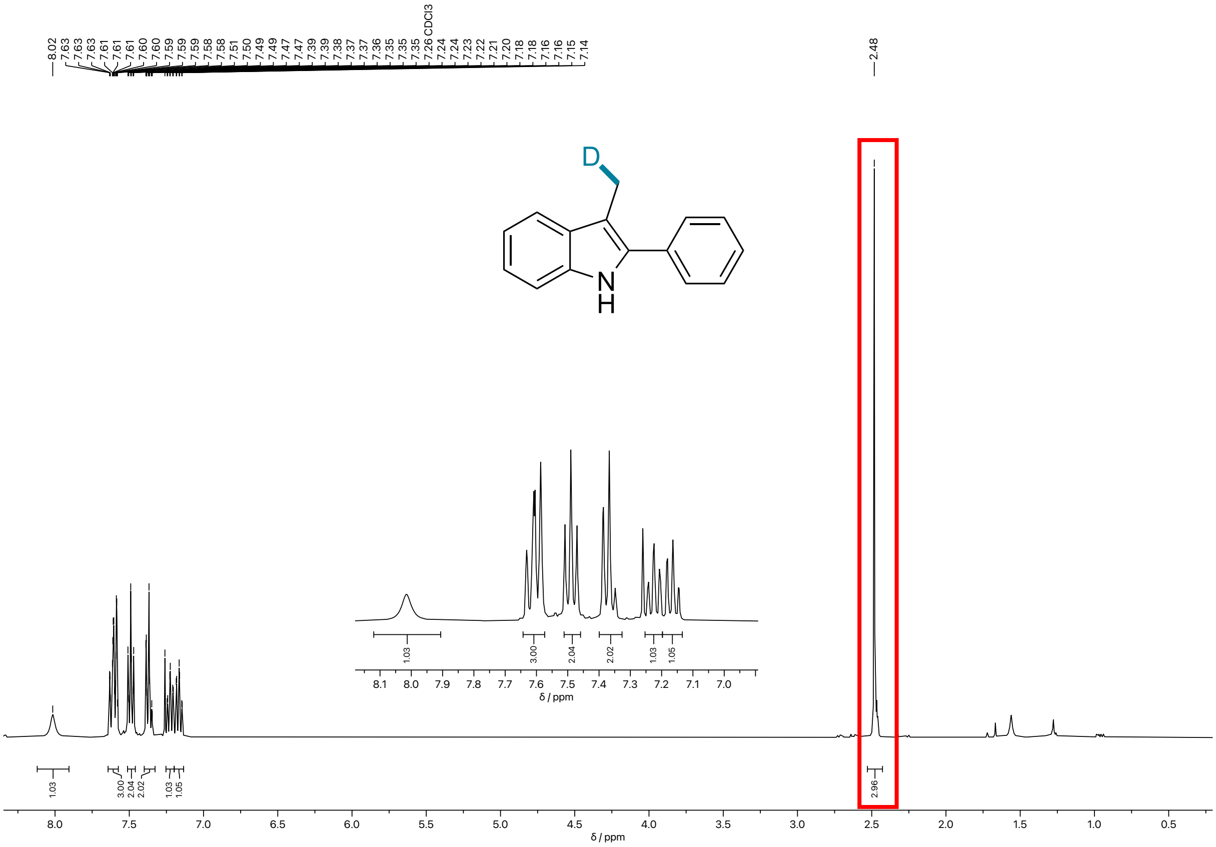

LabelChecker Results

Formula:	C15 H12 N
Mass (monoisotopic):	206,10
Difference Value:	0,000155
Error Sum:	0,012
Error (%):	0,433

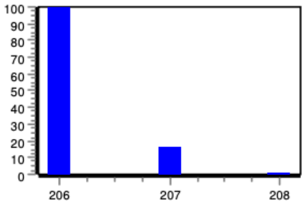

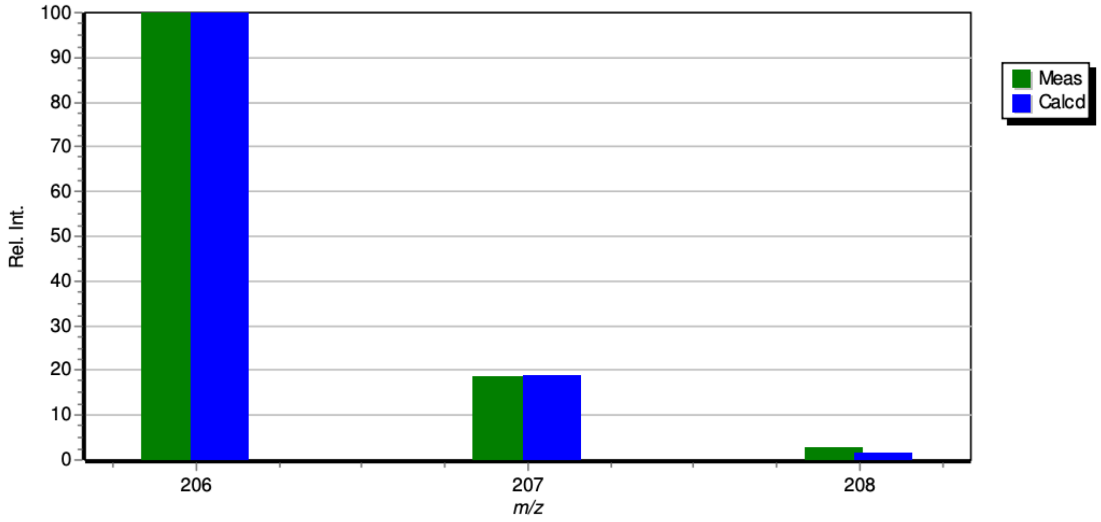

Deuterium: 0-fold (%): 100,00 97,84
Deuterium: 1-fold (%): 2,20 2,16
Label Atom Sum: 0,02 (0,18%)

Isotope List used for fitting data:

m/z	intensity
205,95	32485
206,01	63867
206,09	434648774
206,12	26922
206,17	81823
206,90	12190
206,93	367
206,99	22198
207,10	81365288
208,07	11169
208,11	12611268

2-phenylquinoline-3-D (1d-3-D):

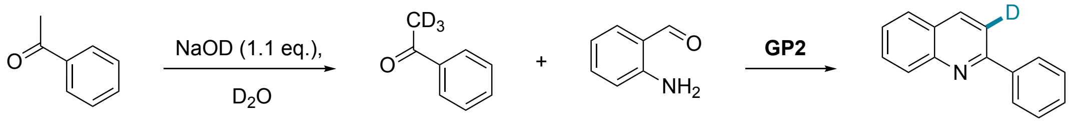

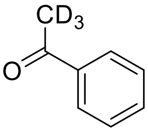

The synthesis of *α,α,α*-Trideuterioacetophenone was performed according in accordance to an adjusted literature procedure.[39] A mixture of acetophenone (5.0 mmol, 0.58 mL, 1.0 eq.), NaOD (40% D_2O solution, 0.56 mL, 1.1 eq.), and D_2O (2.5 mL) was stirred for 24 h at room temperature under argon atmosphere. The mixture was extracted with Et_2O. The organic layer was dried over $MgSO_4$ and filtered. The solvents were removed at the rotary evaporator under reduced pressure. The crude product was used directly in the next reaction step.

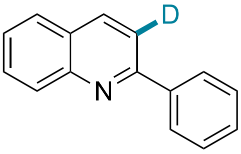

The reaction was performed according to **GP2** with 2-aminobenzaldehyde (0.24 g, 2.0 mmol, 1.0 eq.) and *α,α,α*-Trideuterioacetophenone (0.23 mL, 2.0 mmol, 1.0 eq.) in EtOD. After purification via Flash-Chromatography (P/EtOAc – 20/1), the product **1d-3-D** was obtained as a white solid (0.22 mg, 1.1 mmol, 53%).

^{1}H-NMR (400 MHz, $CDCl_3$): δ (ppm) = 8.25 – 8.21 (m, 1H), 8.20 – 8.14 (m, 3H), 7.89 (d, *J* = 8.6 Hz, 0.1H), 7.84 (dd, *J* = 8.1, 1.5 Hz, 1H), 7.73 (ddd, *J* = 8.4, 6.8, 1.5 Hz, 1H), 7.57 – 7.50 (m, 3H), 7.50 – 7.44 (m, 1H).

HRMS (ESI): m/z calculated for $[M+H]^+$ $C_{15}H_{11}DN^+$ 207.1027, found 207.1027.

The molecule has a 90% D-incorporation at 3-position of the quinoline. The analytical data match those reported in the literature.[40]

^{1}H-NMR (400 MHz, $CDCl_3$)

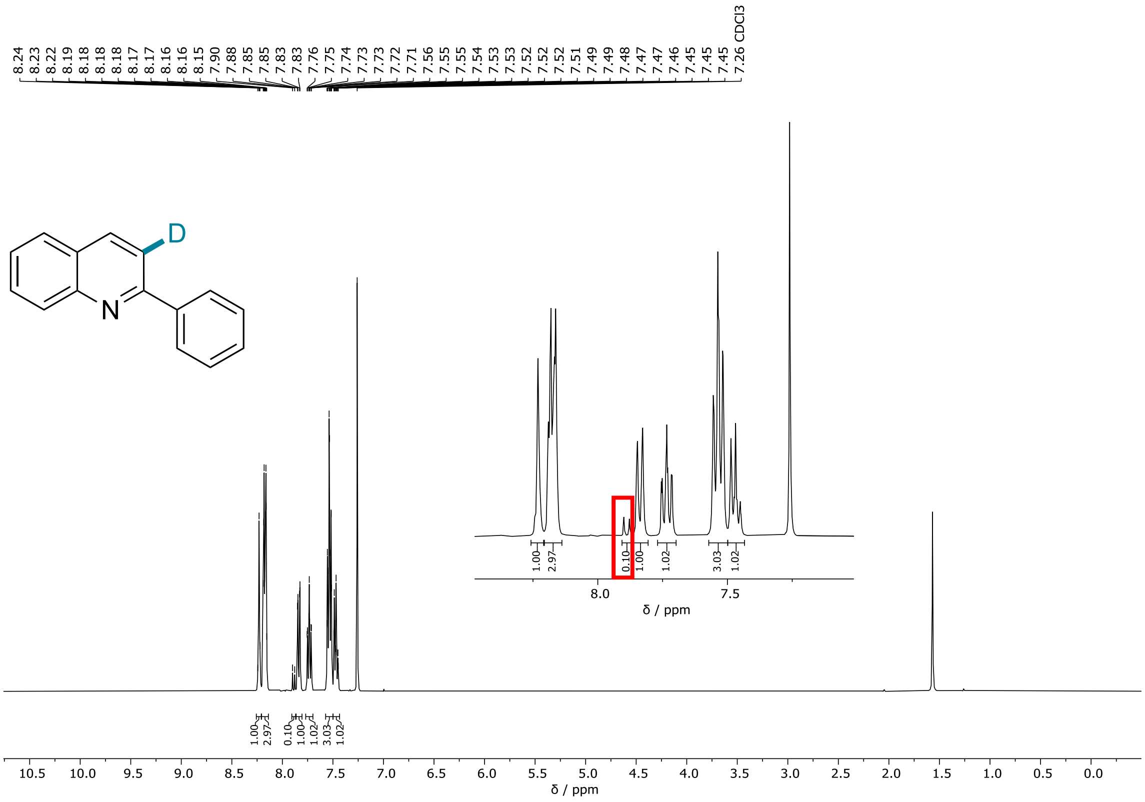

LabelChecker Results

Formula: C15 H11 N Na
Mass (monoisotopic): 228,08

Difference Value: 0,000069
Error Sum: 0,008
Error (%): 0,167

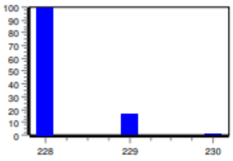

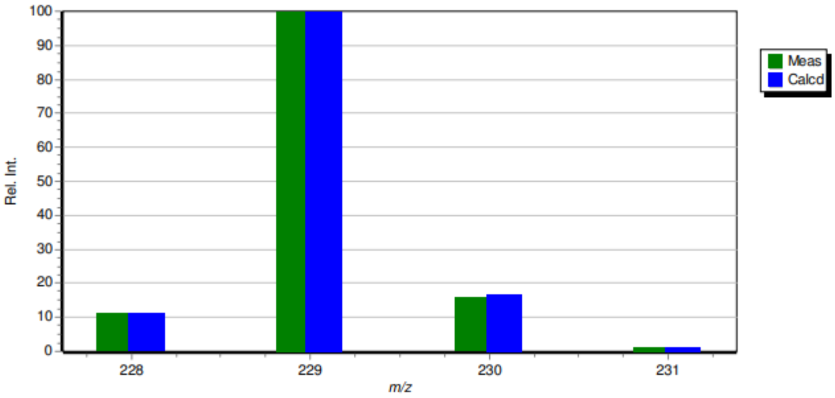

Deuterium: 0-fold (%): 11,34 10,18
Deuterium: 1-fold (%): 100,00 89,78
Deuterium: 2-fold (%): 0,05 0,05
Deuterium: 3-fold (%): 0,00 0,00
Deuterium: 4-fold (%): 0,00 0,00
Label Atom Sum: 0,90 (8,17%)

Isotope list used for fitting data:

m/z	intensity
227,99	117351
228,08	226725328
228,61	58096
228,90	58775
228,97	142851 2
229,07	2041373028
229,12	7456442
229,15	1537727
229,19	53762
230,00	104767
230,09	324660320
230,14	693320
230,97	60125
231,09	25293238
231,12	52846

2-phenylquinoline-4-D (1d-4-D):

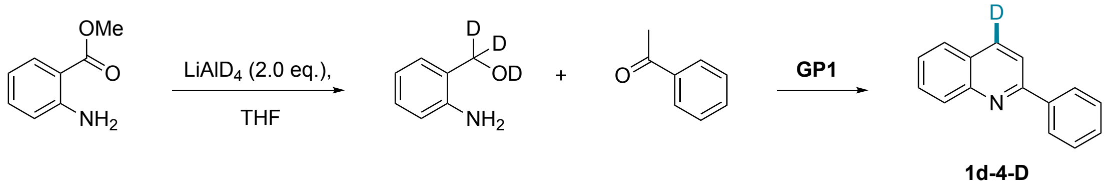

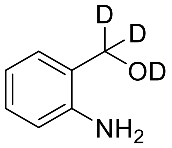

The synthesis of (2-aminophenyl)methan-D_2-ol-D was performed according in accordance to an adjusted literature procedure.[41] Methyl 2-aminobenzoate (0.65 mL, 5.0 mmol, 1.0 eq.) was dissolved in dry THF (12 mL, 0.3 M). The reaction mixture was cooled down to 0 °C and $LiAlD_4$ (0.42 g, 10 mmol, 2.0 eq.) was added at this temperature partially. The mixture was warmed up to r.t. and stirred for further 2h. The reaction mixture was quenched by a Fieser Work-Up. The solvents were removed at the rotary evaporator under reduced pressure. The crude product was used directly in the next reaction step.

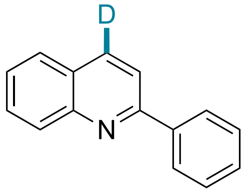

The reaction was performed according to **GP1** with (2-aminophenyl)methan-D_2-ol-D (0.25 g, 2.0 mmol, 1.0 eq.) and acetophenone (0.24 mL, 2.0 mmol, 1.0 eq.). After purification via Flash-Chromatography (P/EtOAc –20/1), the product **1d-4-D** was obtained as an off-white solid (0.18 g, 0.89 mmol, 45%).

^{1}H-NMR (400 MHz, $CDCl_3$): δ (ppm) = 8.21 – 8.13 (m, 3H), 7.89 (s, 1H), 7.84 (dd, J = 8.1, 1.5 Hz, 1H), 7.73 (ddd, J = 8.5, 6.8, 1.5 Hz, 1H), 7.58 – 7.49 (m, 3H), 7.51 – 7.43 (m, 1H).

HRMS (ESI): m/z calculated for $[M+H]^+$ $C_{15}H_{11}DN^+$ 207.1027, found 207.1027.

The molecule has a 100% D-incorporation at 4-position of the quinoline. The analytical data match those reported in the literature.[41]

^{1}H-NMR (400 MHz, $CDCl_3$)

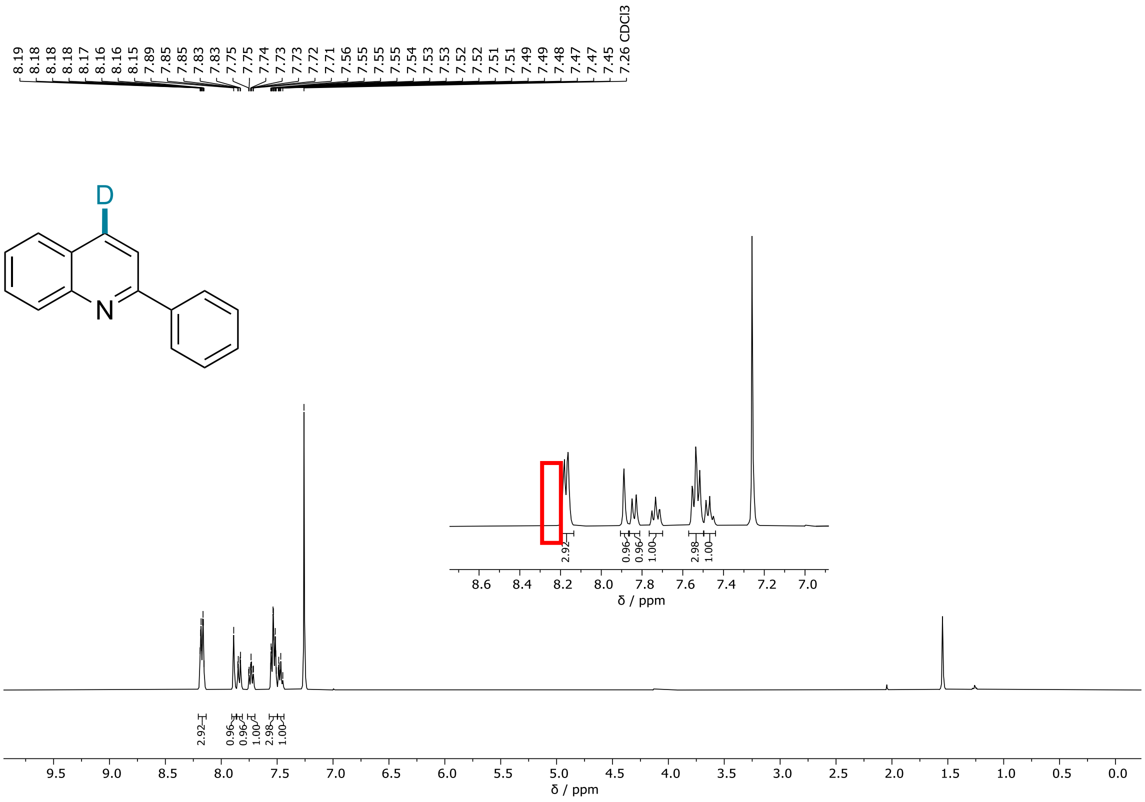

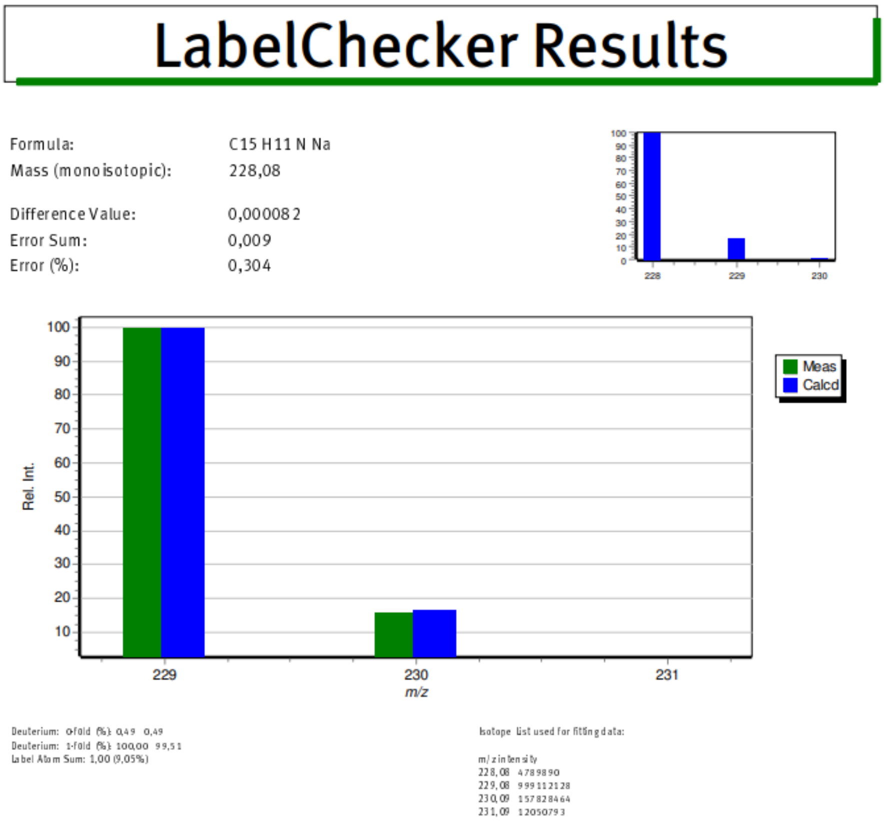

7. NMR Spectra

7.1. NMR Spectra of Starting Material

2-phenyl-3-propan-2-ylquinoline (1a):

^{1}H-NMR (300 MHz, $CDCl_3$)

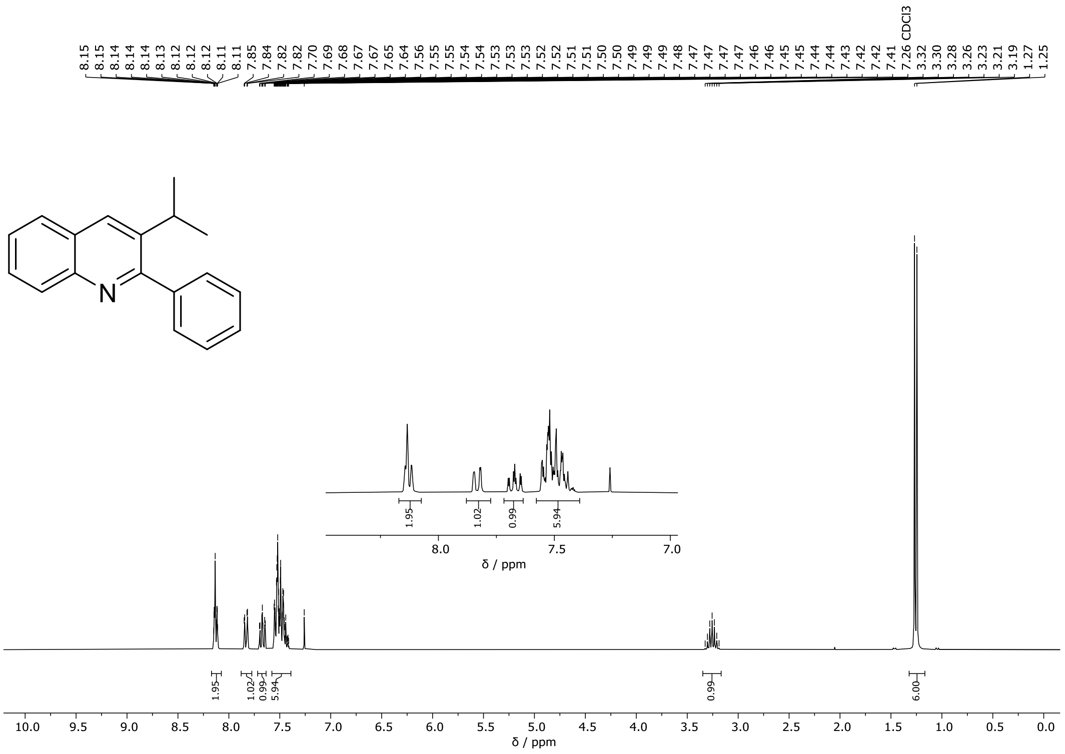

^{13}C-NMR (76 MHz, $CDCl_3$)

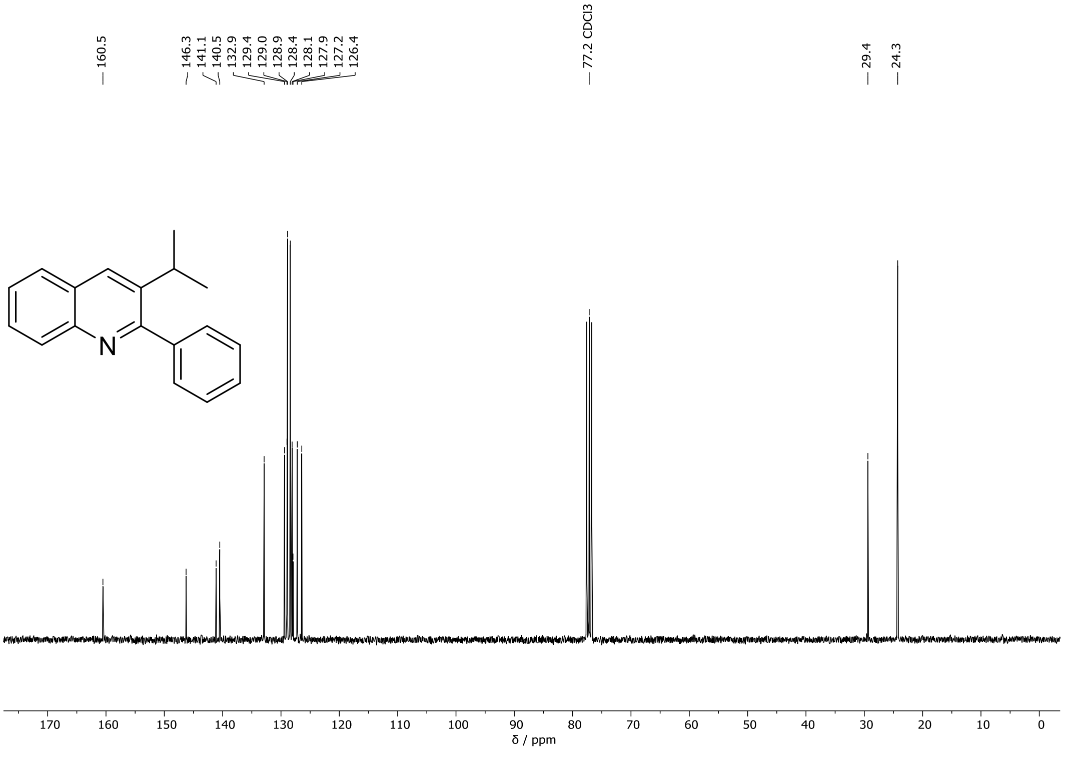

3-hexyl-2-phenylquinoline (1b):

^{1}H-NMR (300 MHz, $CDCl_3$)

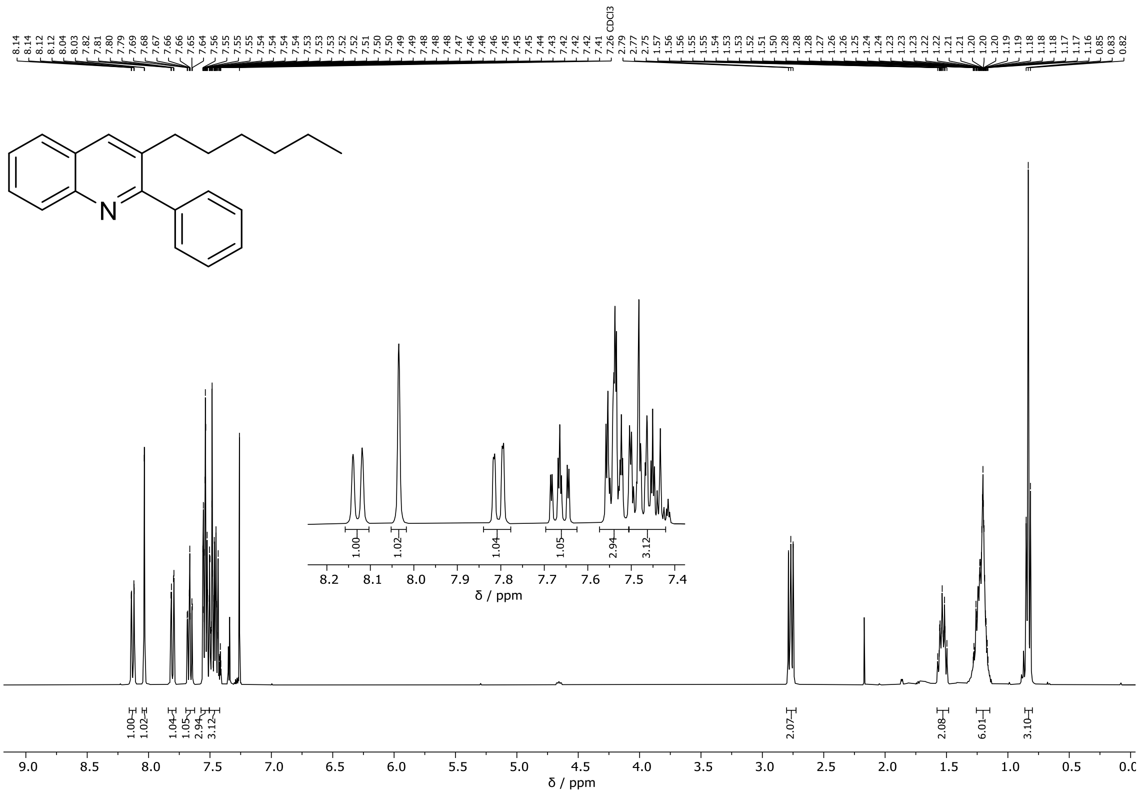

^{13}C-NMR (76 MHz, $CDCl_3$)

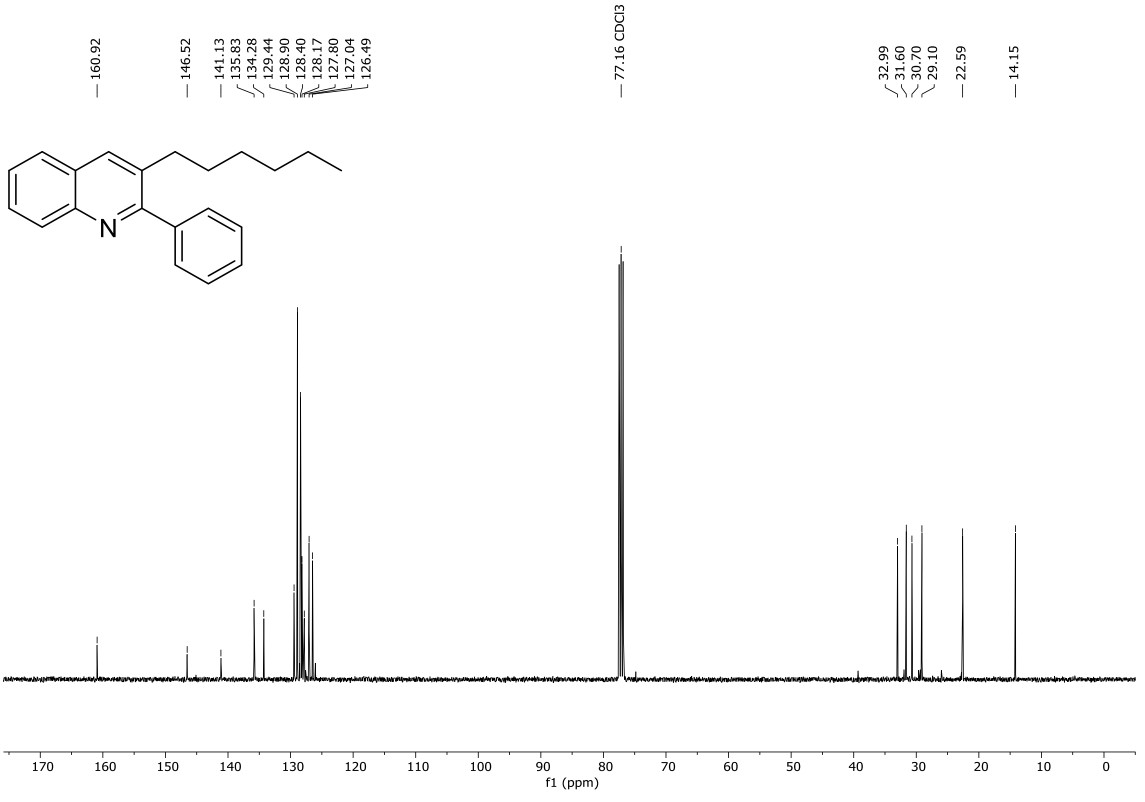

3-ethyl-2-phenylquinoline (1c):

^{1}H-NMR (300 MHz, $CDCl_3$)

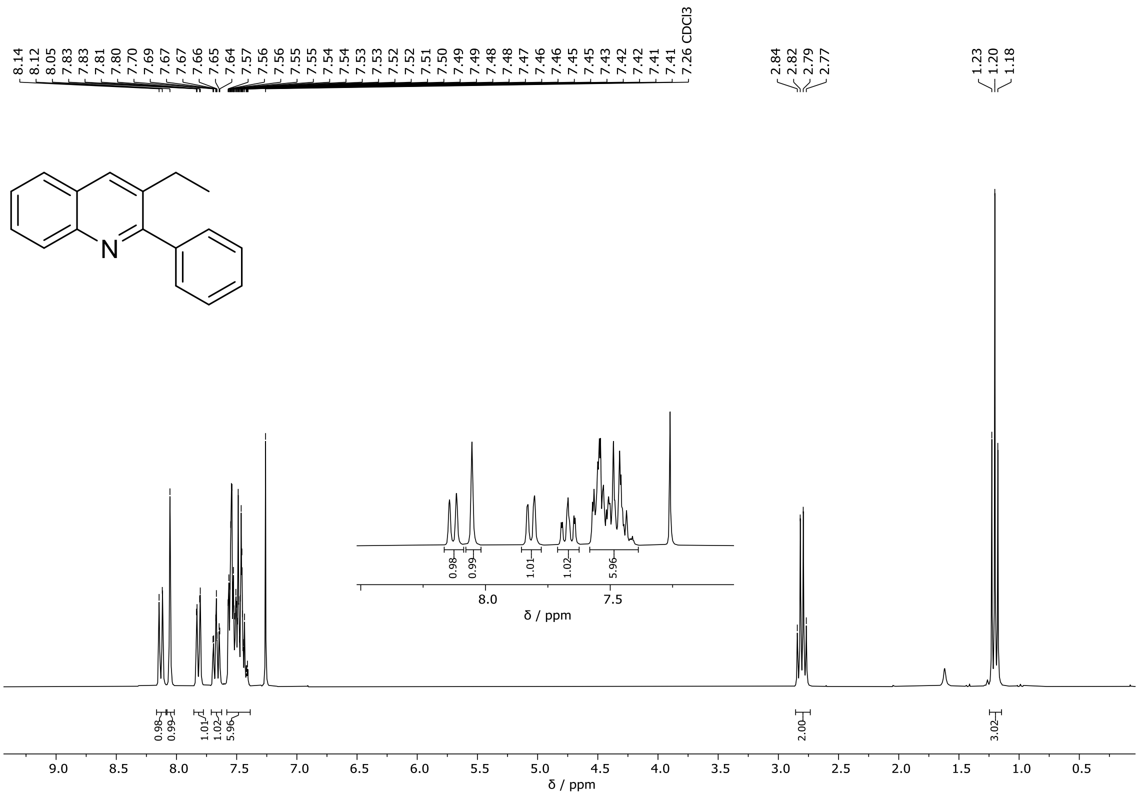

^{13}C-NMR (76 MHz, $CDCl_3$)

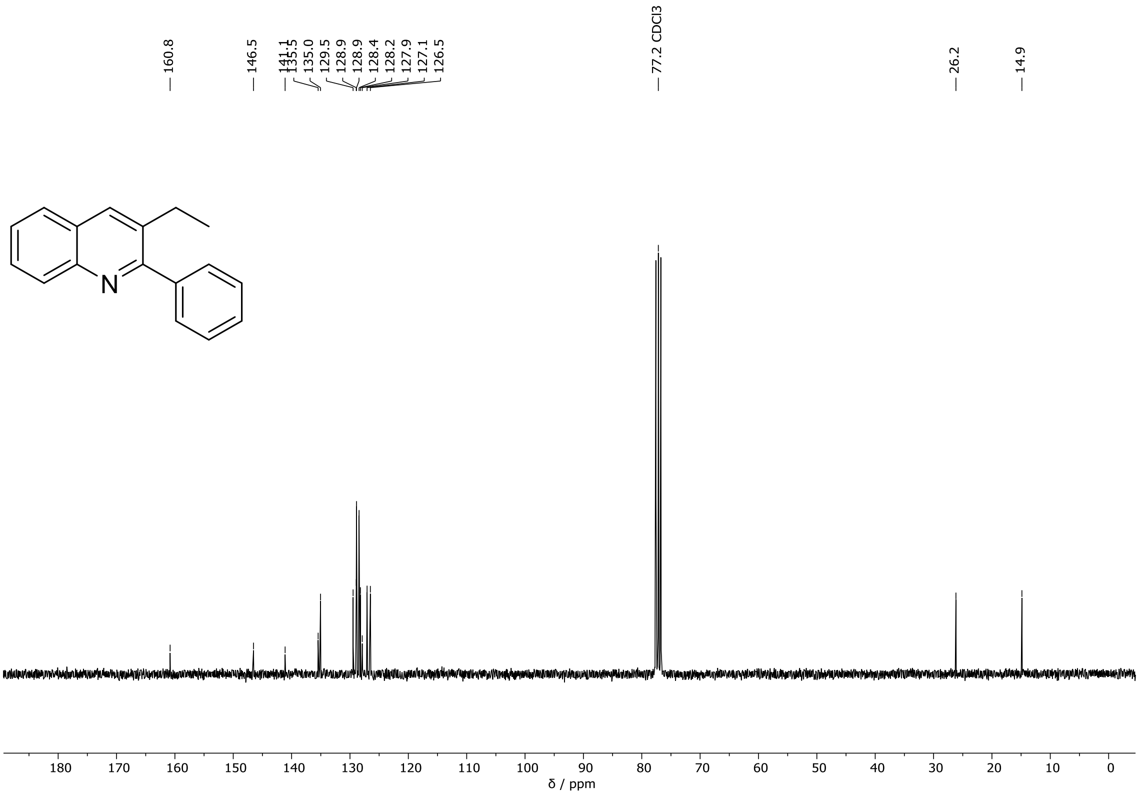

2-phenylquinoline (1d):

^{1}H-NMR (400 MHz, $CDCl_3$)

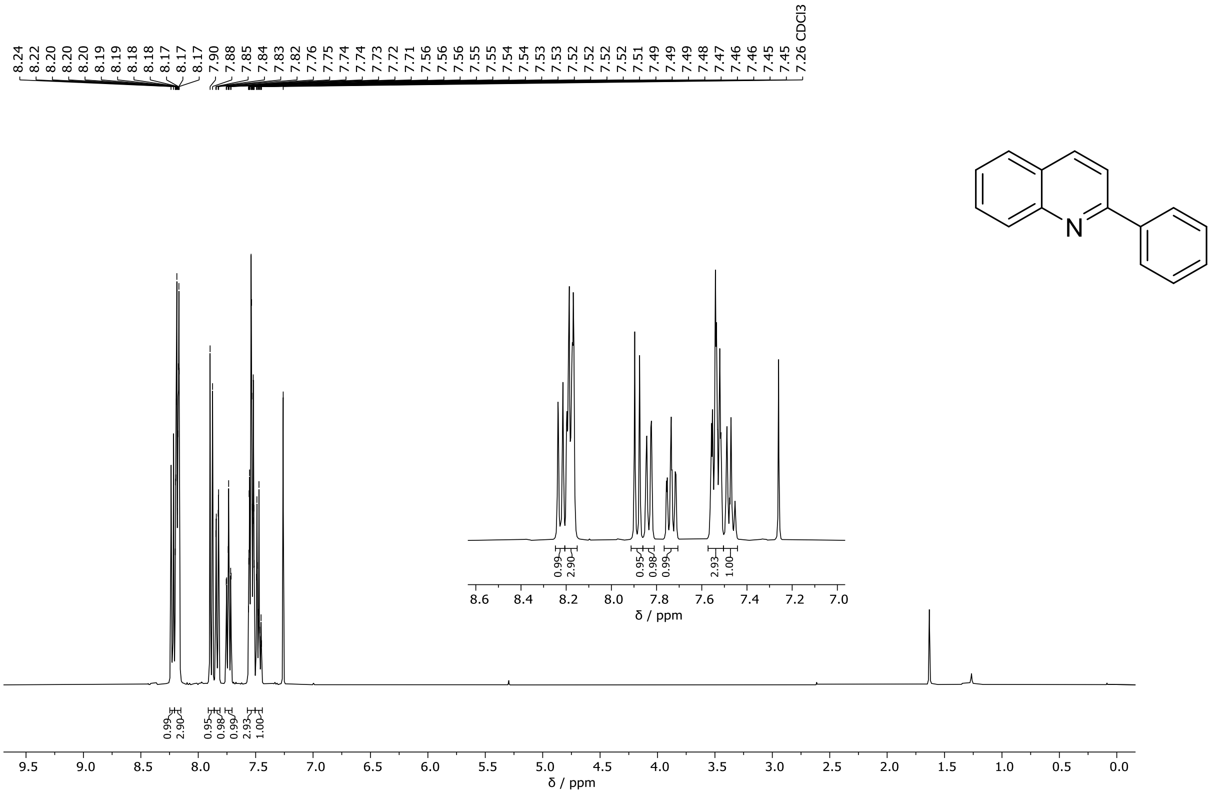

^{13}C-NMR (101 MHz, $CDCl_3$)

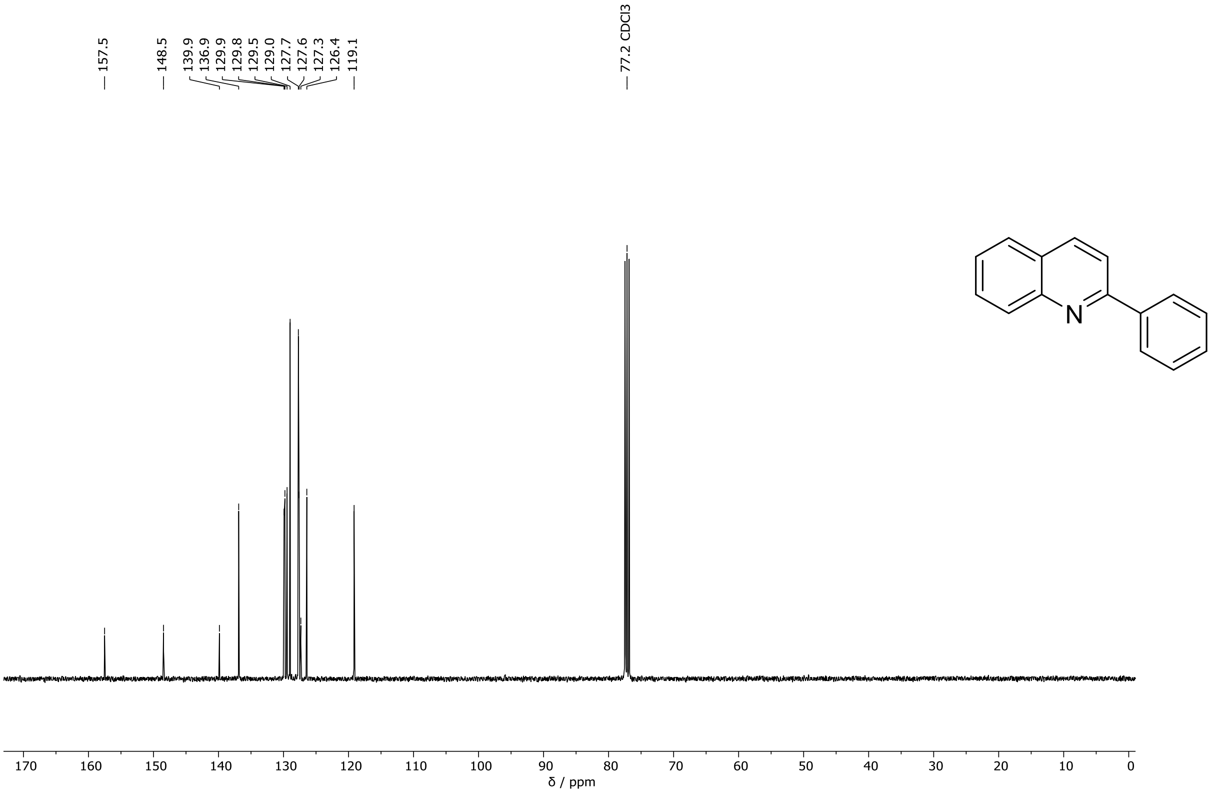

3-isopropyl-2-(4-(trifluoromethyl)phenyl)quinoline (1e):

^{1}H-NMR (500 MHz, $CDCl_3$)

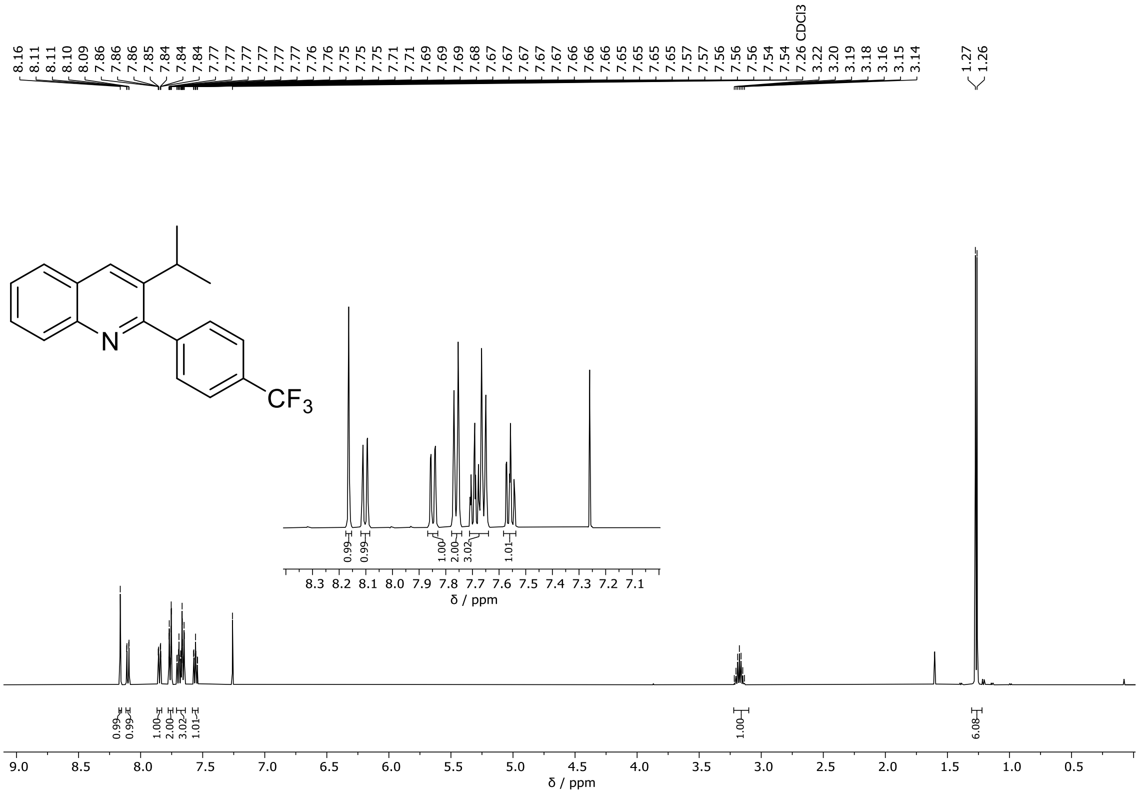

^{13}C{^{19}F}-NMR (126 MHz, $CDCl_3$)

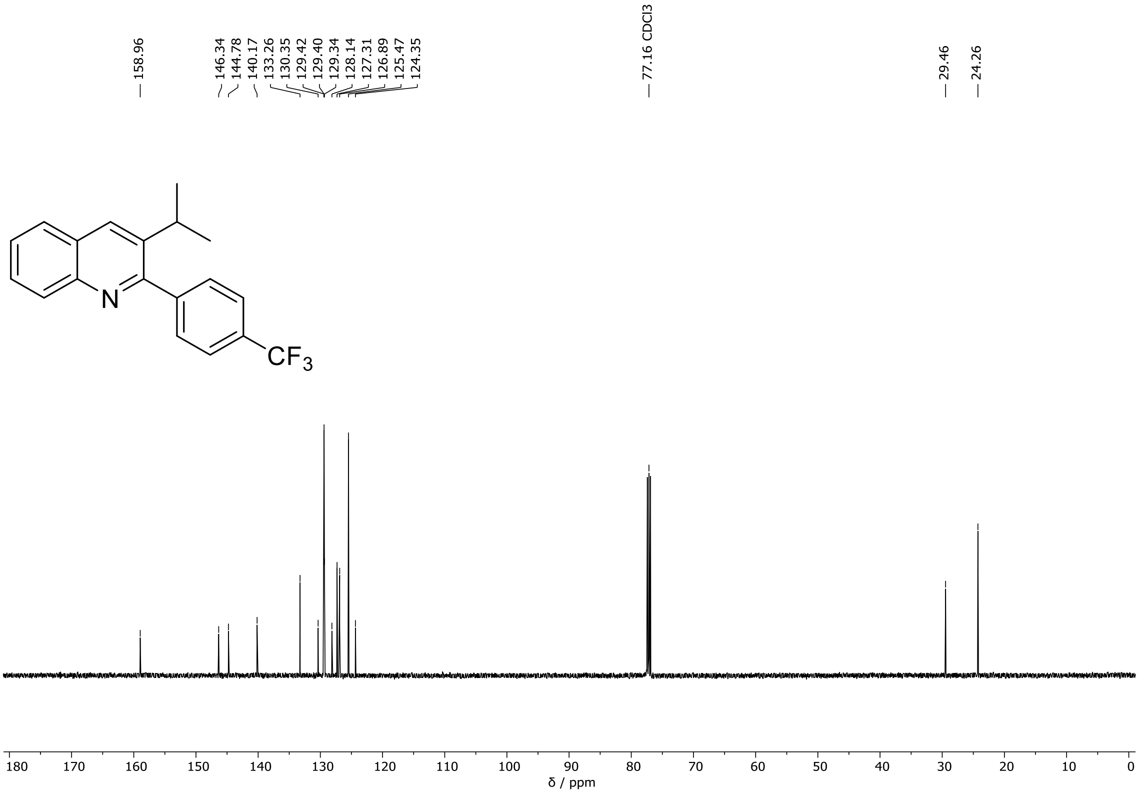

$^{19}F\{^{1}H\}$-NMR (470 MHz, $CDCl_3$)

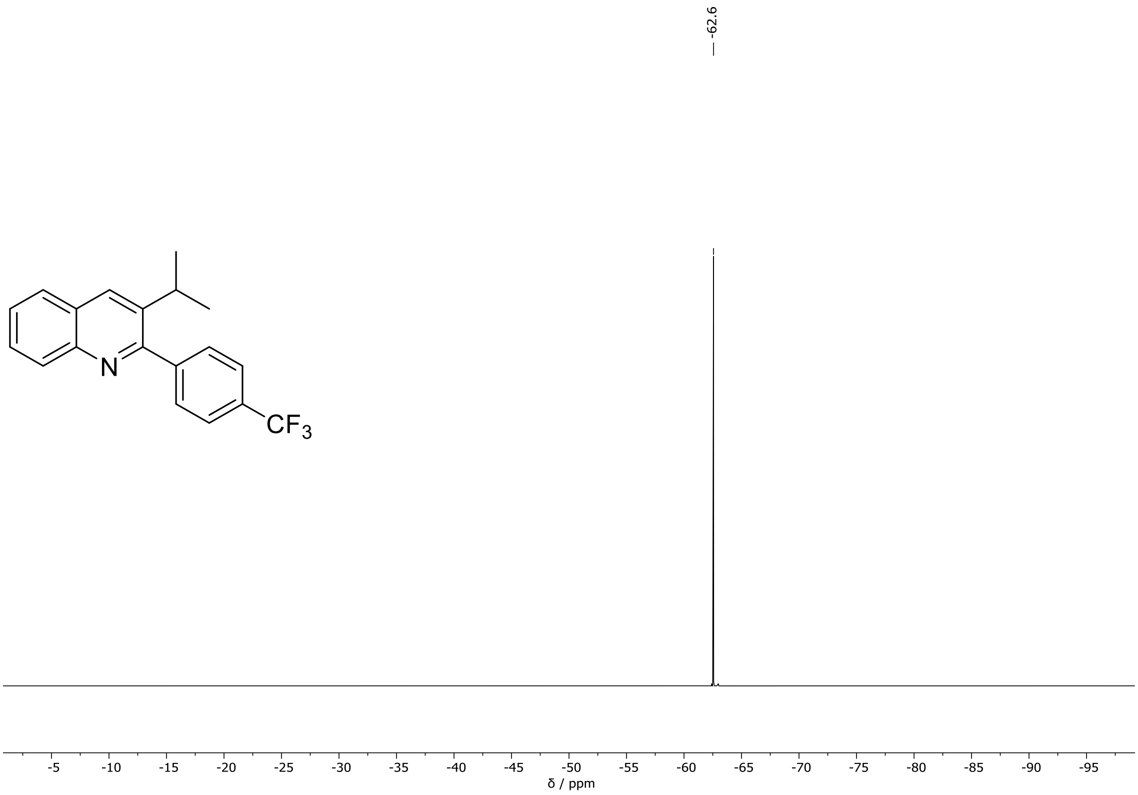

2-(4-(trifluoromethyl)phenyl)quinoline (1f):

^{1}H-NMR (500 MHz, $CDCl_3$)

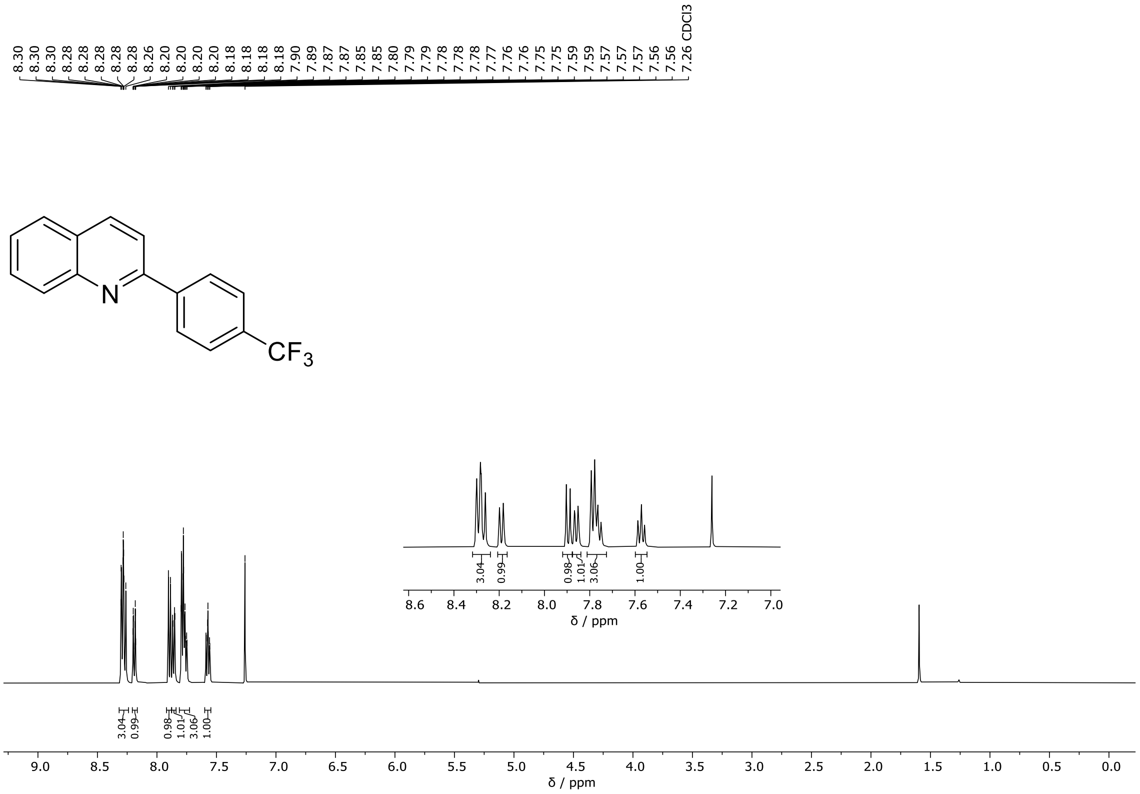

^{13}C{^{19}F}-NMR (126 MHz, $CDCl_3$)

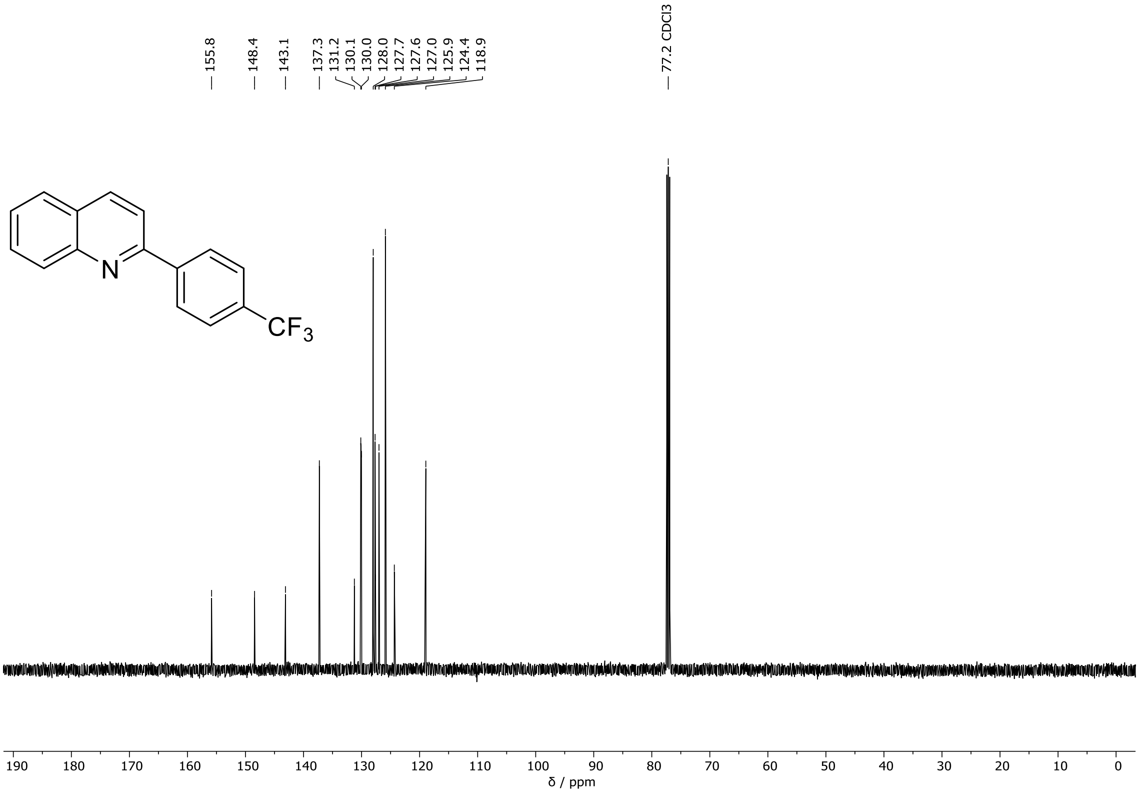

$^{19}F\{^{1}H\}$-NMR (126 MHz, $CDCl_3$)

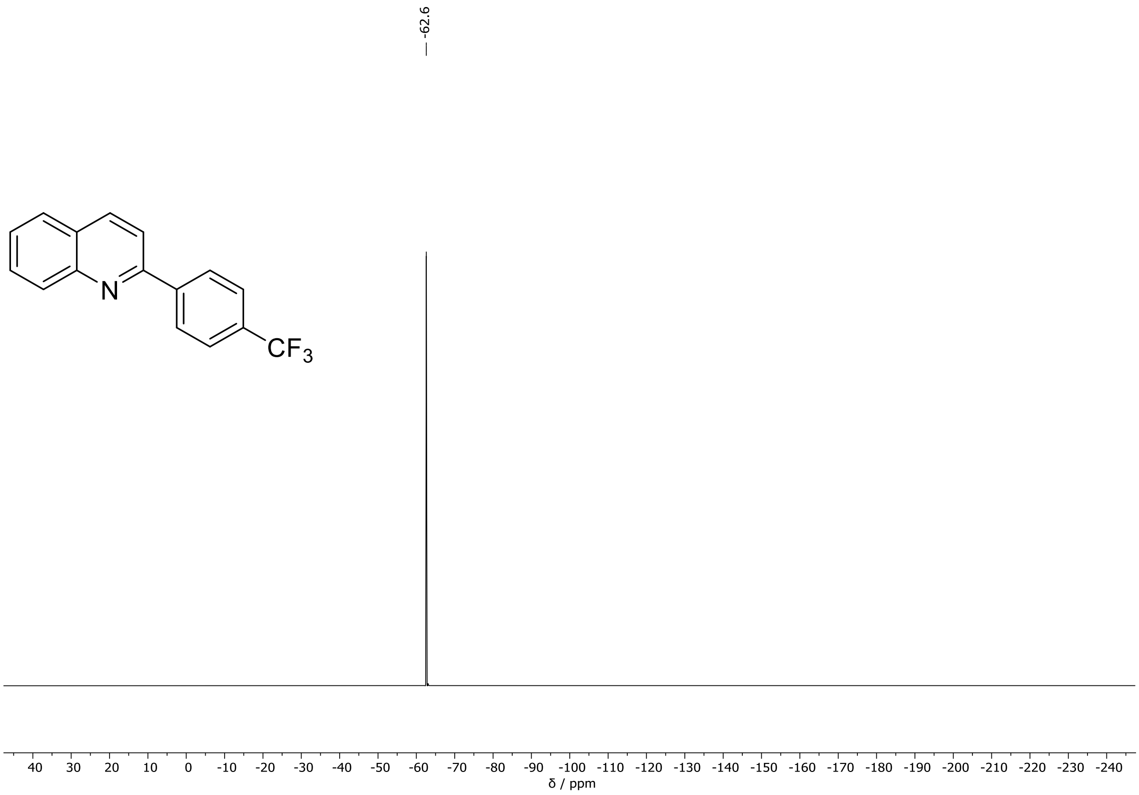

3-isopropyl-2-(4-methoxyphenyl)quinoline (1g):

^{1}H-NMR (599 MHz, $CDCl_3$)

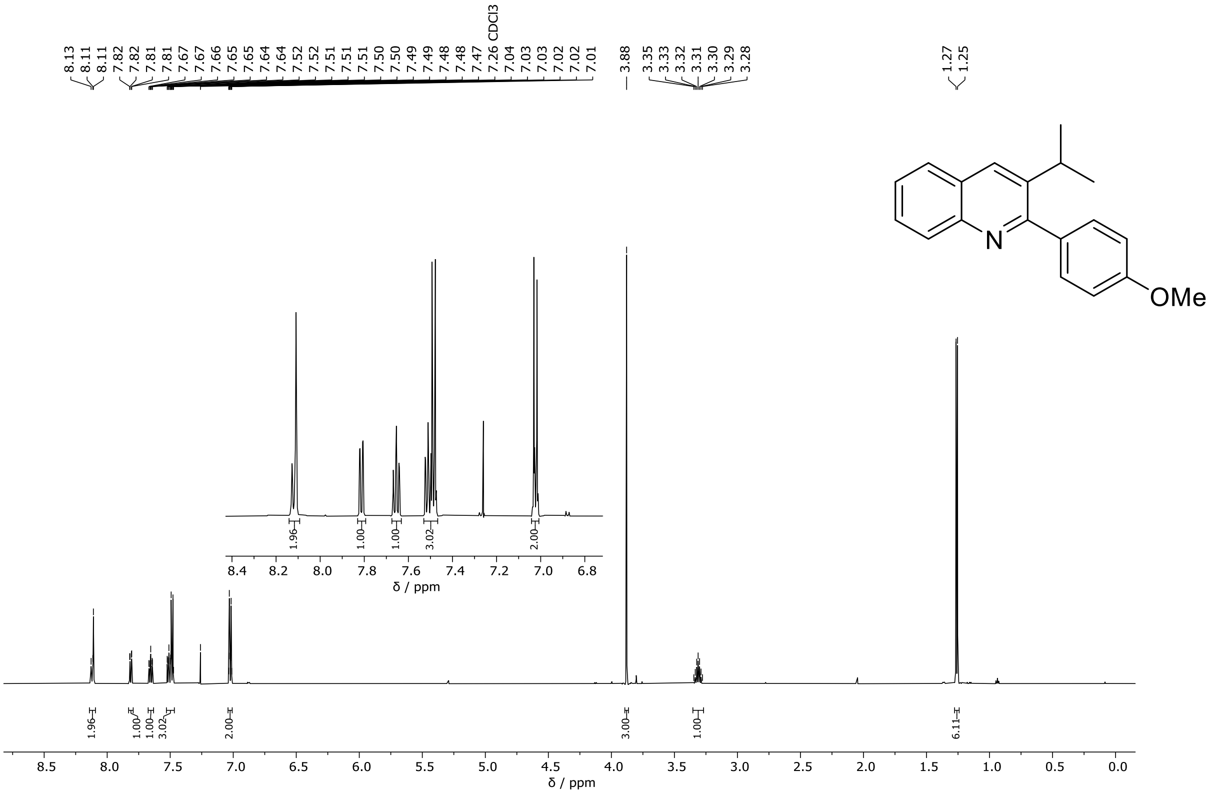

^{13}C-NMR (151 MHz, $CDCl_3$)

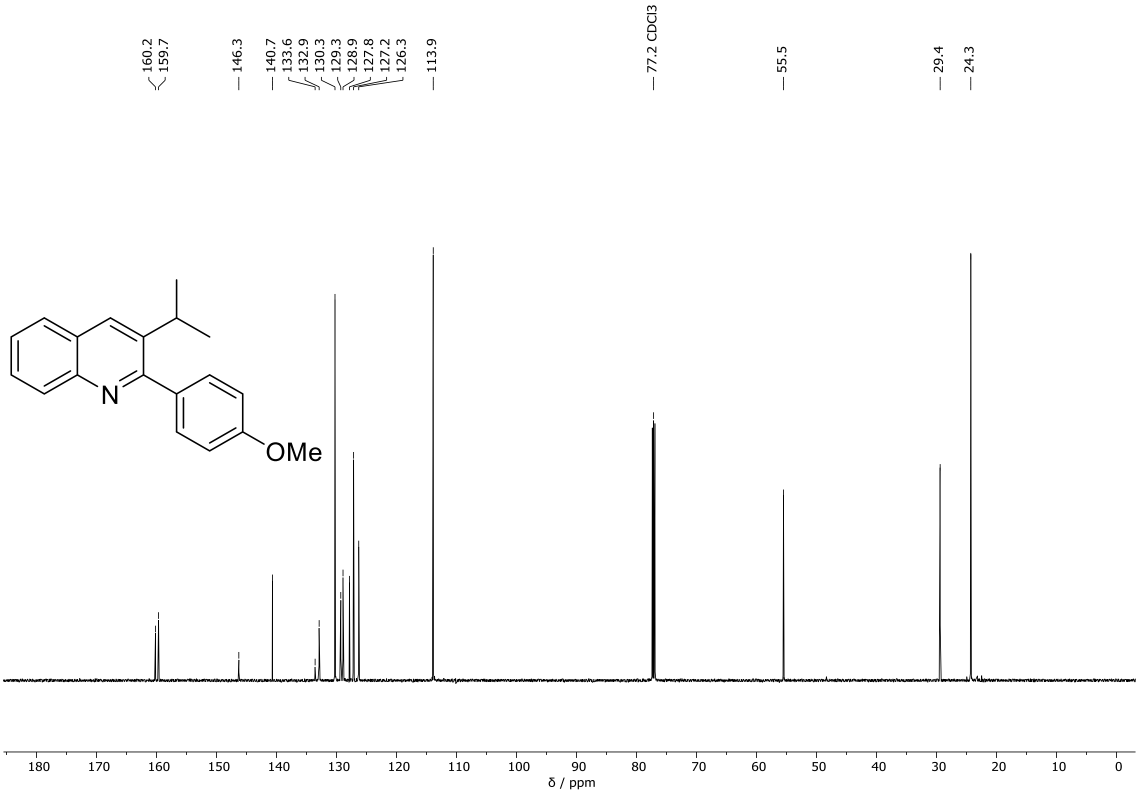

2-(4-methoxyphenyl)quinoline (1h):

^{1}H-NMR (400 MHz, $CDCl_3$)

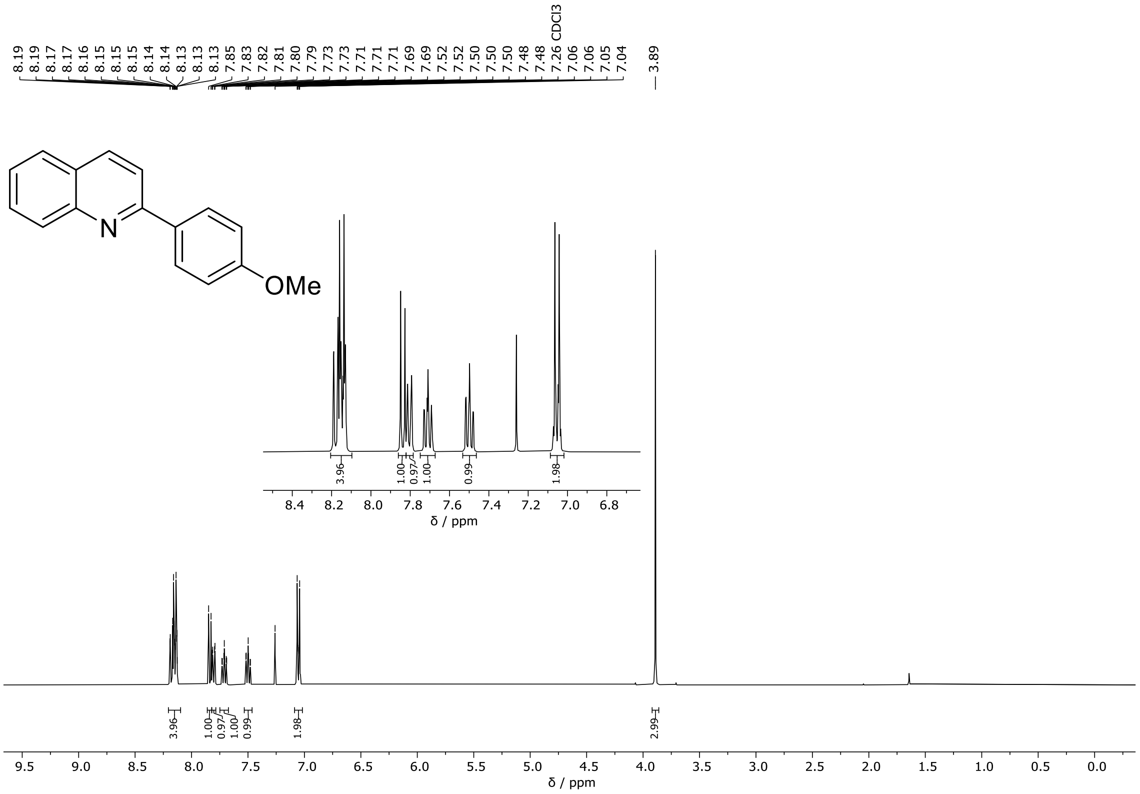

^{13}C-NMR (101 MHz, $CDCl_3$)

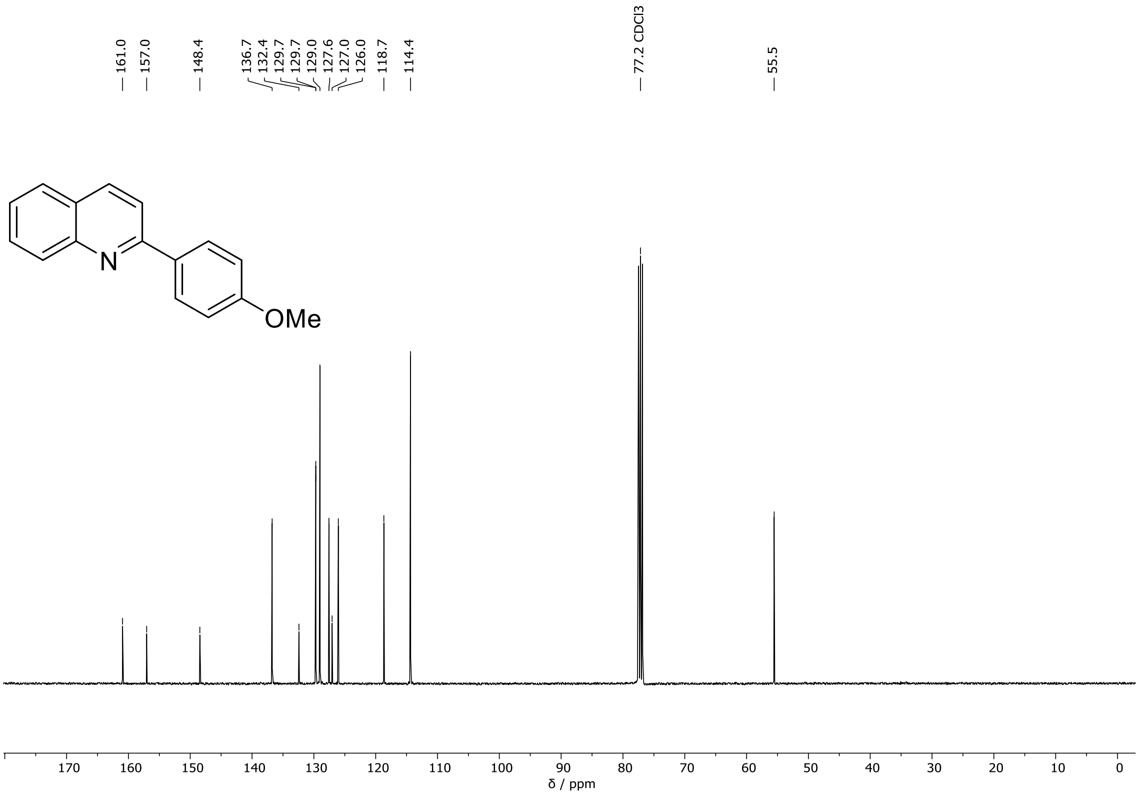

2-(*p*-tolyl)quinoline (1i):

^{1}H-NMR (400 MHz, $CDCl_3$)

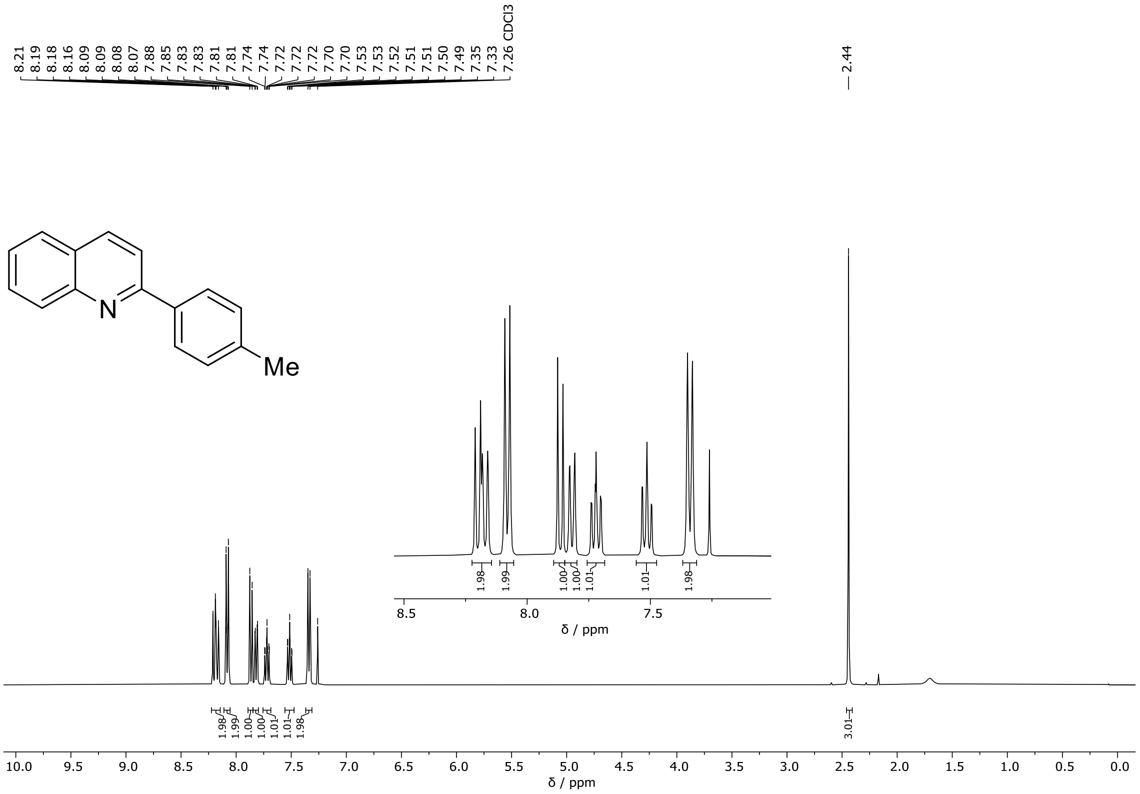

^{13}C-NMR (101 MHz, $CDCl_3$)

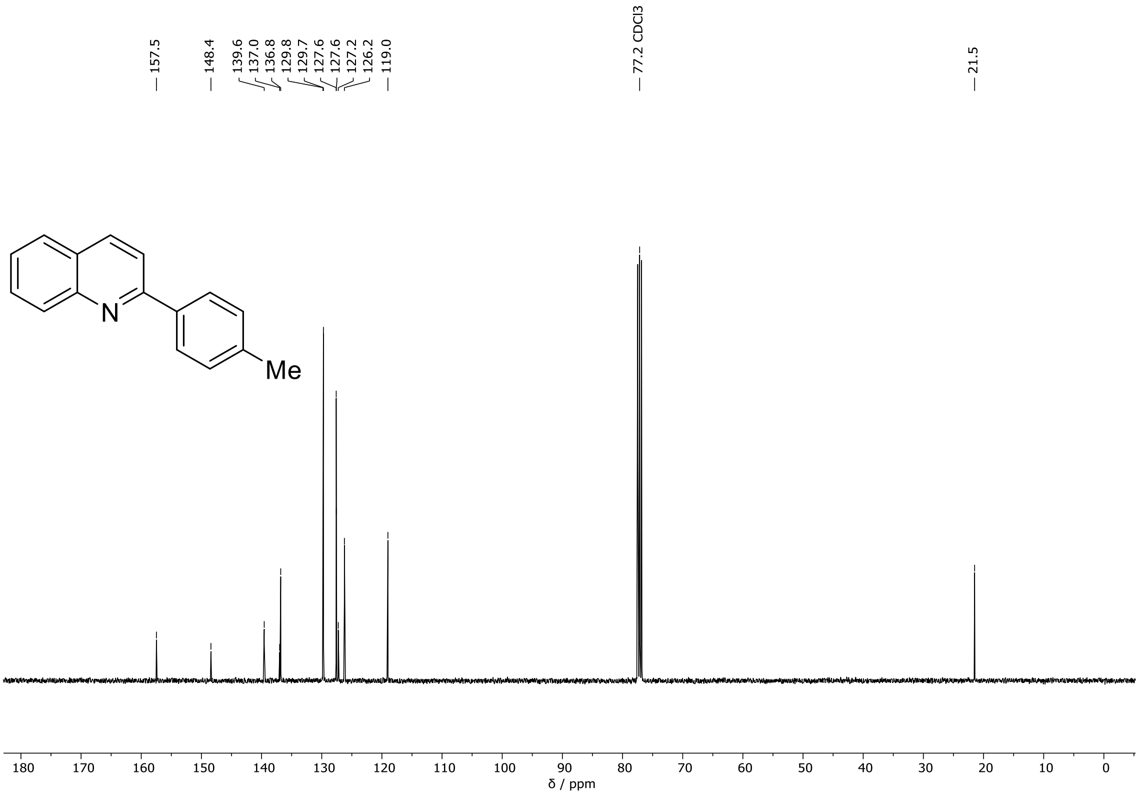

2-(4-fluorophenyl)quinoline (1j):

^{1}H-NMR (300 MHz, $CDCl_3$)

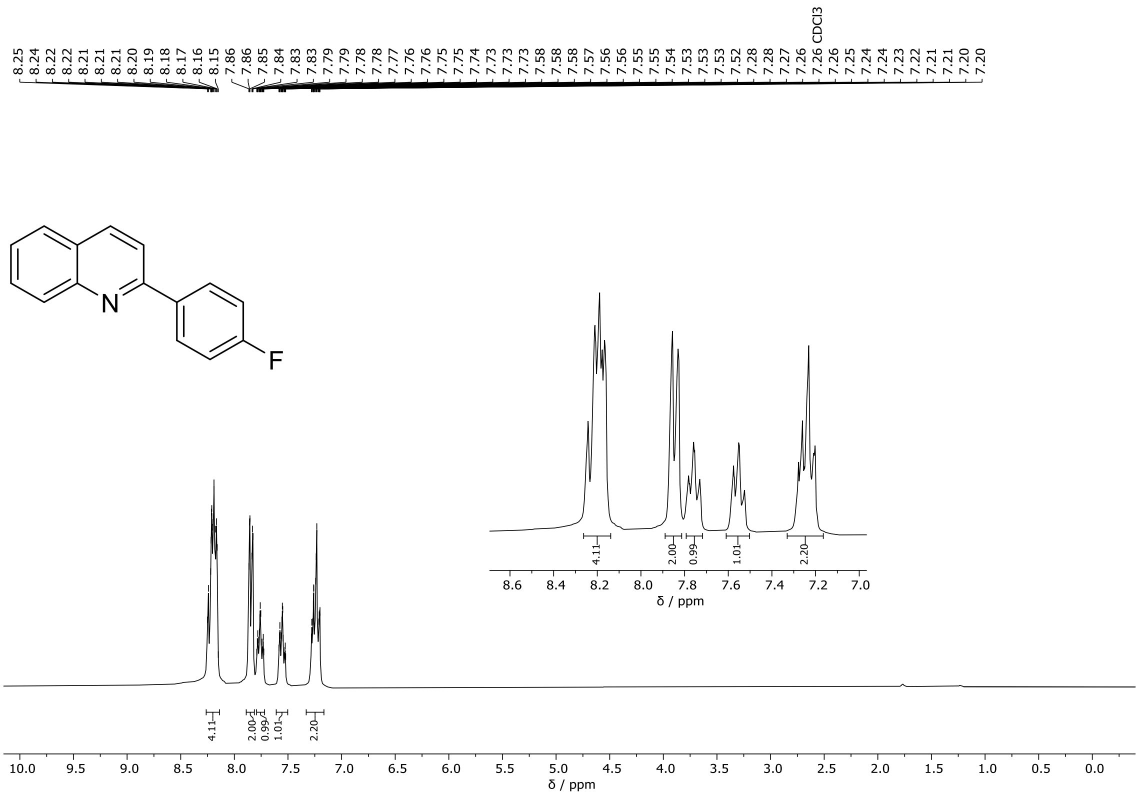

^{13}C-NMR (76 MHz, $CDCl_3$)

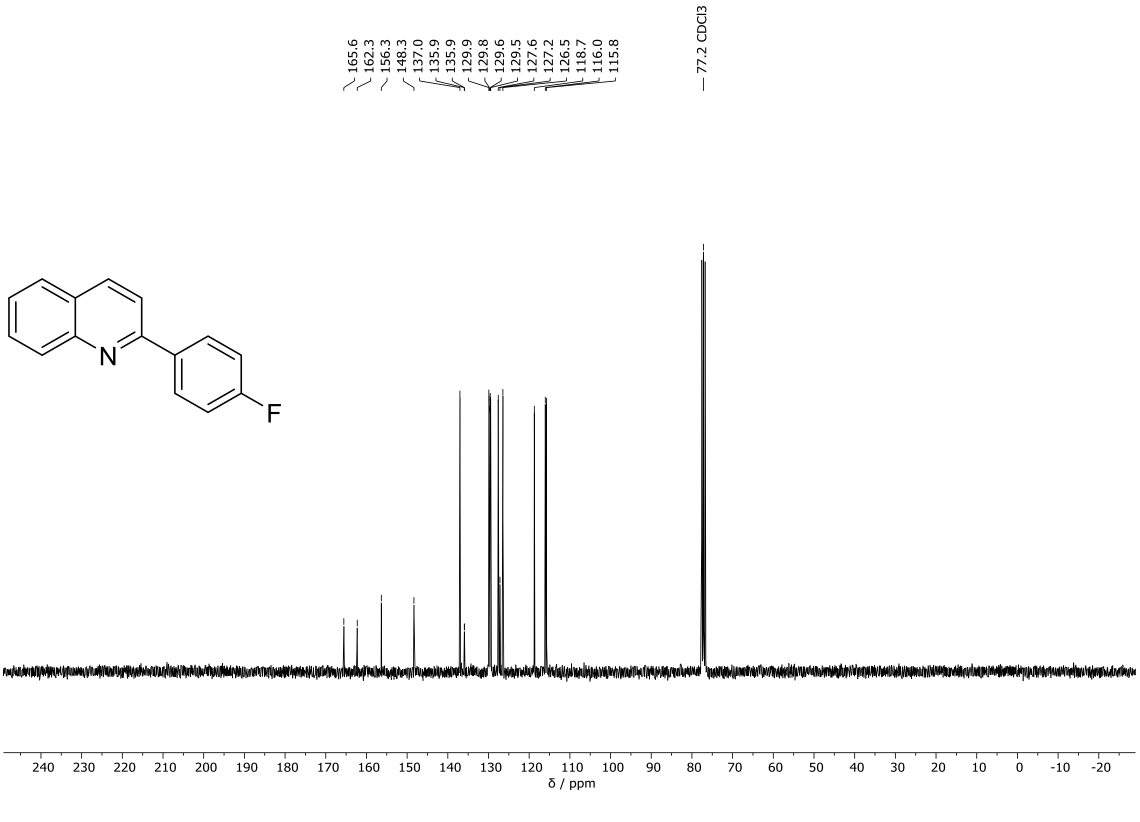

^{19}F-NMR (282 MHz, $CDCl_3$)

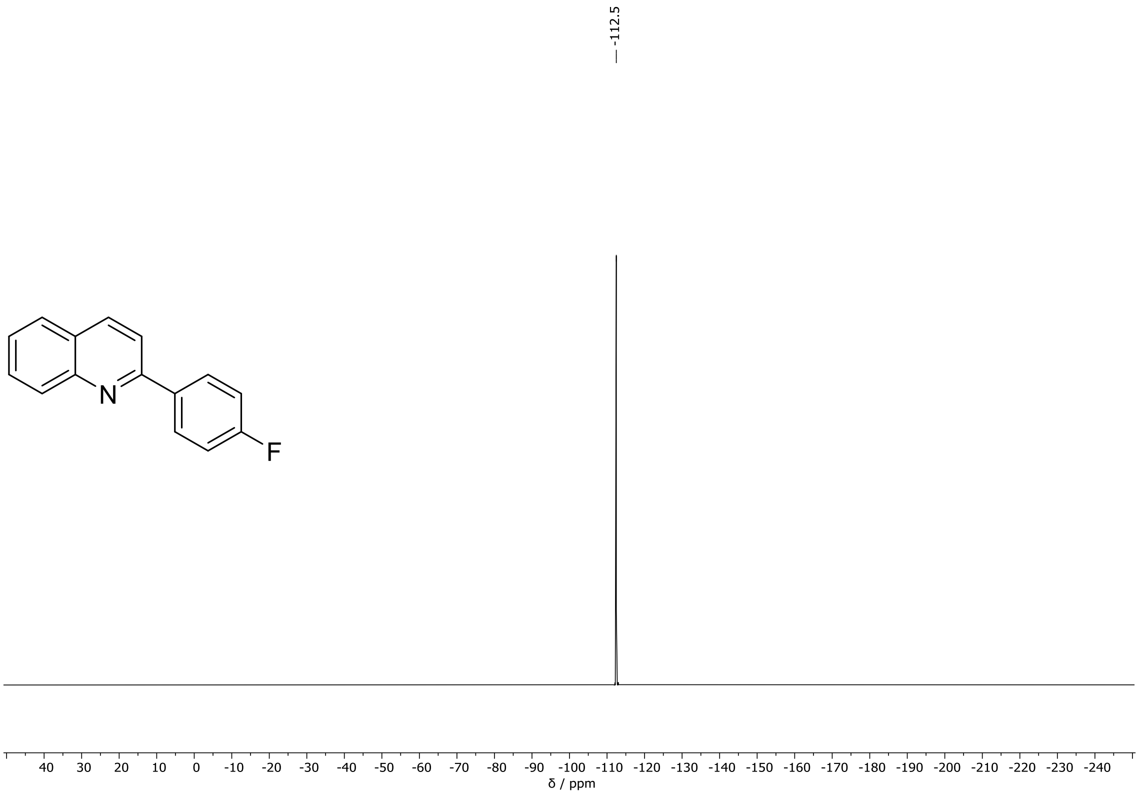

2-(3-(trimethylsilyl)phenyl)quinoline (1k):

^{1}H-NMR (300 MHz, $CDCl_3$)

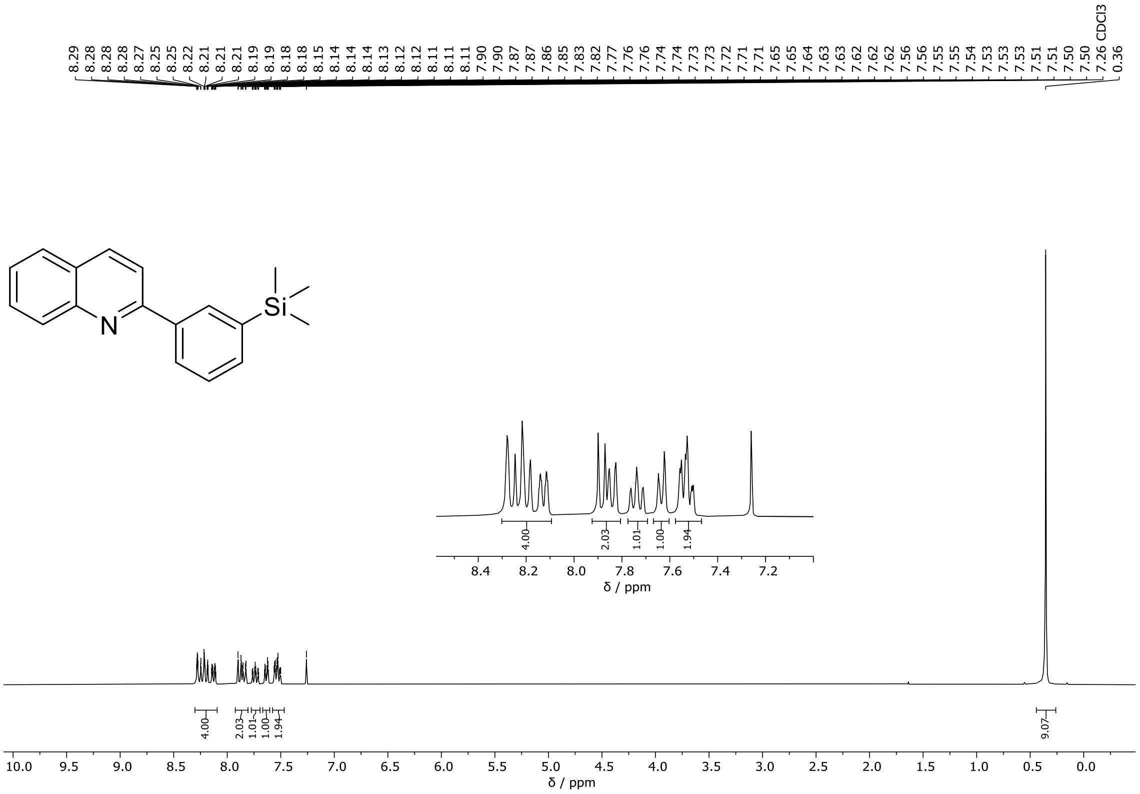

^{13}C-NMR (76 MHz, $CDCl_3$)

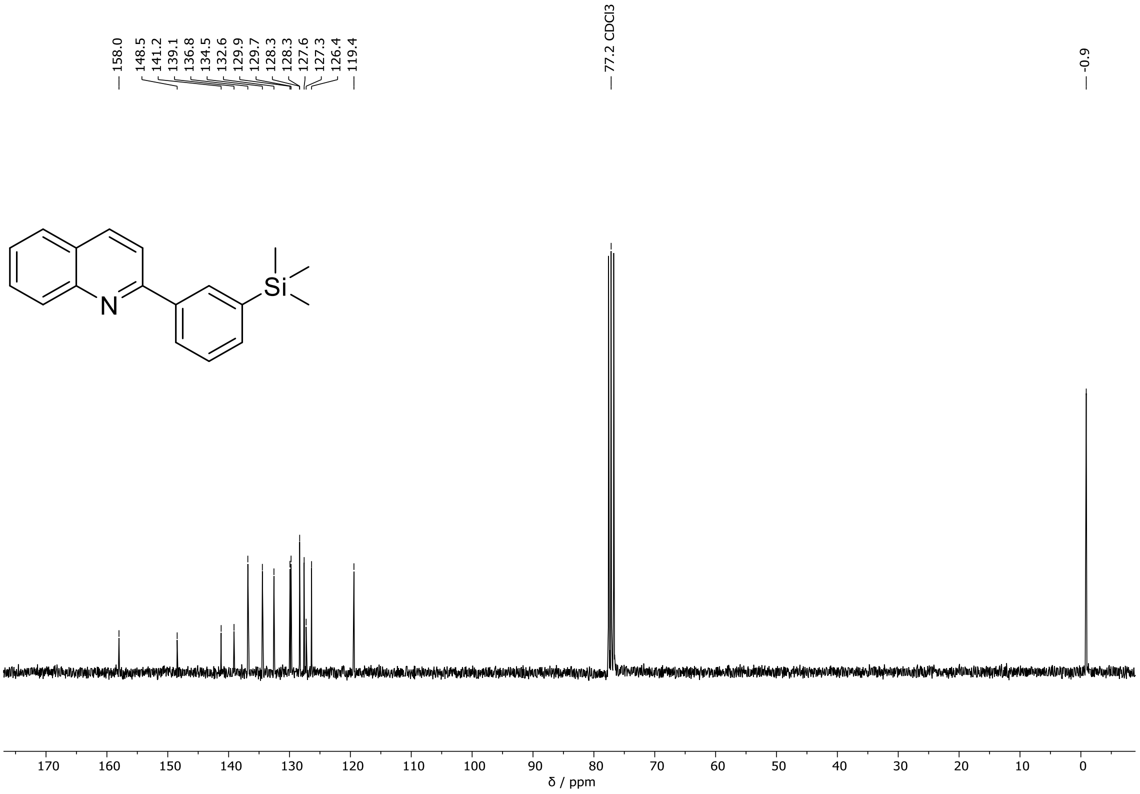

²⁹Si-NMR (80 MHz, CDCl$_3$)

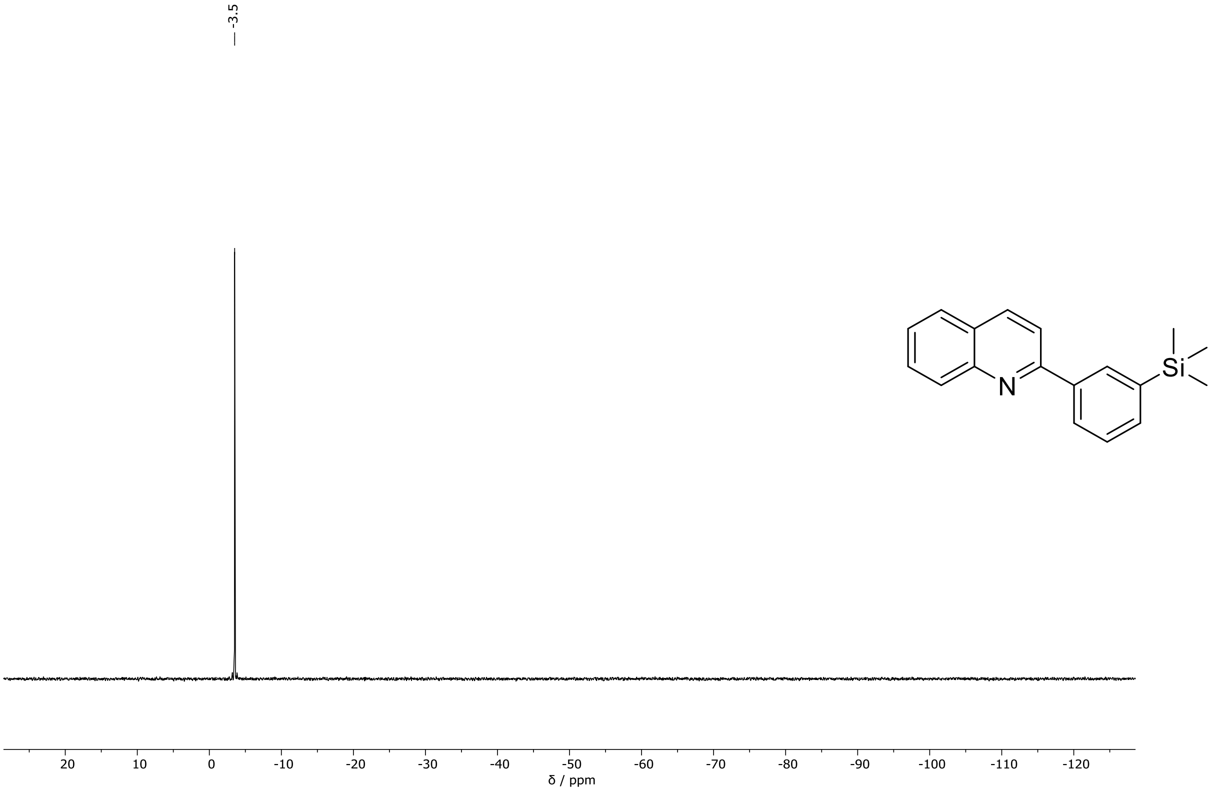

3-(3-methylquinolin-2-yl)phenol (1l):

^{1}H-NMR (599 MHz, $CDCl_3$)

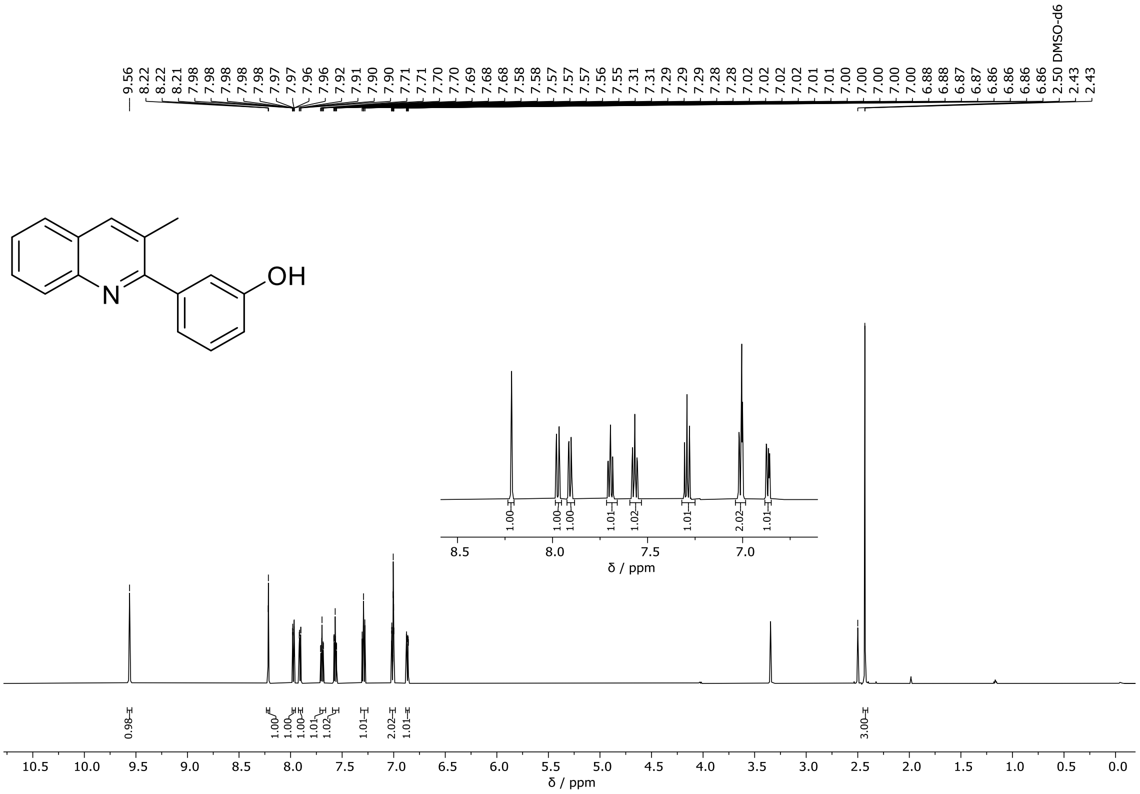

^{13}C-NMR (151 MHz, $CDCl_3$)

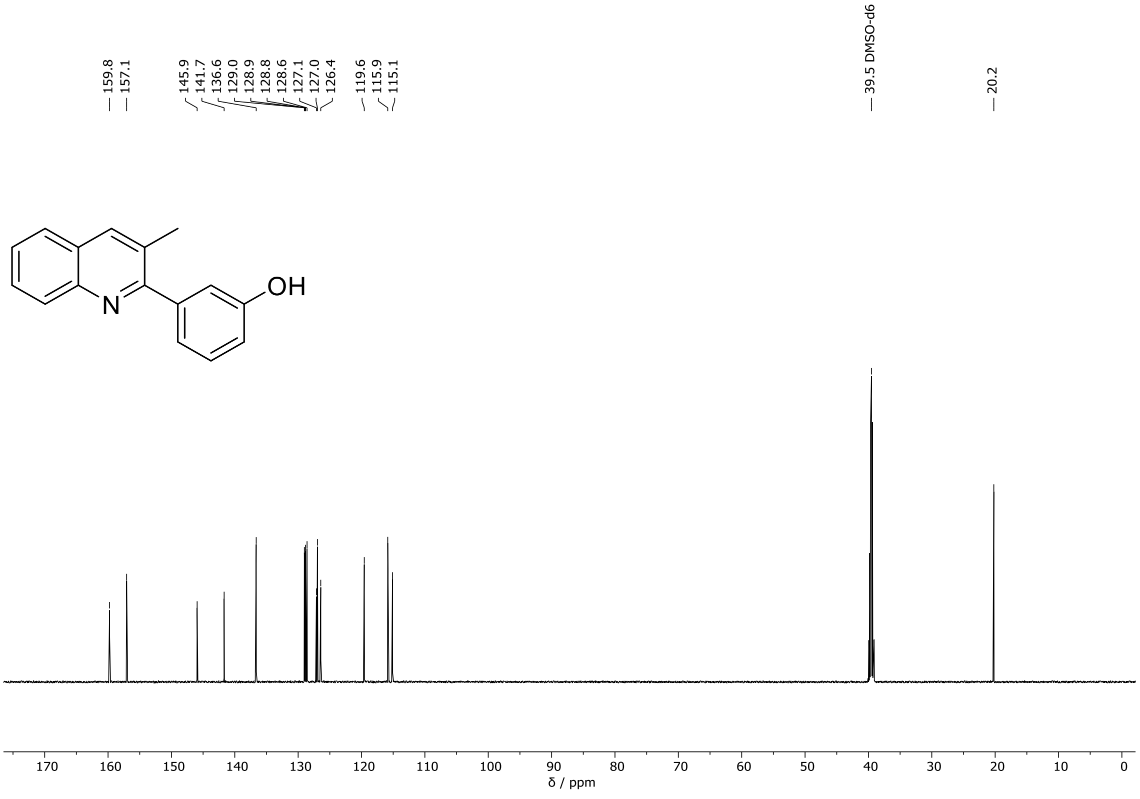

2-(3-methoxyphenyl)quinoline (1m):

^{1}H-NMR (400 MHz, $CDCl_3$)

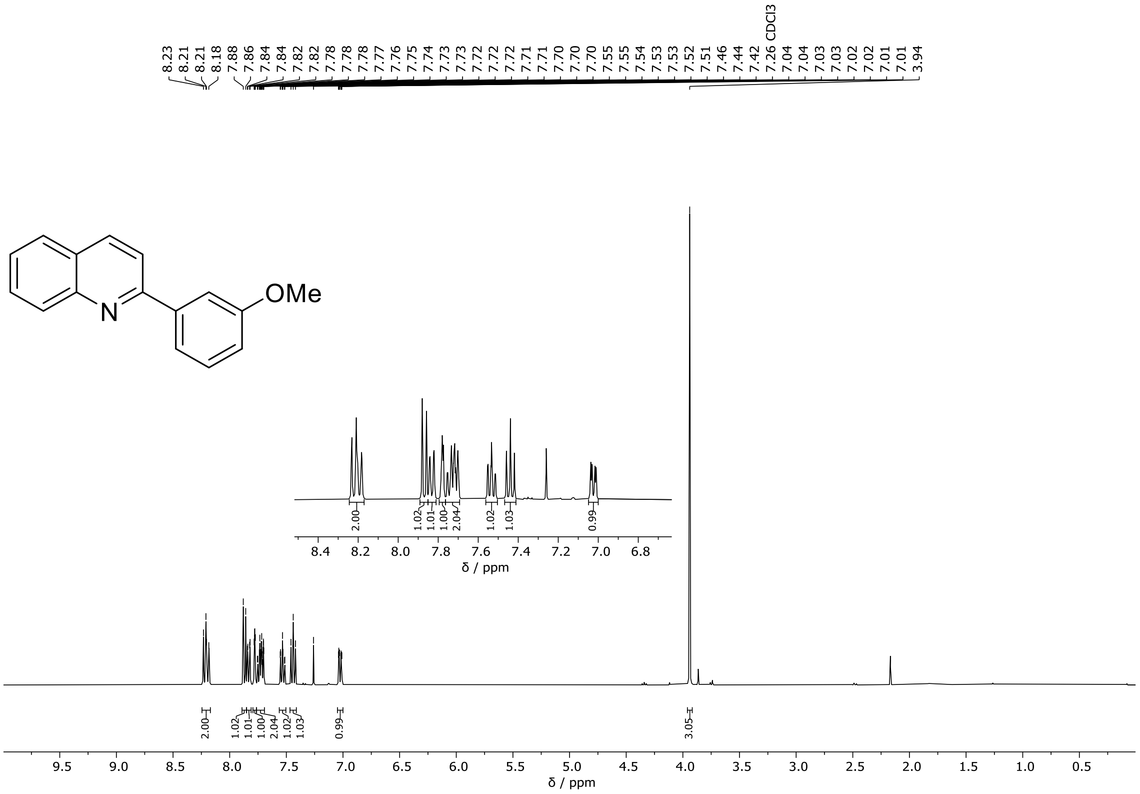

^{13}C-NMR (101 MHz, $CDCl_3$)

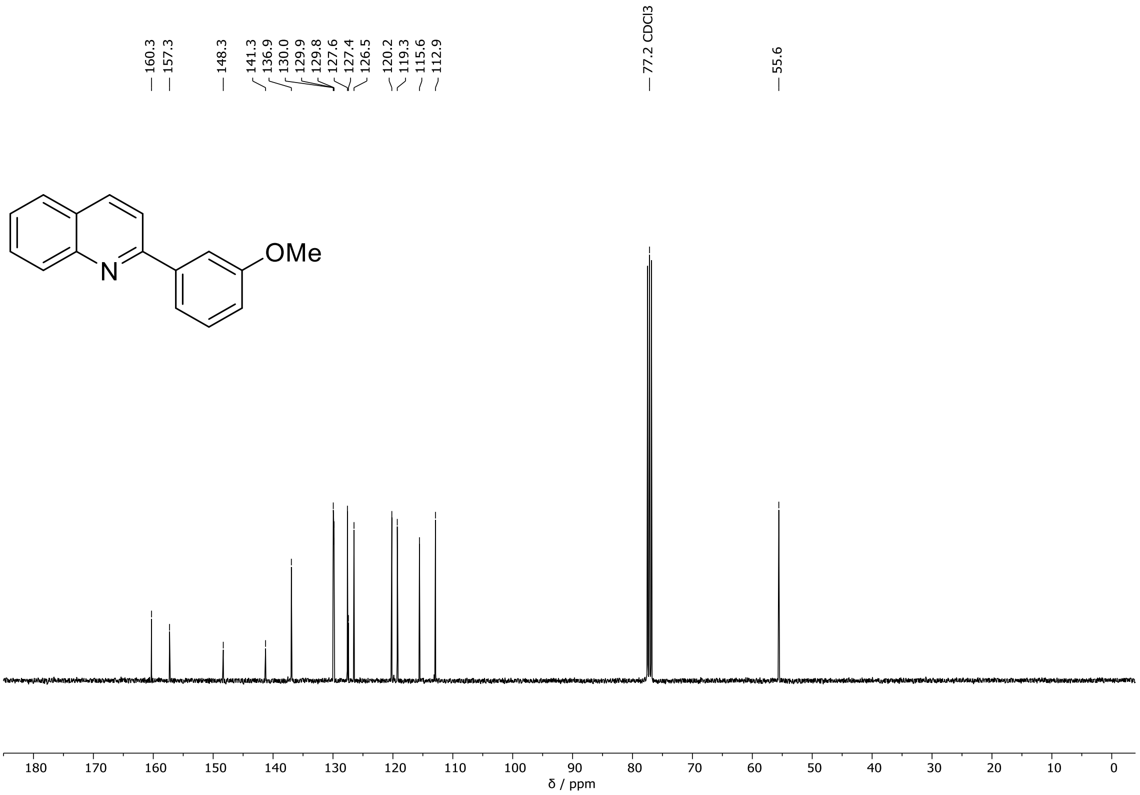

2-(2-methoxyphenyl)quinoline (1n):

^{1}H-NMR (400 MHz, $CDCl_3$)

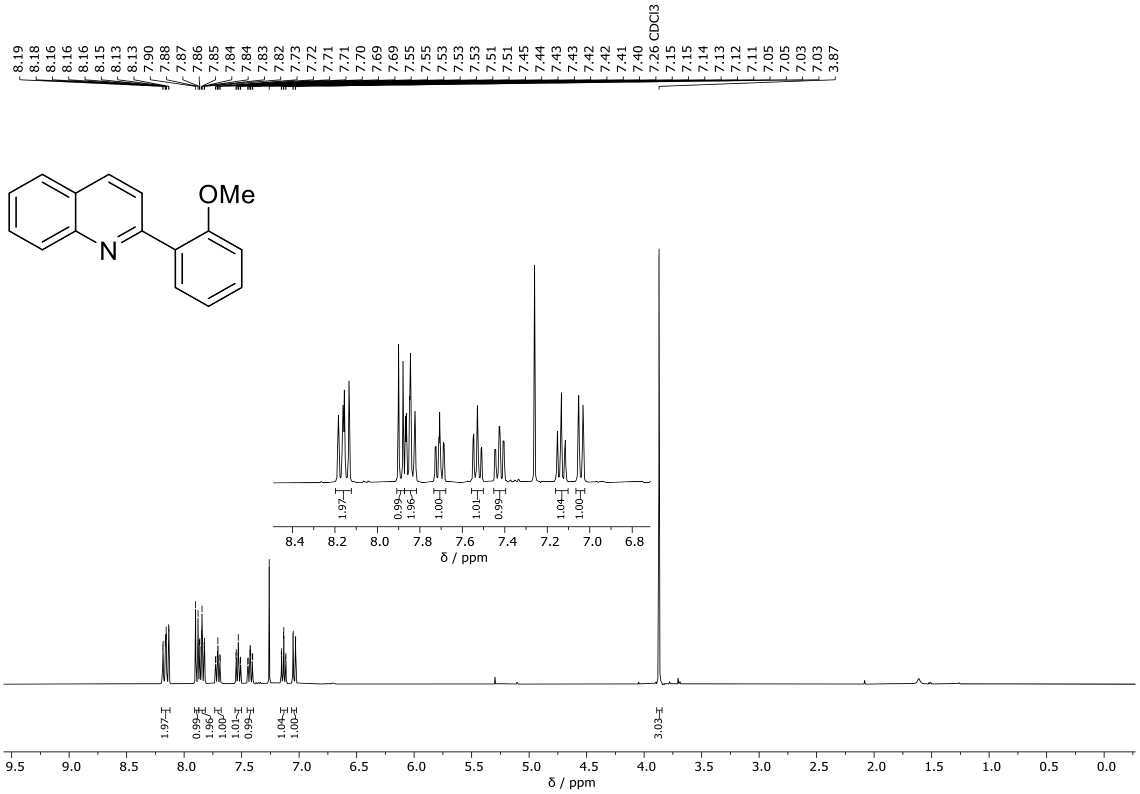

^{13}C-NMR (101 MHz, $CDCl_3$)

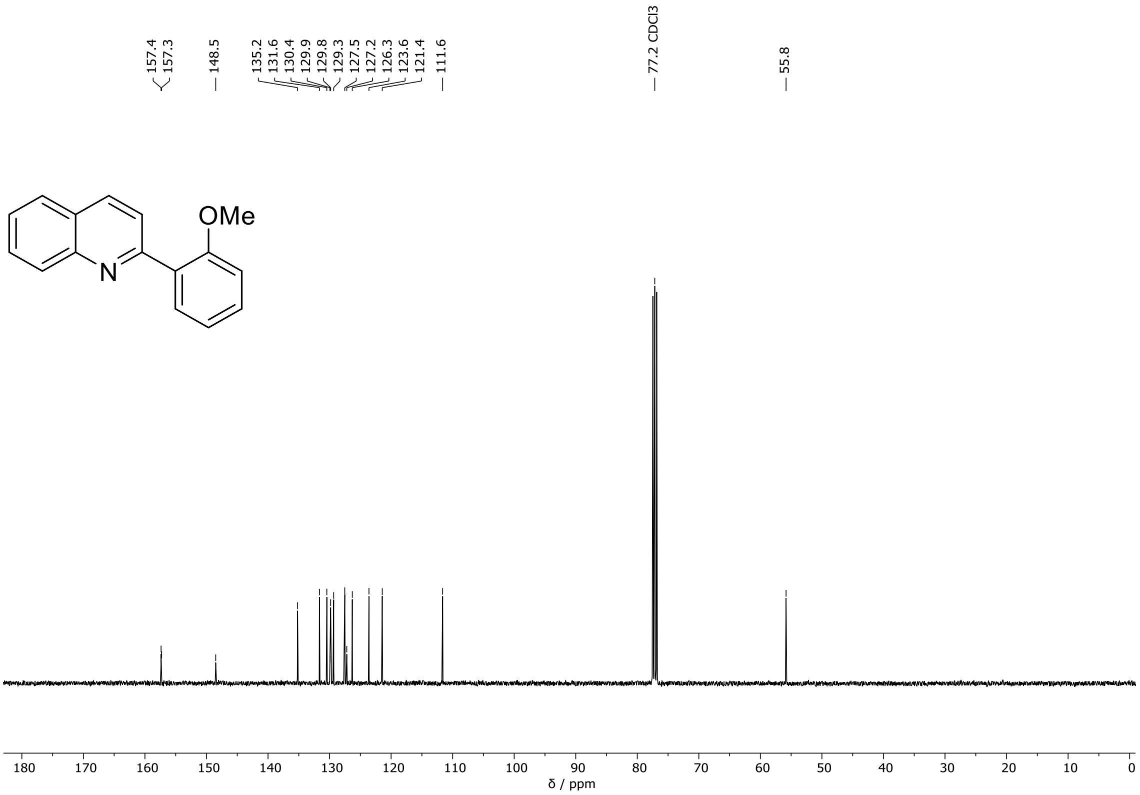

3-isopropyl-5-methyl-2-phenylquinoline (1o):

^{1}H-NMR (400 MHz, $CDCl_3$)

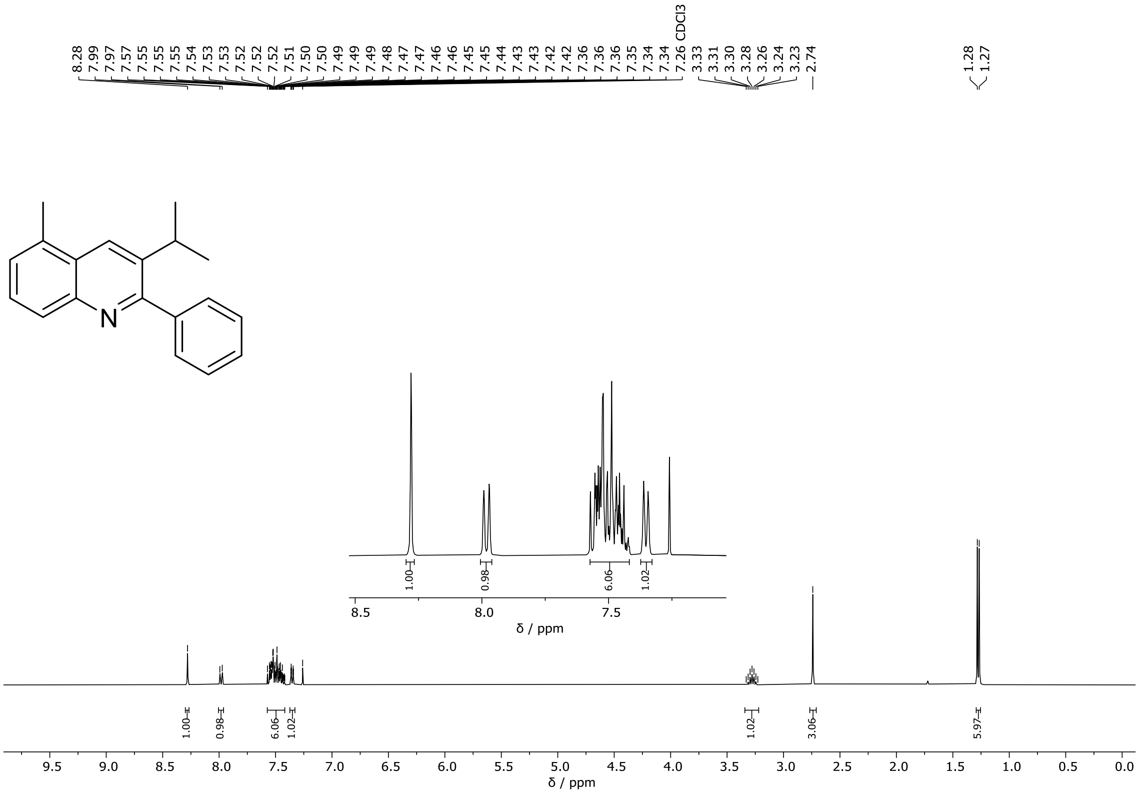

^{13}C-NMR (101 MHz, $CDCl_3$)

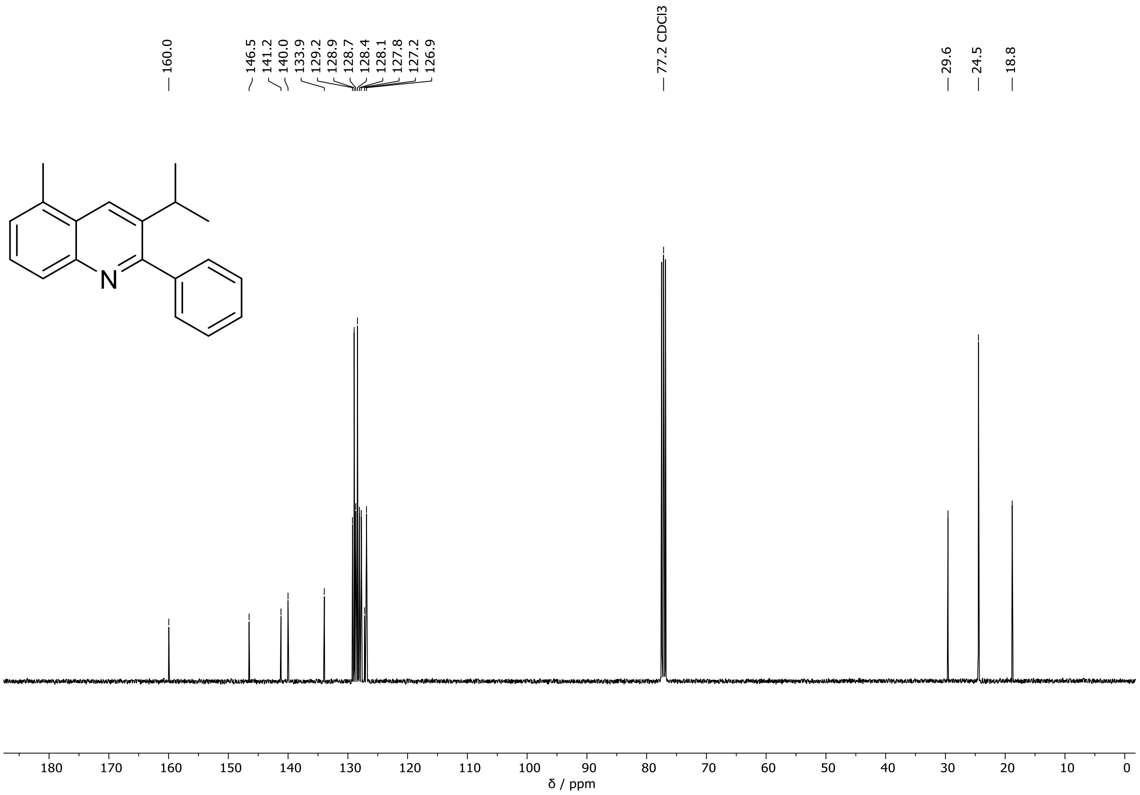

3-isopropyl-6-methyl-2-phenylquinoline (1p):

^{1}H-NMR (400 MHz, $CDCl_3$)

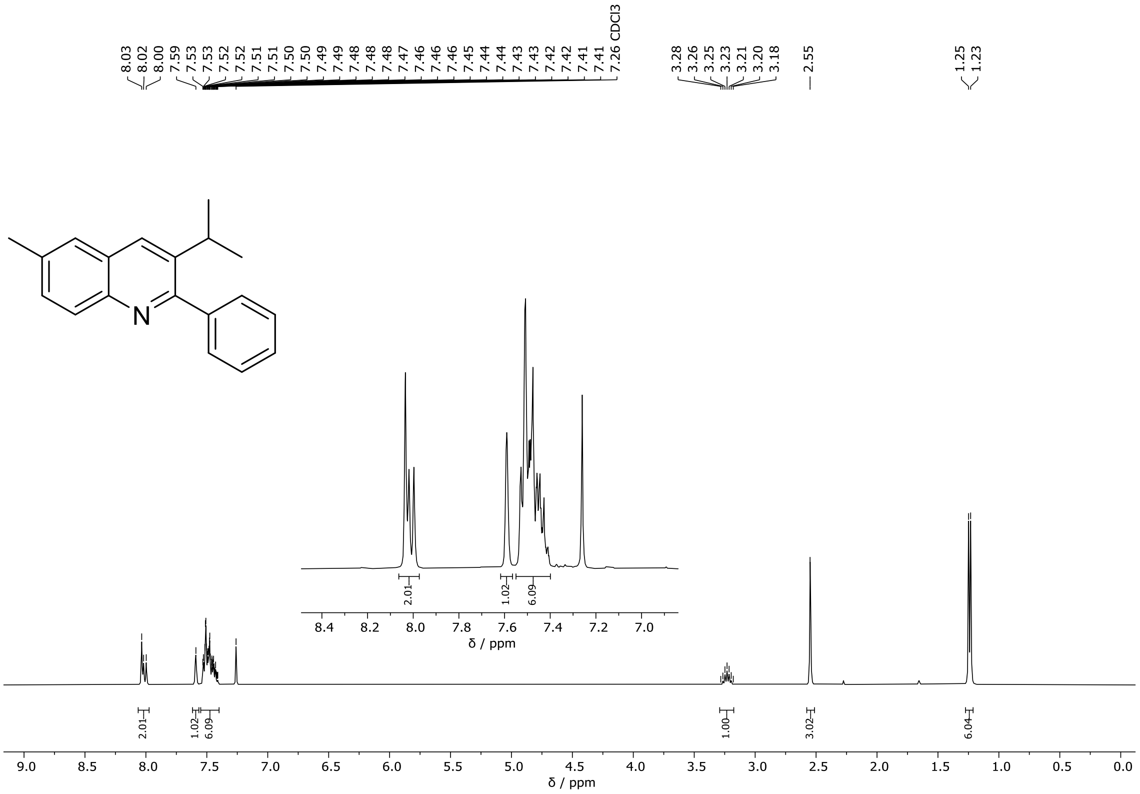

^{13}C-NMR (101 MHz, $CDCl_3$)

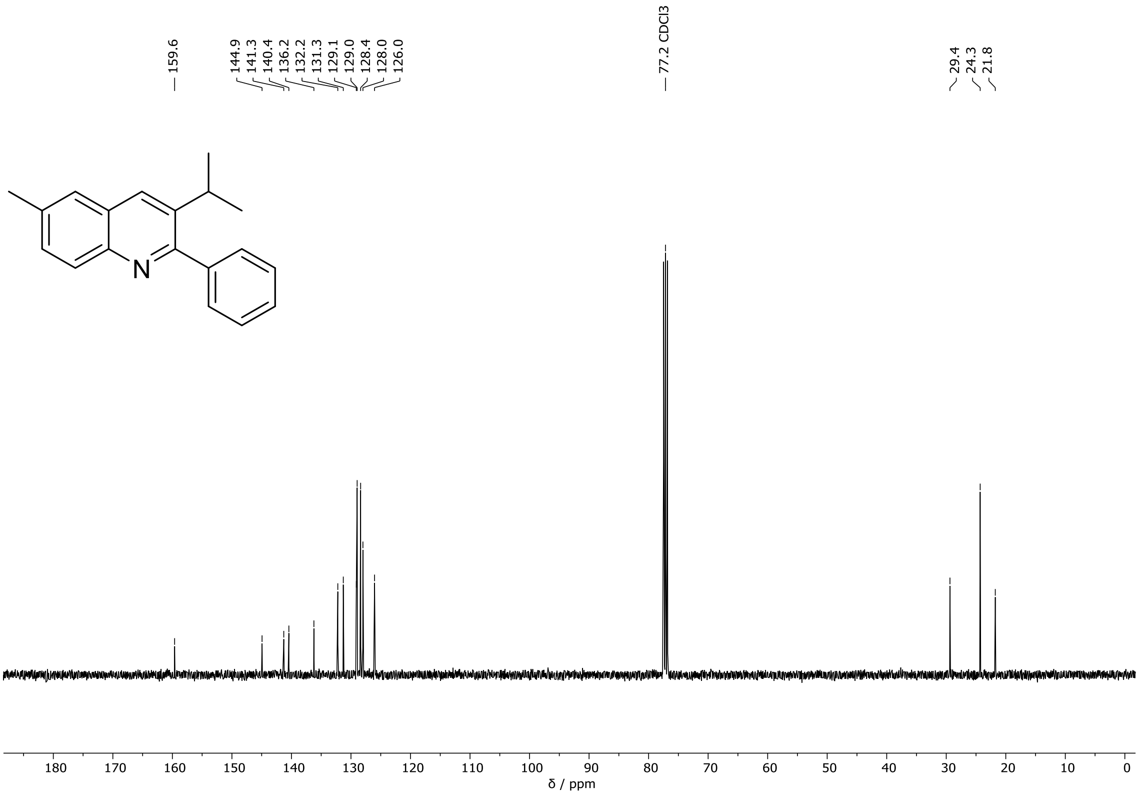

3-isopropyl-7-methyl-2-phenylquinoline (1q):

^{1}H-NMR (400 MHz, $CDCl_3$)

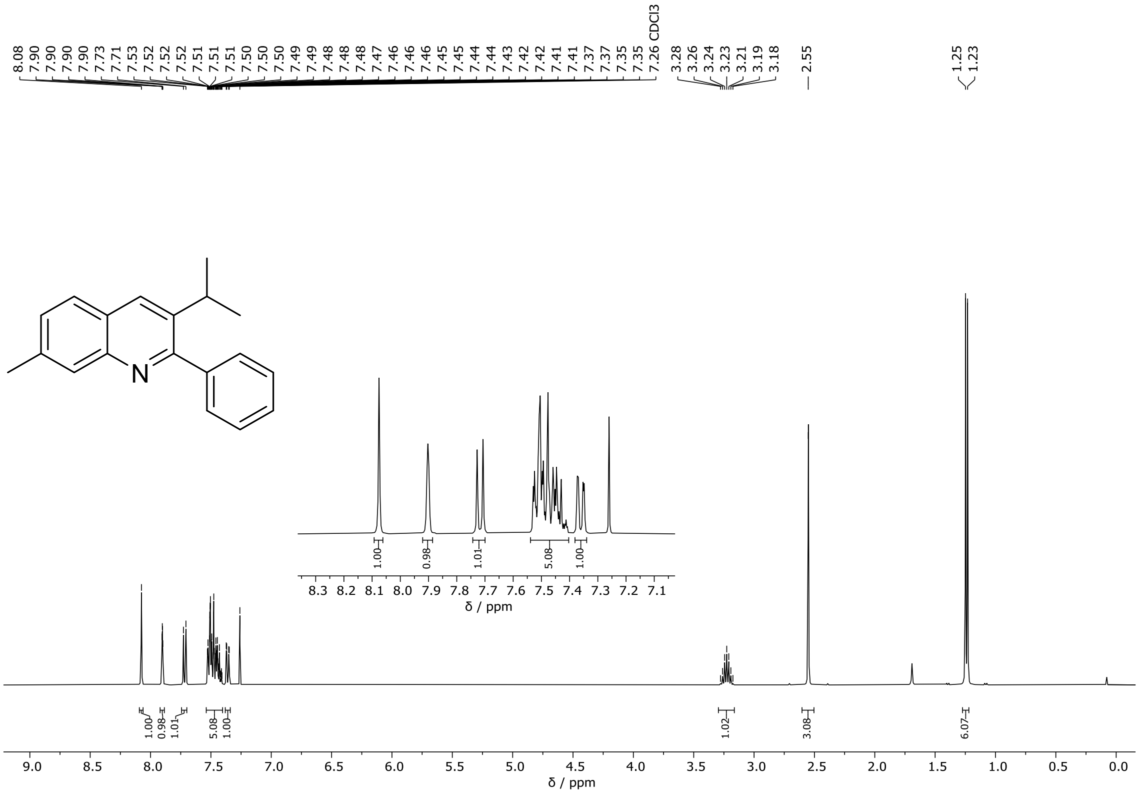

^{13}C-NMR (101 MHz, $CDCl_3$)

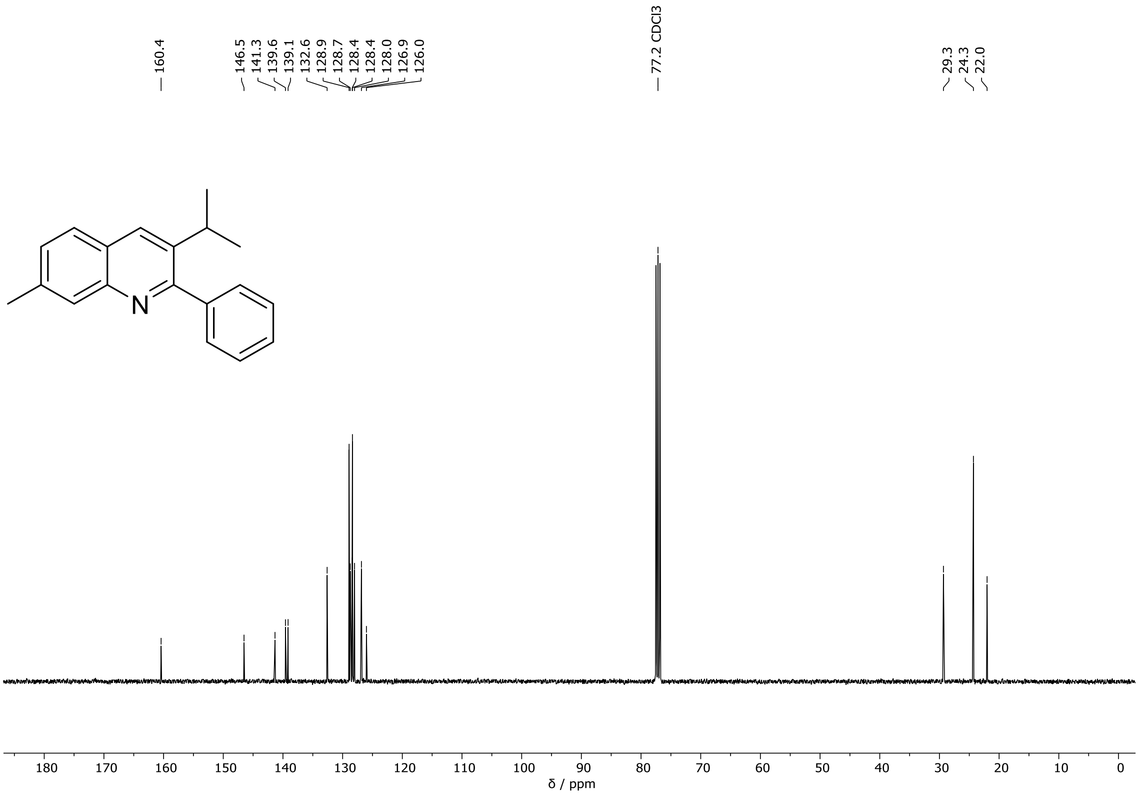

3-isopropyl-8-methyl-2-phenylquinoline (1r):

^{1}H-NMR (400 MHz, $CDCl_3$)

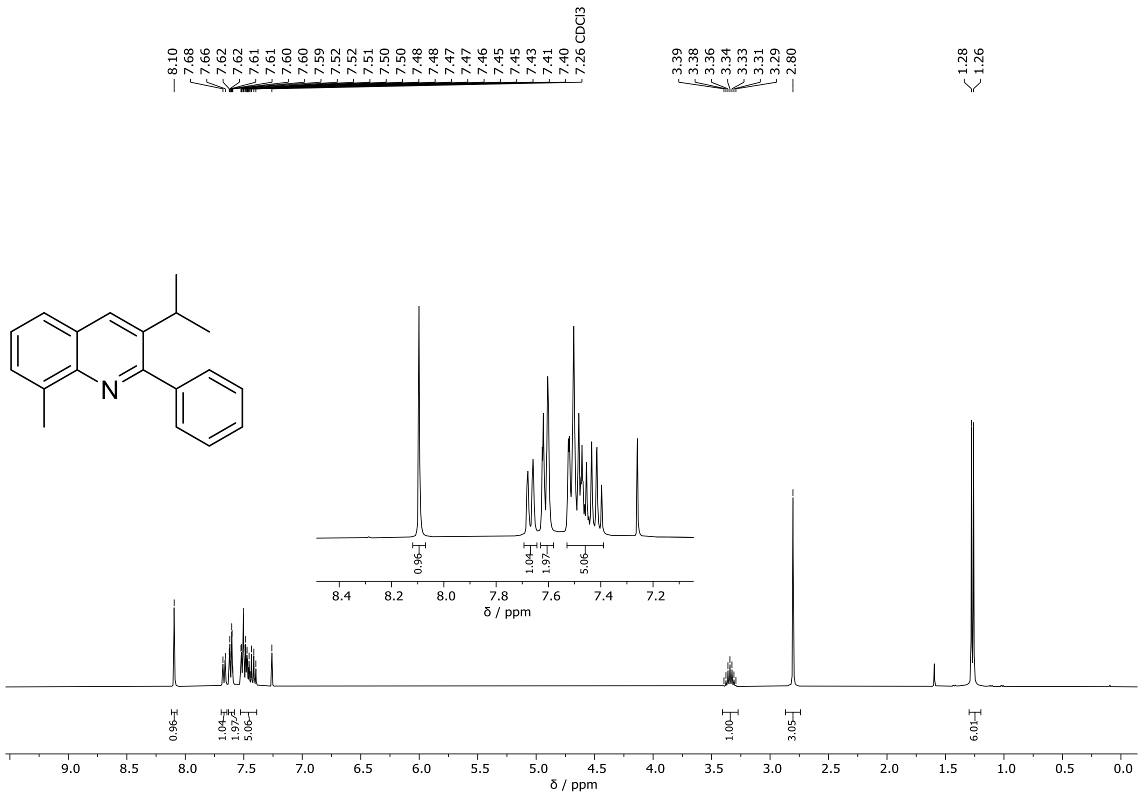

^{13}C-NMR (101 MHz, $CDCl_3$)

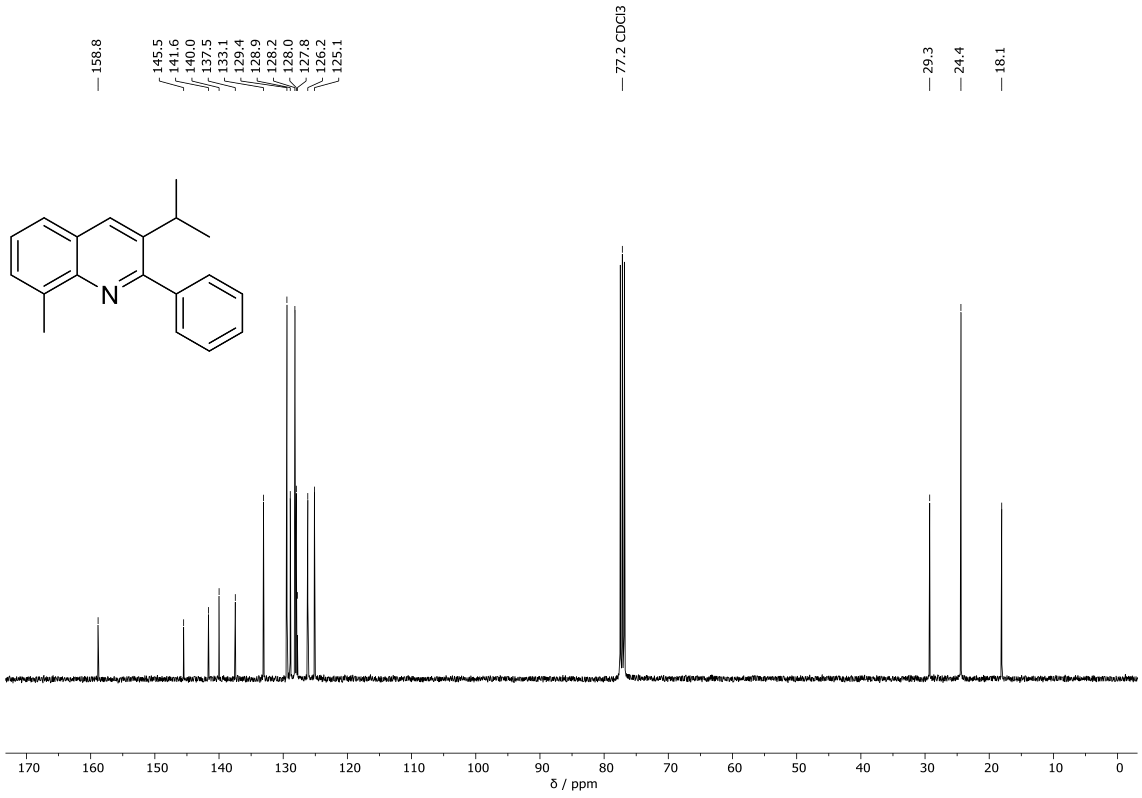

6-bromo-3-isopropyl-2-phenylquinoline (1s):

^{1}H-NMR (300 MHz, $CDCl_3$)

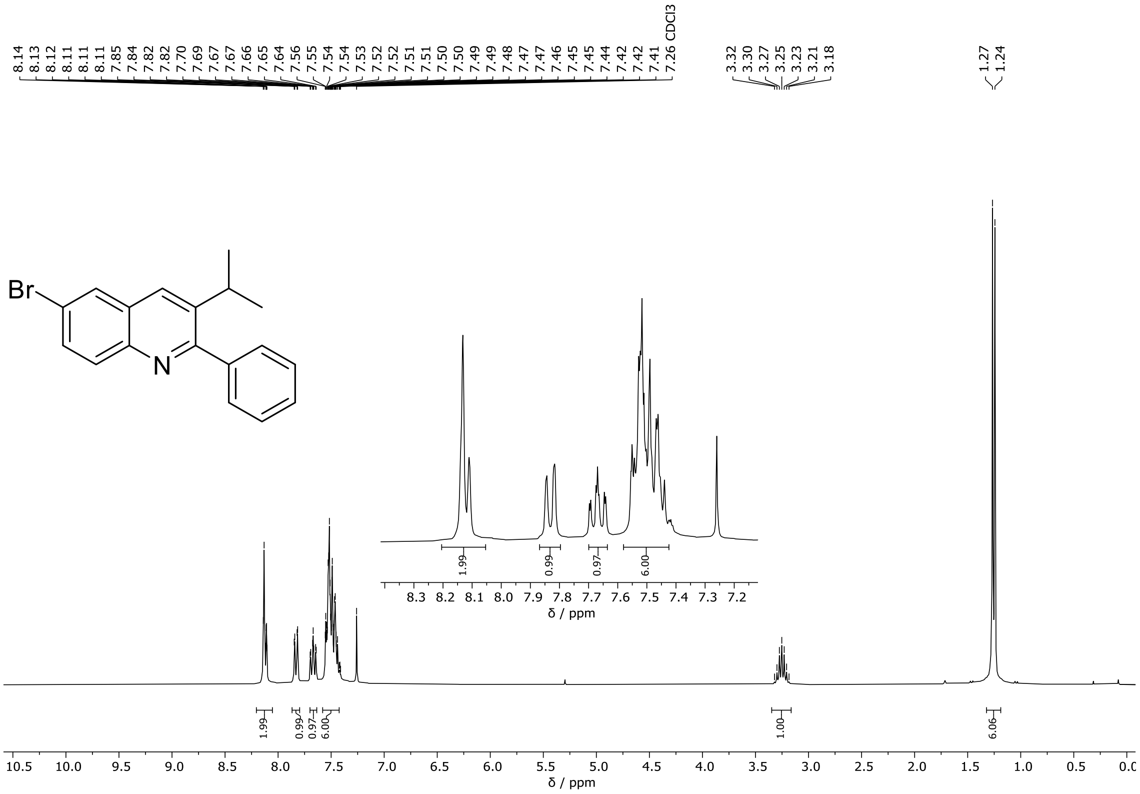

^{13}C-NMR (101 MHz, $CDCl_3$)

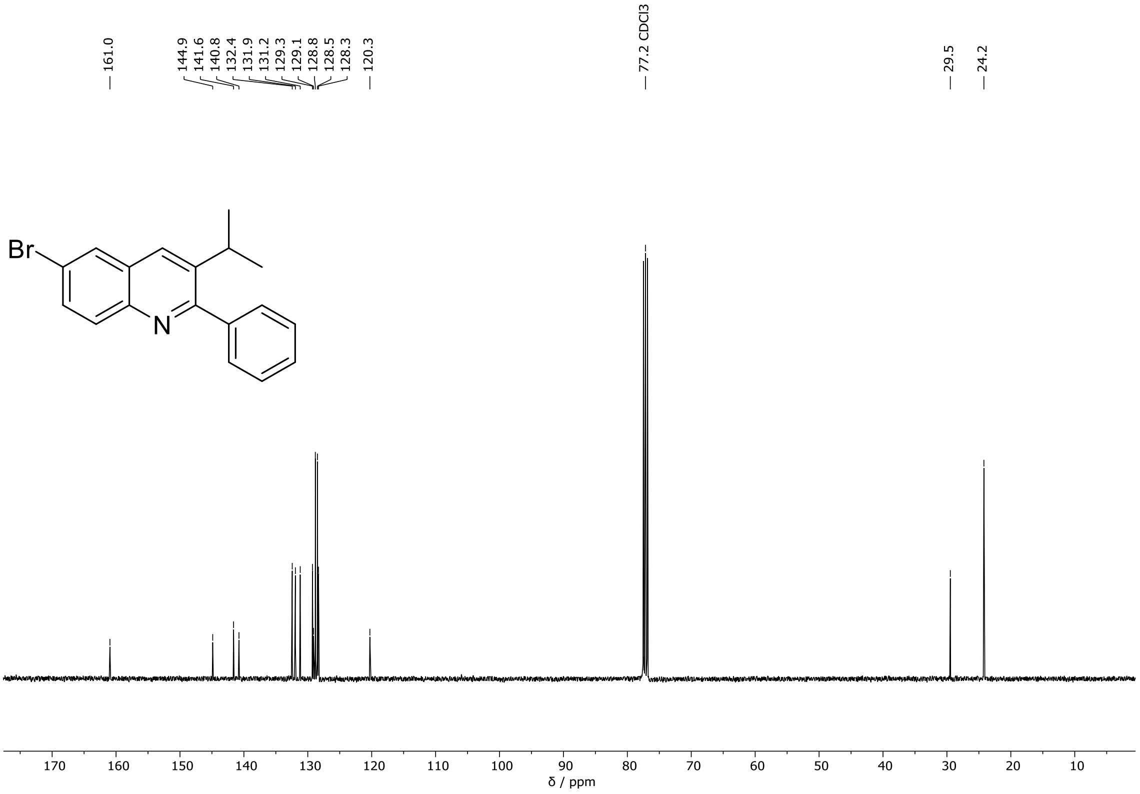

7-chloro-3-isopropyl-2-phenylquinoline (1t):

^{1}H-NMR (400 MHz, $CDCl_3$)

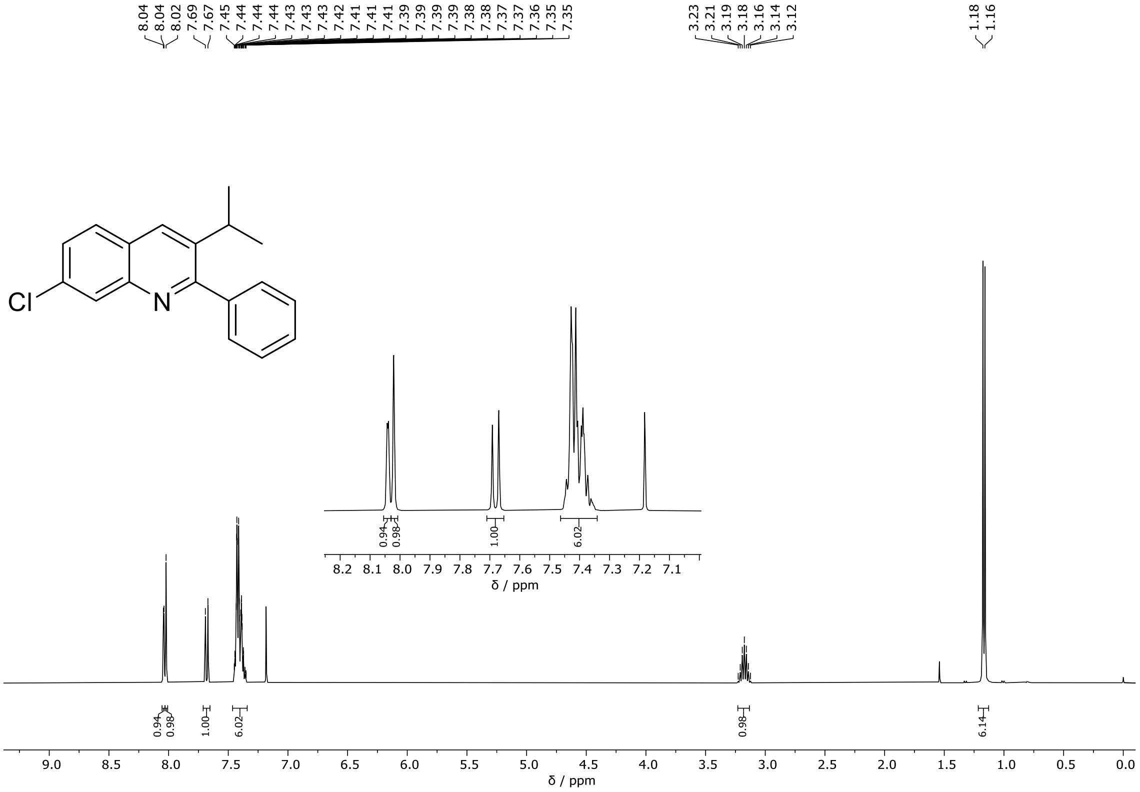

^{13}C-NMR (101 MHz, $CDCl_3$)

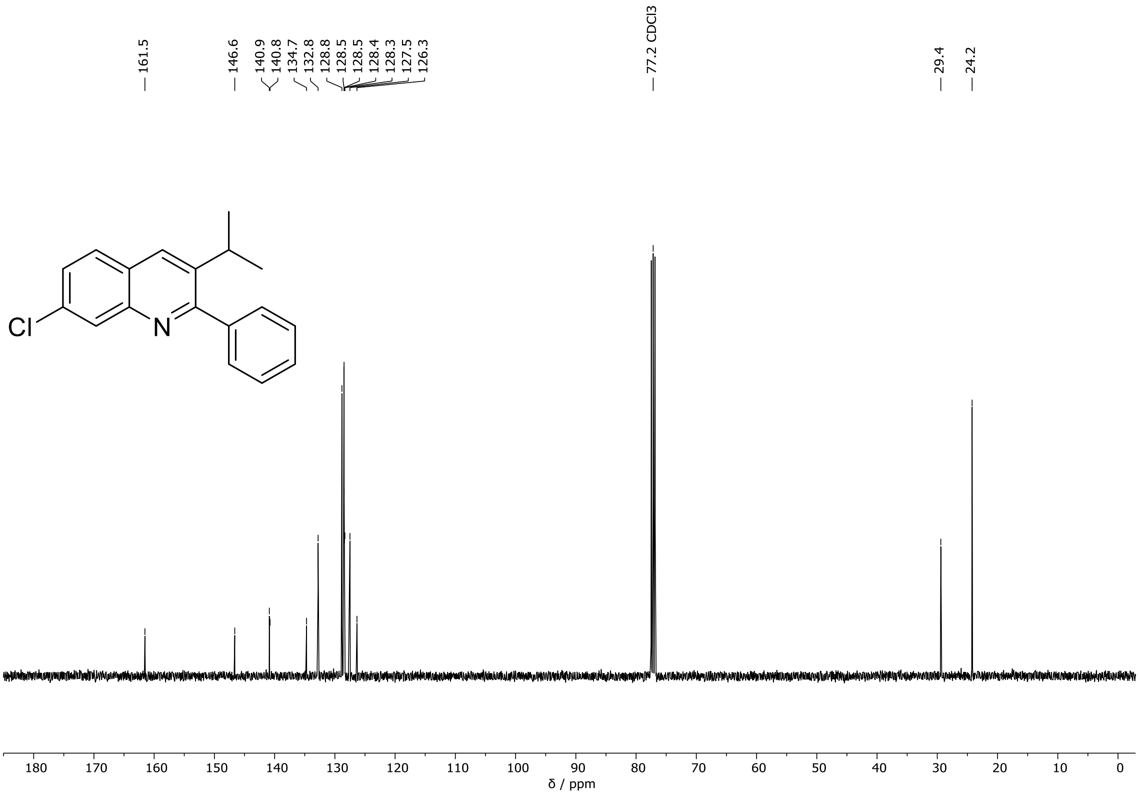

6-fluoro-3-methyl-2-phenylquinoline (1u):

^{1}H-NMR (400 MHz, $CDCl_3$)

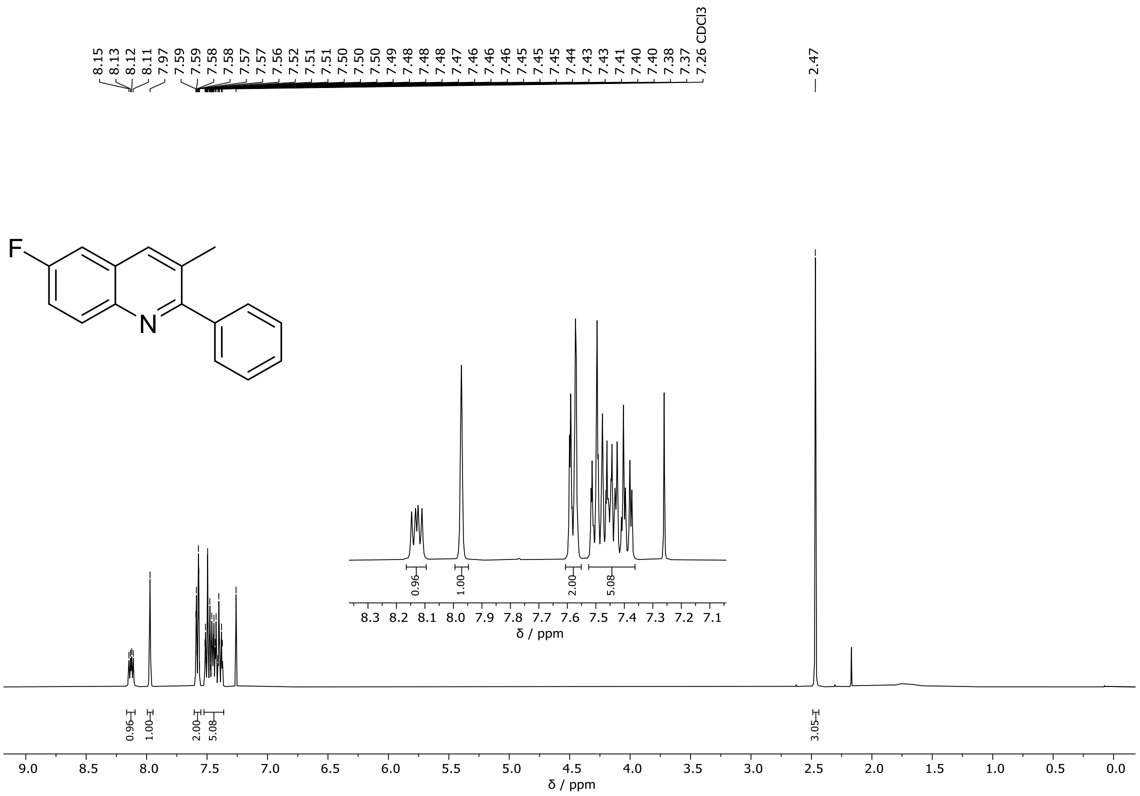

^{13}C-NMR (101 MHz, $CDCl_3$)

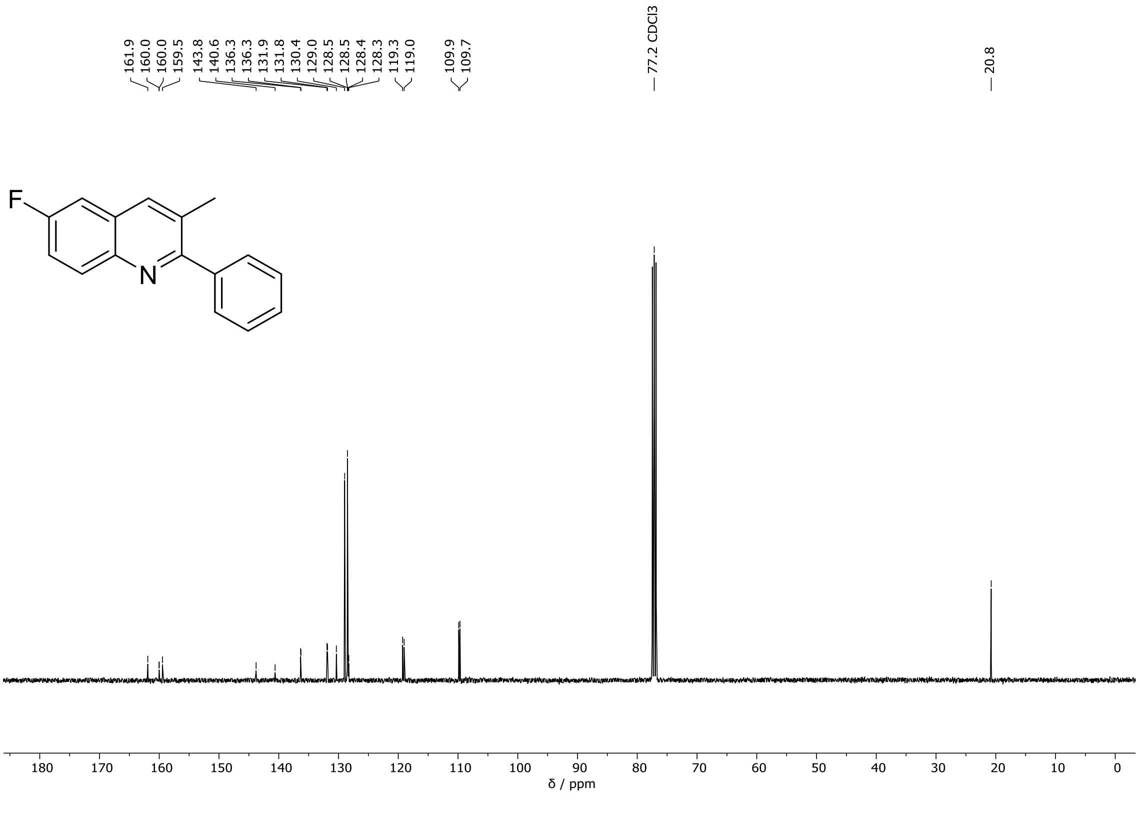

^{19}F-NMR (282 MHz, $CDCl_3$)

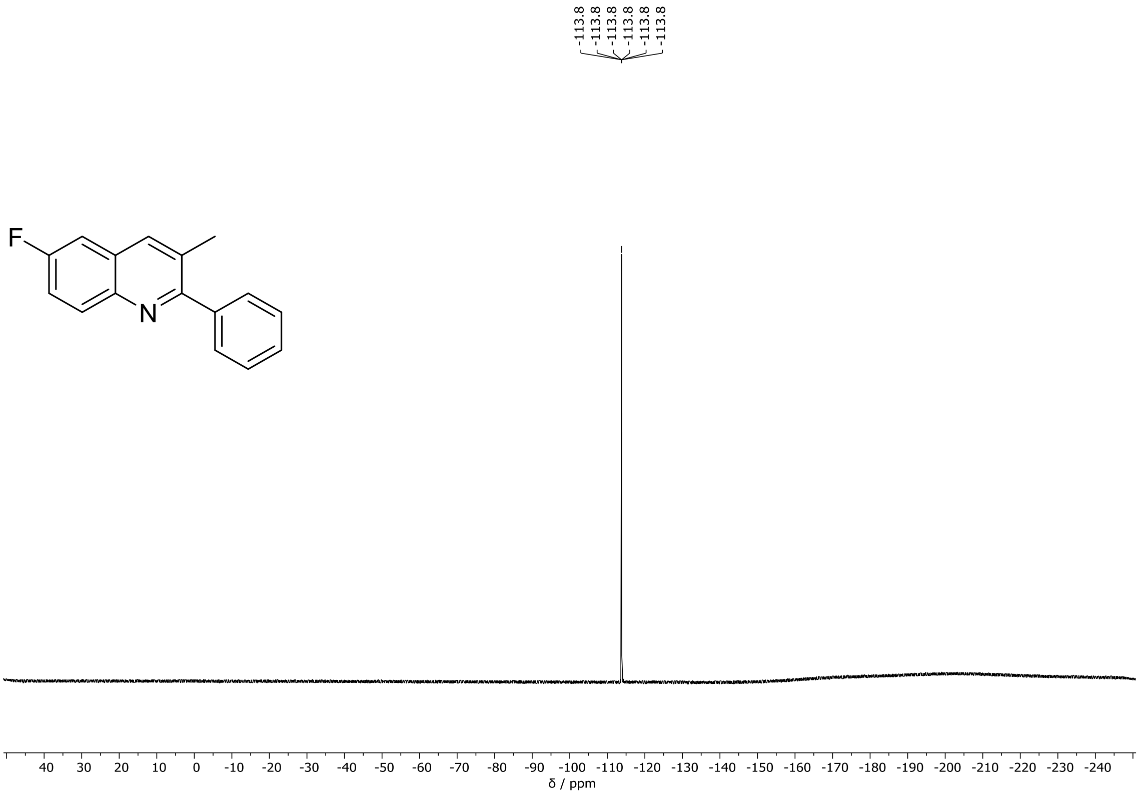

methyl 2-phenylquinoline-6-carboxylate (1v):

^{1}H-NMR (300 MHz, $CDCl_3$)

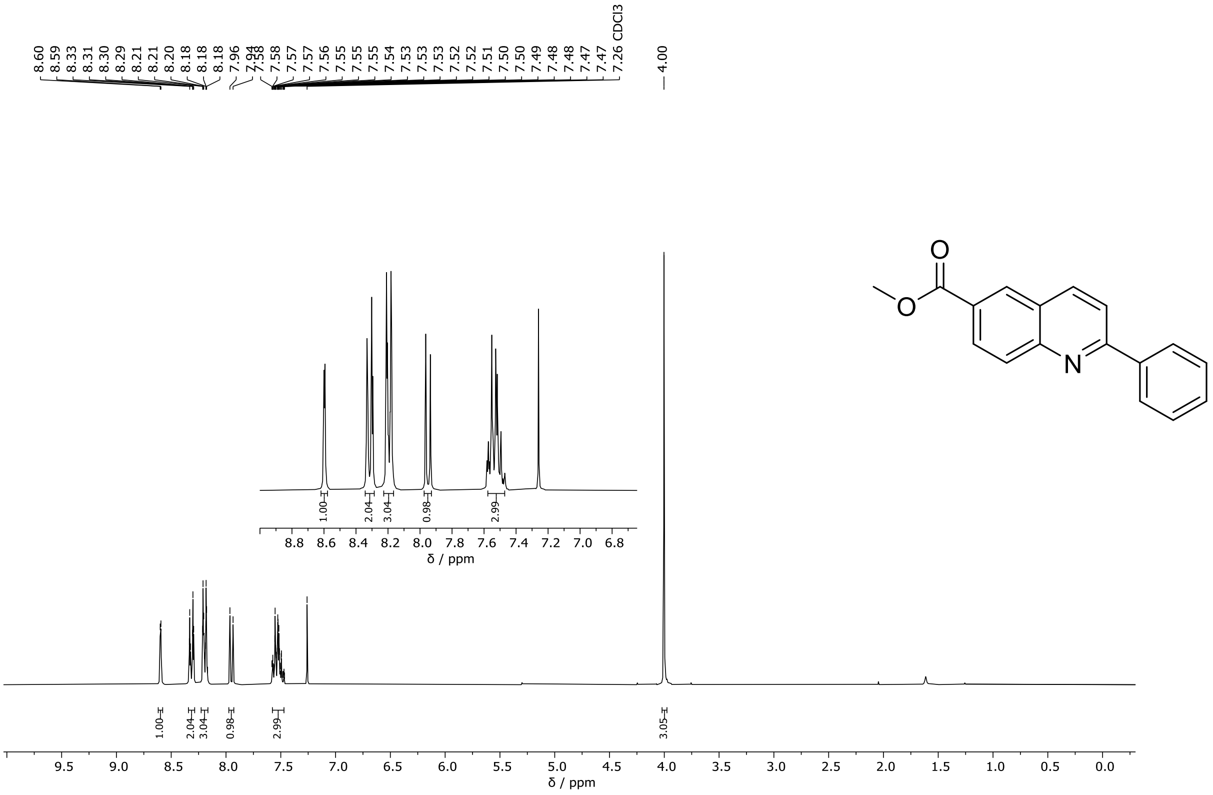

^{13}C-NMR (76 MHz, $CDCl_3$)

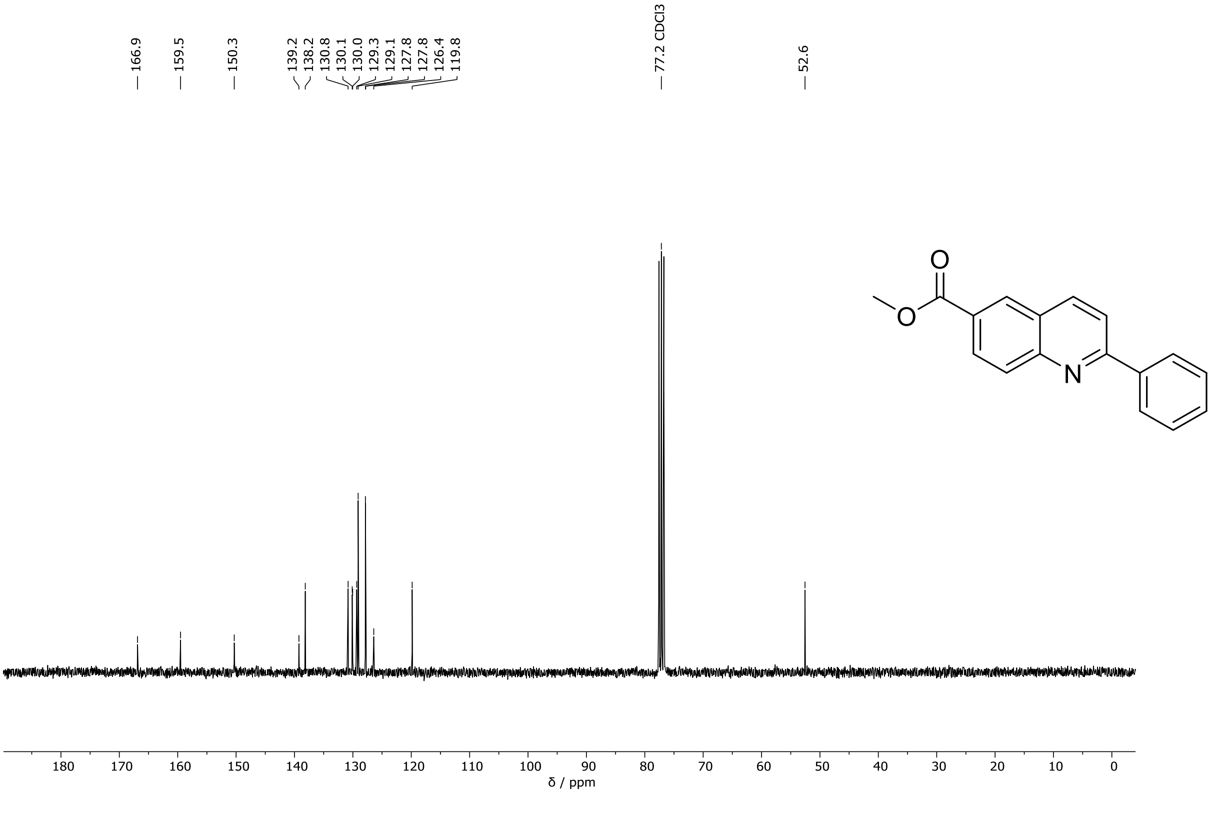

ethyl 2-phenylquinoline-3-carboxylate (1w):

^{1}H-NMR (400 MHz, CDCl$_3$)

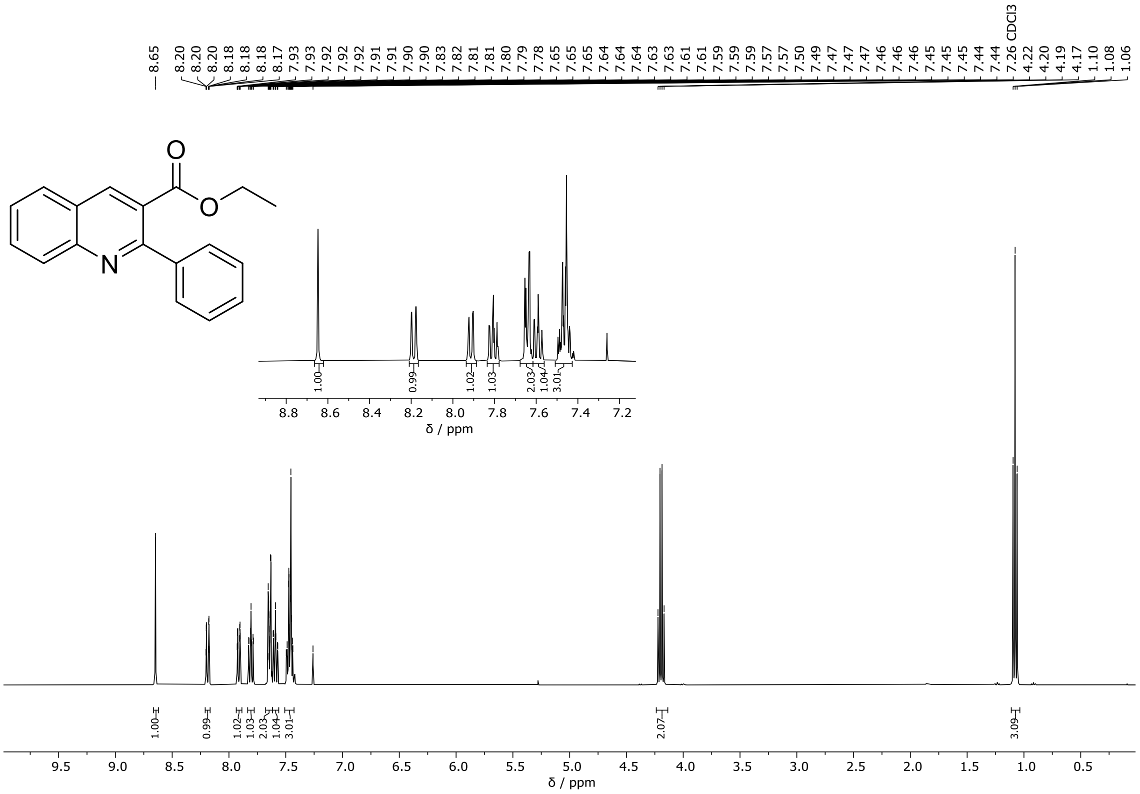

^{13}C-NMR (101 MHz, CDCl$_3$)

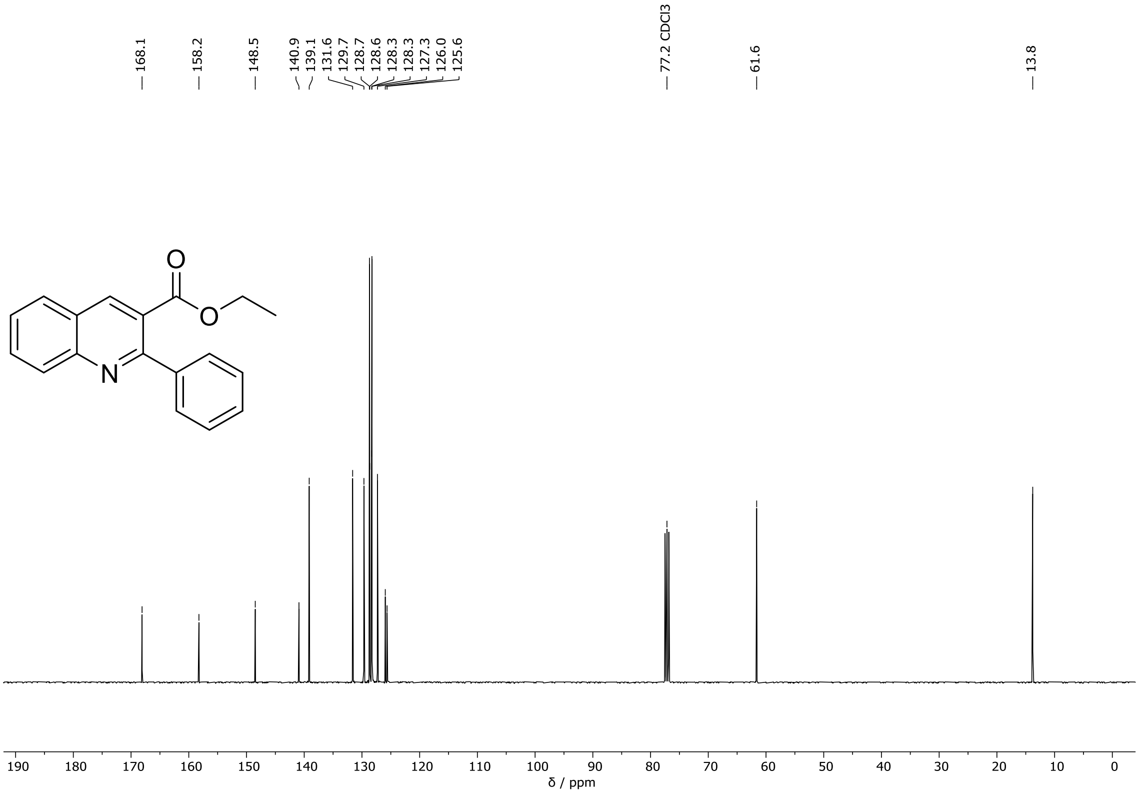

2-chloro-*N*-isopropylquinoline-3-carboxamide:

^{1}H-NMR (400 MHz, $CDCl_3$)

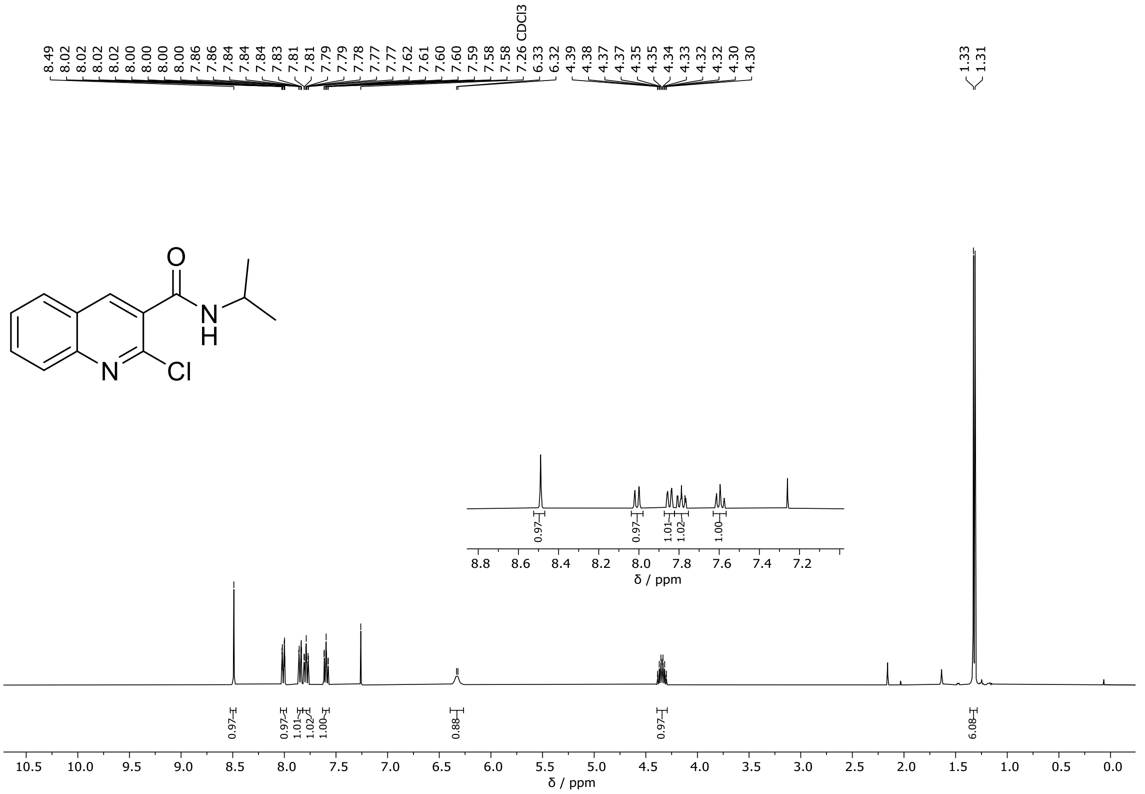

^{13}C-NMR (101 MHz, $CDCl_3$)

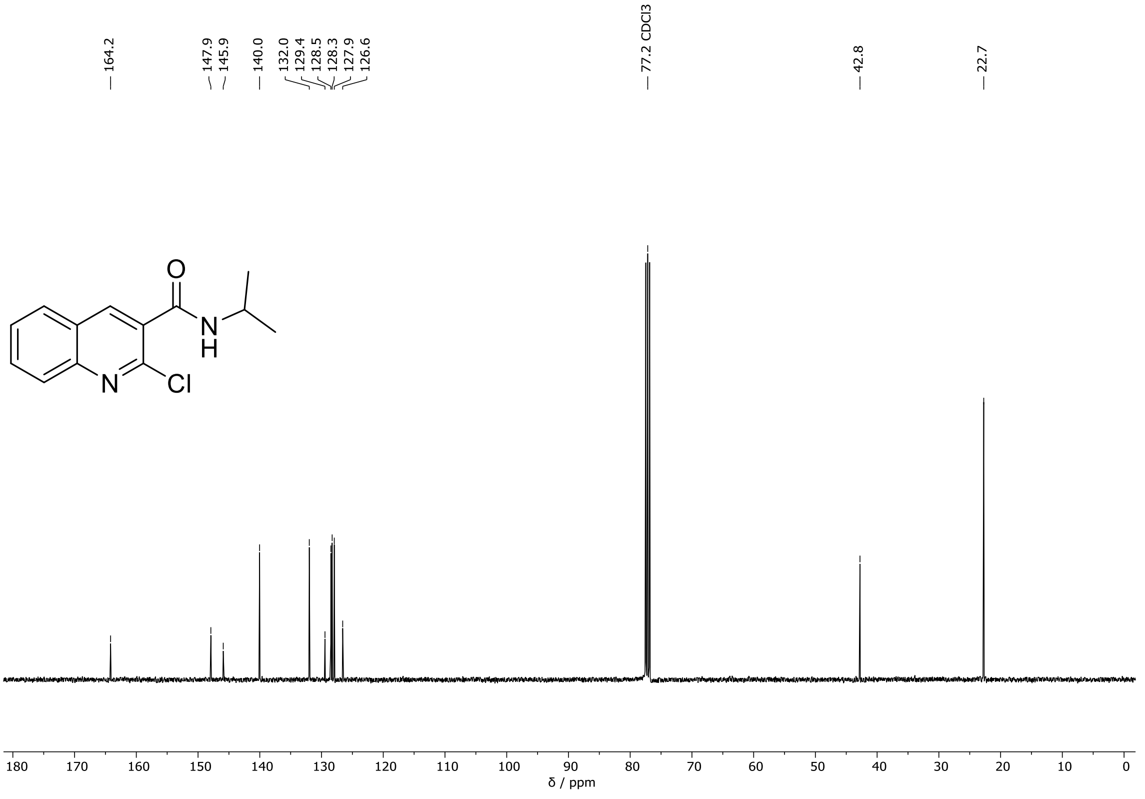

***N*-isopropyl-2-phenylquinoline-3-carboxamide (1x):**

^{1}H-NMR (400 MHz, $CDCl_3$)

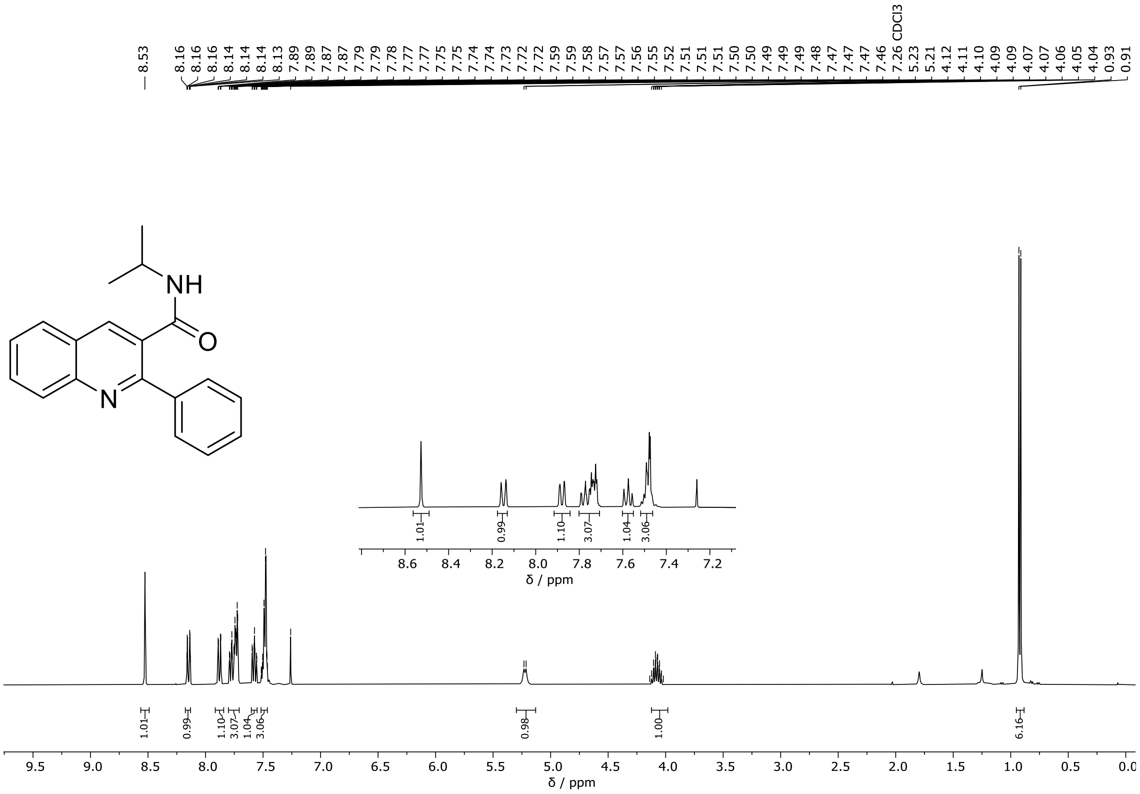

^{13}C-NMR (76 MHz, $CDCl_3$)

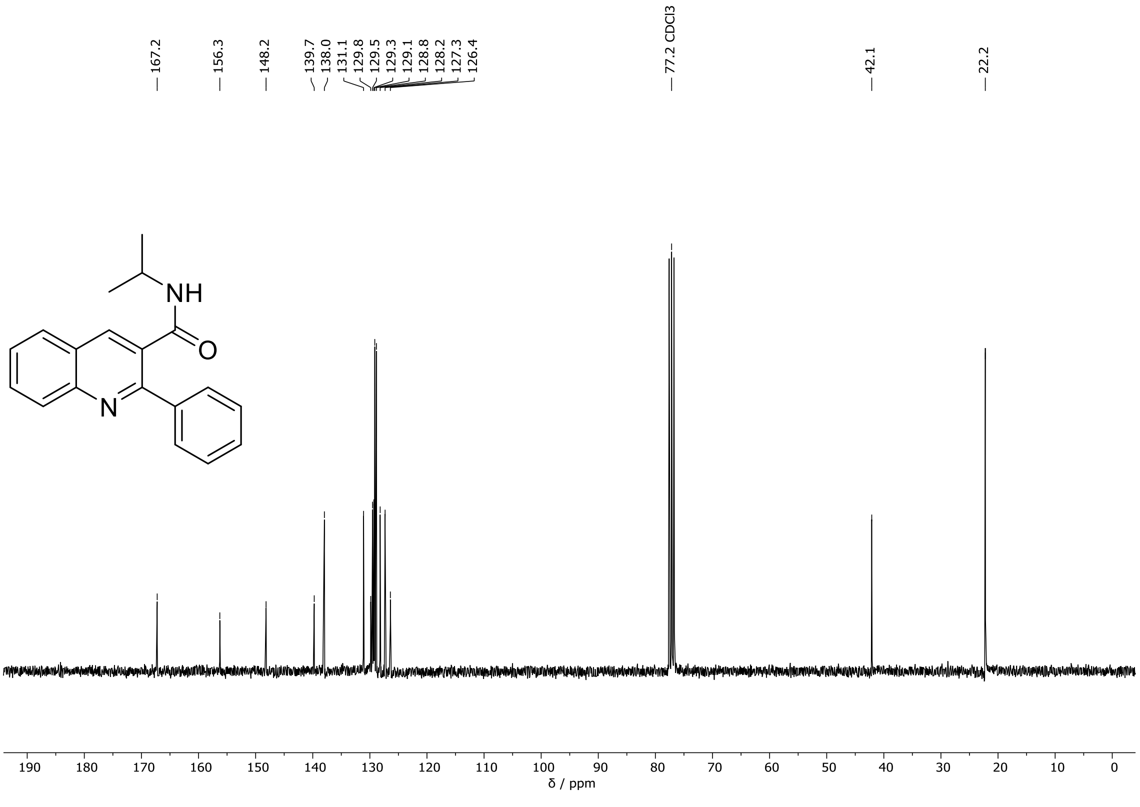

(2-chloroquinolin-3-yl)(pyrrolidin-1-yl)methanone:

^{1}H-NMR (599 MHz, $CDCl_3$)

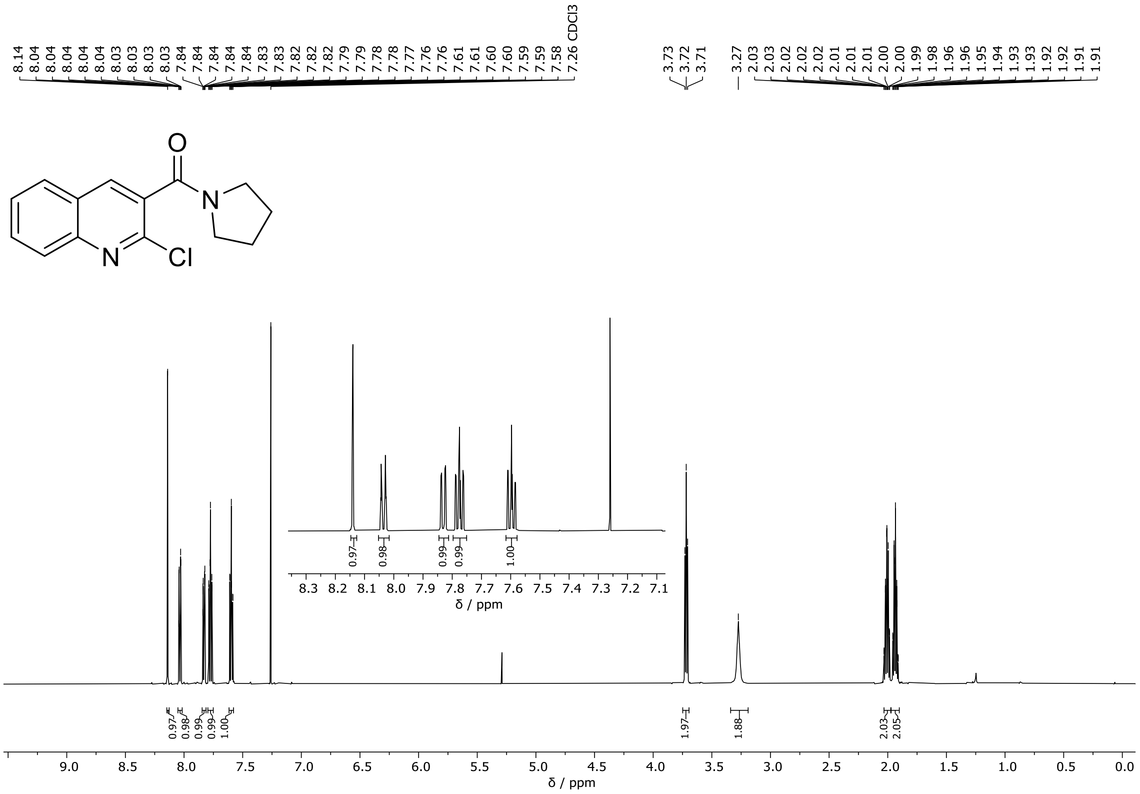

^{13}C-NMR (151 MHz, $CDCl_3$)

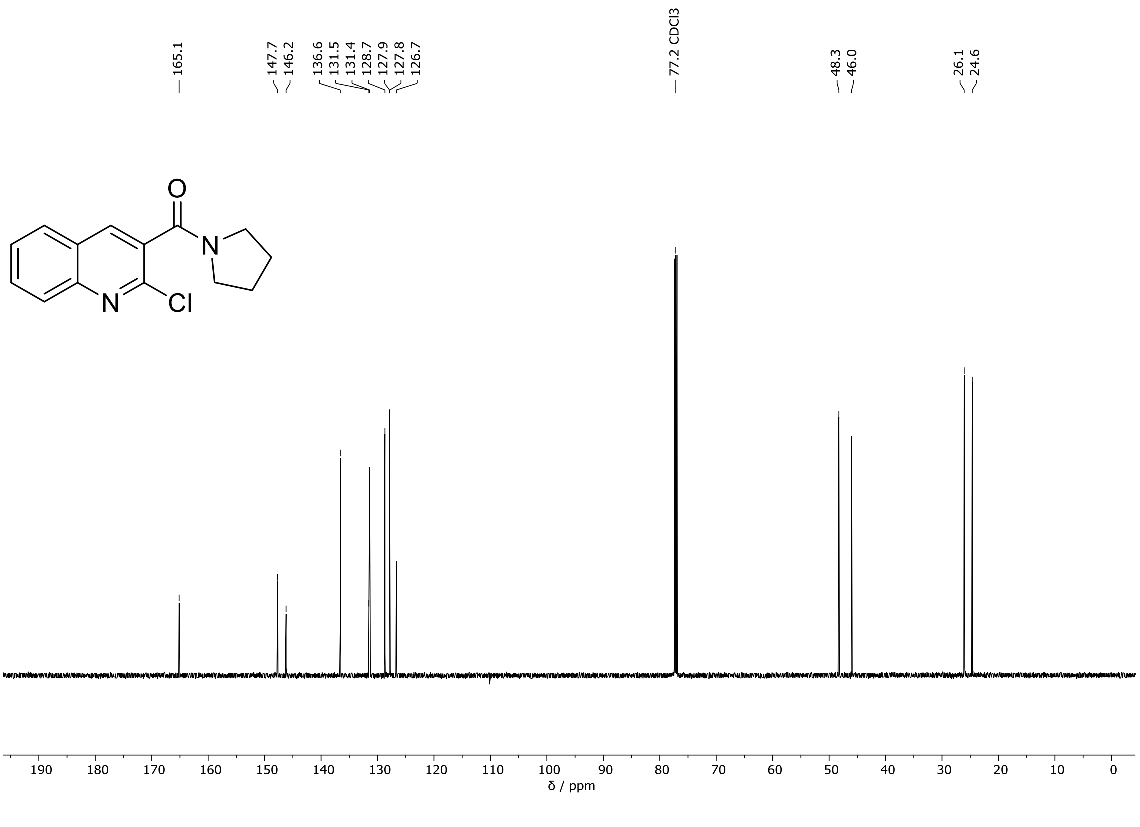

(2-phenylquinolin-3-yl)(pyrrolidin-1-yl)methanone (1y):

^{1}H-NMR (599 MHz, $CDCl_3$)

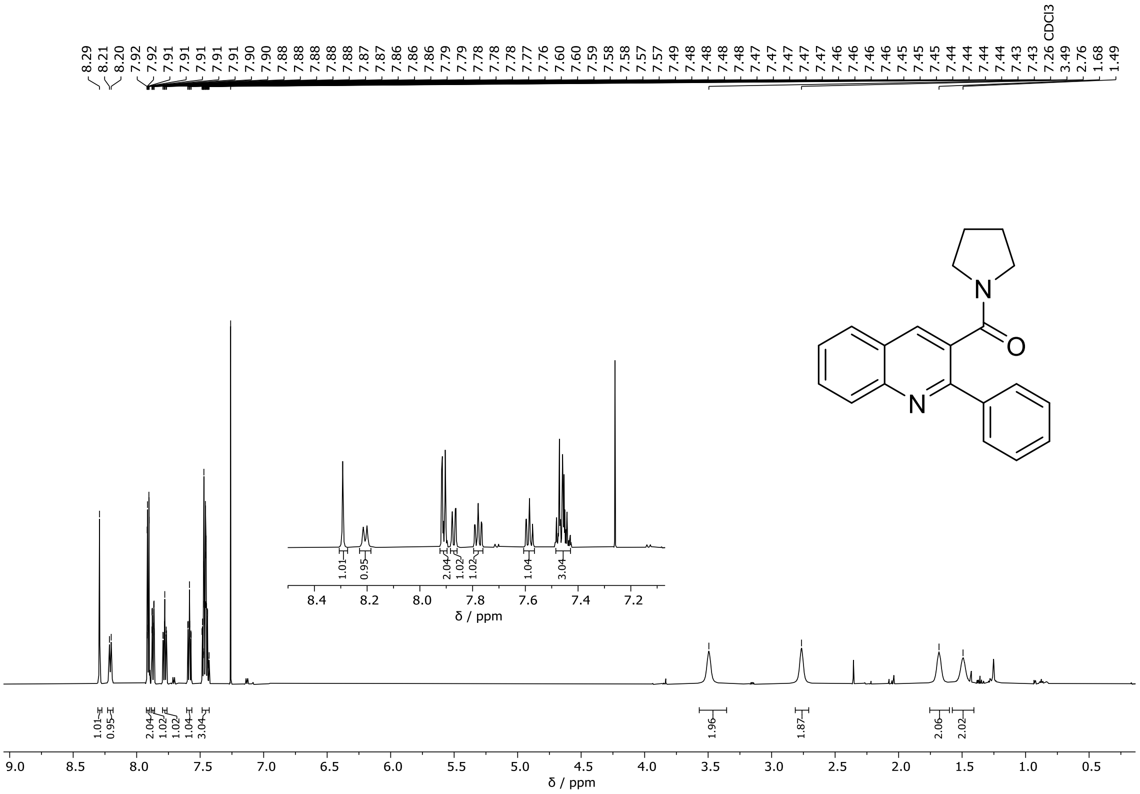

^{13}C-NMR (151 MHz, $CDCl_3$)

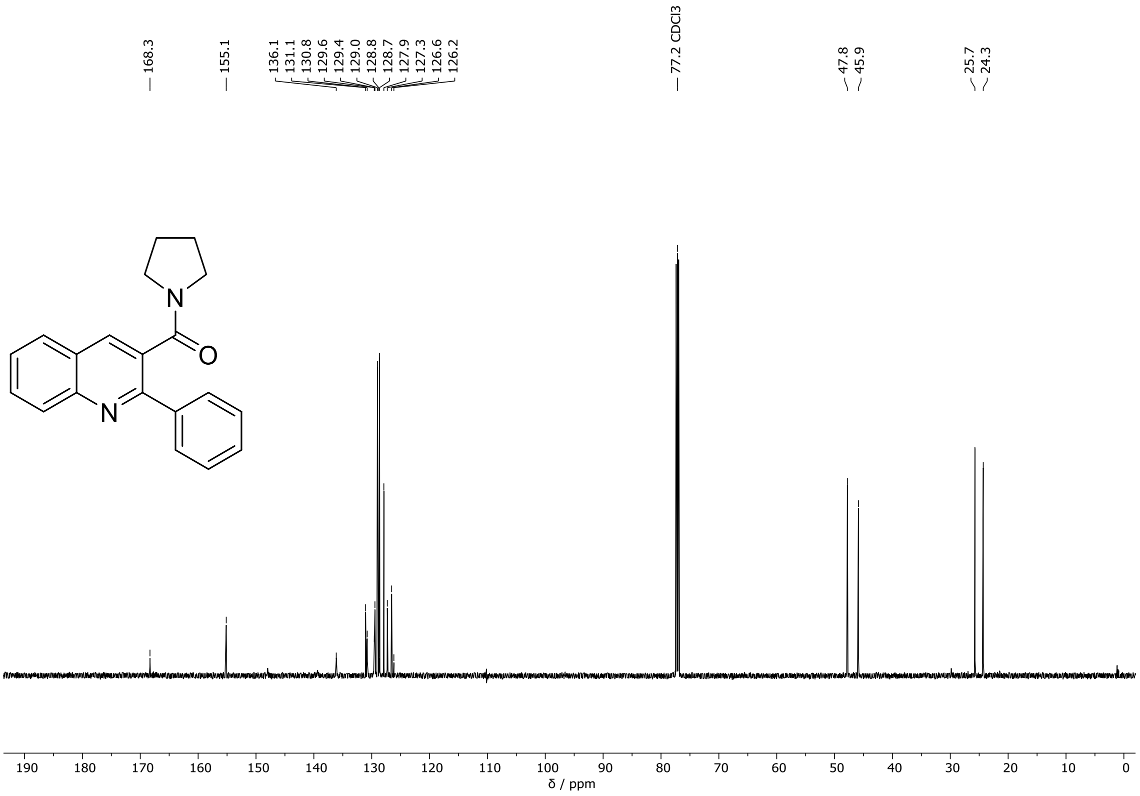

2,3-diphenylquinoline (1z):

^{1}H-NMR (400 MHz, $CDCl_3$)

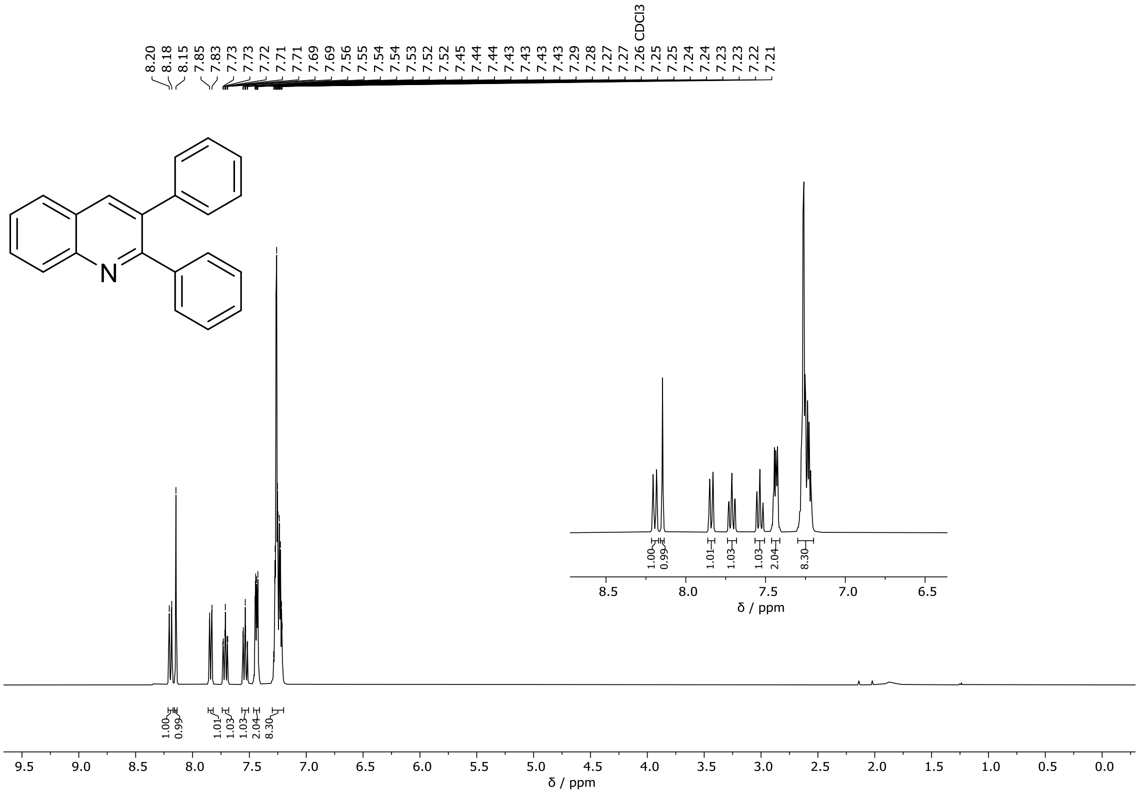

^{13}C-NMR (101 MHz, $CDCl_3$)

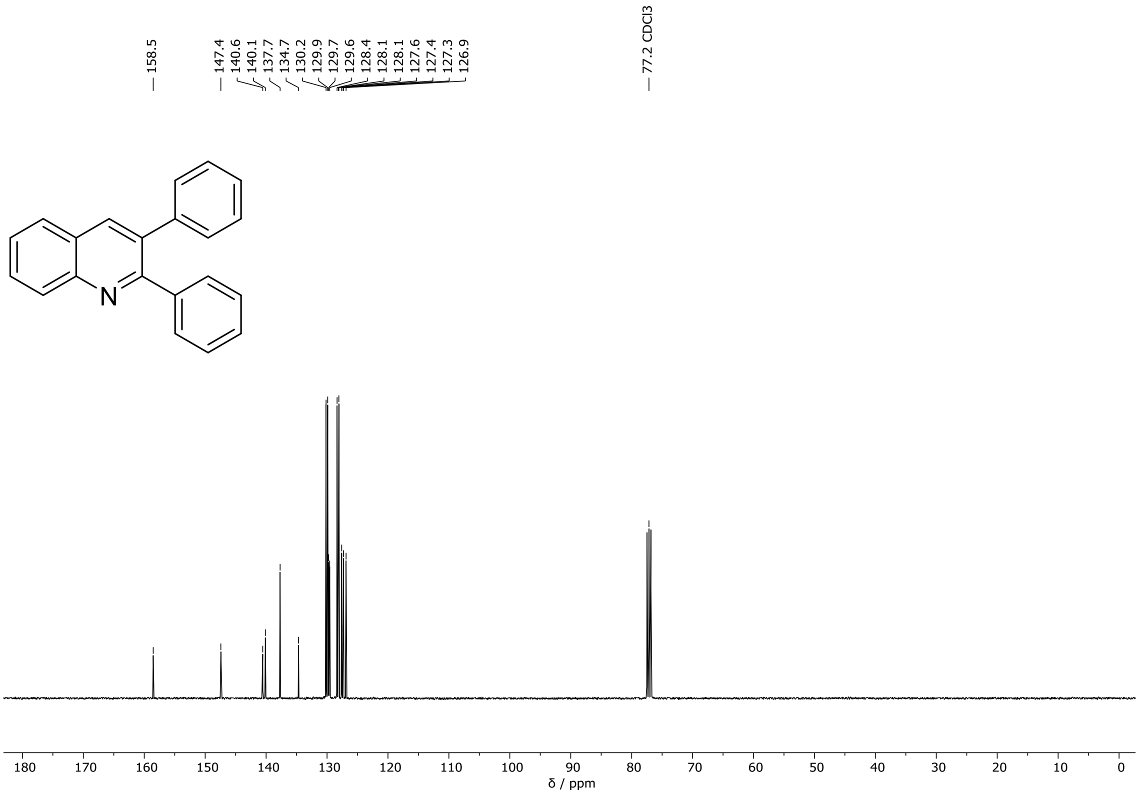

2-isopropyl-3-phenylquinoline (1aa):

^{1}H-NMR (400 MHz, $CDCl_3$)

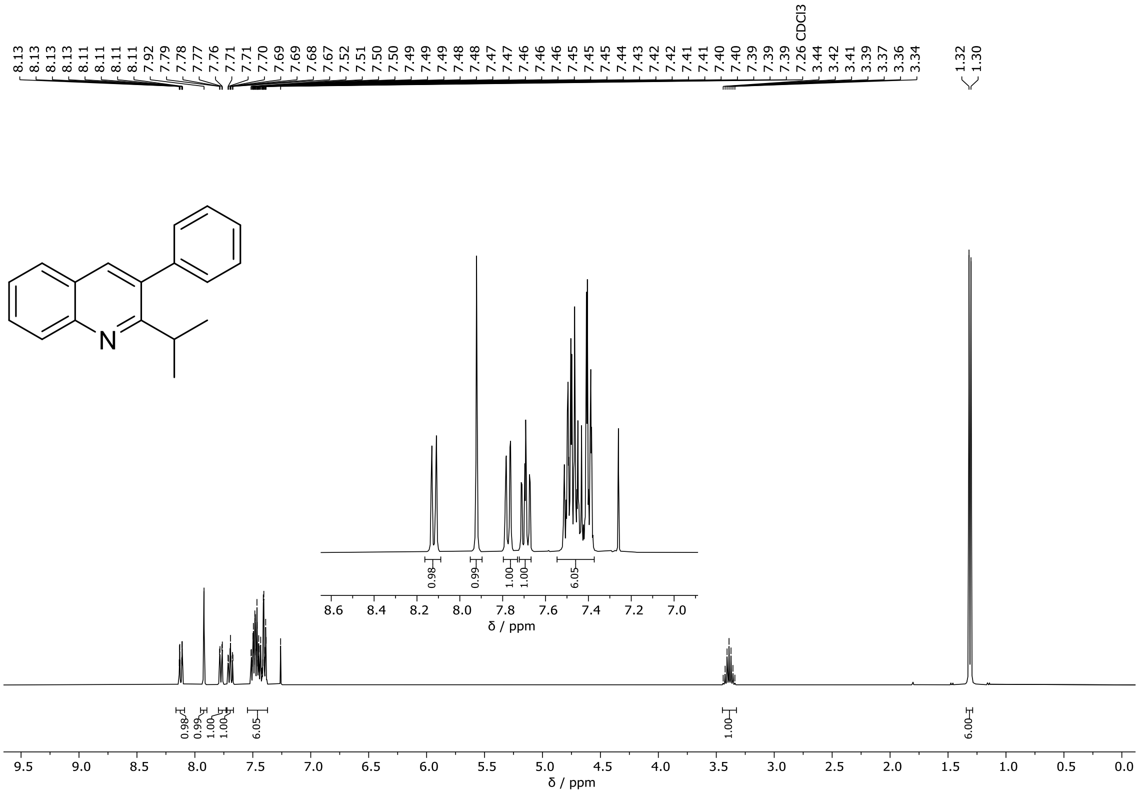

^{13}C-NMR (101 MHz, $CDCl_3$)

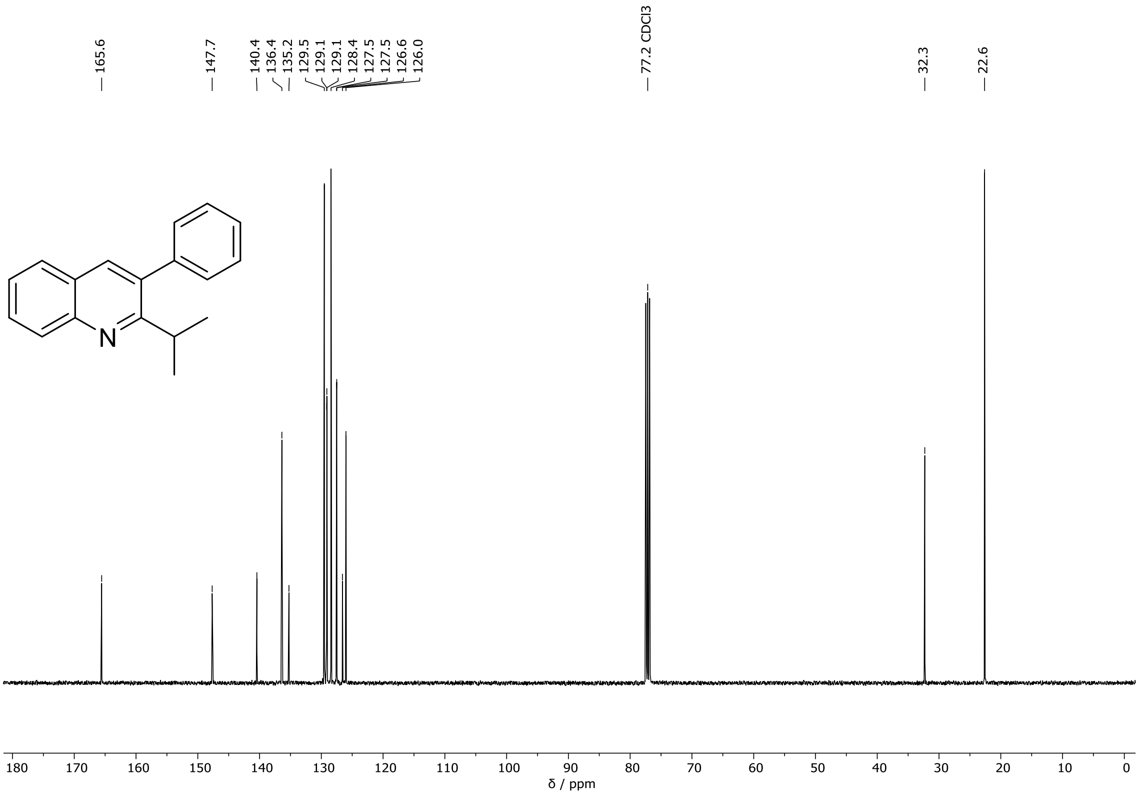

ethyl 2-(*tert*-butyl)quinoline-3-carboxylate (1ab):

^{1}H-NMR (599 MHz, $CDCl_3$)

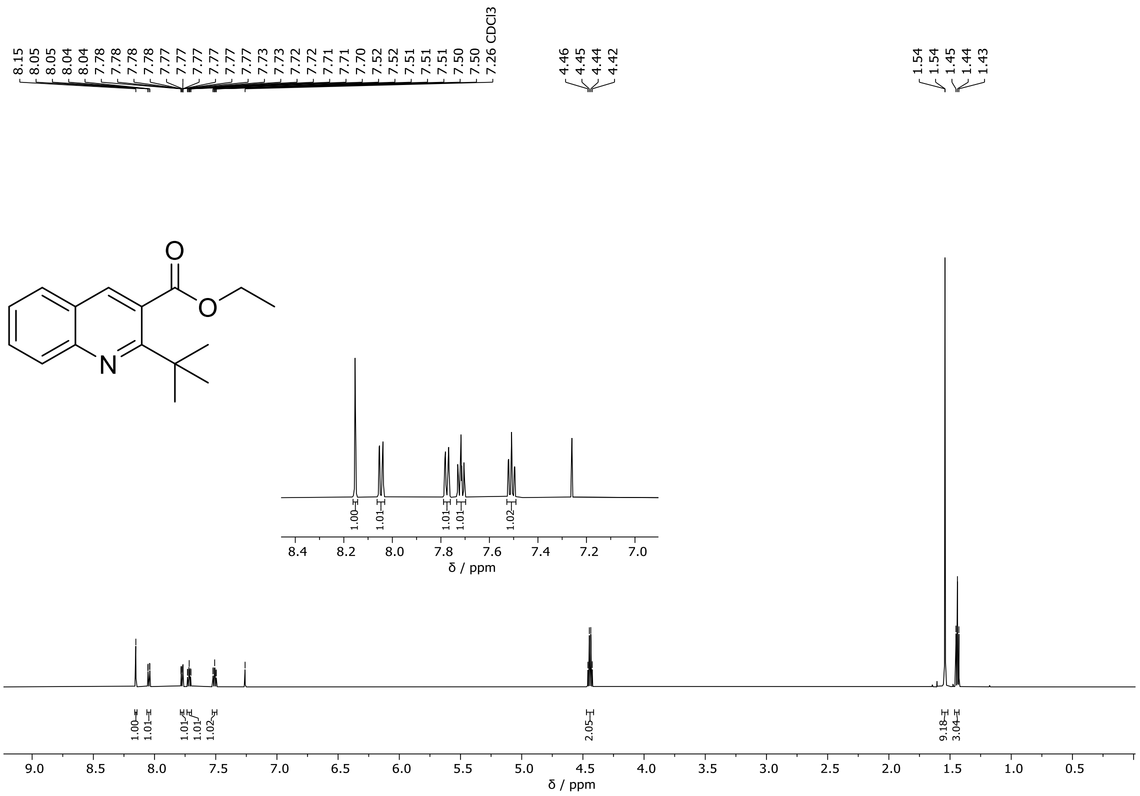

^{13}C-NMR (151 MHz, $CDCl_3$)

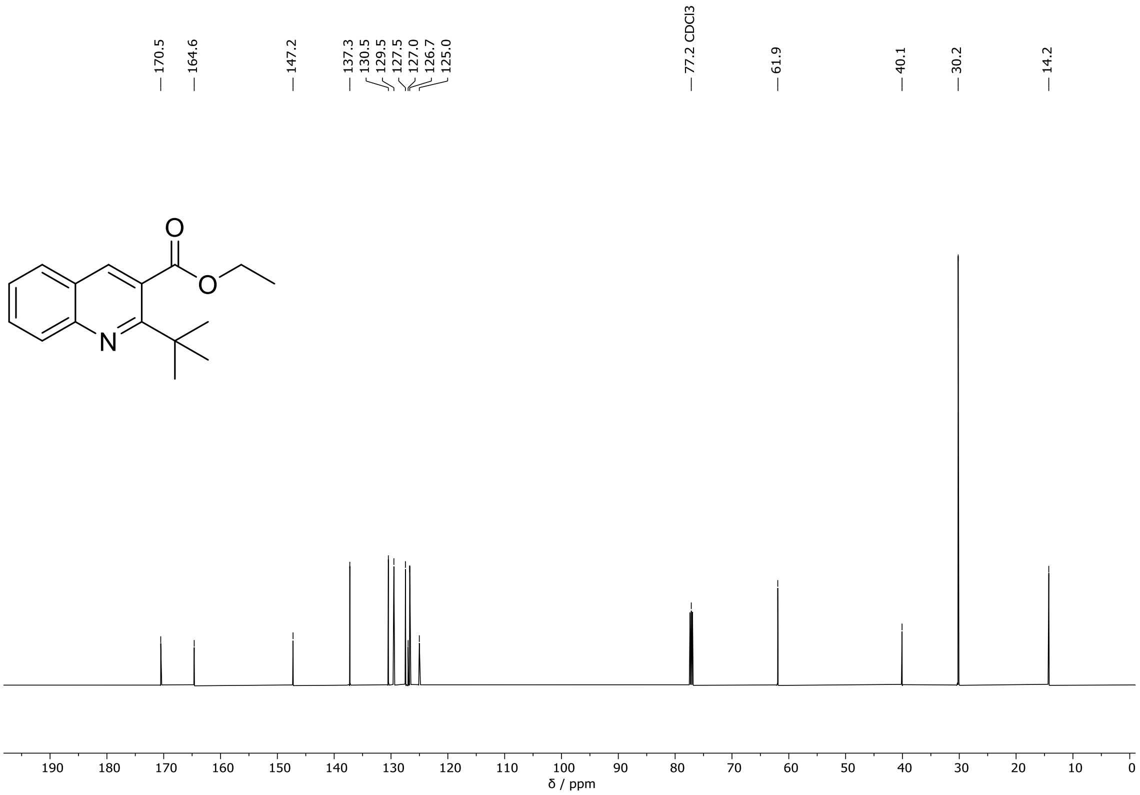

ethyl 2-isopropylquinoline-3-carboxylate (1ac):

^{1}H-NMR (300 MHz, $CDCl_3$)

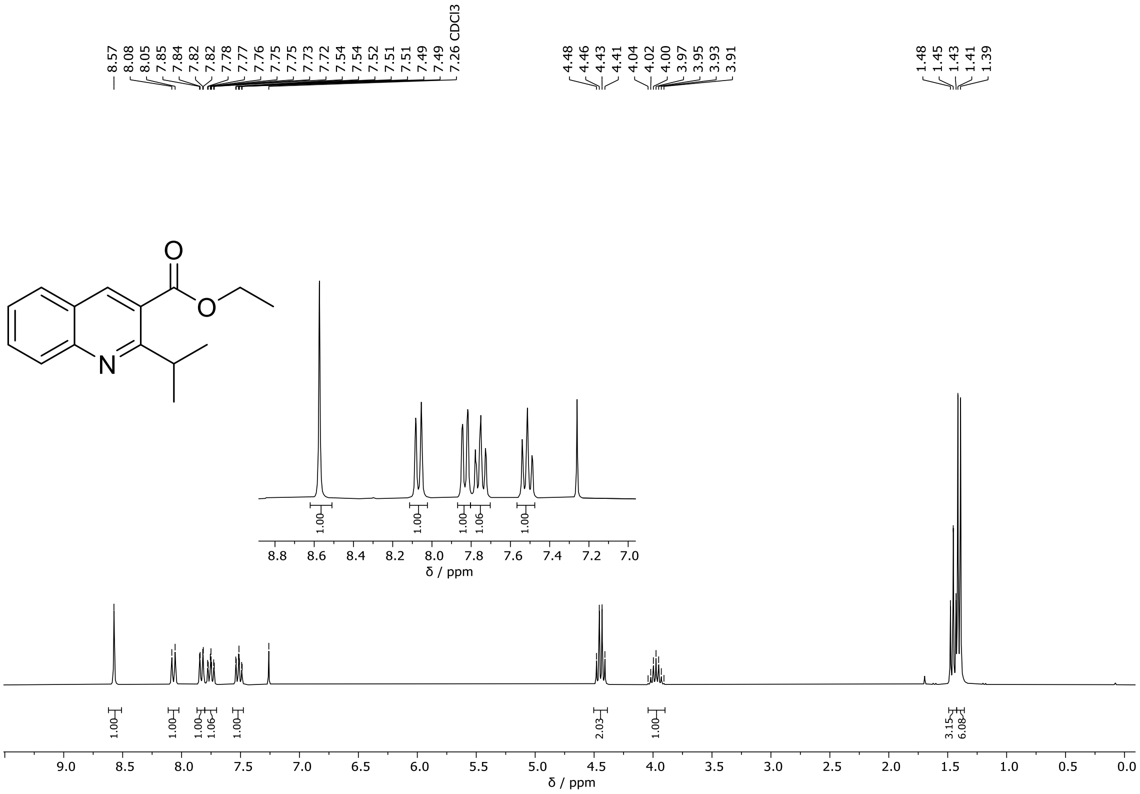

^{13}C-NMR (76 MHz, $CDCl_3$)

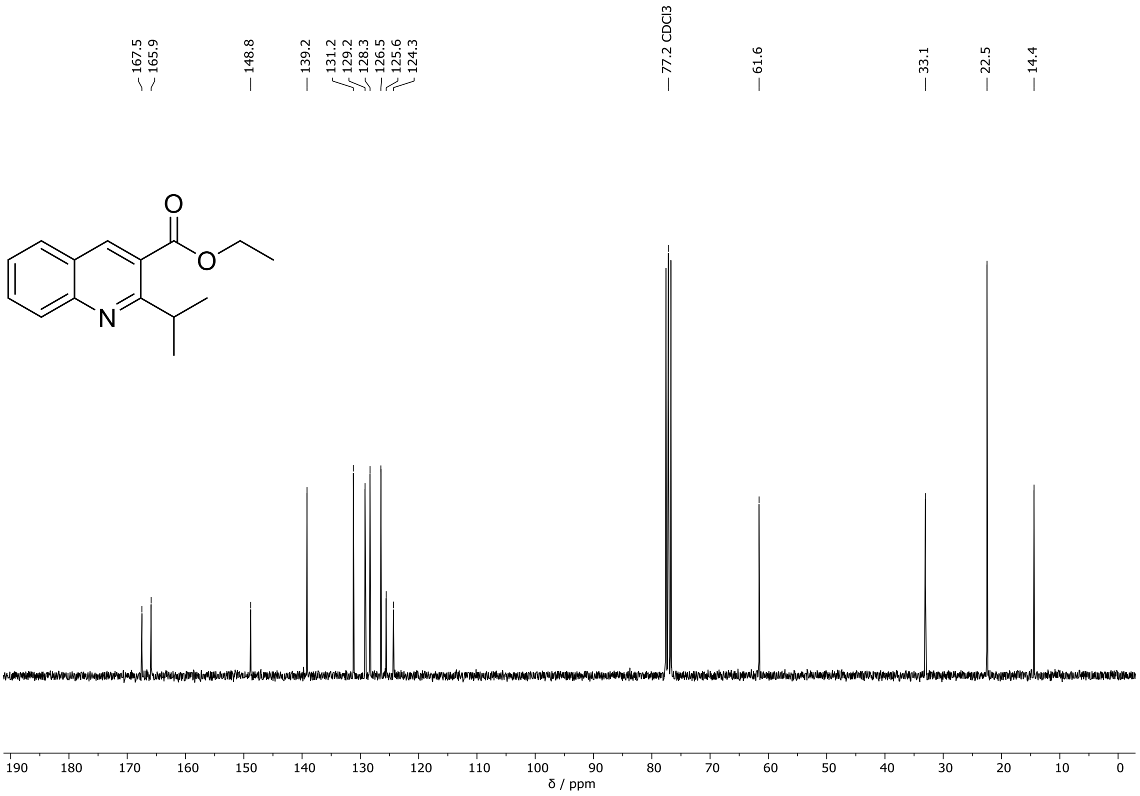

***N,N*-diethyl-2-methylquinoline-3-carboxamide (1ad):**

^{1}H-NMR (400 MHz, $CDCl_3$)

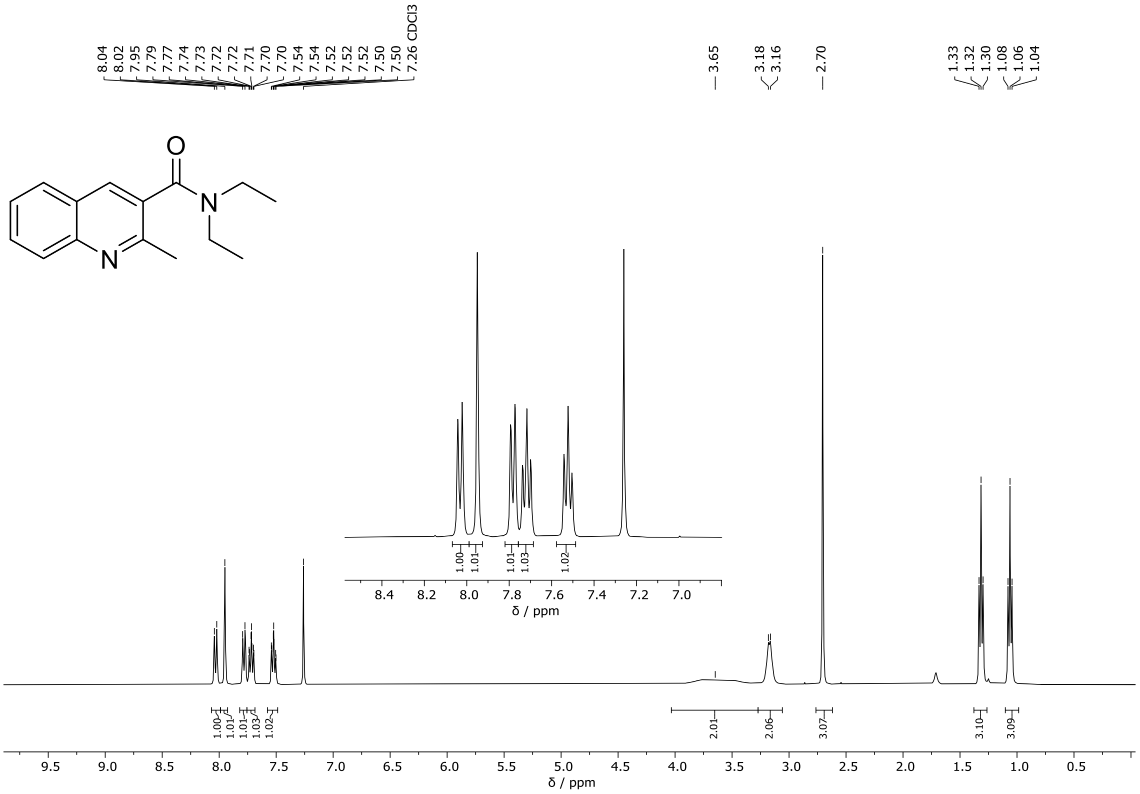

^{13}C-NMR (101 MHz, $CDCl_3$)

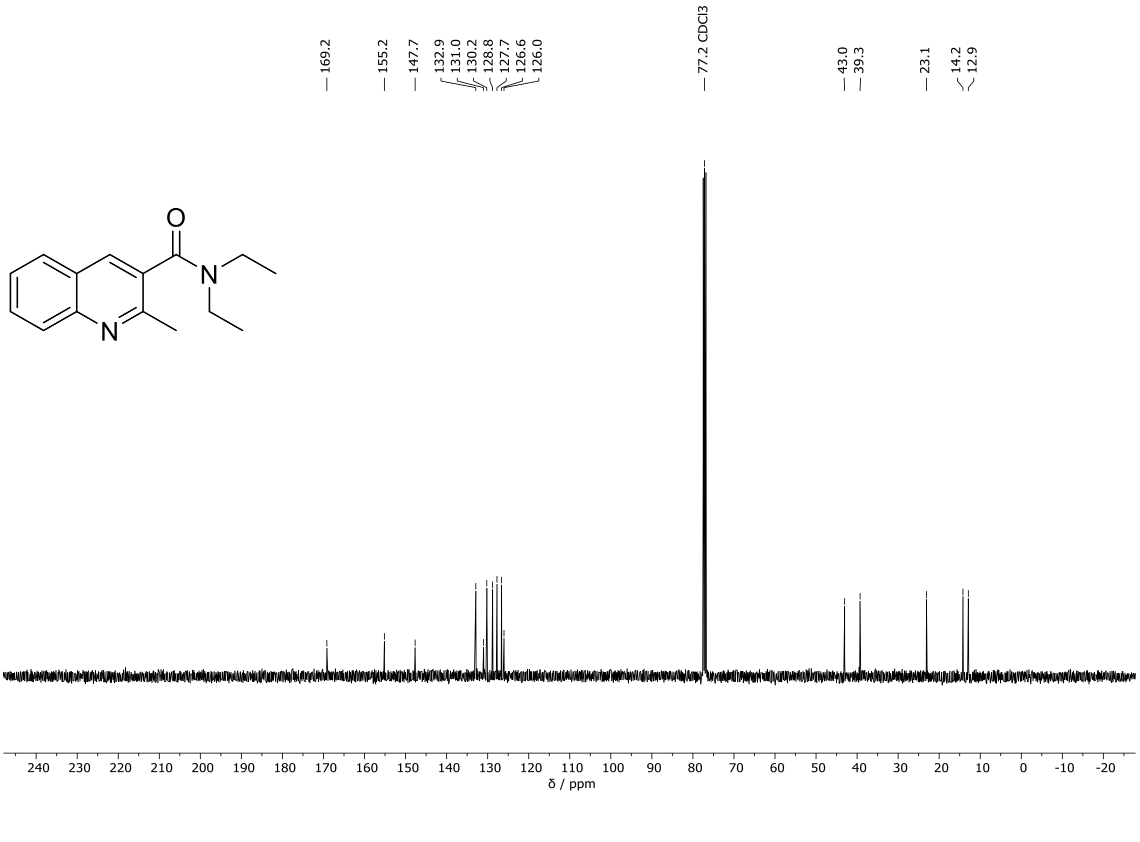

phenyl(2-(trifluoromethyl)quinolin-3-yl)methanone (1ae):

^{1}H-NMR (300 MHz, $CDCl_3$)

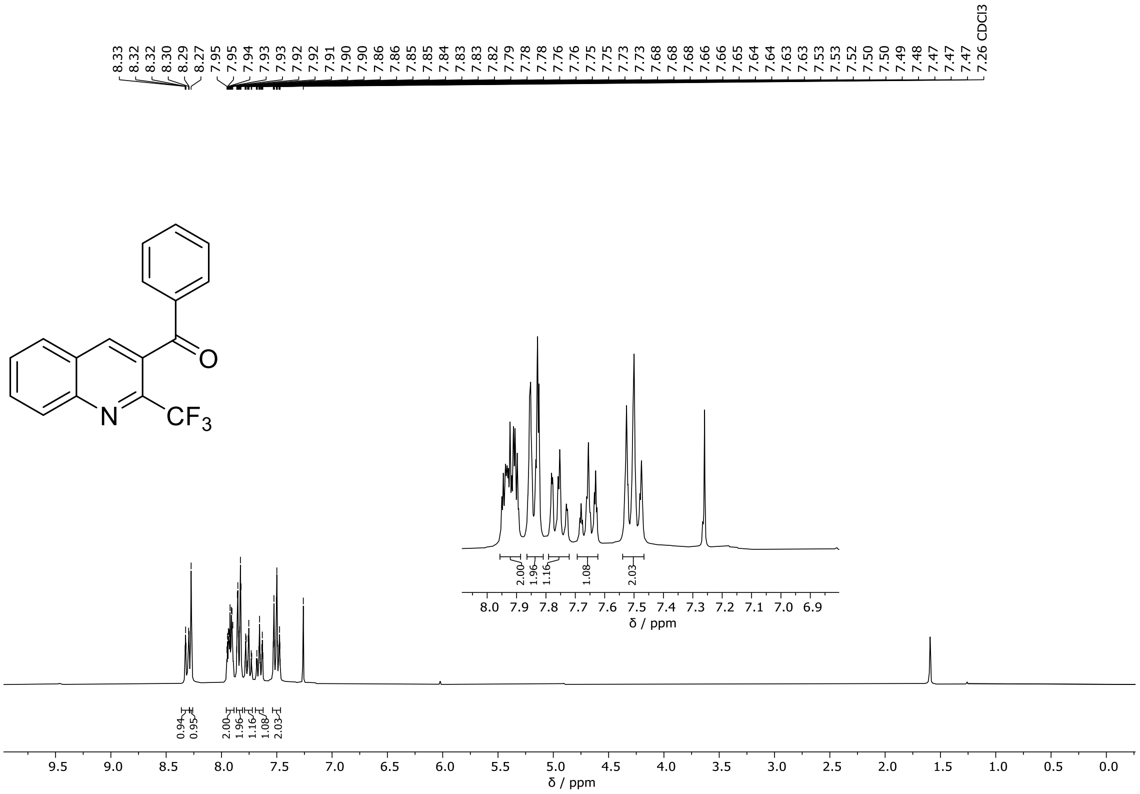

^{13}C-NMR (76 MHz, $CDCl_3$)

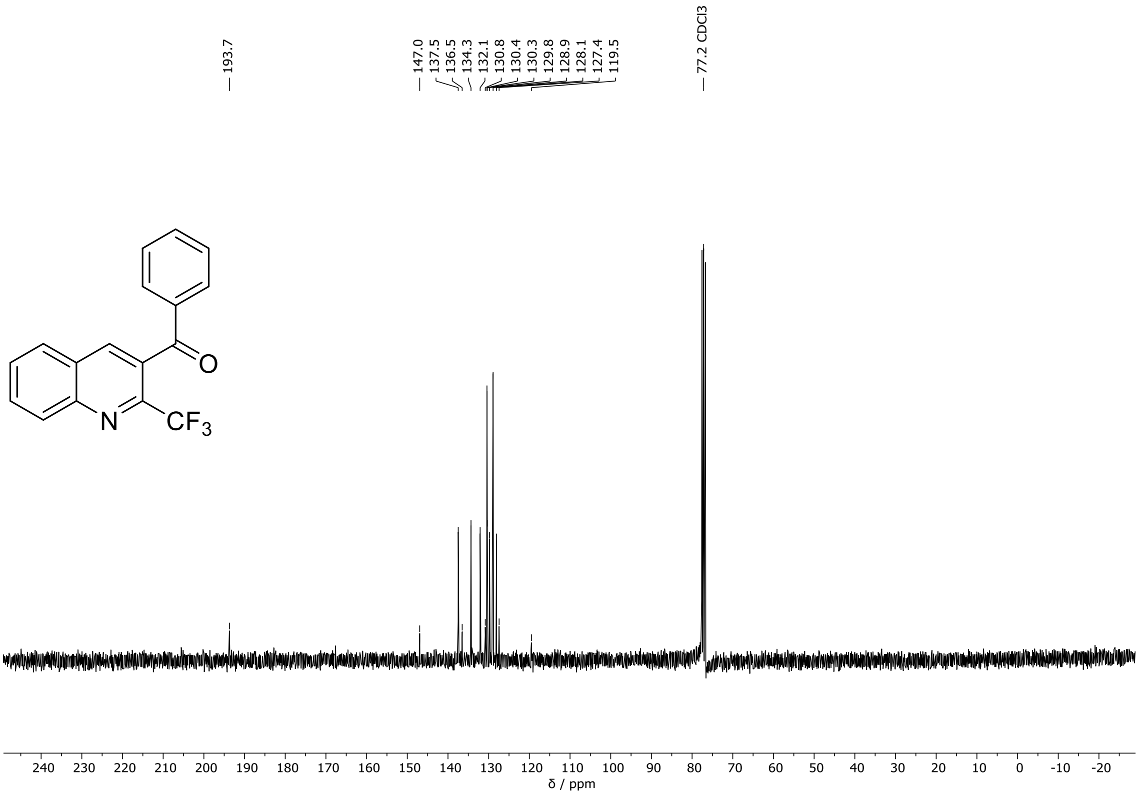

$^{19}F\{^1H\}$-NMR (282 MHz, $CDCl_3$)

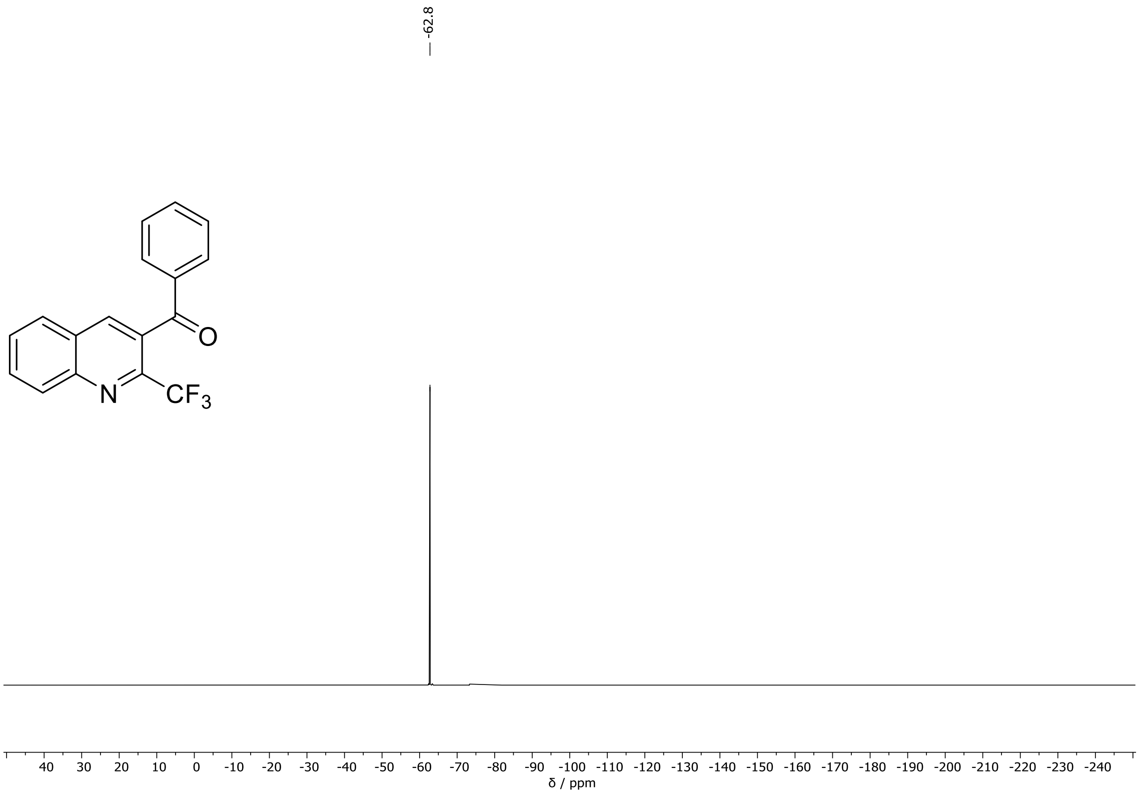

11*H*-indeno[1,2-*b*]quinoline (1af):

^{1}H-NMR (400 MHz, $CDCl_3$)

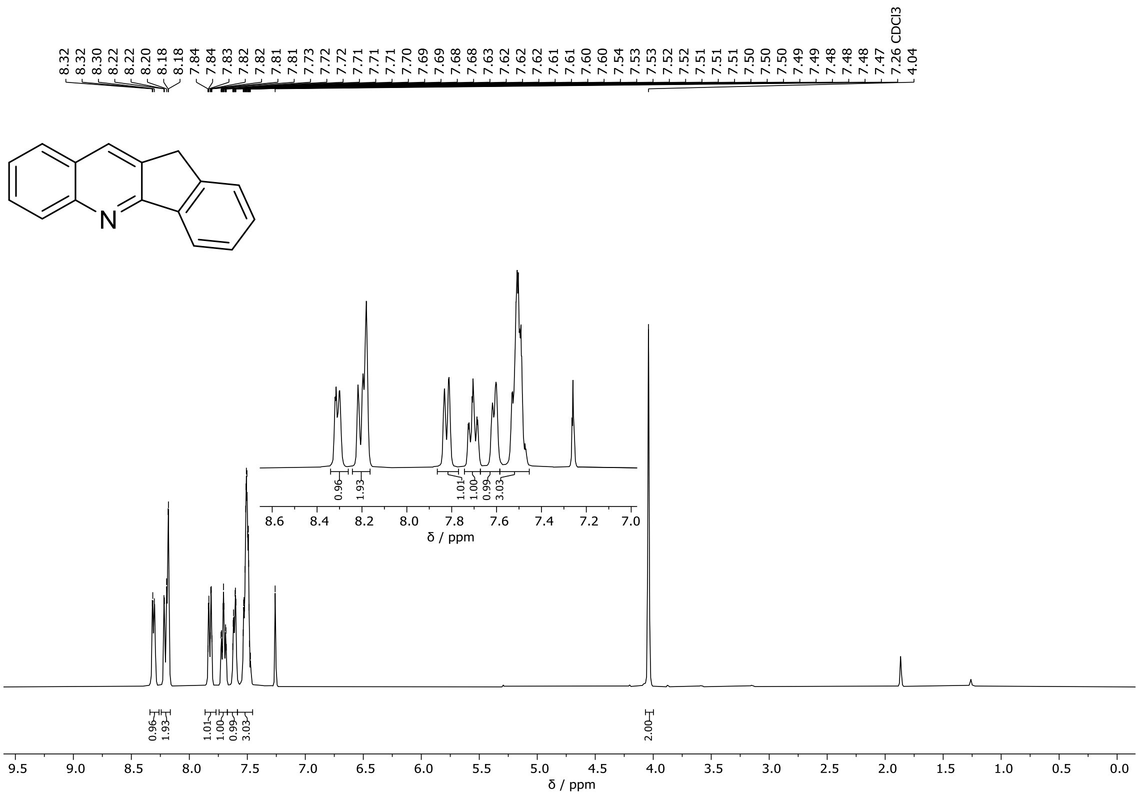

^{13}C-NMR (101 MHz, $CDCl_3$)

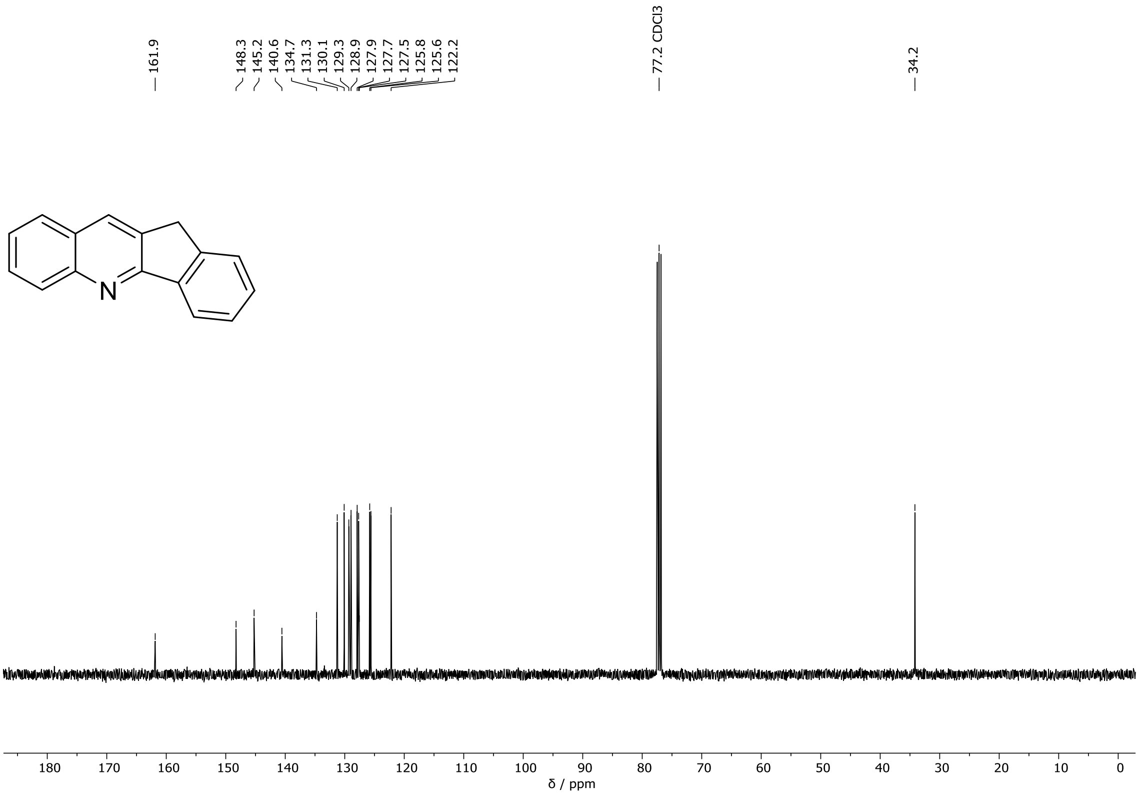

5,6-dihydrobenzo[*c*]acridine (1ag):

^{1}H-NMR (400 MHz, $CDCl_3$)

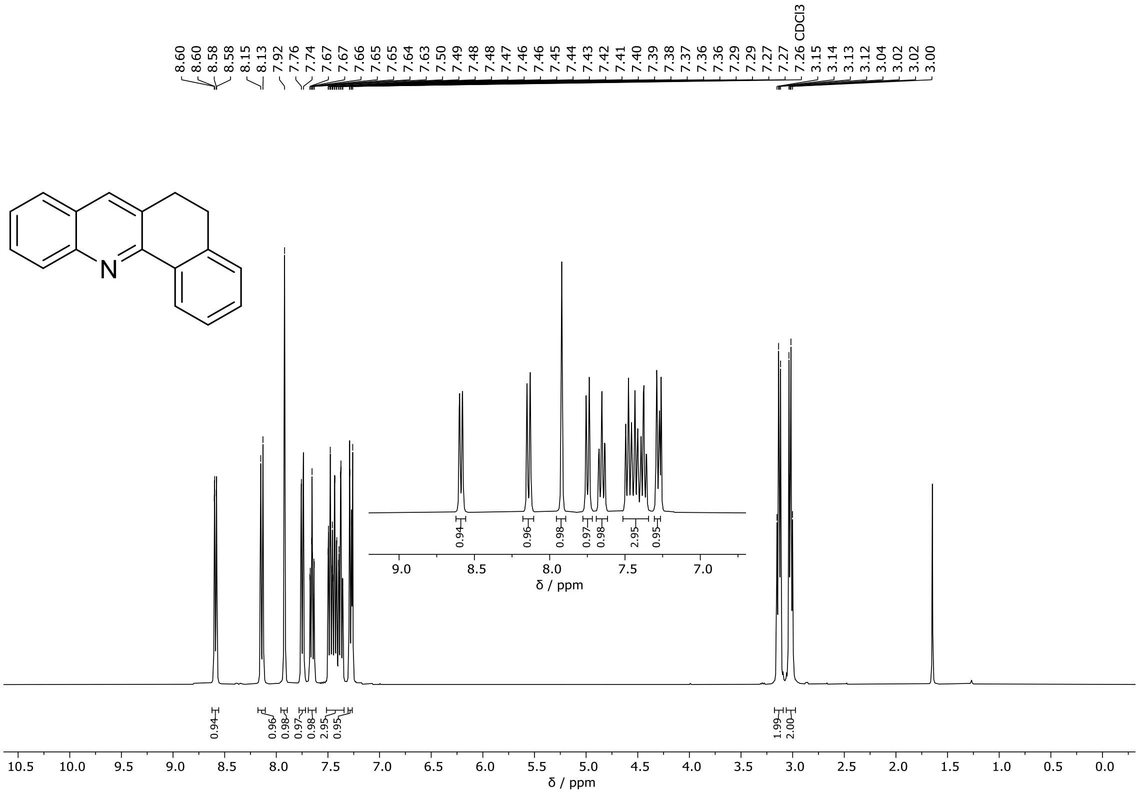

^{13}C-NMR (101 MHz, $CDCl_3$)

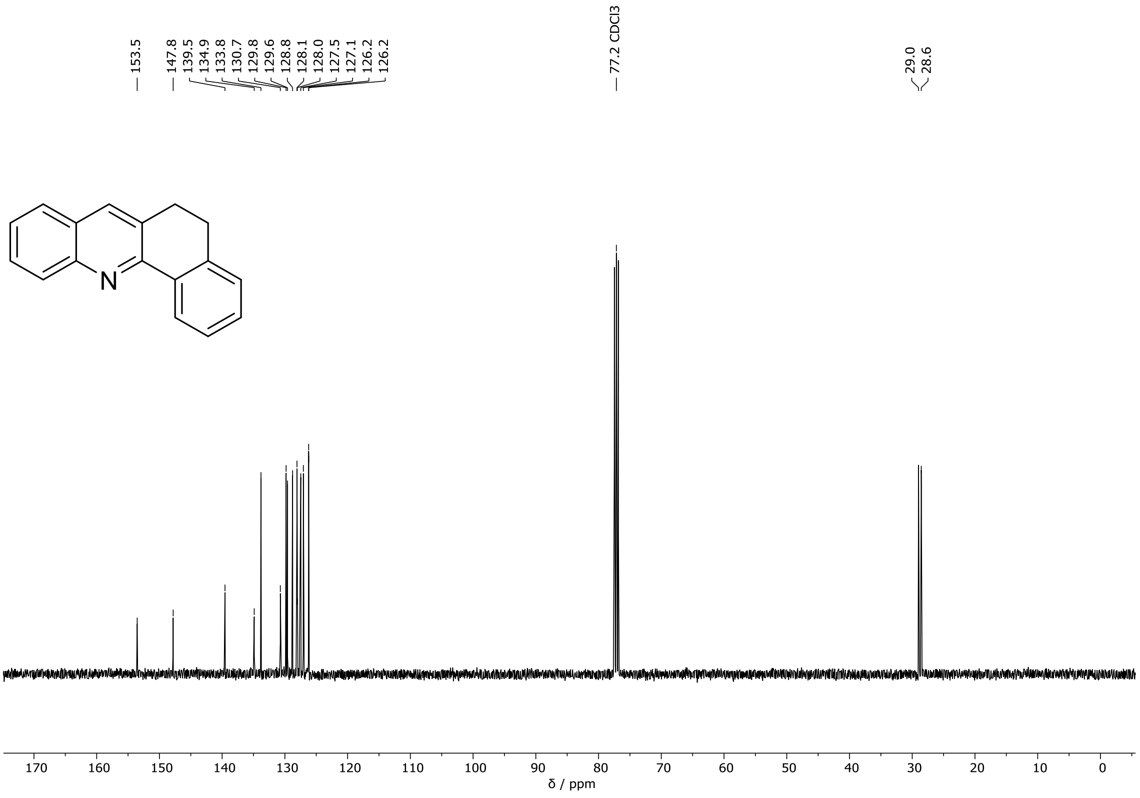

19-azatetracyclo[9.8.0.02,7.013,18]nonadeca-1(19),2,4,6,11,13,15,17-octaene (1ah):

^{1}H-NMR (400 MHz, $CDCl_3$)

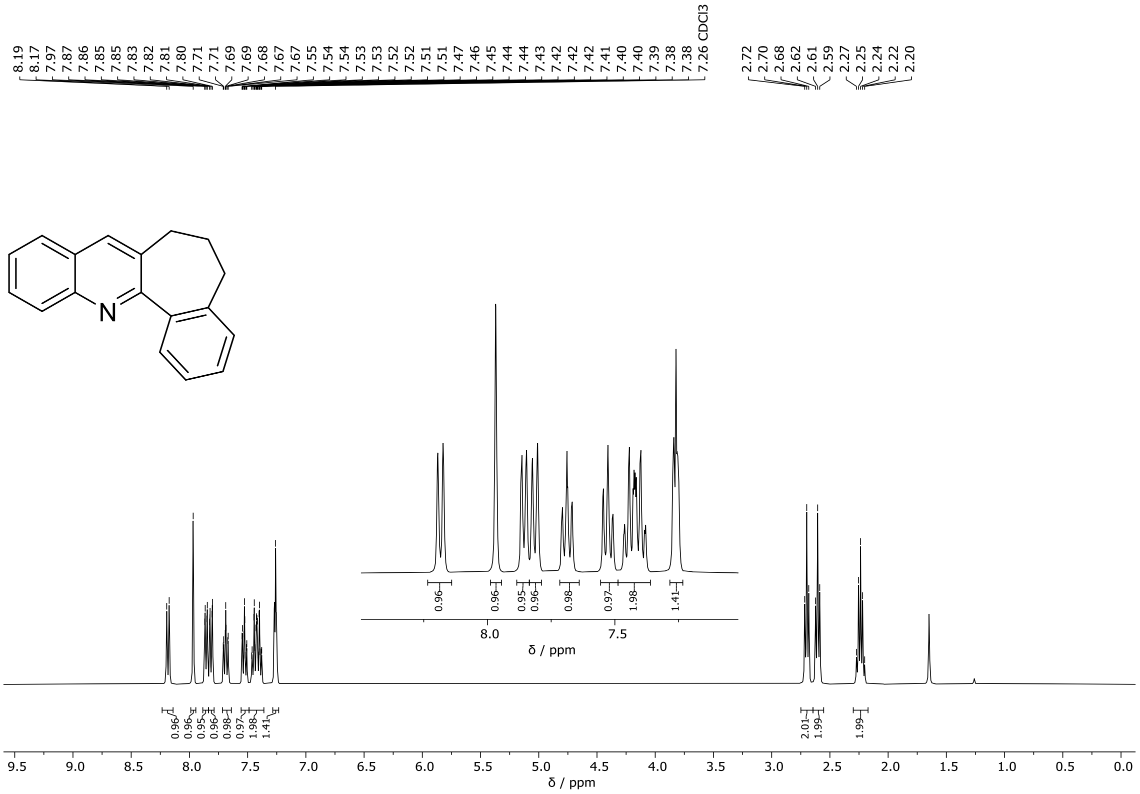

^{13}C-NMR (101 MHz, $CDCl_3$)

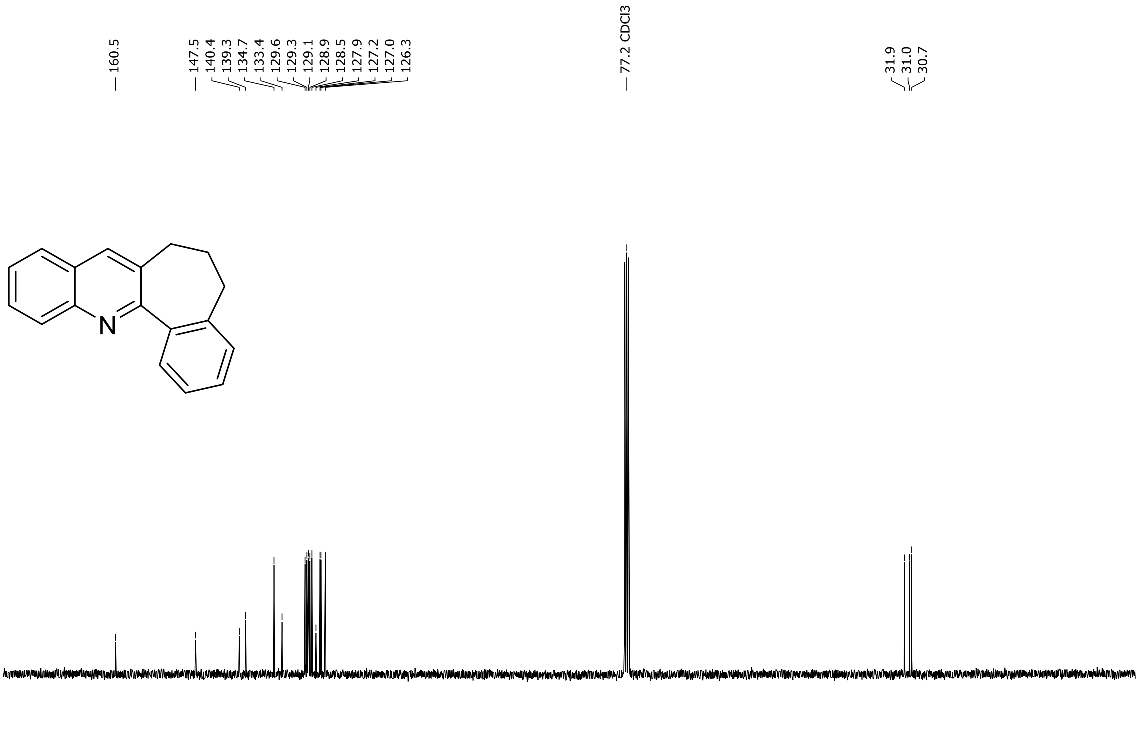

3-isopropyl-2-phenylbenzo[*g*]quinoline (1ai):

^{1}H-NMR (400 MHz, $CDCl_3$)

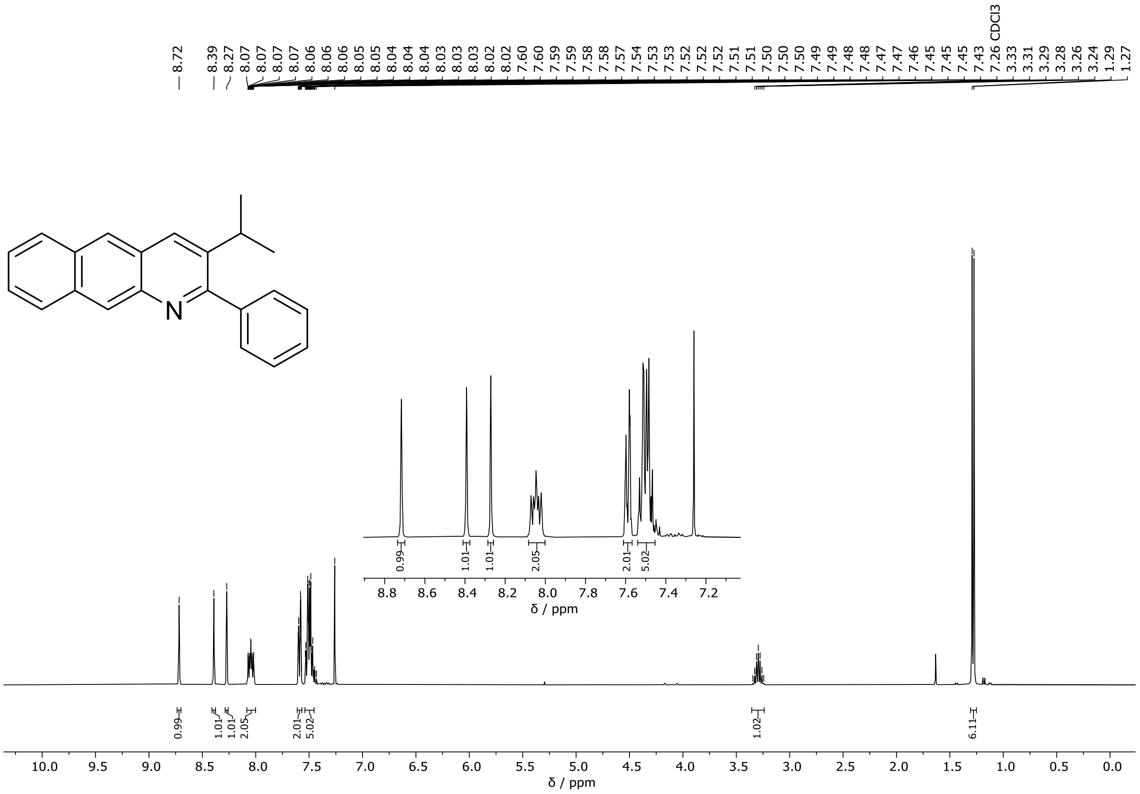

^{13}C-NMR (101 MHz, $CDCl_3$)

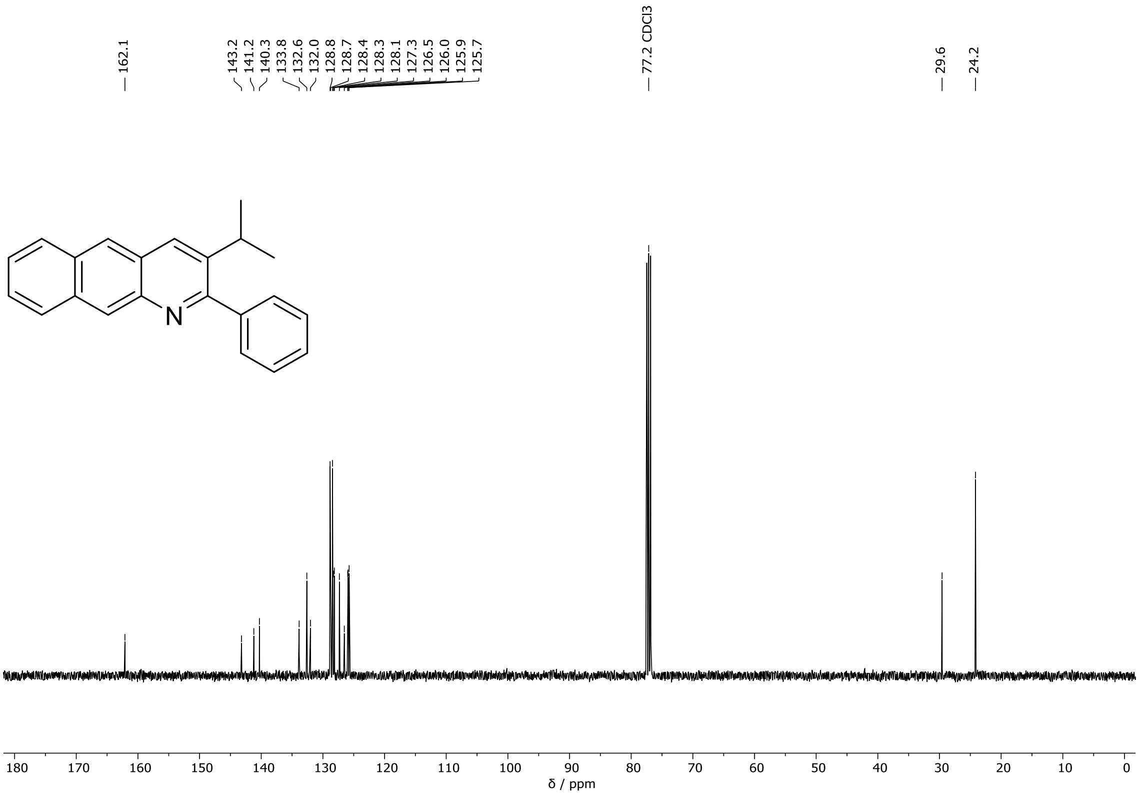

6-methoxy-3-methyl-2-phenylquinoline (1aj):

^{1}H-NMR (400 MHz, $CDCl_3$)

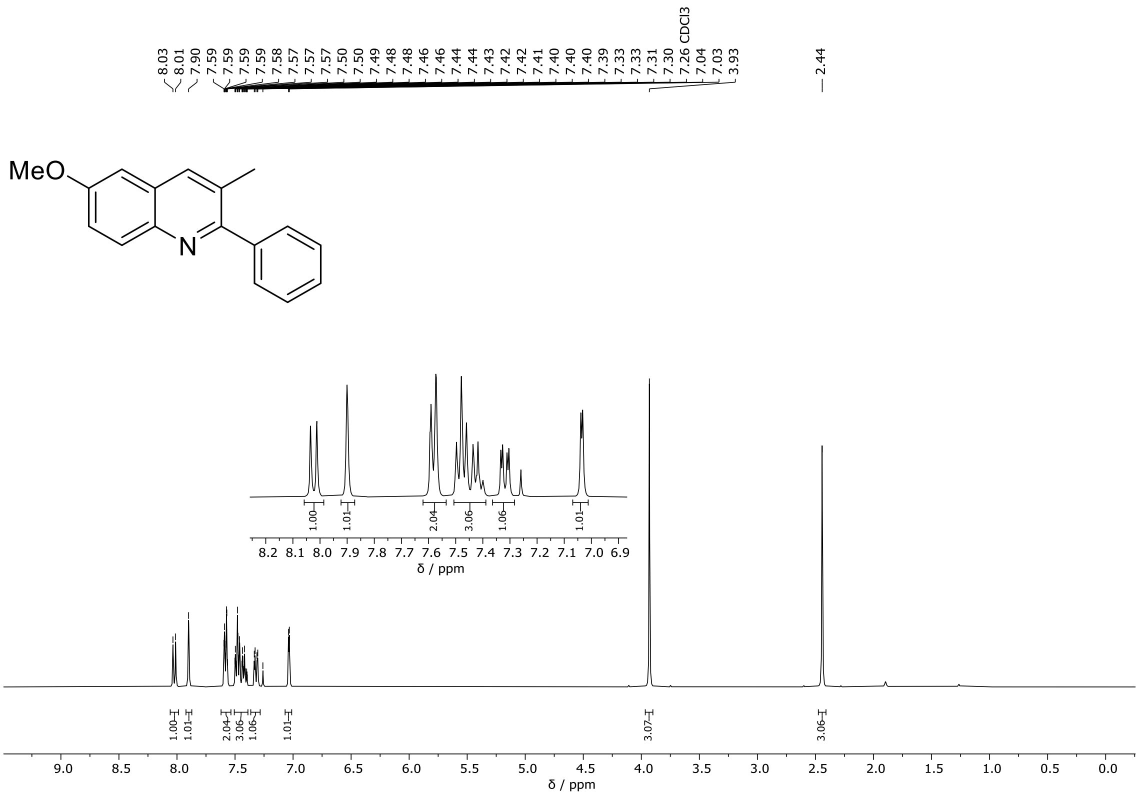

^{13}C-NMR (101 MHz, $CDCl_3$)

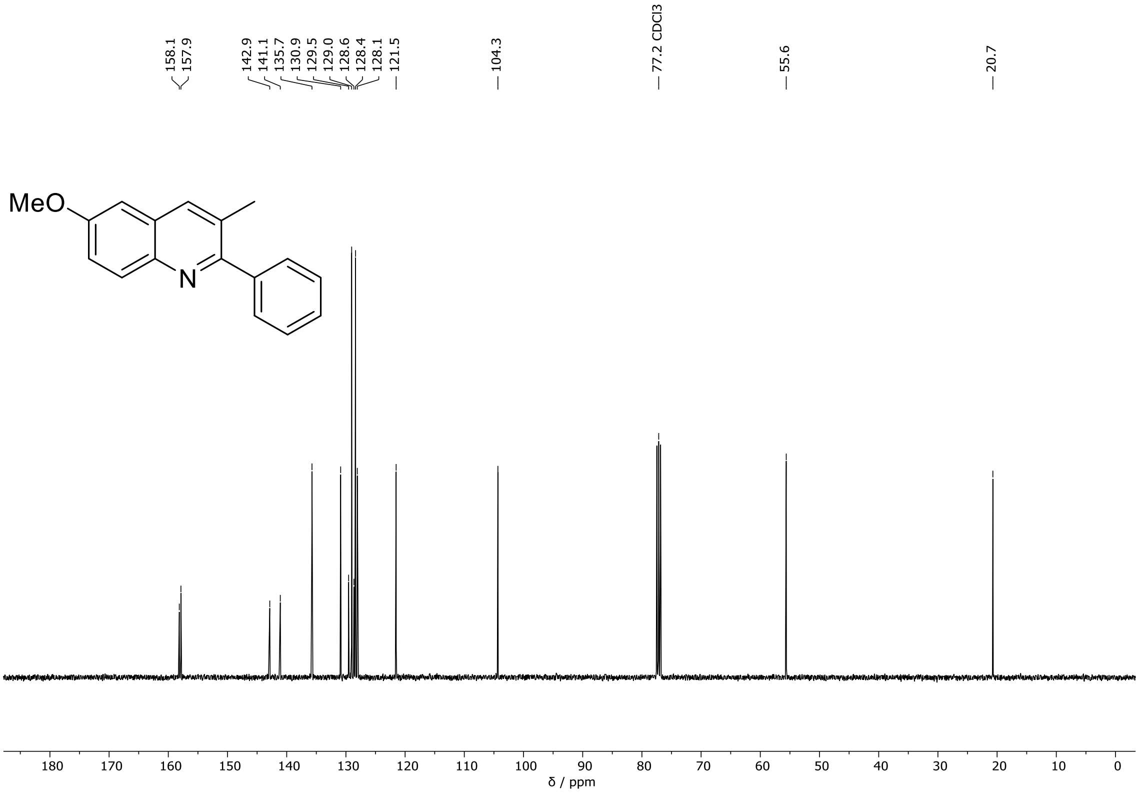

3-(benzenesulfonyl)-2-phenylquinoline (1ak):

^{1}H-NMR (400 MHz, $CDCl_3$)

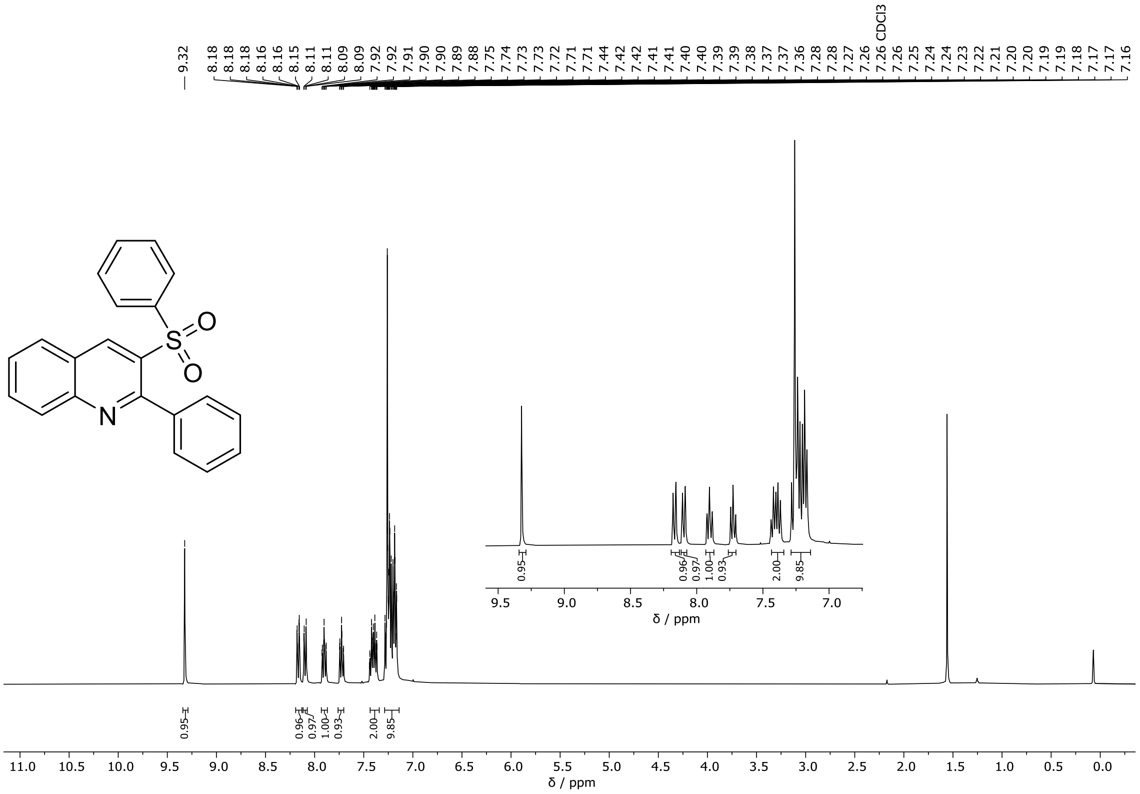

^{13}C-NMR (101 MHz, $CDCl_3$)

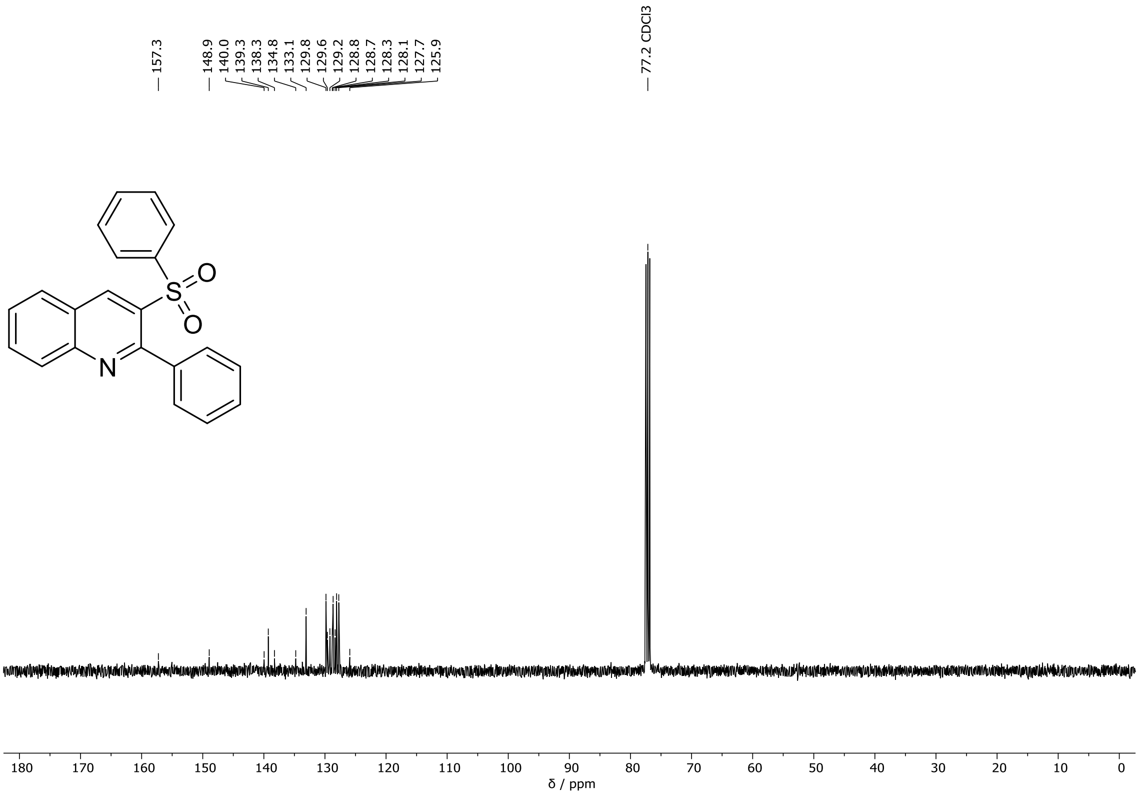

3-cyclopentylidene-2,3-dihydro-1*H*-cyclopenta[*b*]quinoline (1al):

^{1}H-NMR (599 MHz, $CDCl_3$)

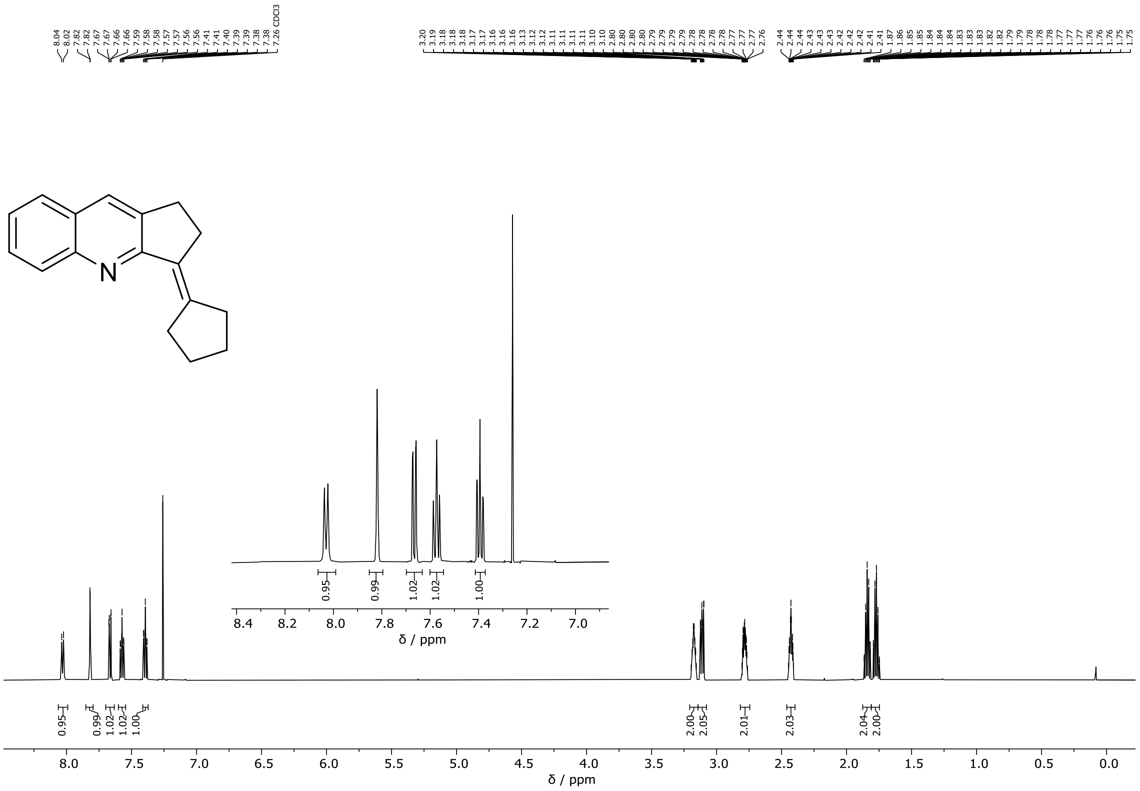

^{13}C-NMR (151 MHz, $CDCl_3$)

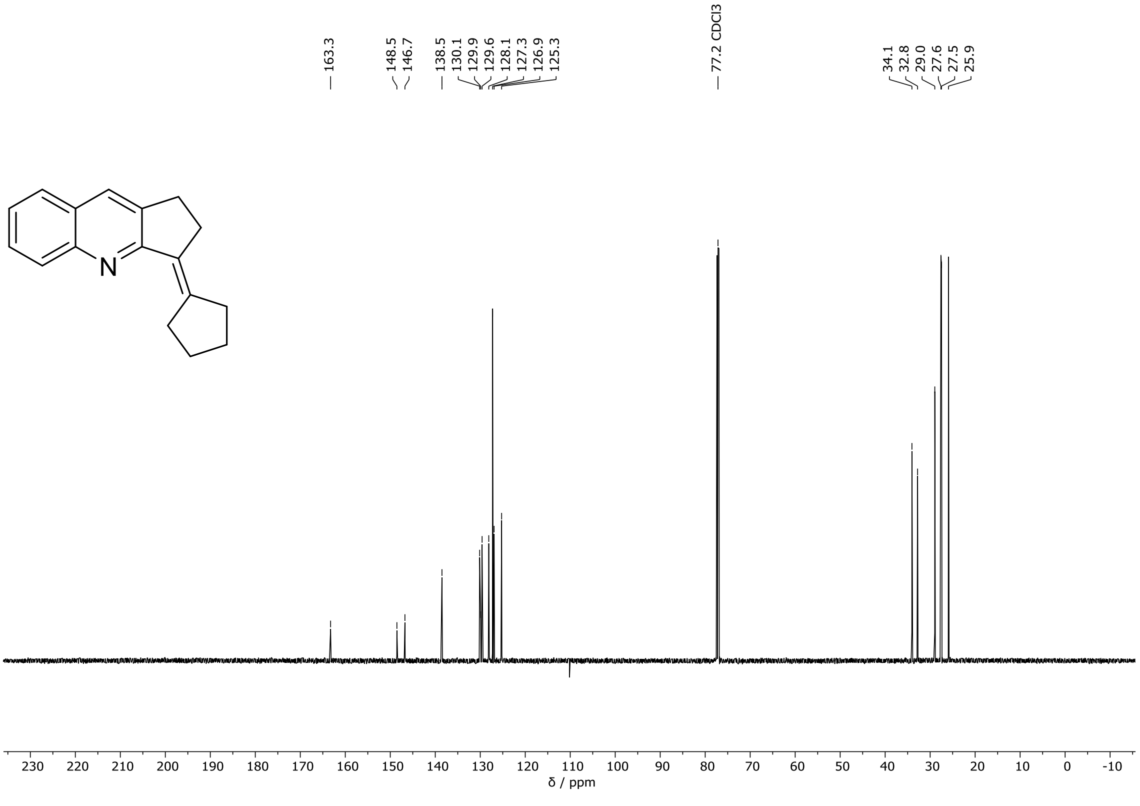

7.2. NMR Spectra of Indole Products

3-isobutyl-2-phenyl-1*H*-indole (2a):

^{1}H-NMR (599 MHz, $CDCl_3$)

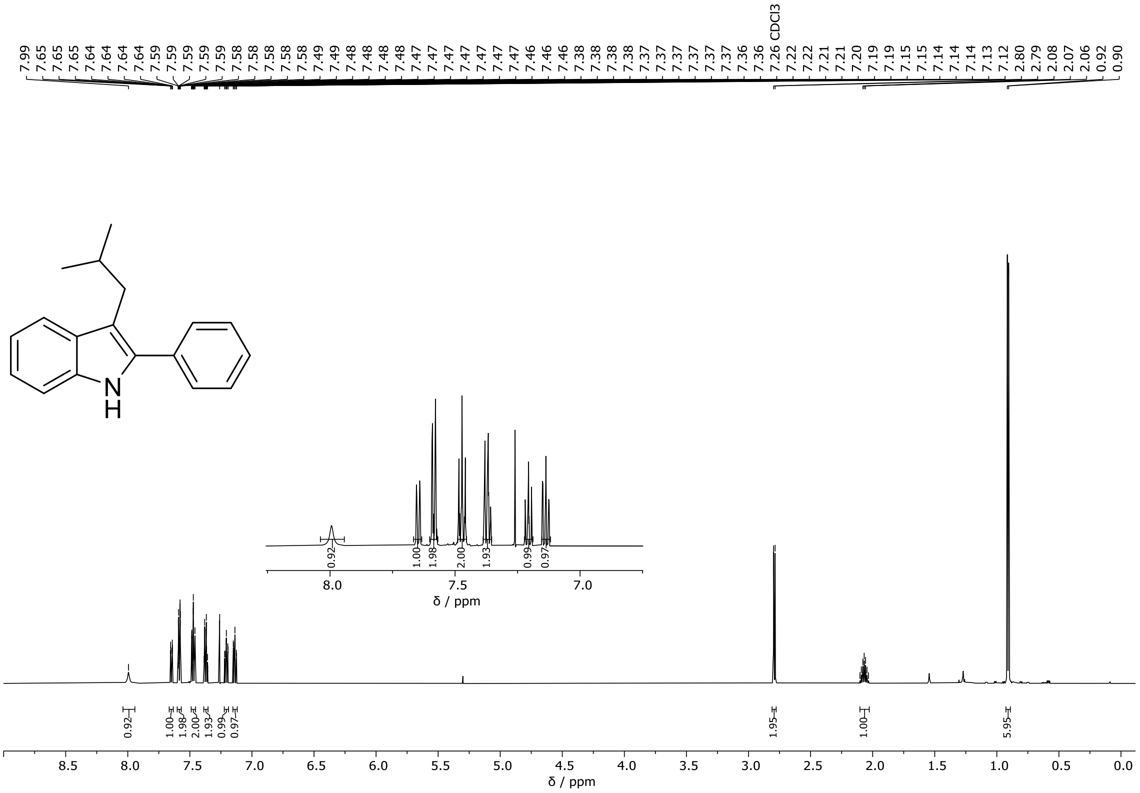

^{13}C-NMR (151 MHz, $CDCl_3$)

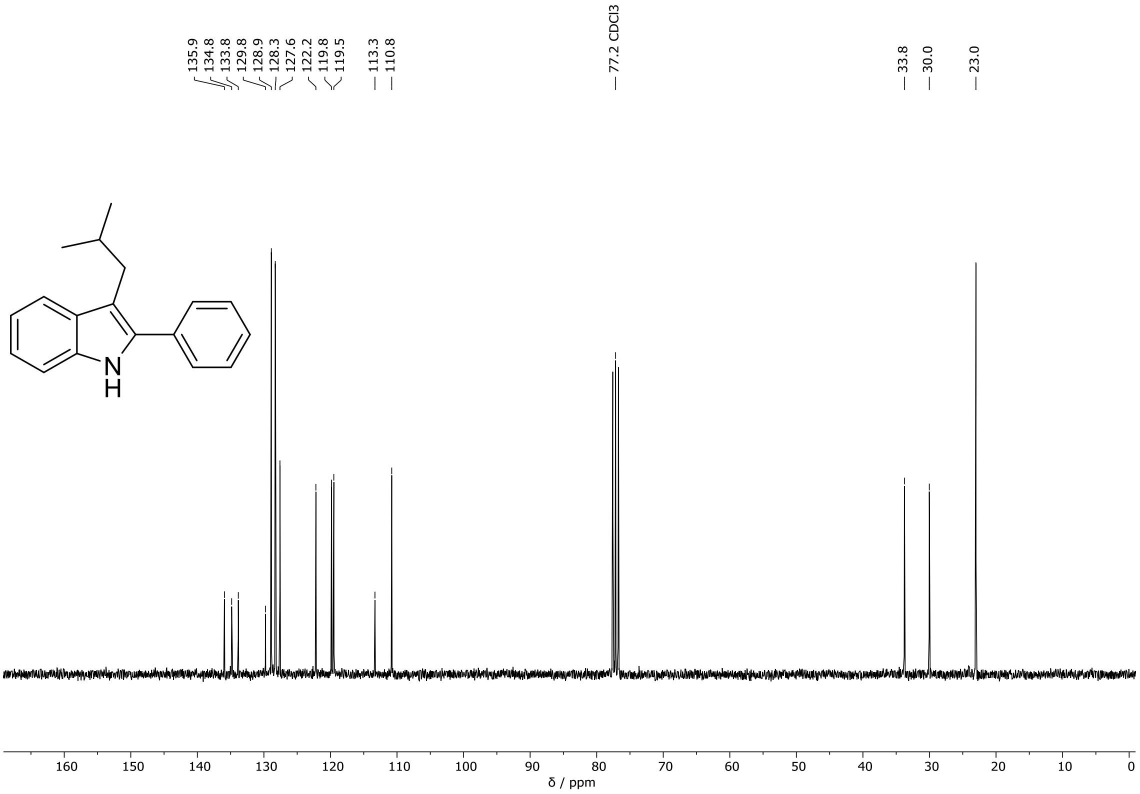

3-heptyl-2-phenyl-1*H*-indole (2b):

^{1}H-NMR (300 MHz, $CDCl_3$)

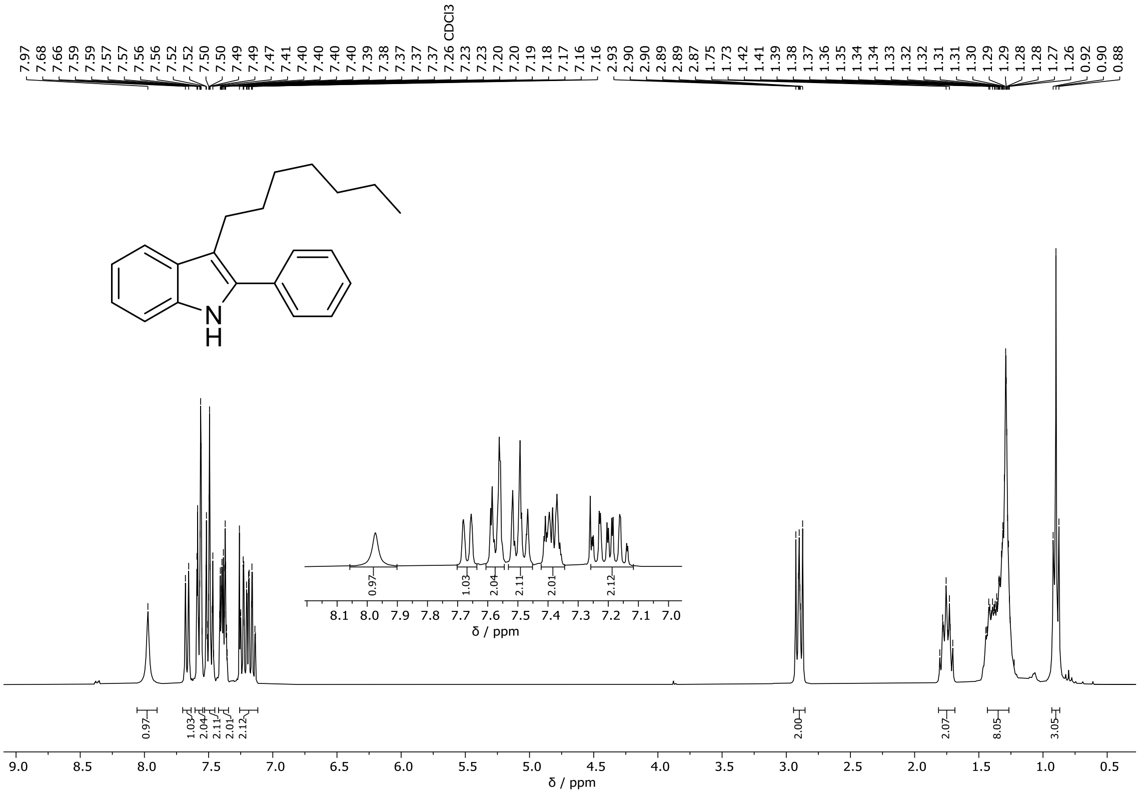

^{13}C-NMR (101 MHz, $CDCl_3$)

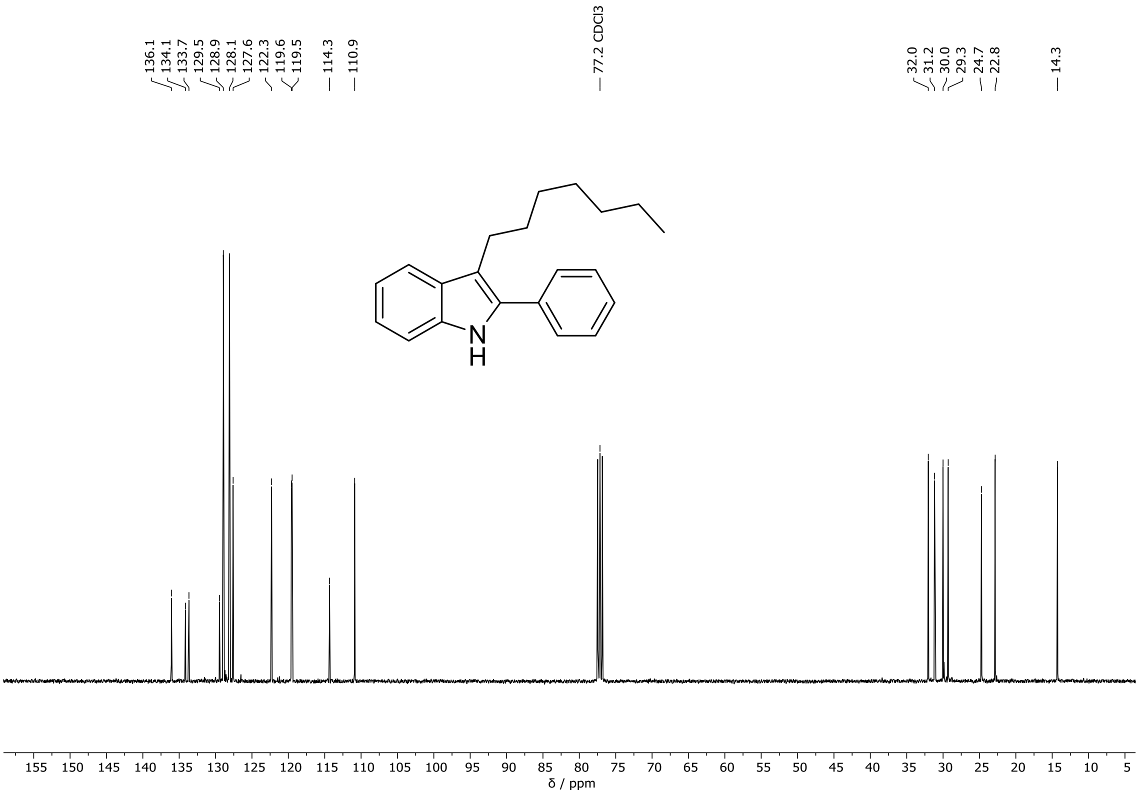

2-phenyl-3-propyl-1*H*-indole (2c):

^{1}H-NMR (400 MHz, $CDCl_3$)

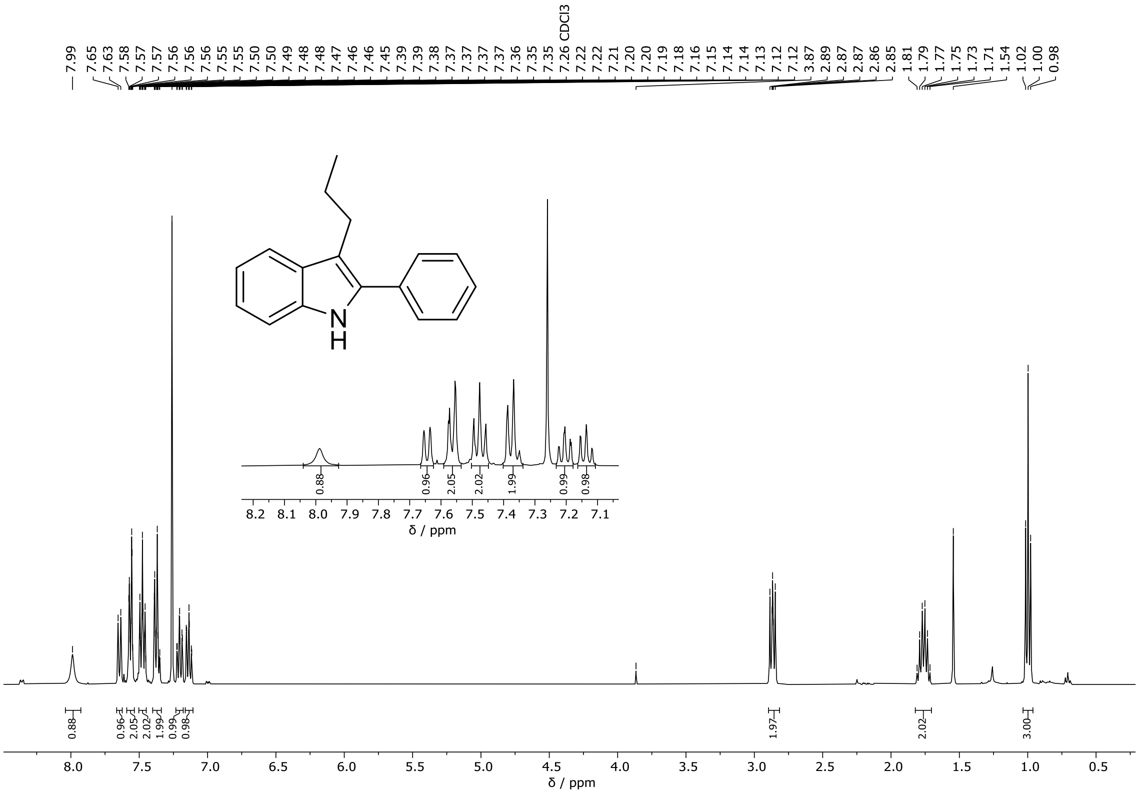

^{13}C-NMR (101 MHz, $CDCl_3$)

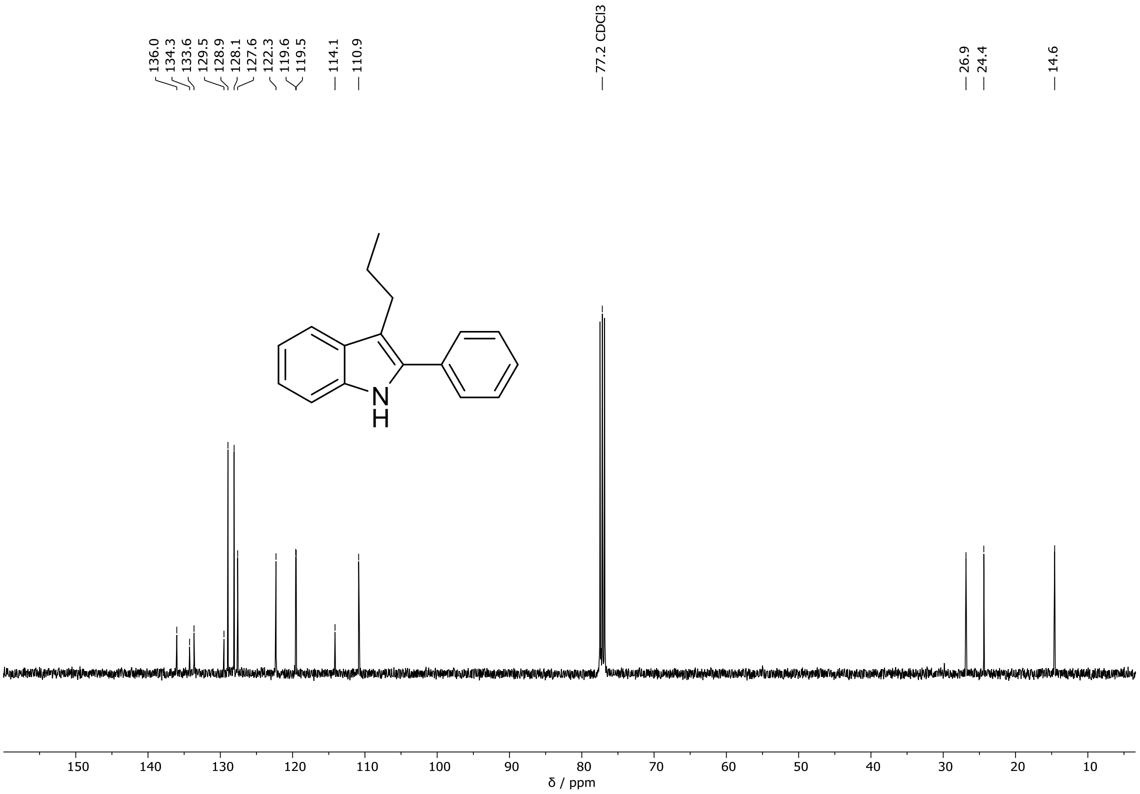

3-methyl-2-phenyl-1*H*-indole (2d):

^{1}H-NMR (400 MHz, $CDCl_3$)

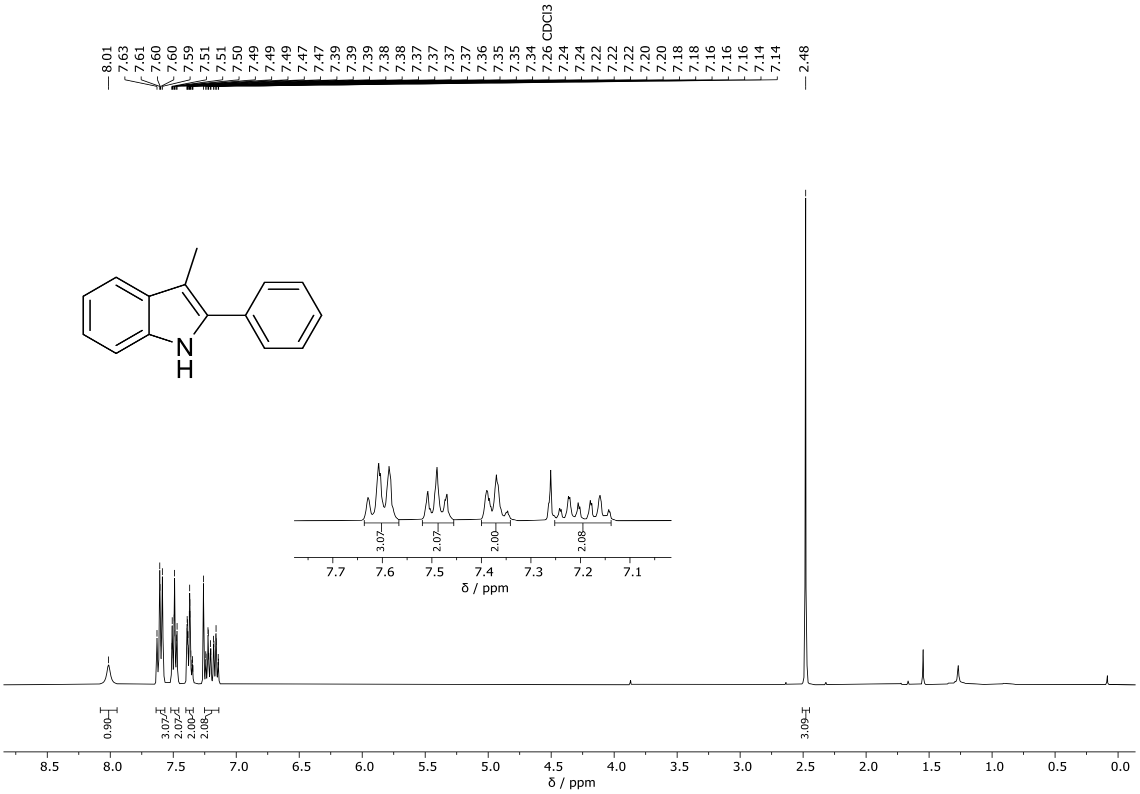

^{13}C-NMR (101 MHz, $CDCl_3$)

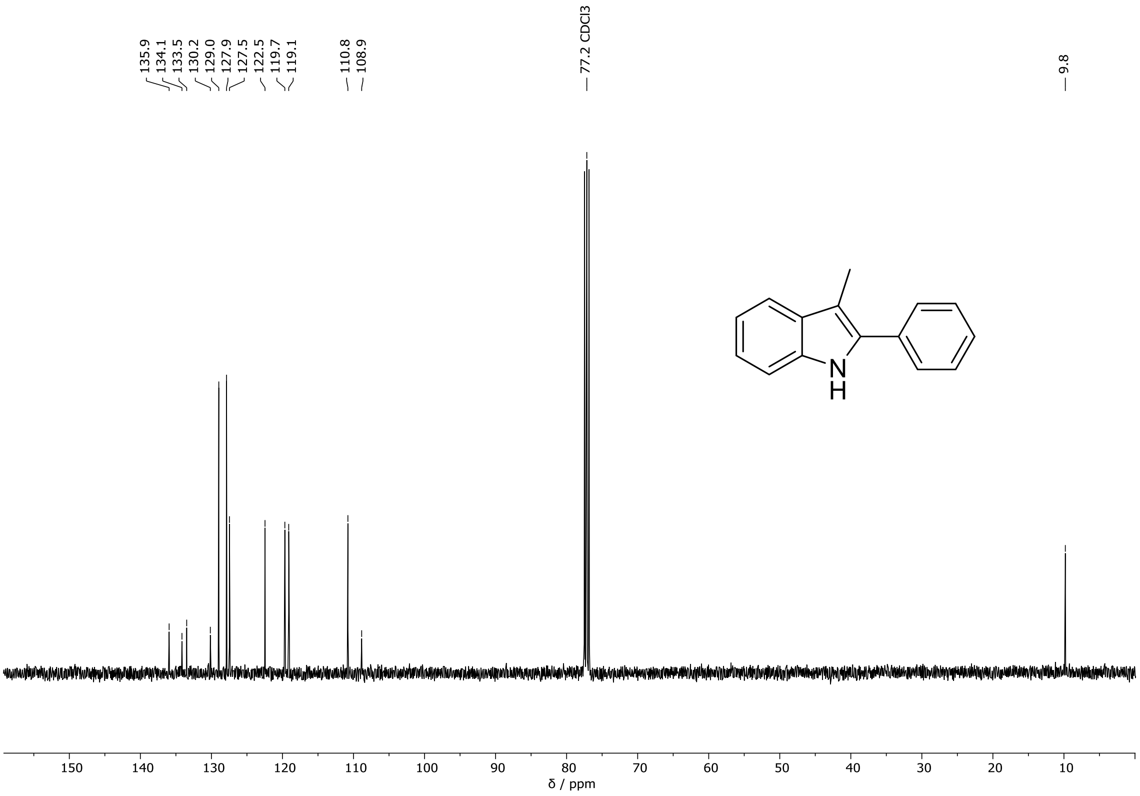

3-isobutyl-2-(4-(trifluoromethyl)phenyl)-1*H*-indole (2e):

^{1}H-NMR (500 MHz, $CDCl_3$)

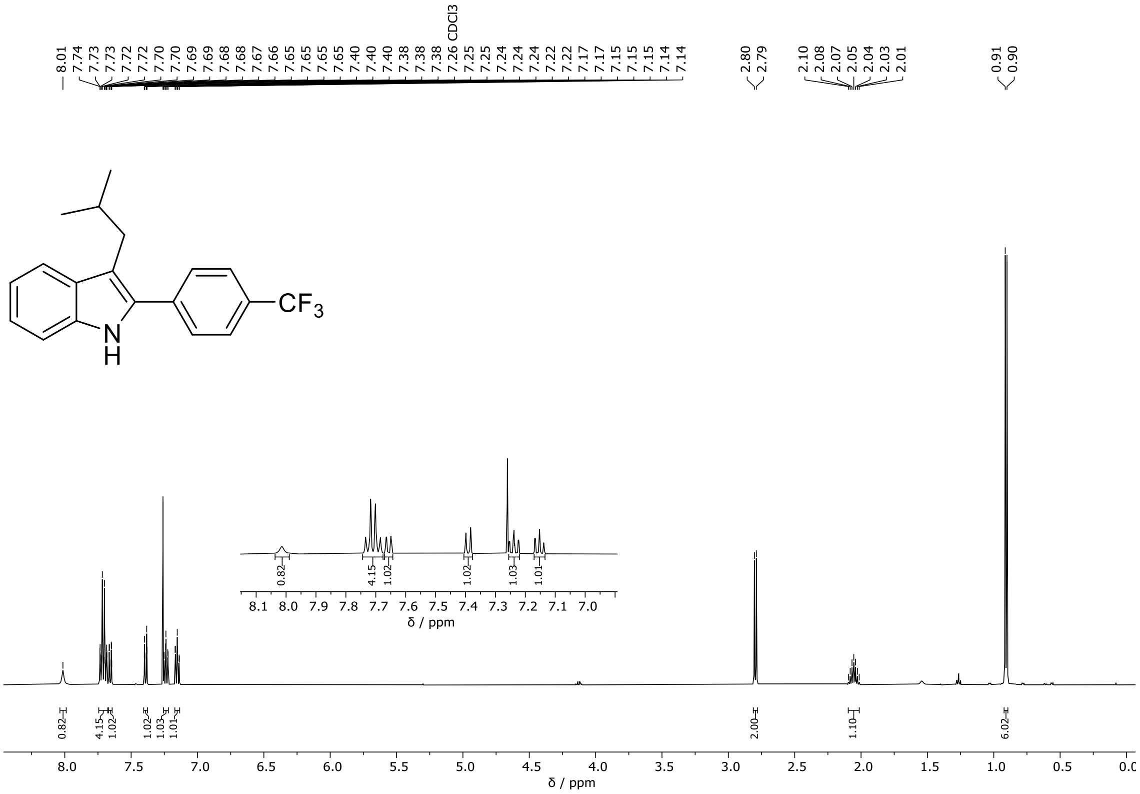

^{13}C-NMR{^{19}F} (126 MHz, $CDCl_3$)

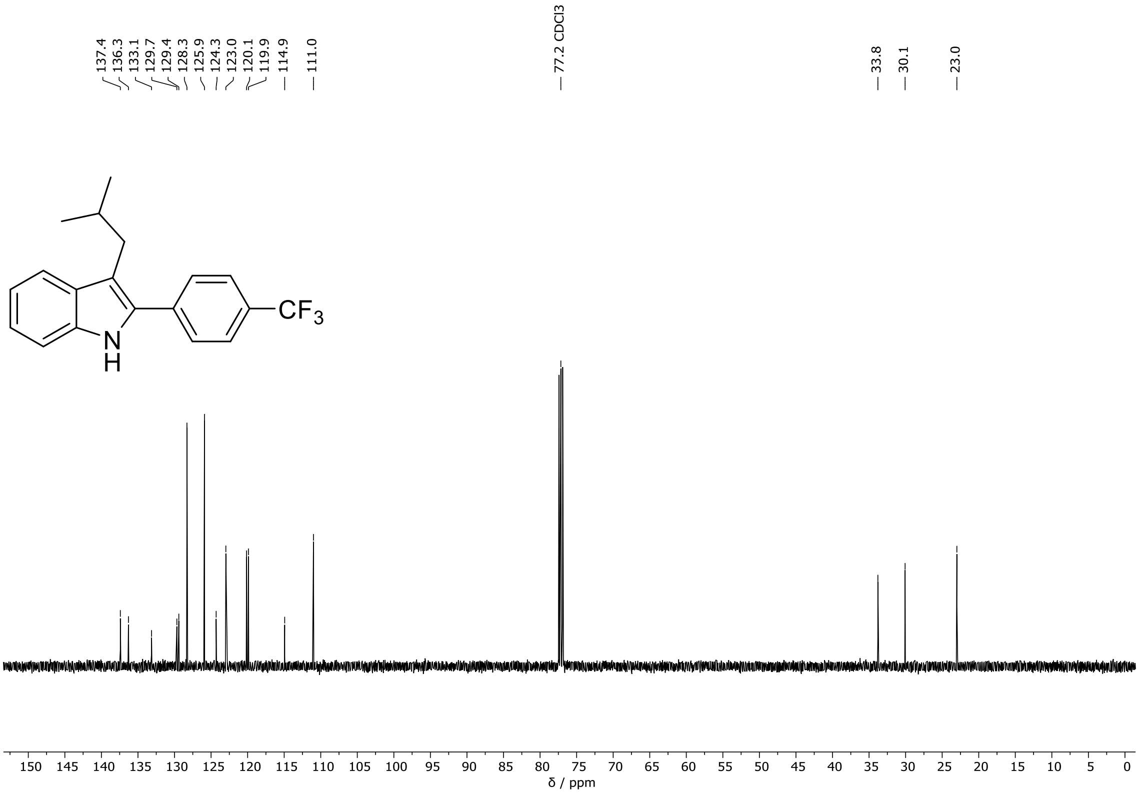

$^{19}F\{^{1}H\}$-NMR (470 MHz, $CDCl_3$)

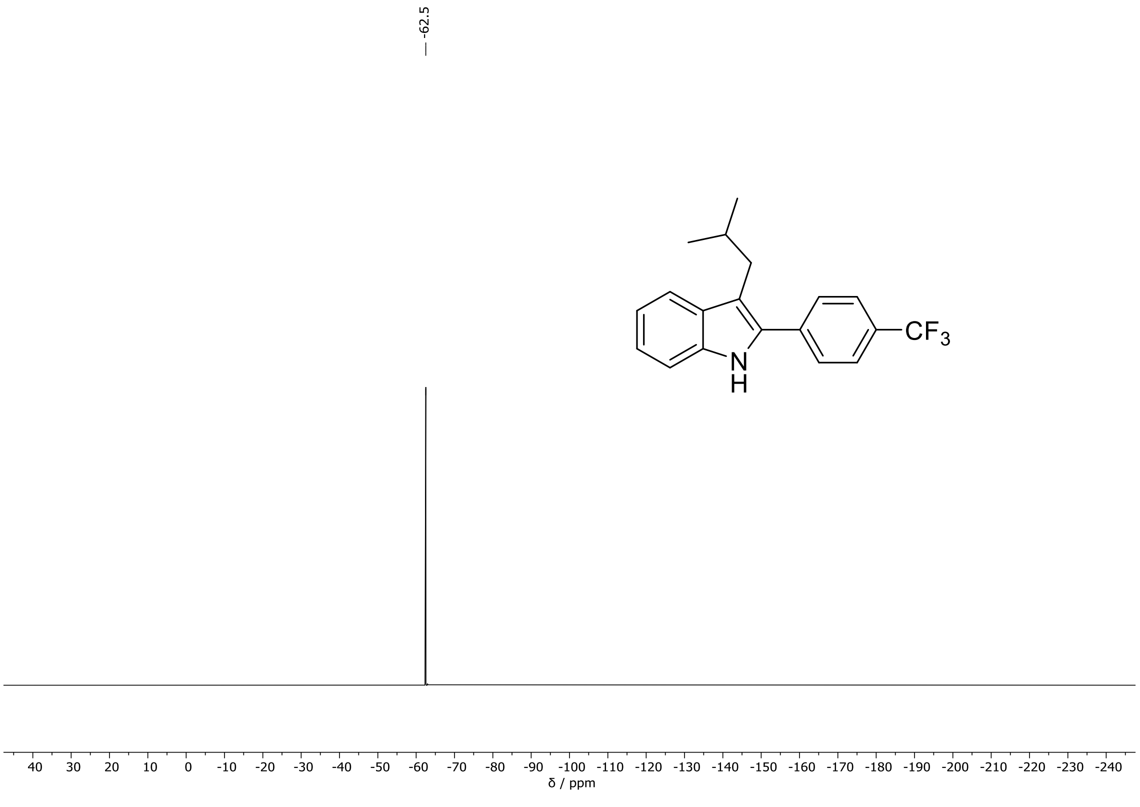

3-methyl-2-(4-(trifluoromethyl)phenyl)-1*H*-indole (2f):

^{1}H-NMR (400 MHz, DMSO-d_6)

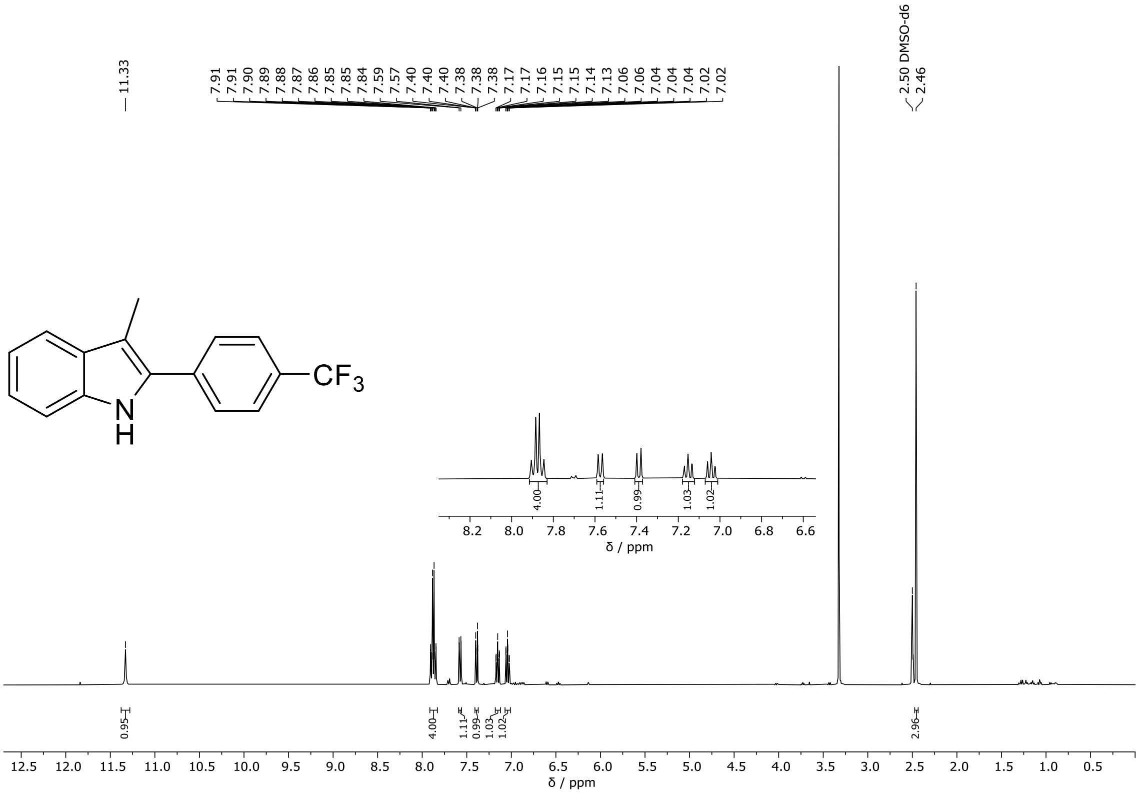

^{13}C-NMR (101 MHz, DMSO-d_6)

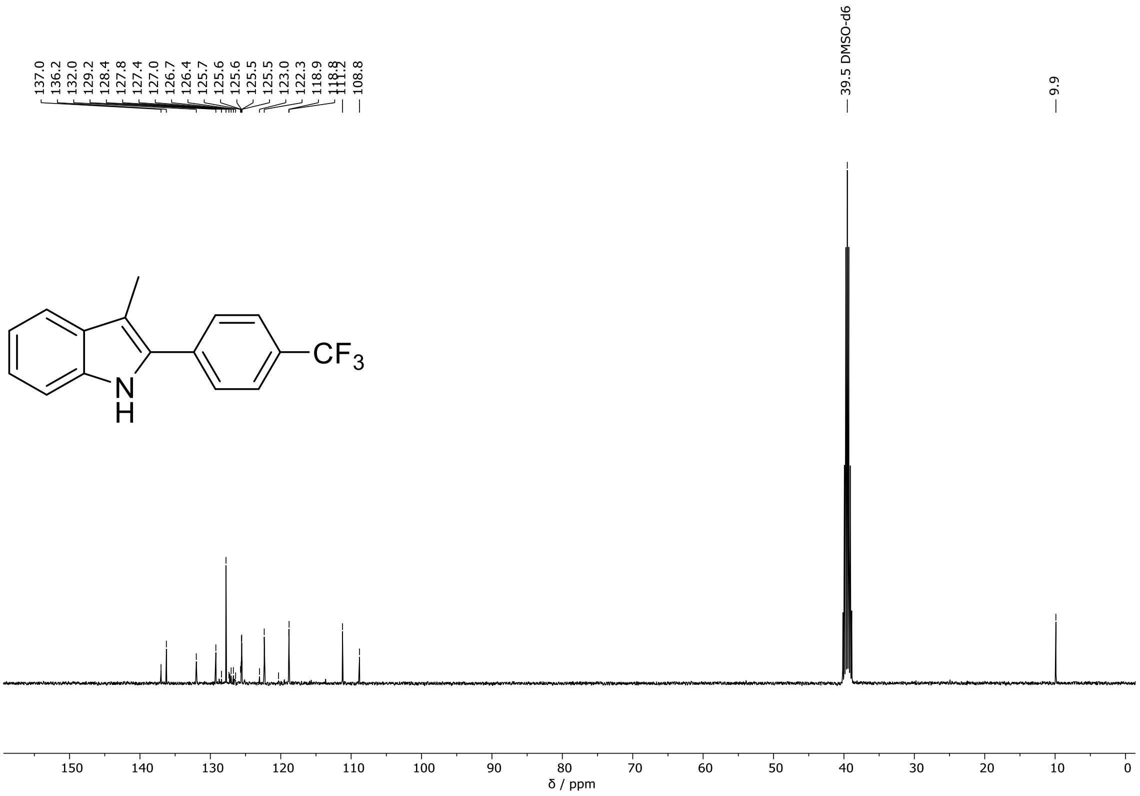

$^{19}F\{^{1}H\}$ NMR (376 MHz, DMSO-d_6)

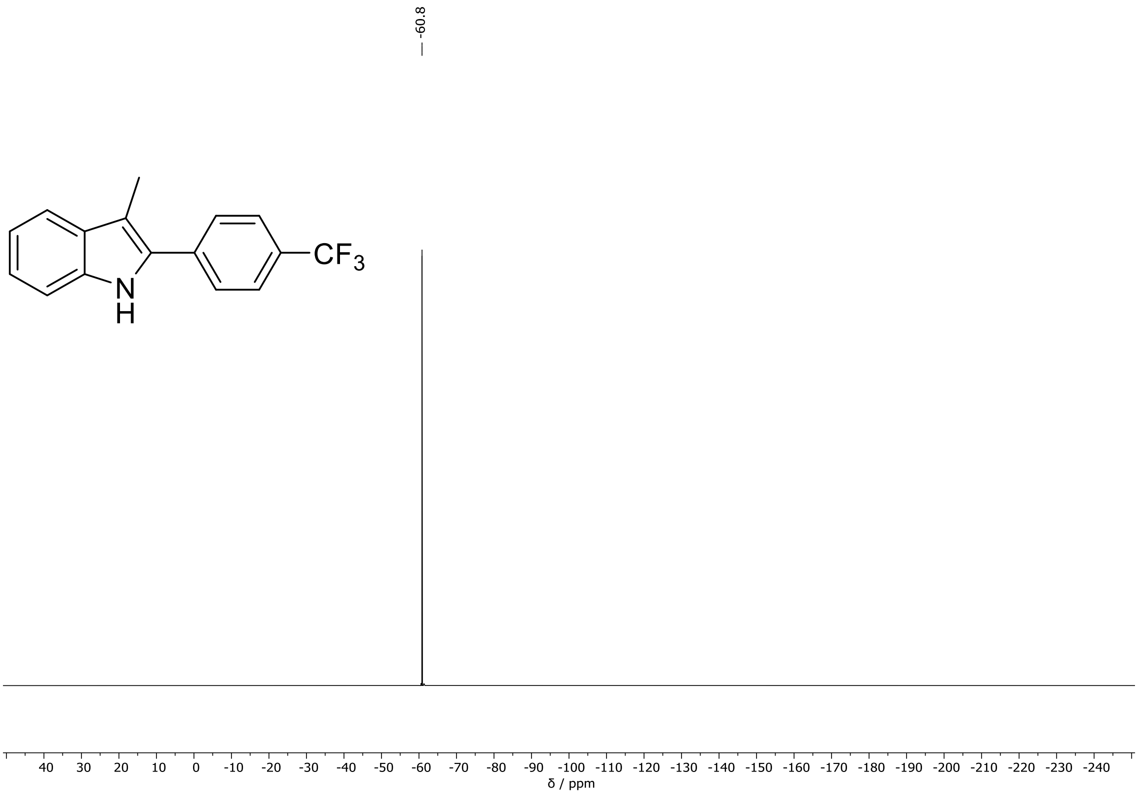

3-(2-methylpropyl)-2-(4-methoxyphenyl)-1*H*-indole (2g):

^{1}H-NMR (599 MHz, $CDCl_3$)

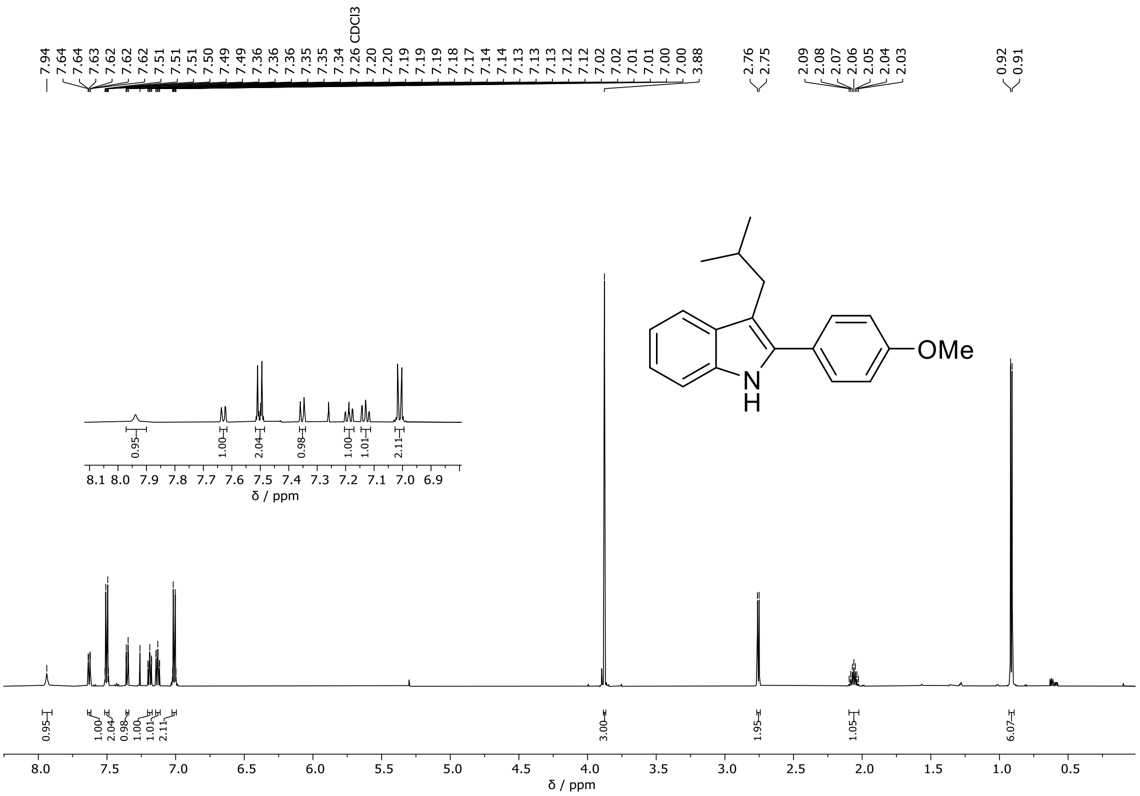

^{13}C-NMR (101 MHz, $CDCl_3$)

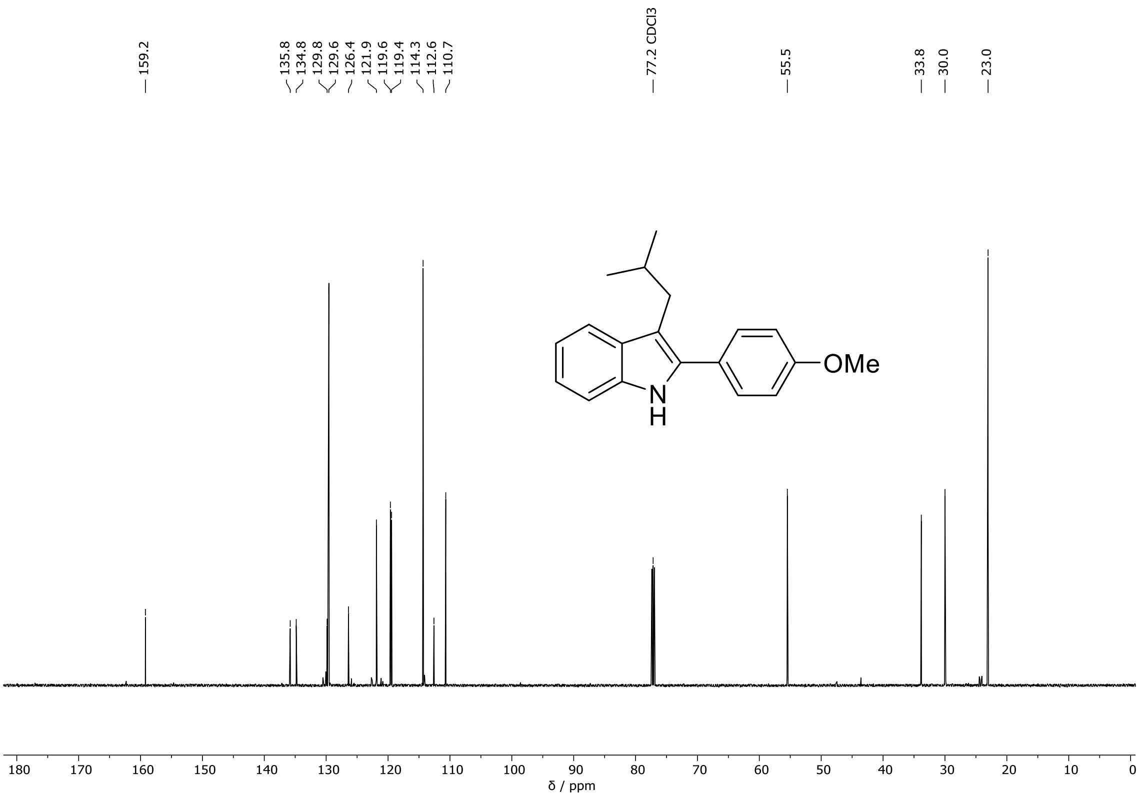

2-(4-methoxyphenyl)-3-methyl-1*H*-indole (2h):

^{1}H-NMR (400 MHz, $CDCl_3$)

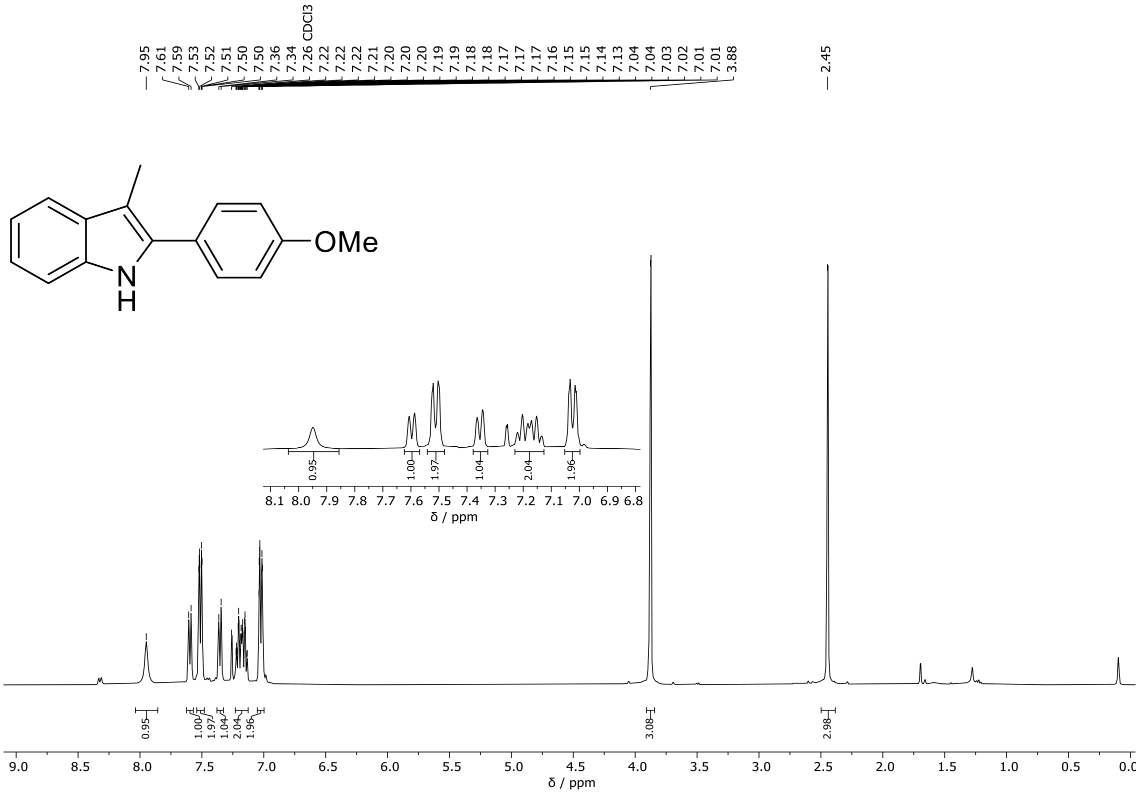

^{13}C-NMR (101 MHz, $CDCl_3$)

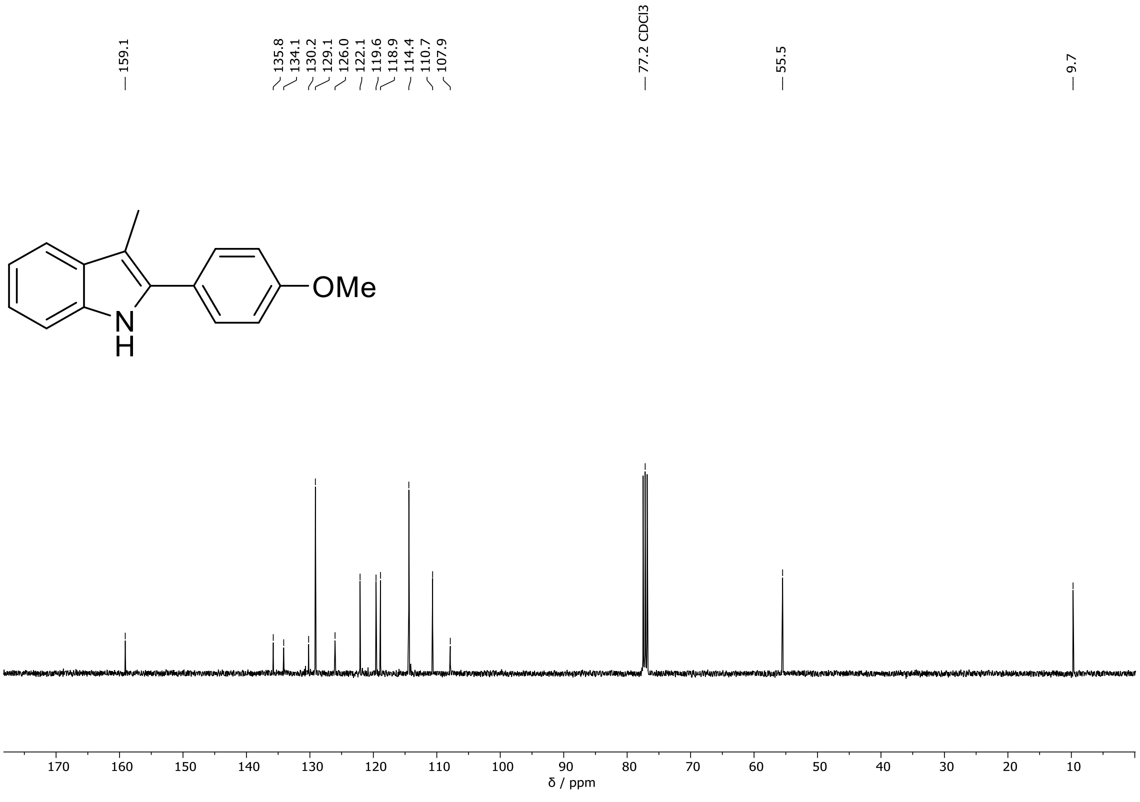

3-methyl-2-(4-methylphenyl)-1*H*-indole (2i):

^{1}H-NMR (400 MHz, $CDCl_3$)

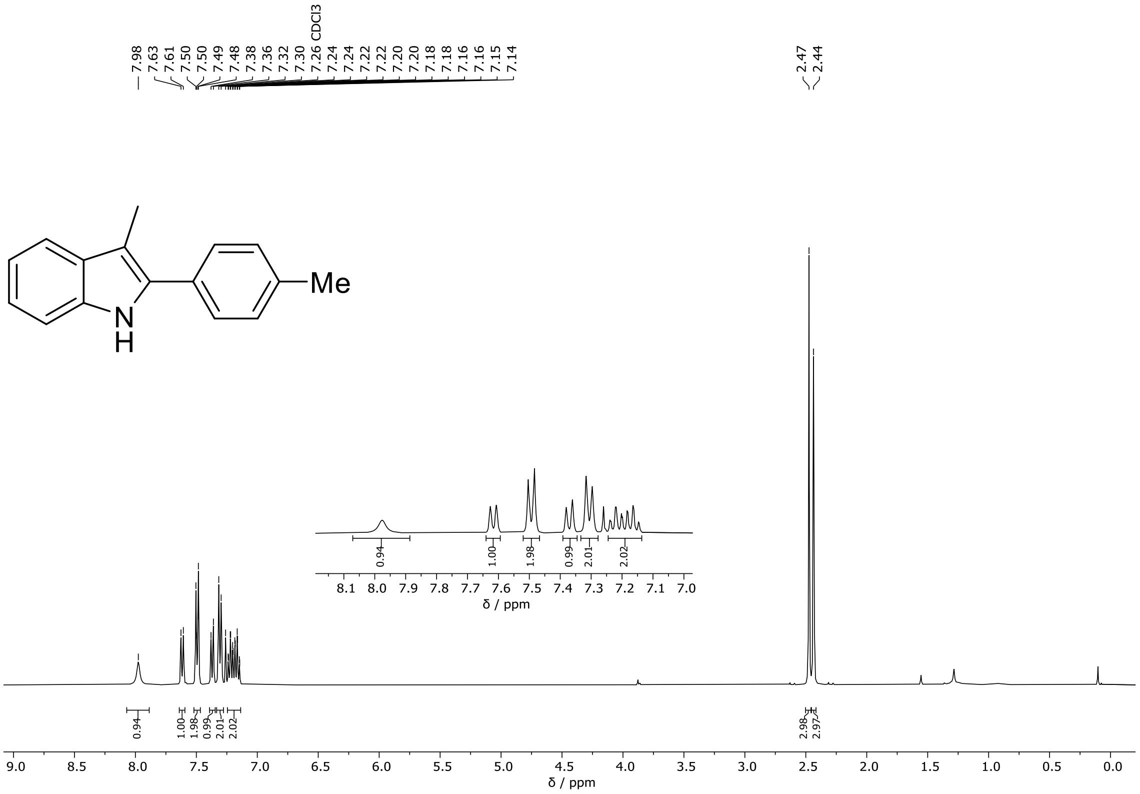

^{13}C-NMR (101 MHz, $CDCl_3$)

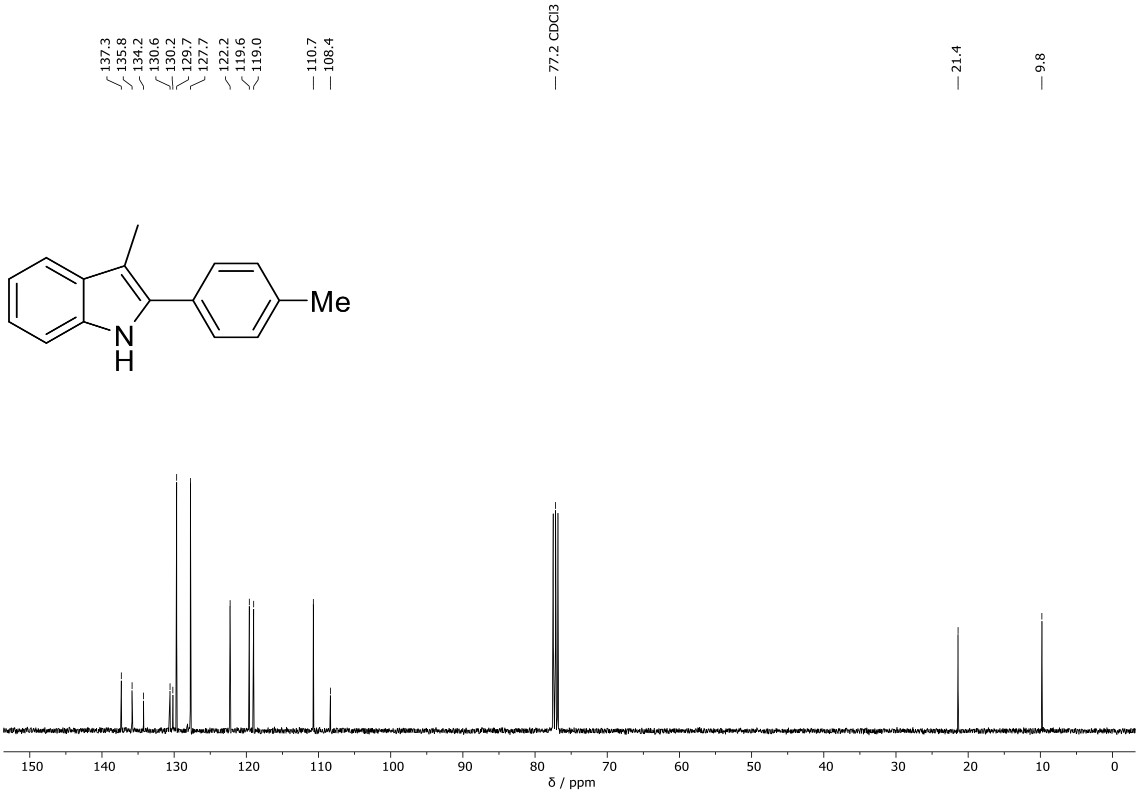

2-(4-fluorophenyl)-3-methyl-1*H*-indole (2j):

^{1}H-NMR (400 MHz, $CDCl_3$)

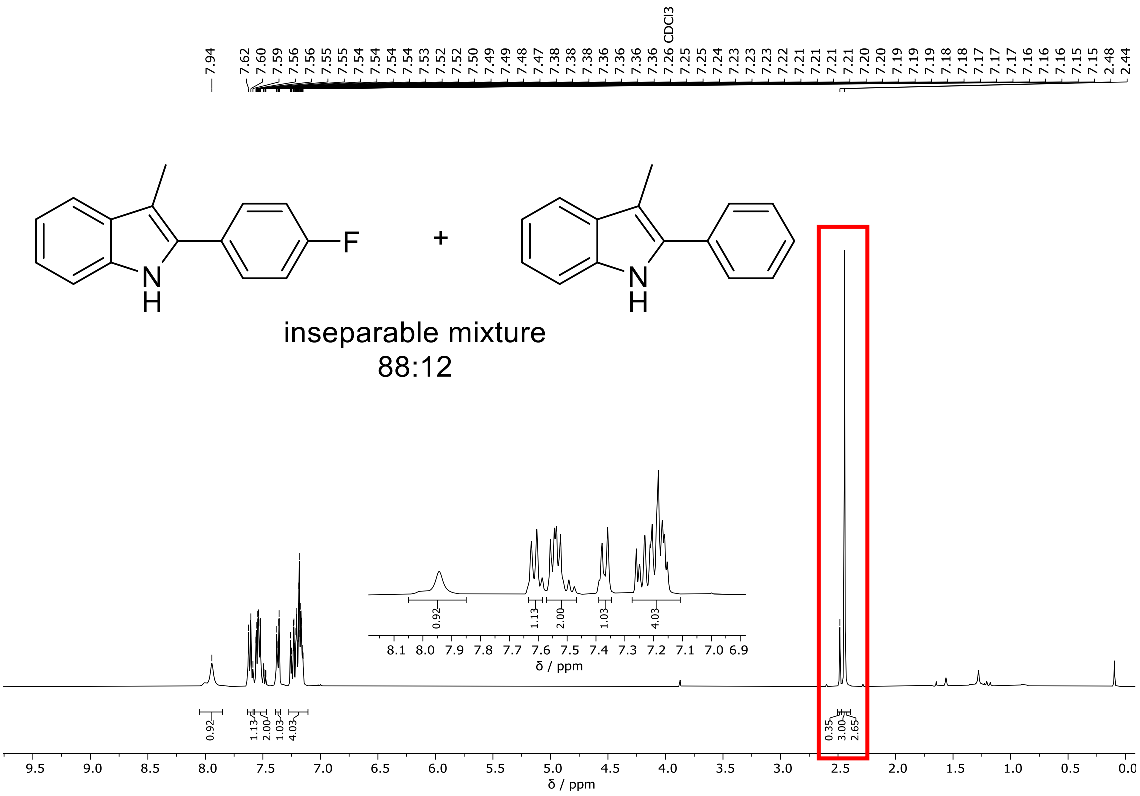

^{13}C-NMR (101 MHz, $CDCl_3$)

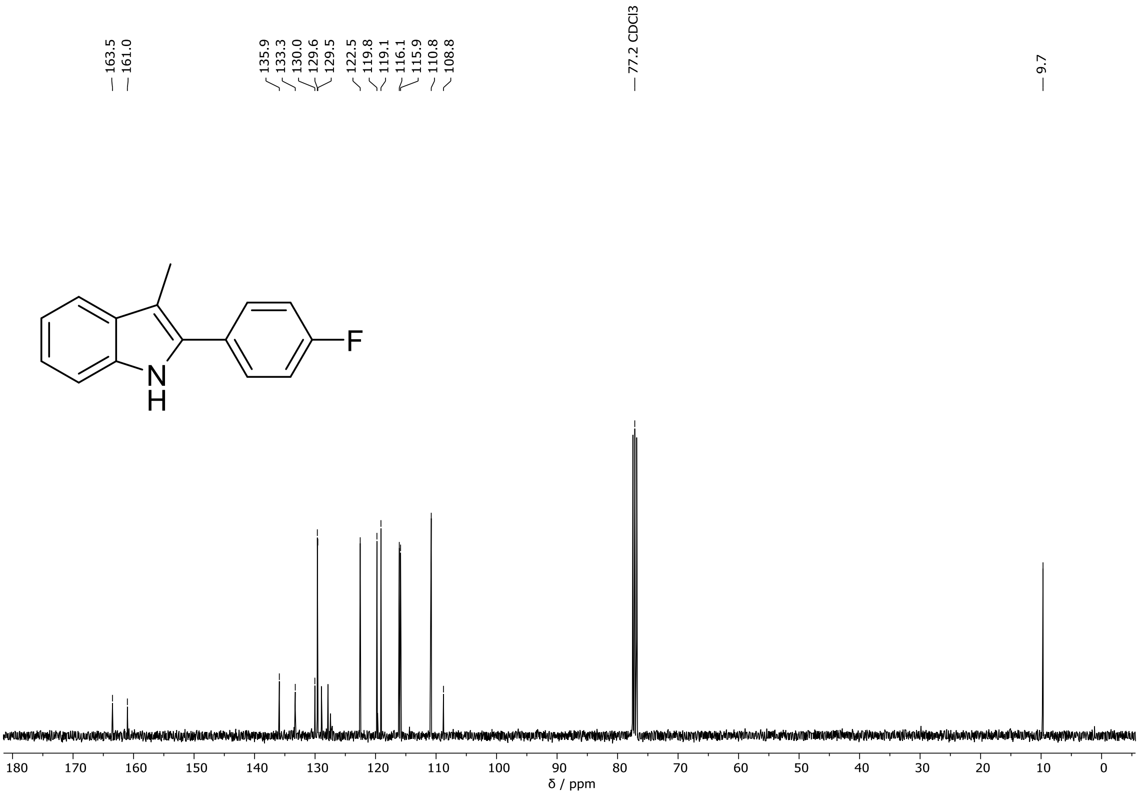

$^{19}F\{^{1}H\}$-NMR (376 MHz, $CDCl_3$)

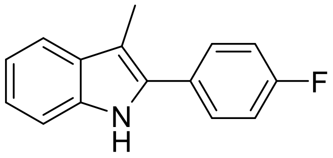

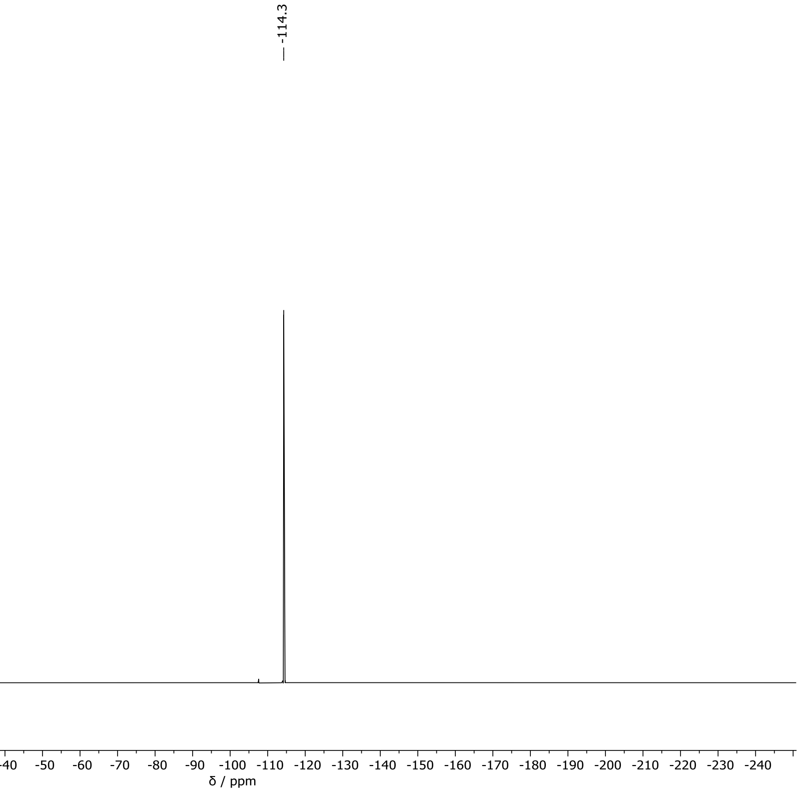

3-methyl-2-(3-(trimethylsilyl)phenyl)-1*H*-indole (2k):

^{1}H-NMR (599 MHz, $CDCl_3$)

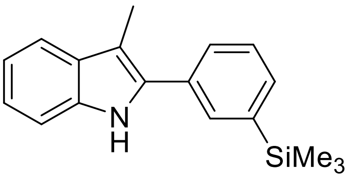

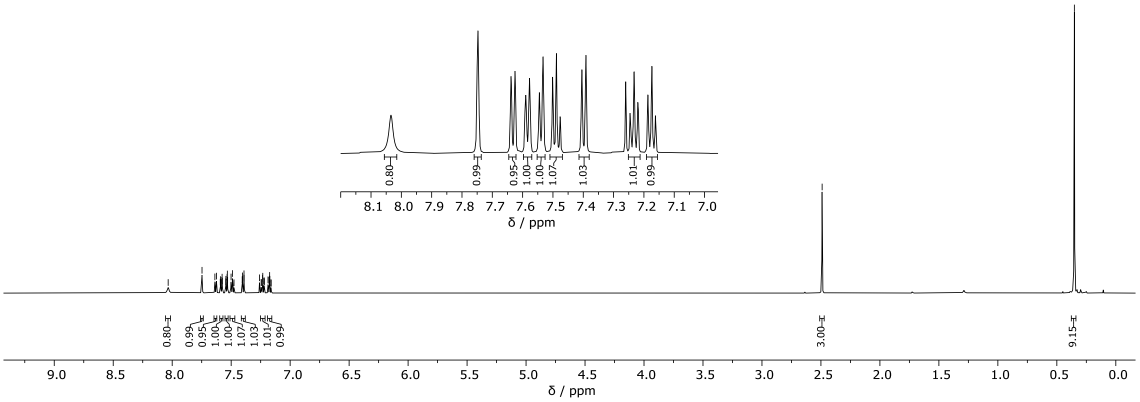

^{13}C-NMR (151 MHz, $CDCl_3$)

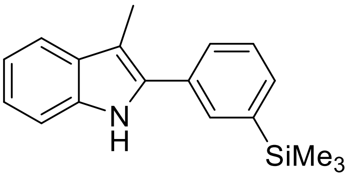

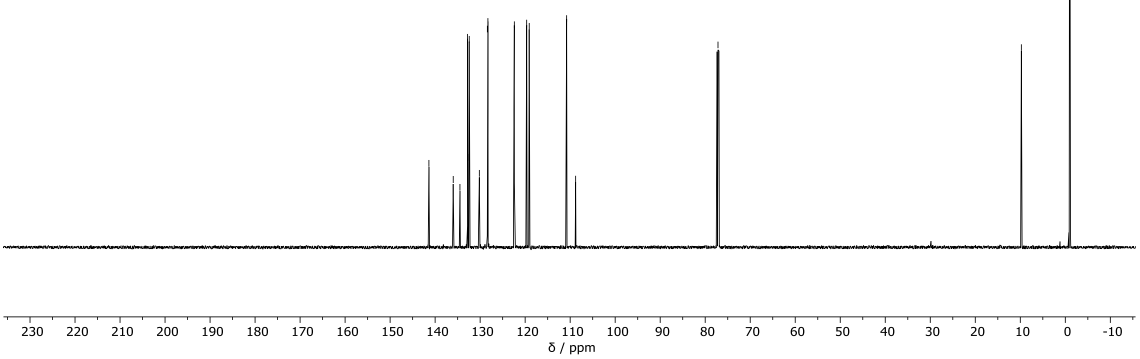

^{29}Si-NMR (119 MHz, $CDCl_3$)

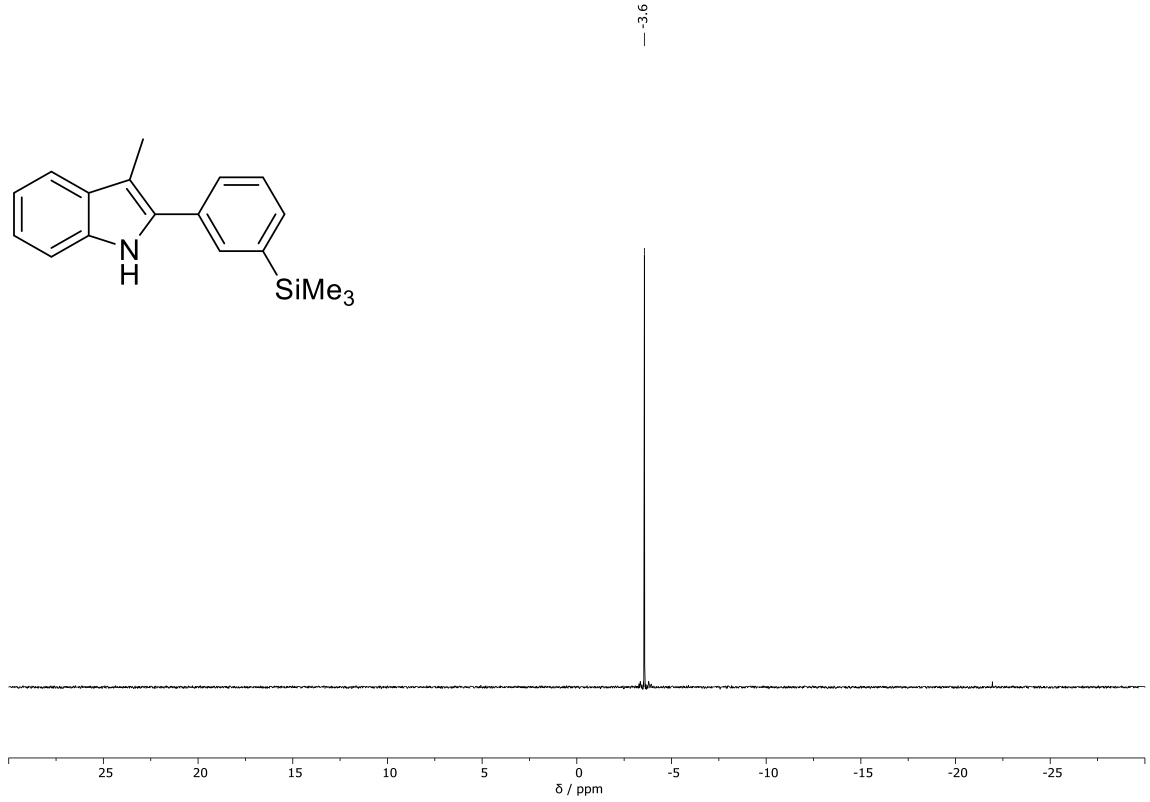

3-(3-ethyl-1*H*-indol-2-yl)phenol (2l):

^{1}H-NMR (300 MHz, $CDCl_3$)

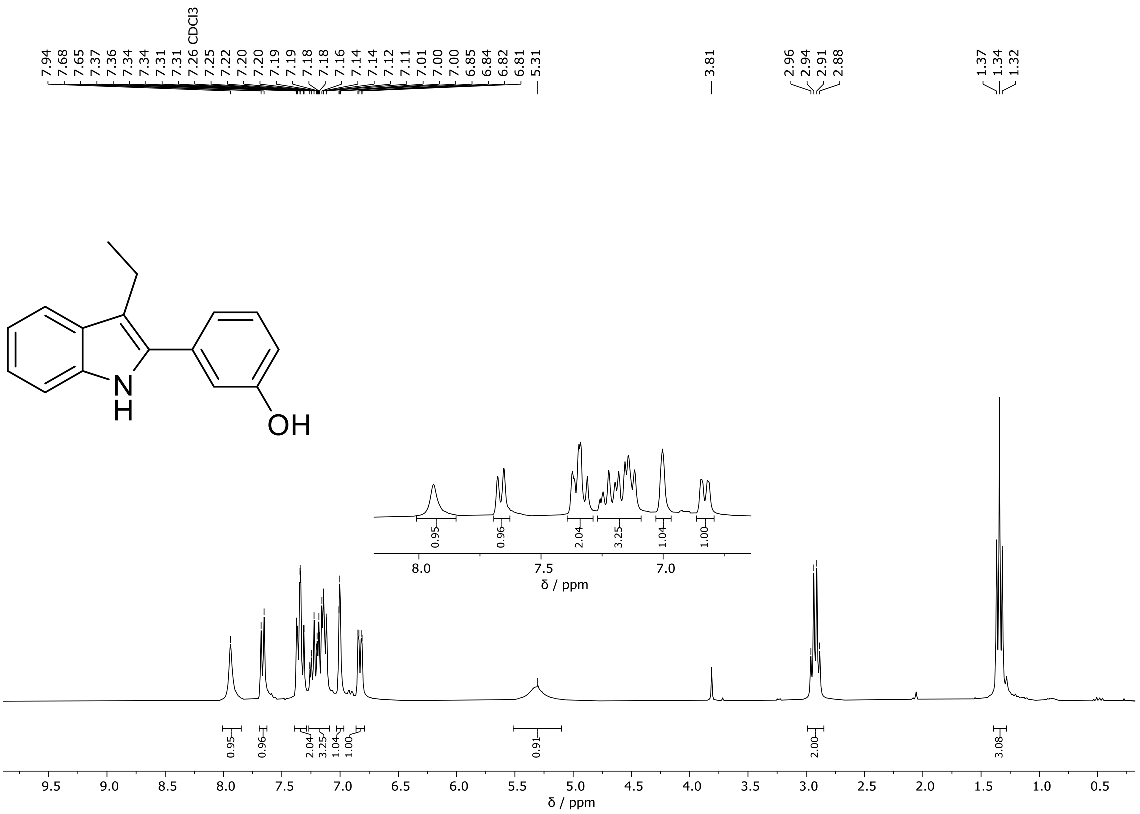

^{13}C-NMR (101 MHz, $CDCl_3$)

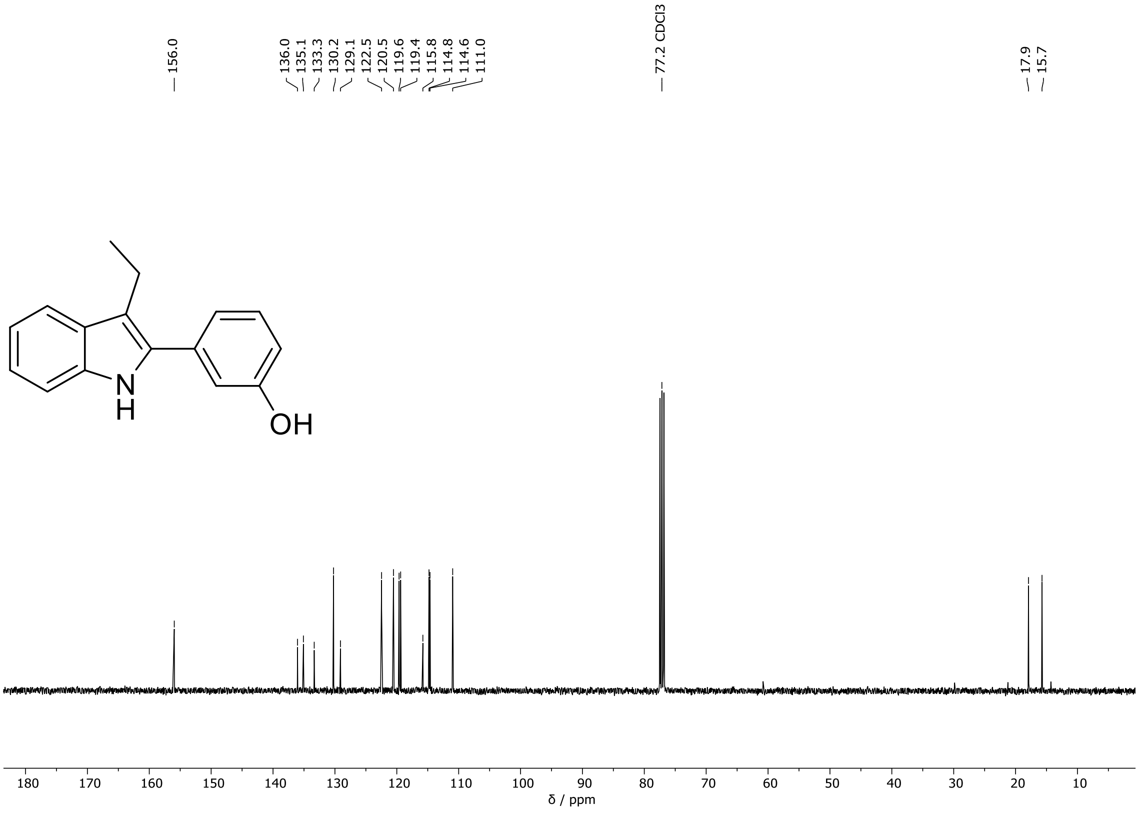

2-(3-methoxyphenyl)-3-methyl-1*H*-indole (2m):

^{1}H-NMR (400 MHz, $CDCl_3$)

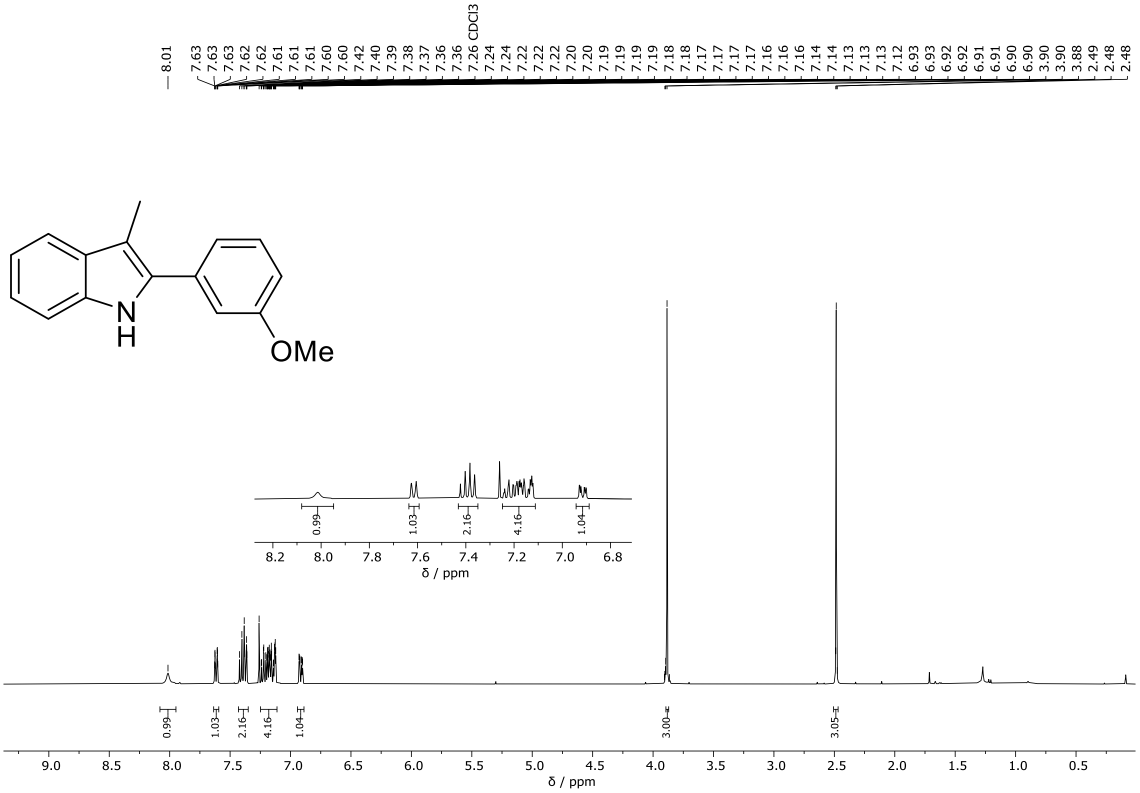

^{13}C-NMR (101 MHz, $CDCl_3$)

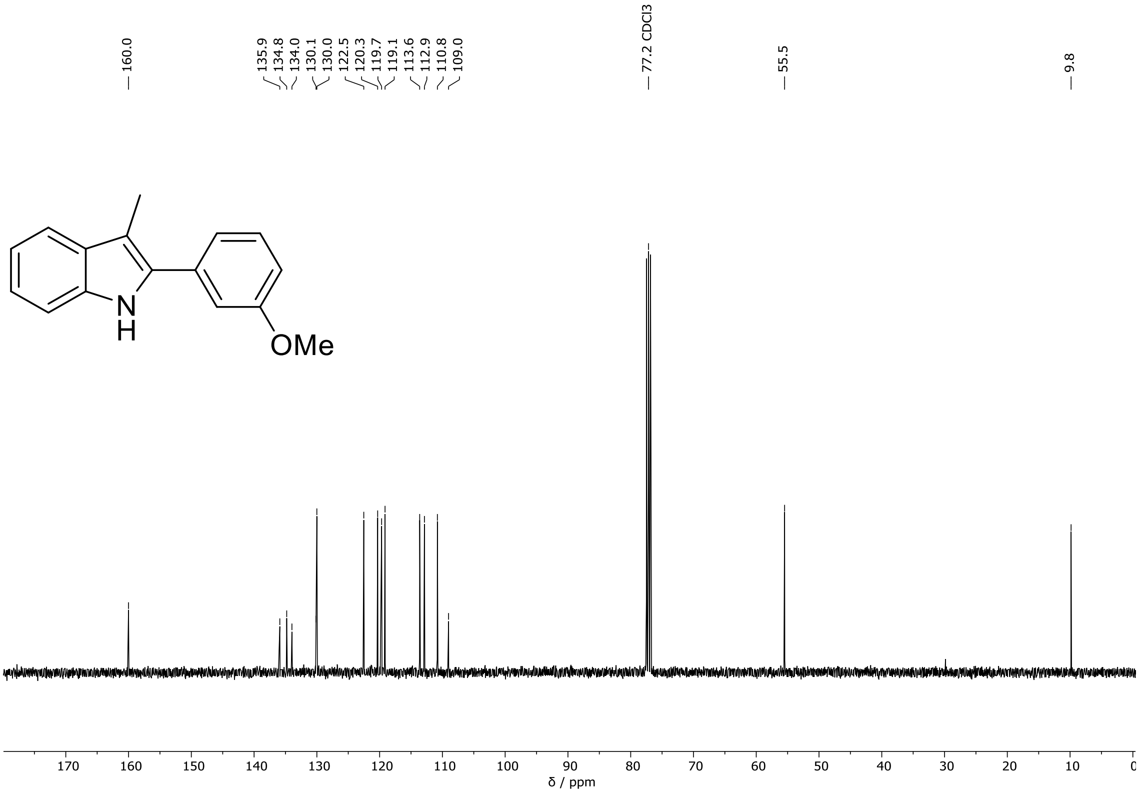

2-(2-methoxyphenyl)-3-methyl-1*H*-indole (2n):

^{1}H-NMR (400 MHz, $CDCl_3$)

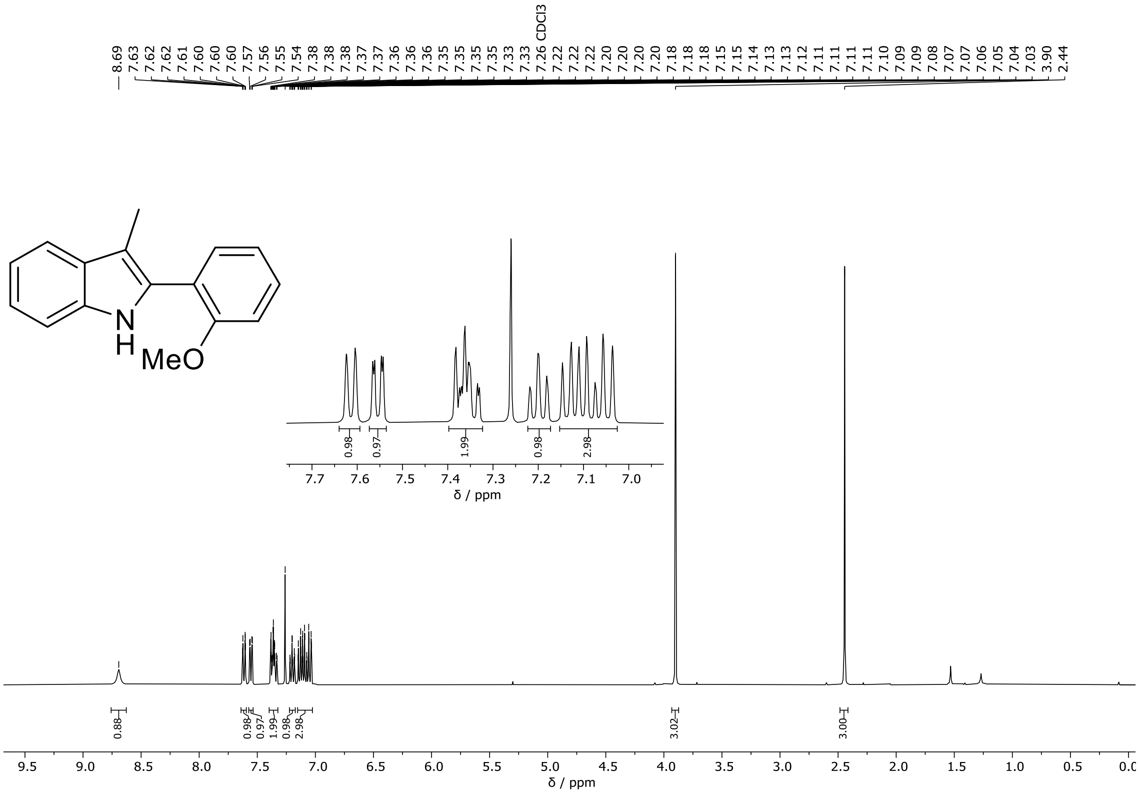

^{13}C-NMR (101 MHz, $CDCl_3$)

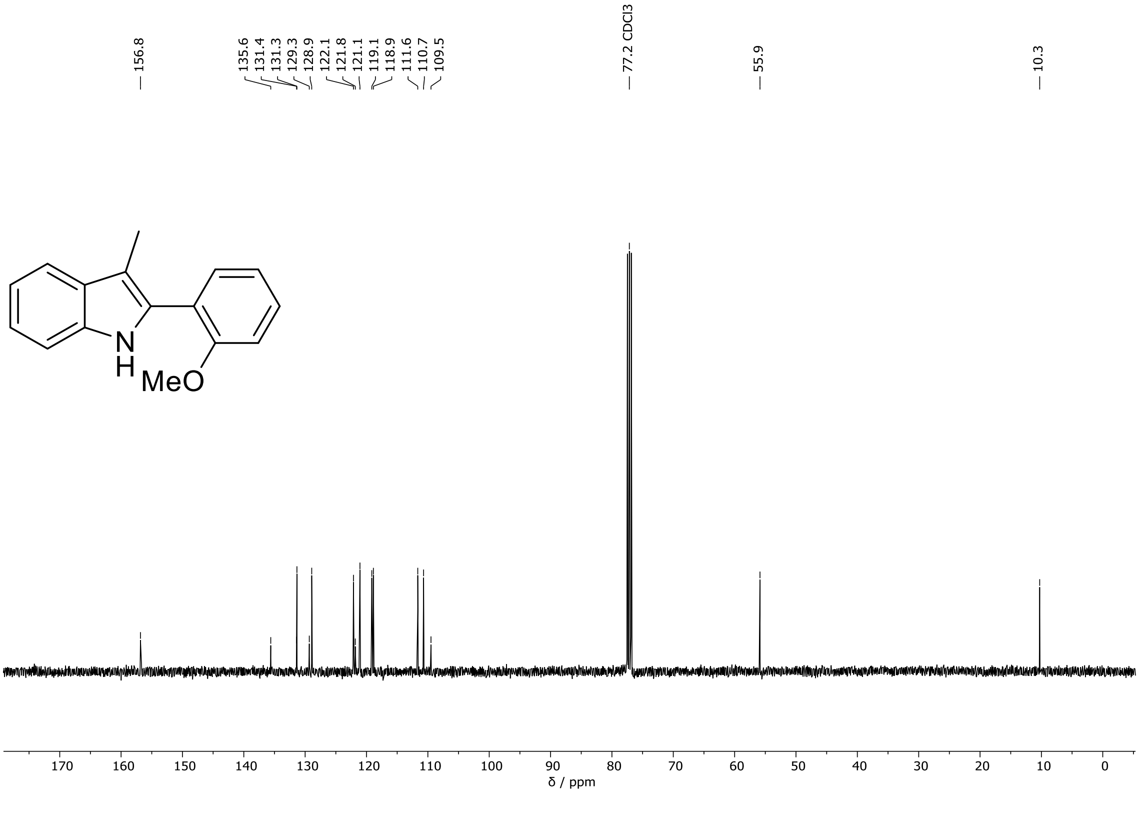

3-isobutyl-4-methyl-2-phenyl-1*H*-indole (2o):

^{1}H-NMR (400 MHz, $CDCl_3$)

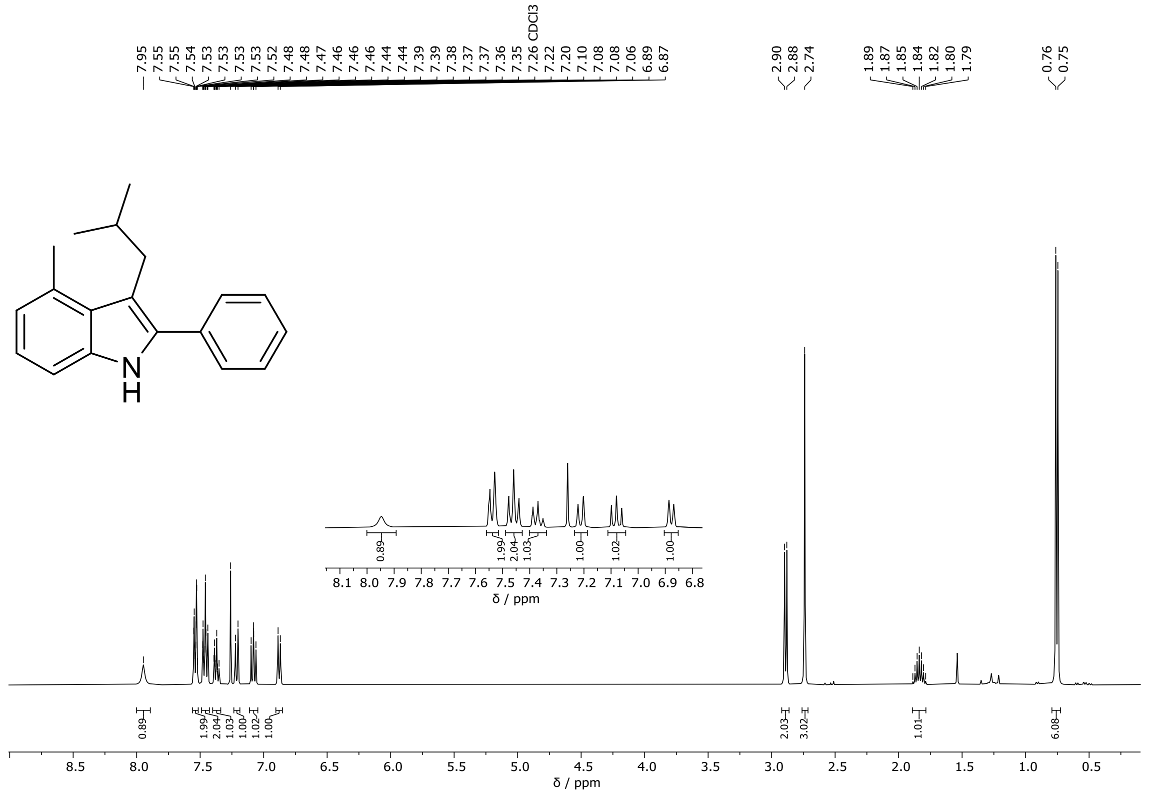

^{13}C-NMR (101 MHz, $CDCl_3$)

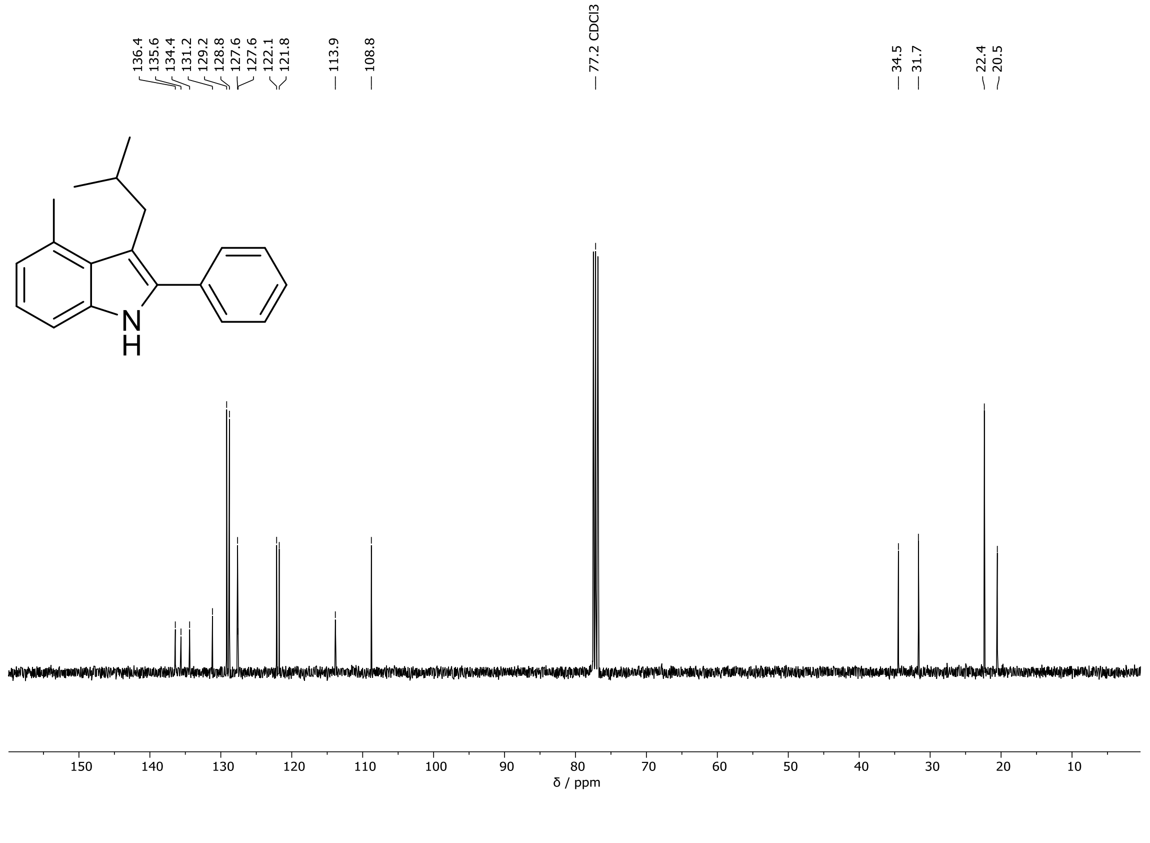

3-isobutyl-5-methyl-2-phenyl-1*H*-indole (2p):

^{1}H-NMR (400 MHz, $CDCl_3$)

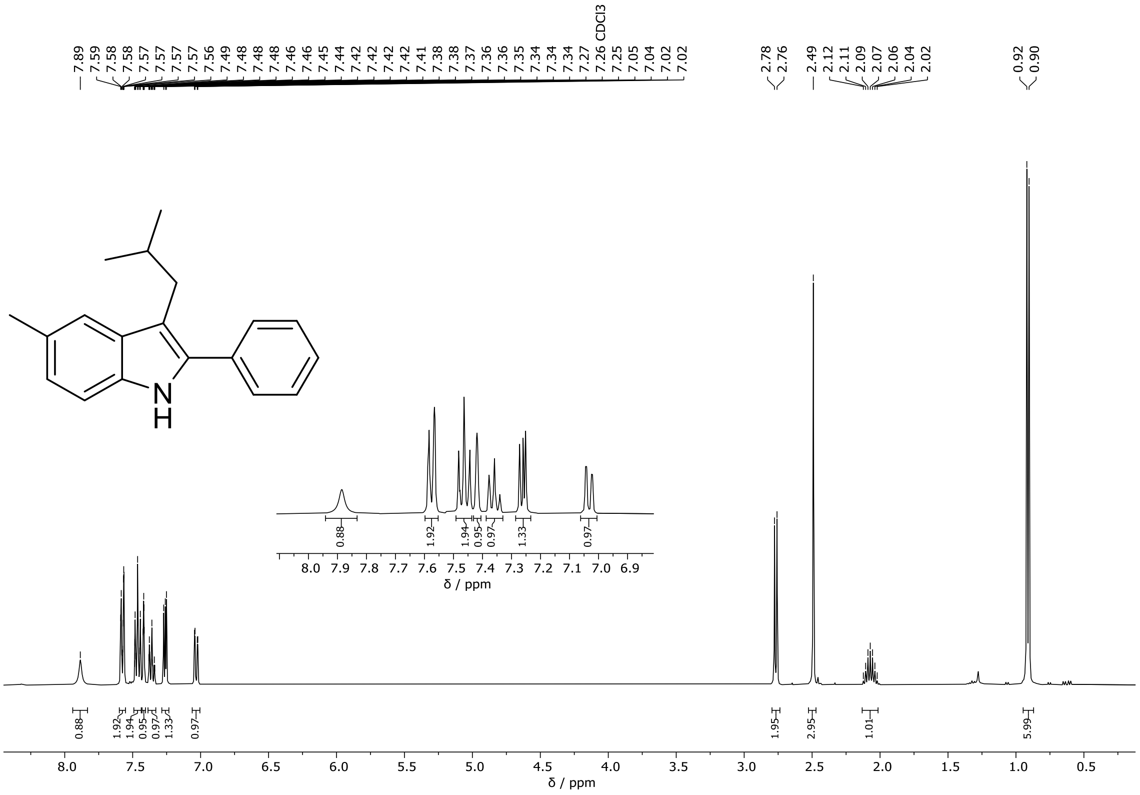

^{13}C-NMR (101 MHz, $CDCl_3$)

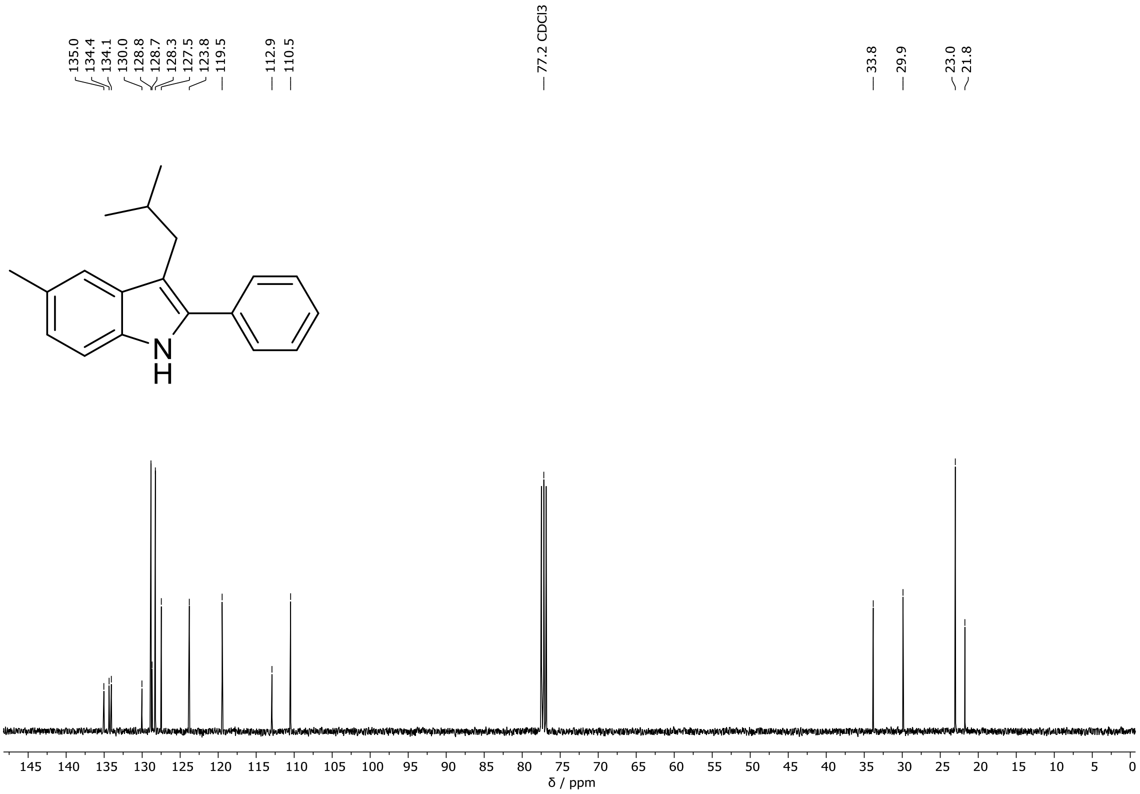

3-isobutyl-6-methyl-2-phenyl-1*H*-indole (2q):

^{1}H-NMR (400 MHz, $CDCl_3$)

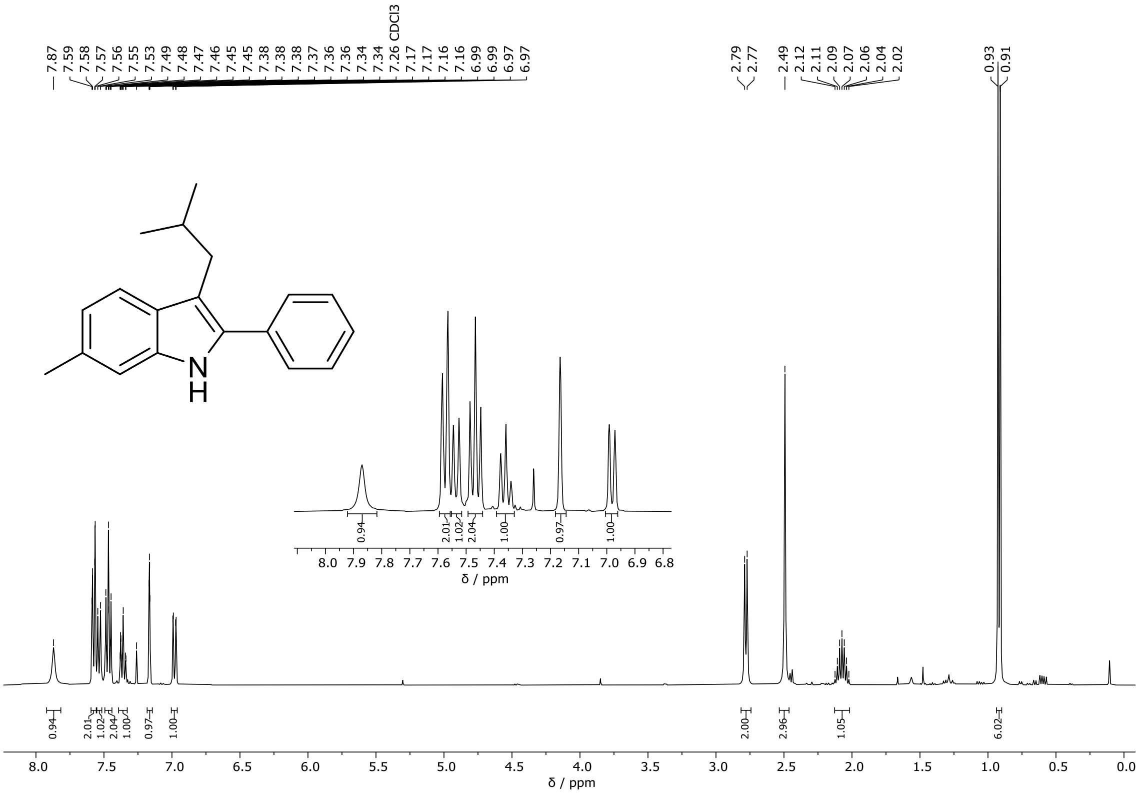

^{13}C-NMR (101 MHz, $CDCl_3$)

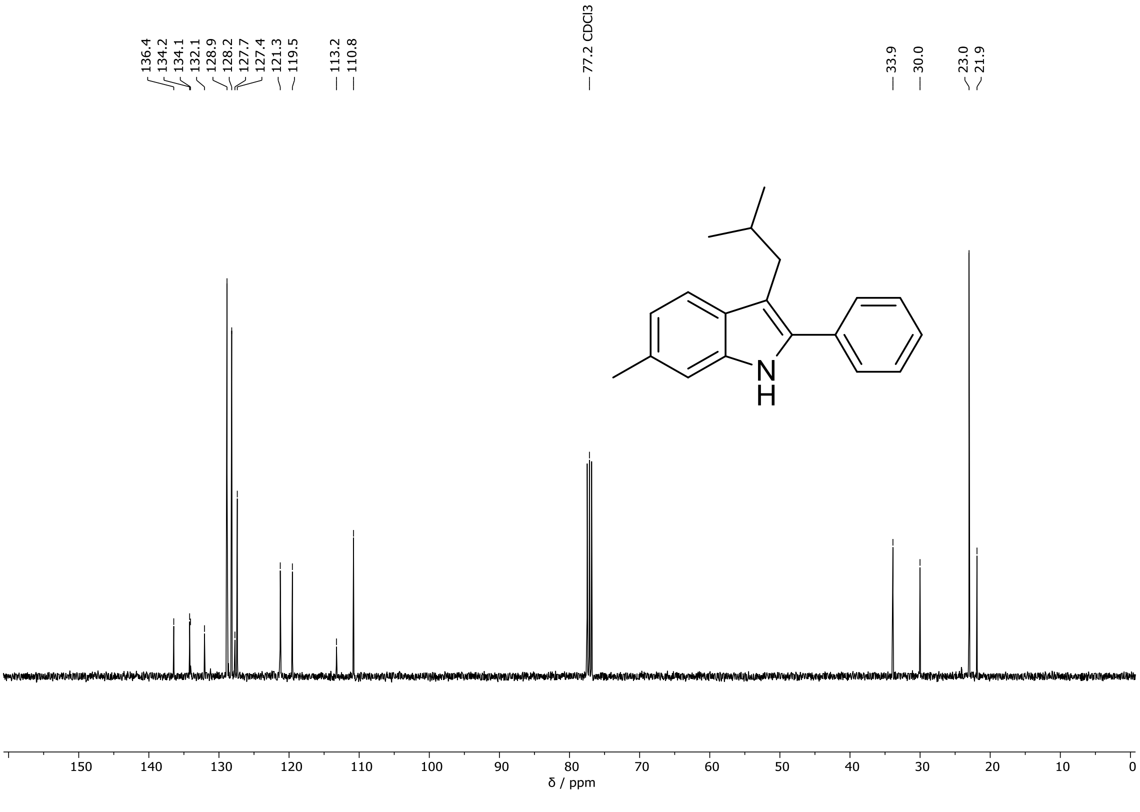

3-isobutyl-7-methyl-2-phenyl-1*H*-indole (2r):

^{1}H-NMR (400 MHz, $CDCl_3$)

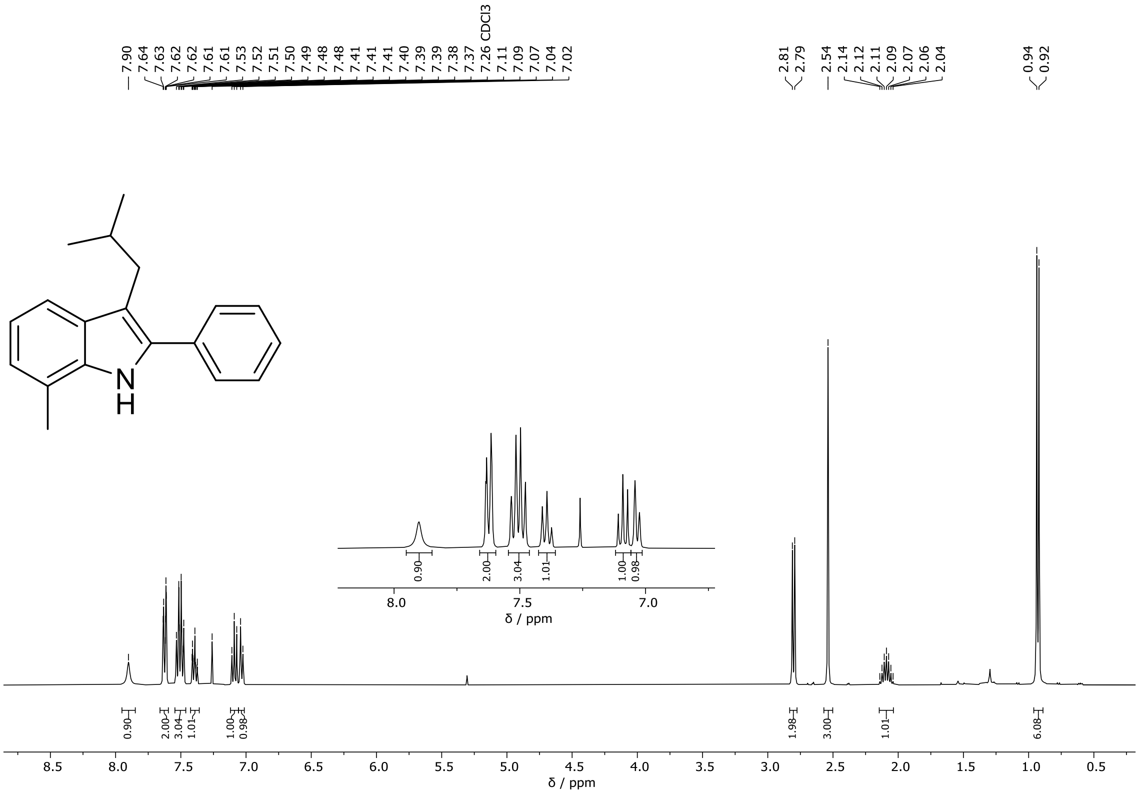

^{13}C-NMR (101 MHz, $CDCl_3$)

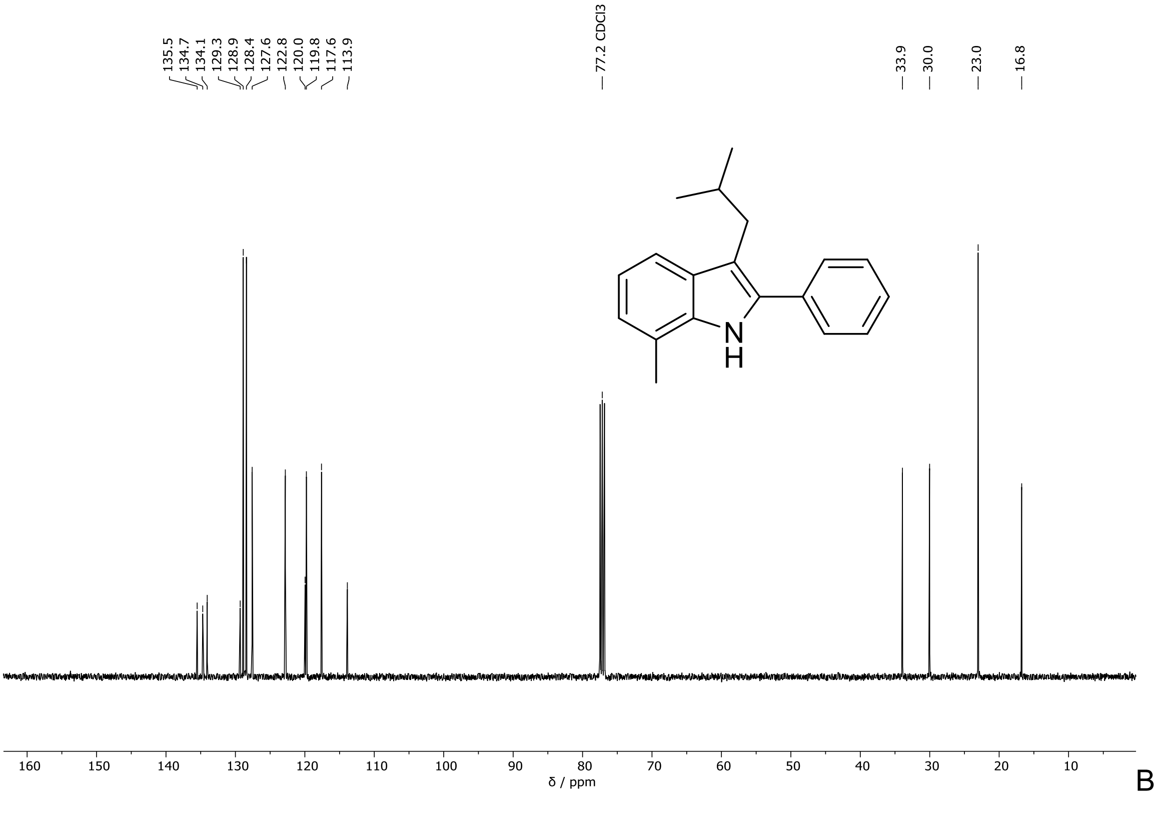

B

5-bromo-3-isobutyl-2-phenyl-1*H*-indole (2s):

^{1}H-NMR (599 MHz, $CDCl_3$)

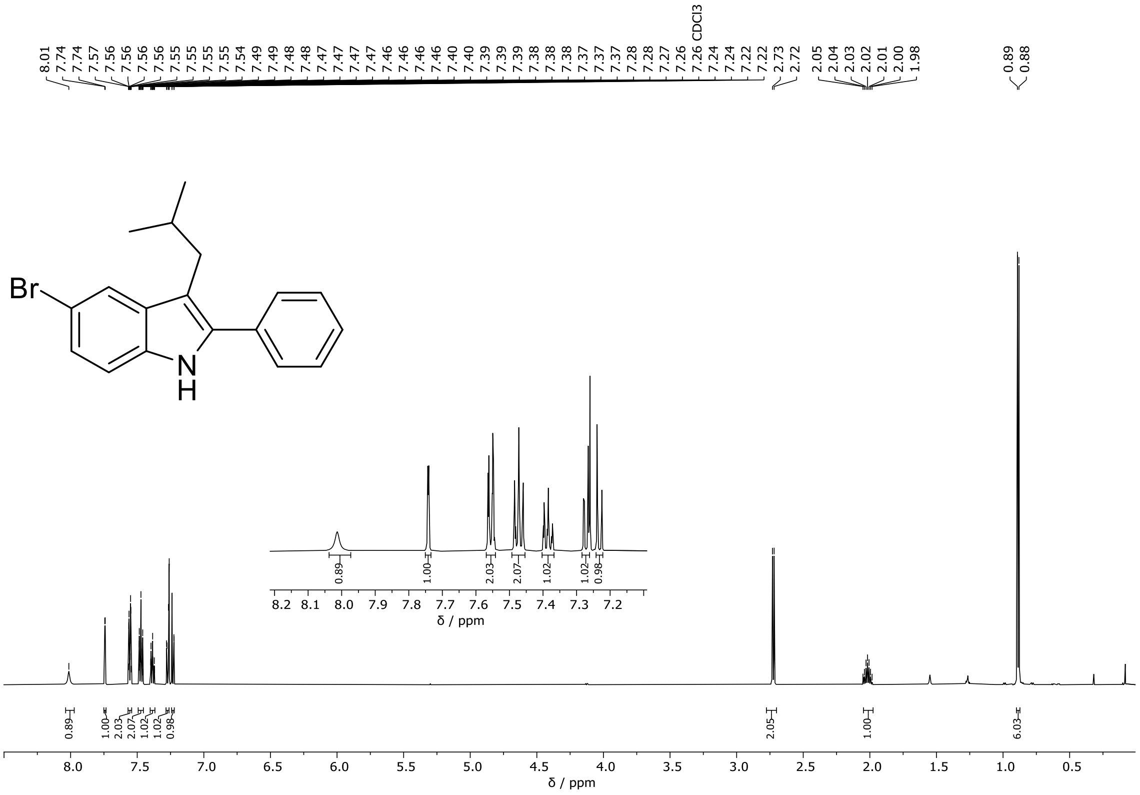

^{13}C-NMR (151 MHz, $CDCl_3$)

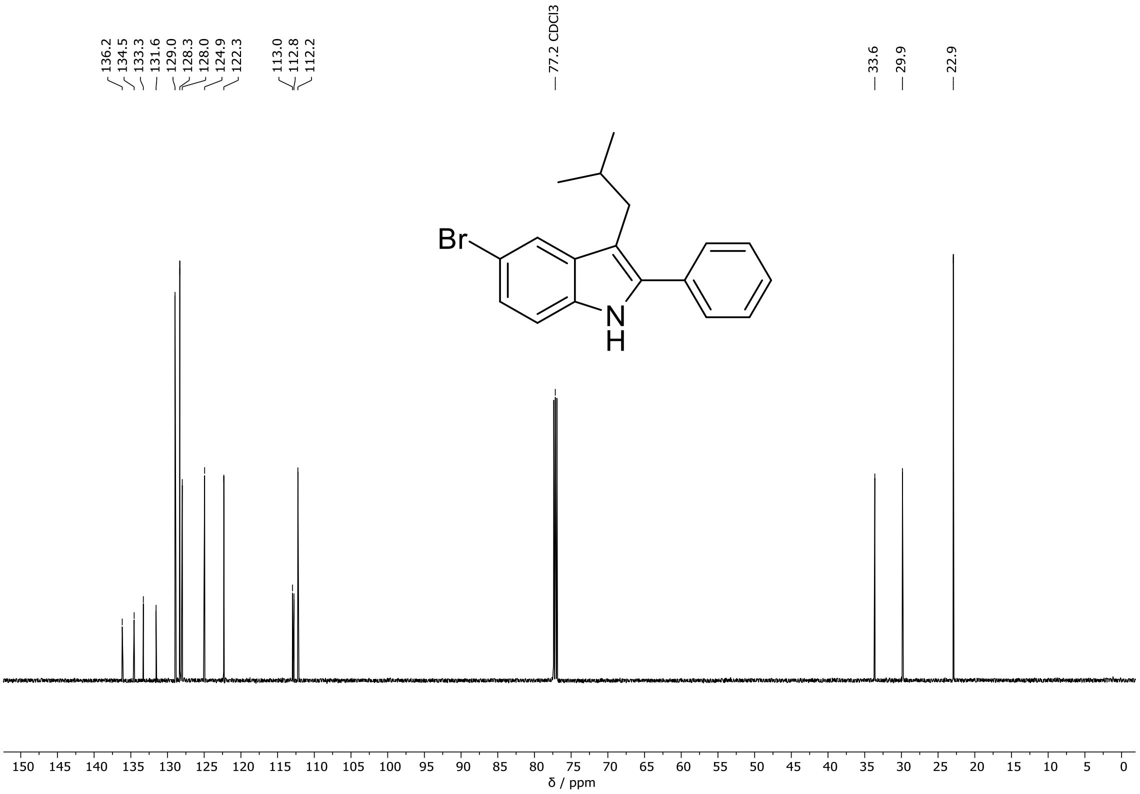

6-chloro-3-isobutyl-2-phenyl-1*H*-indole (2t):

^{1}H-NMR (400 MHz, $CDCl_3$)

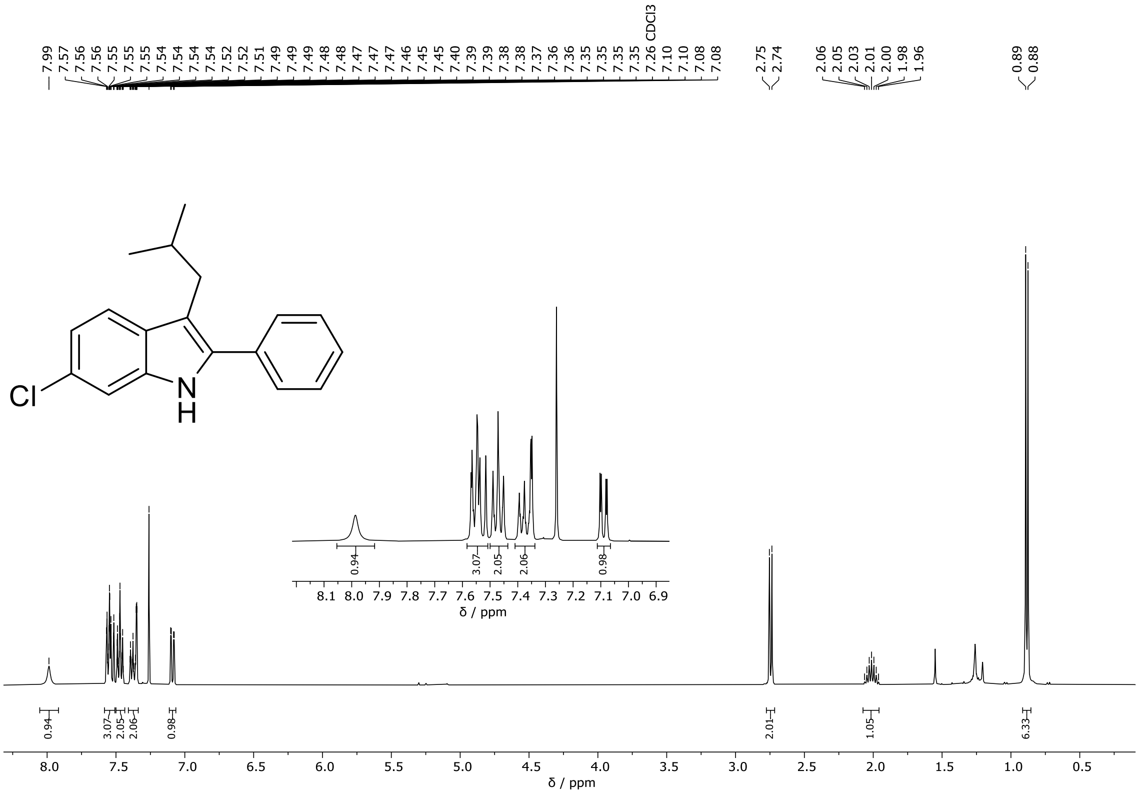

^{13}C-NMR (101 MHz, $CDCl_3$)

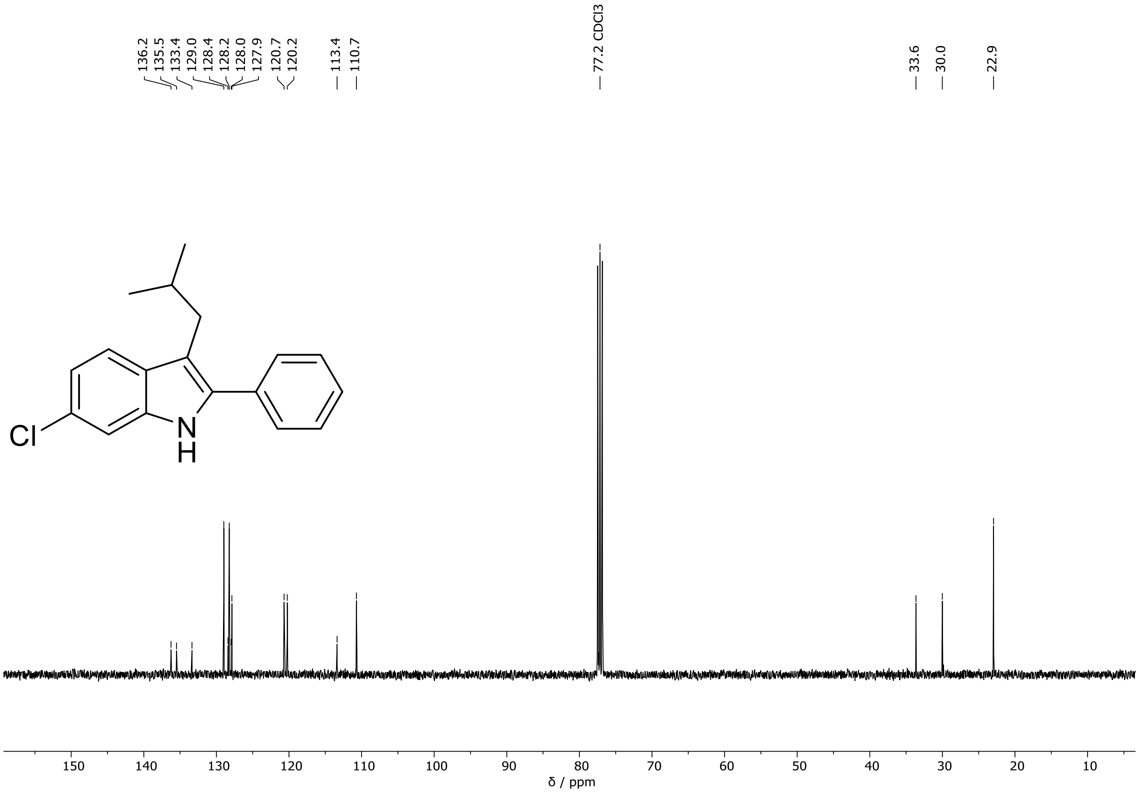

3-ethyl-5-fluoro-2-phenyl-1*H*-indole (2u):

^{1}H-NMR (400 MHz, $CDCl_3$)

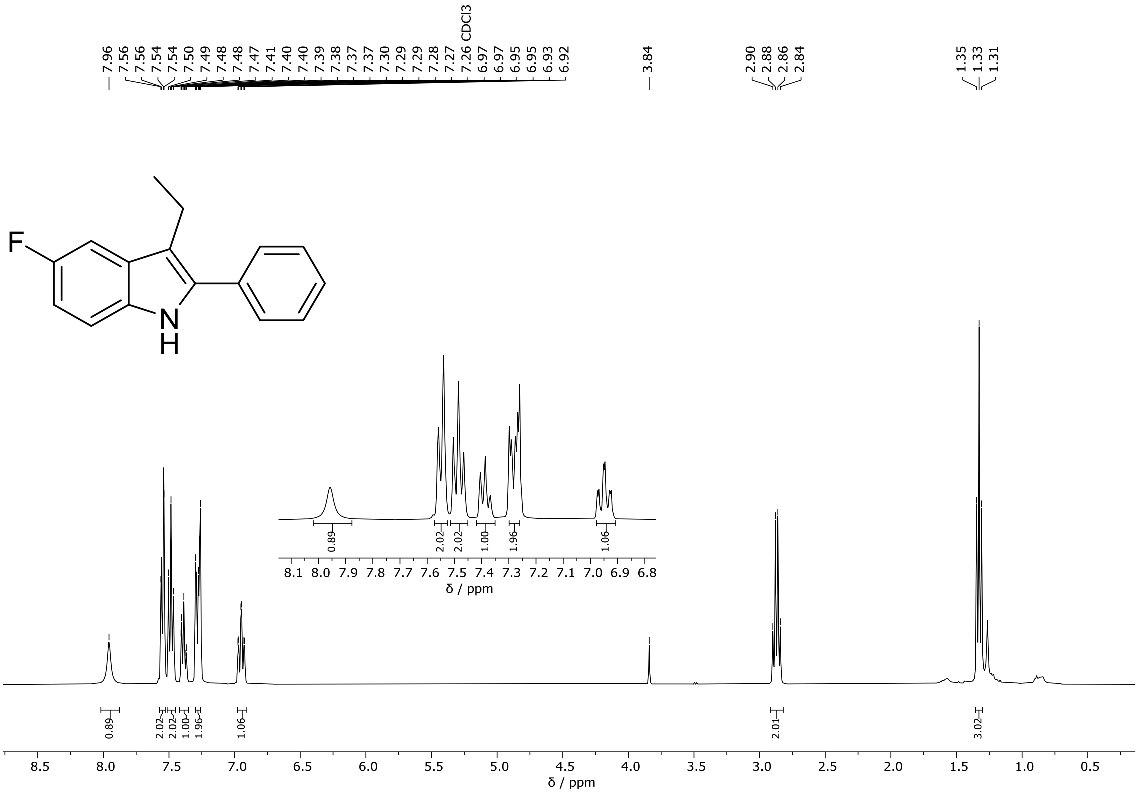

^{13}C-NMR (101 MHz, $CDCl_3$)

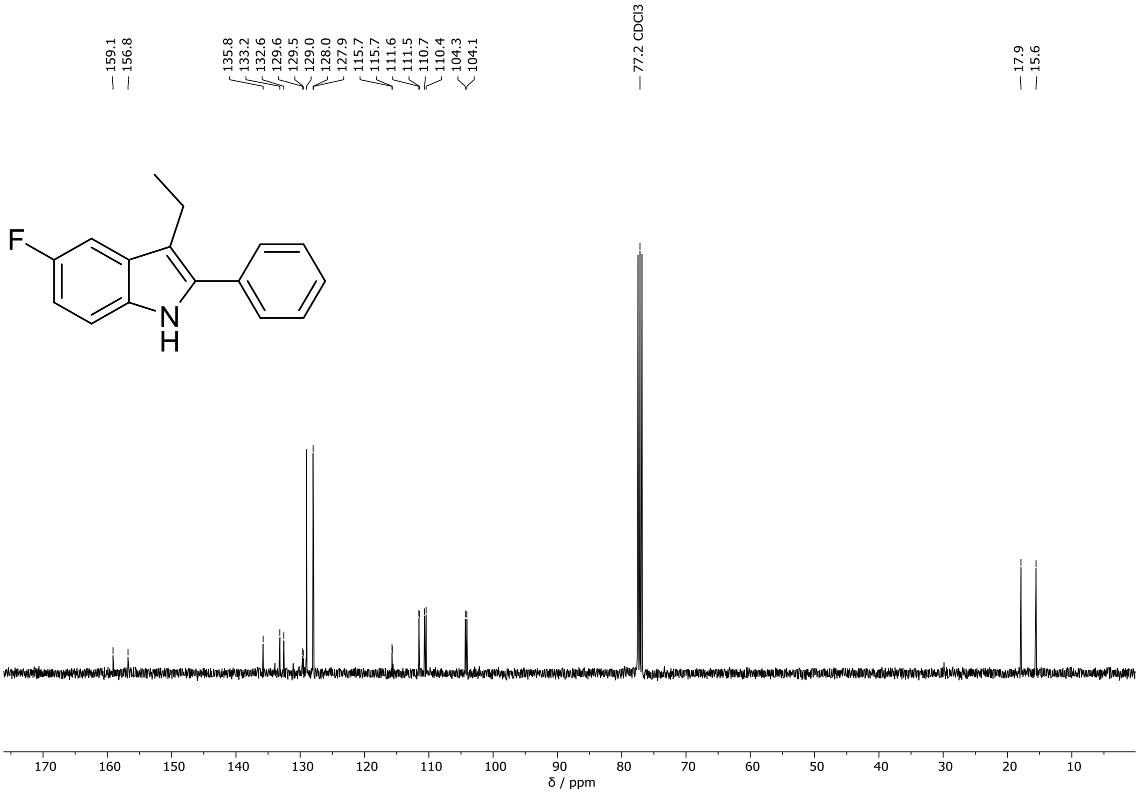

$^{19}F\{^{1}H\}$-NMR (367 MHz, $CDCl_3$)

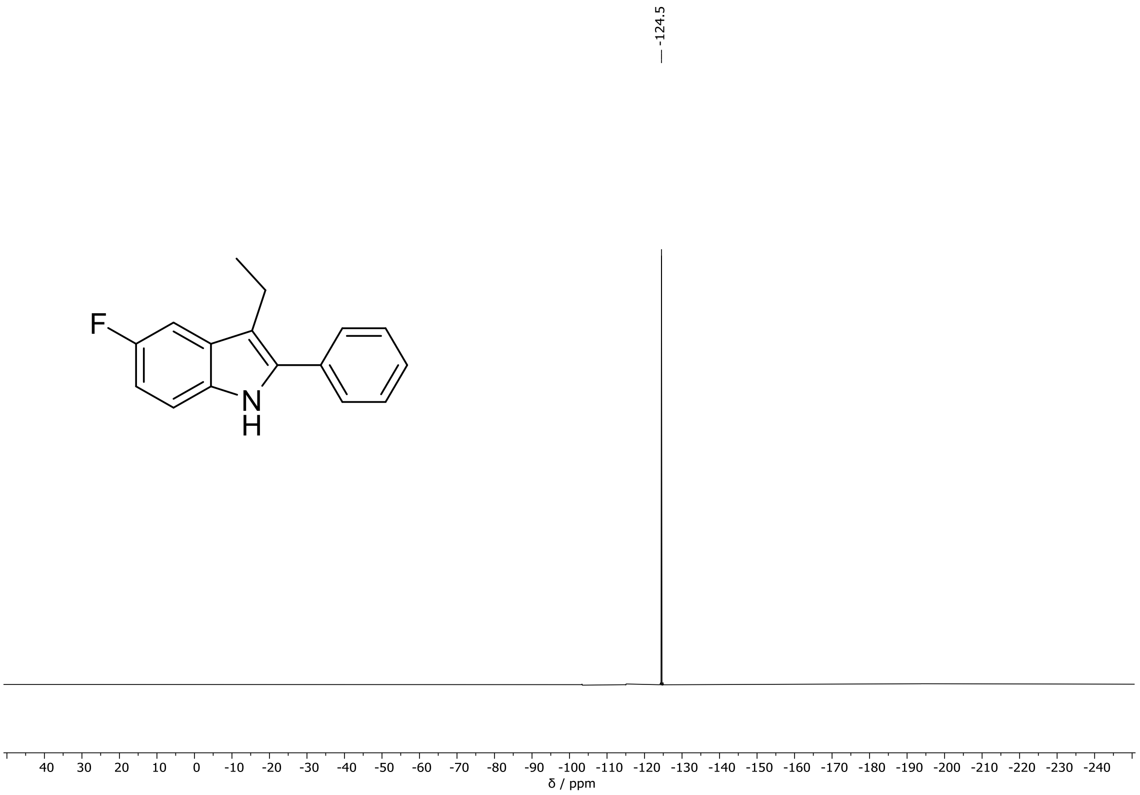

methyl 3-methyl-2-phenyl-1*H*-indole-5-carboxylate (2v):

^{1}H-NMR (400 MHz, $CDCl_3$)

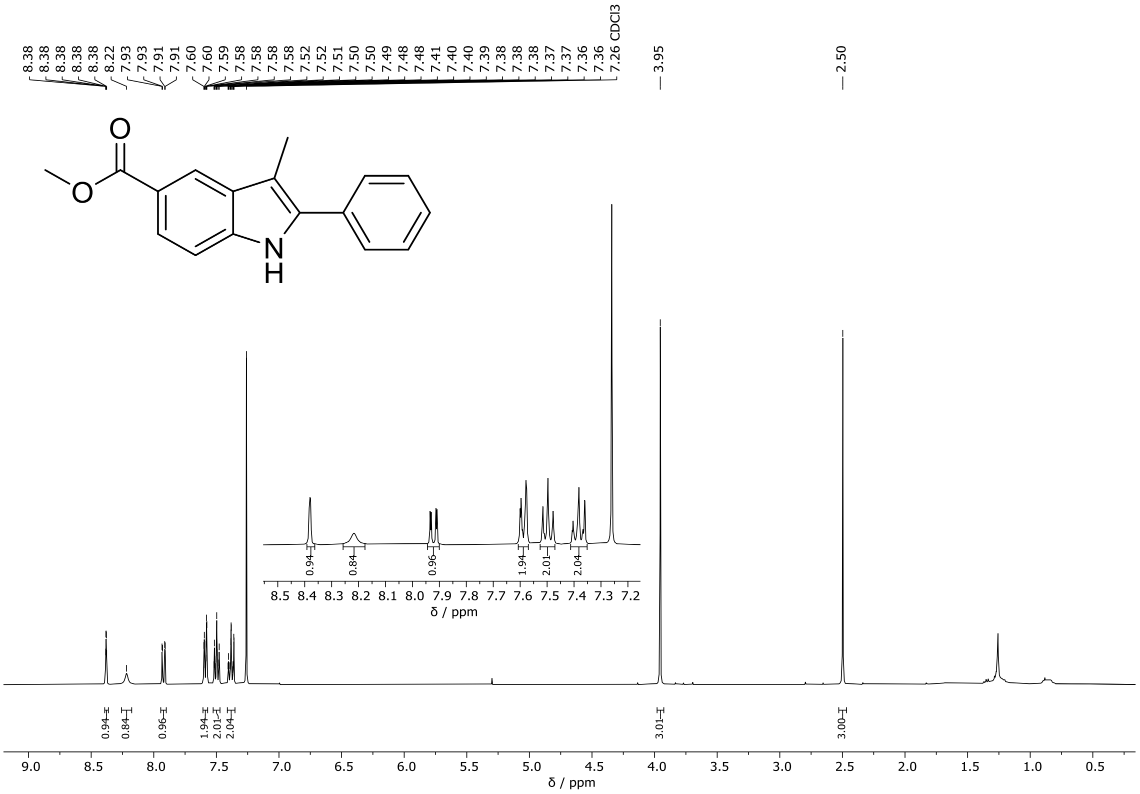

^{13}C-NMR (101 MHz, $CDCl_3$)

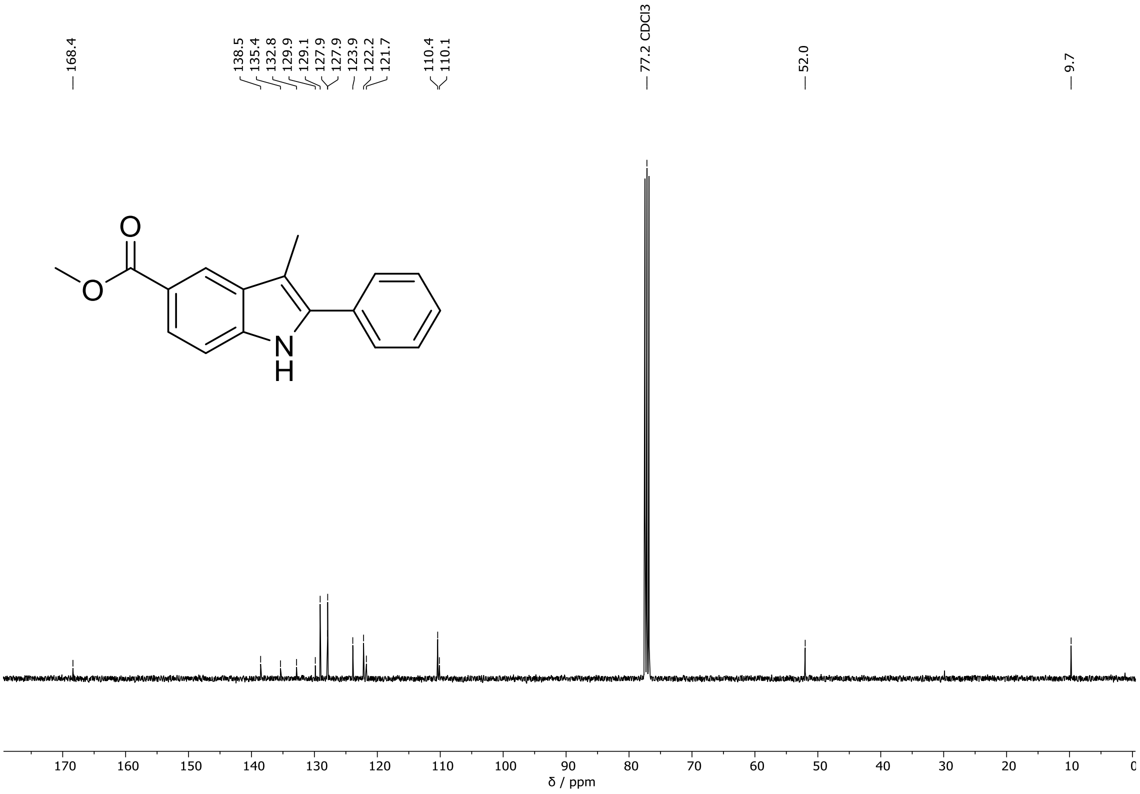

ethyl 2-(2-phenyl-1*H*-indol-3-yl)acetate (2w):

^{1}H-NMR (300 MHz, $CDCl_3$)

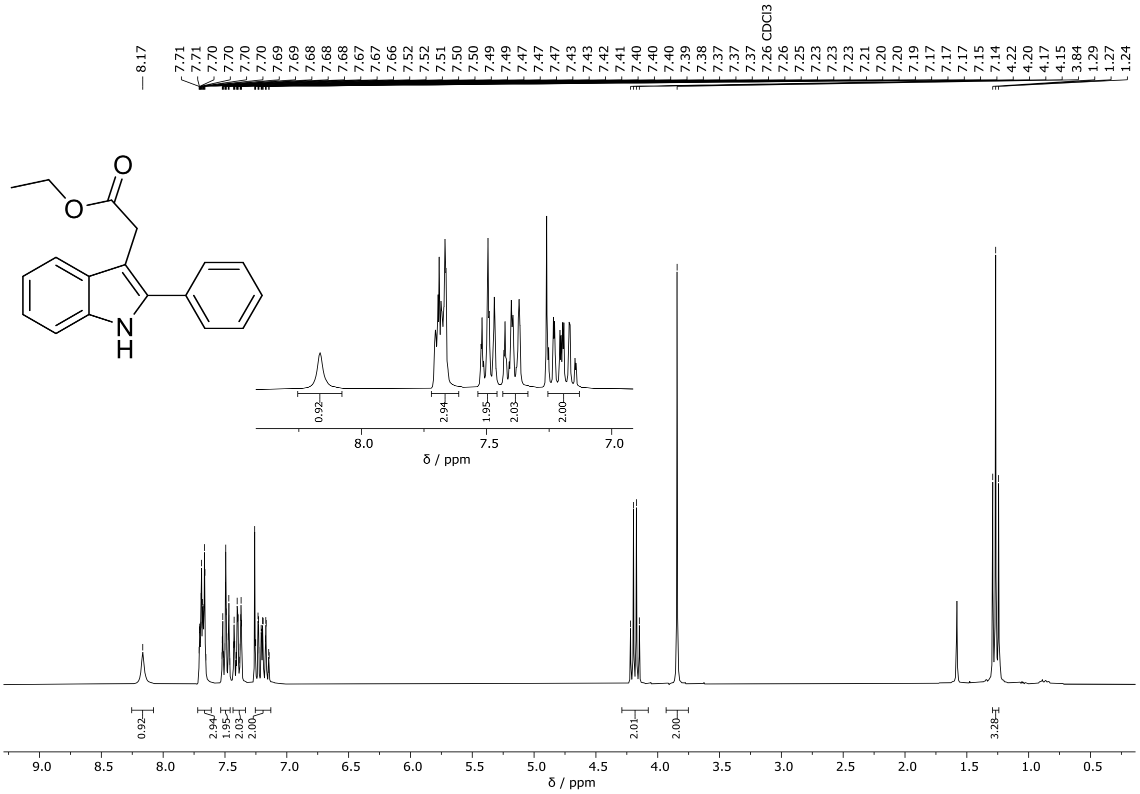

^{13}C-NMR (76 MHz, $CDCl_3$)

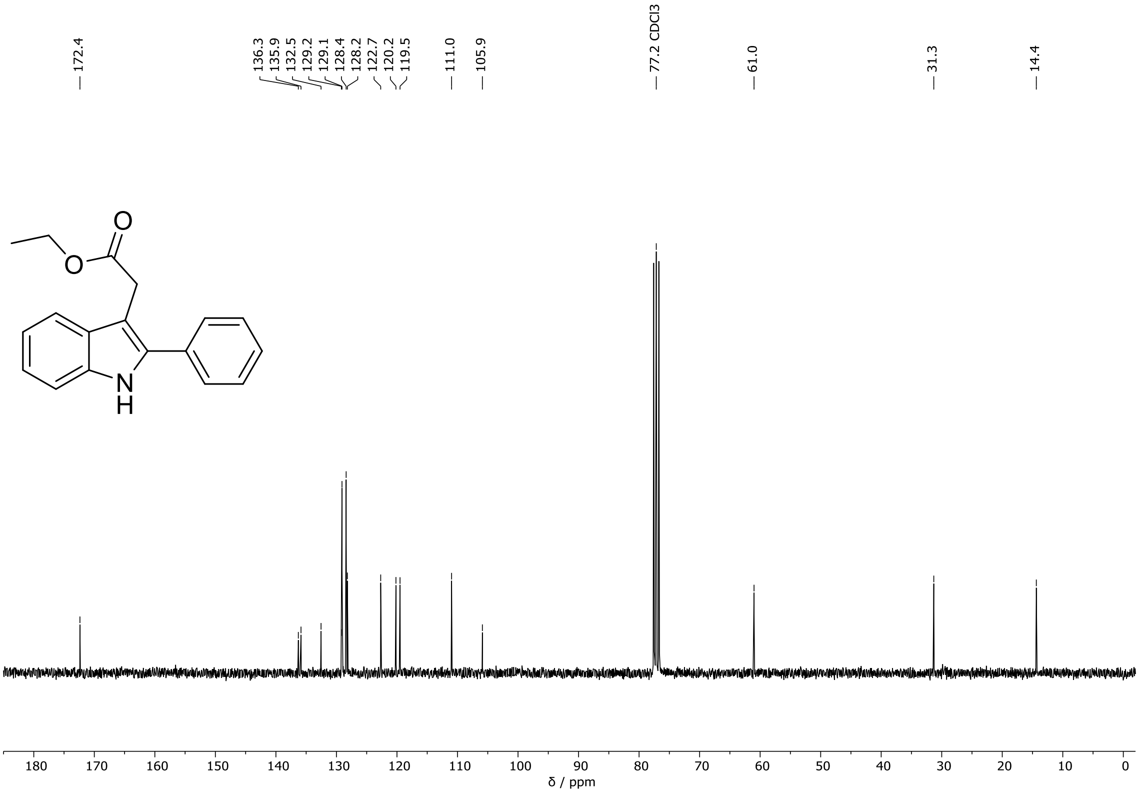

N-isopropyl-2-(2-phenyl-1H-indol-3-yl)acetamide (2x):

^{1}H-NMR (599 MHz, $CDCl_3$)

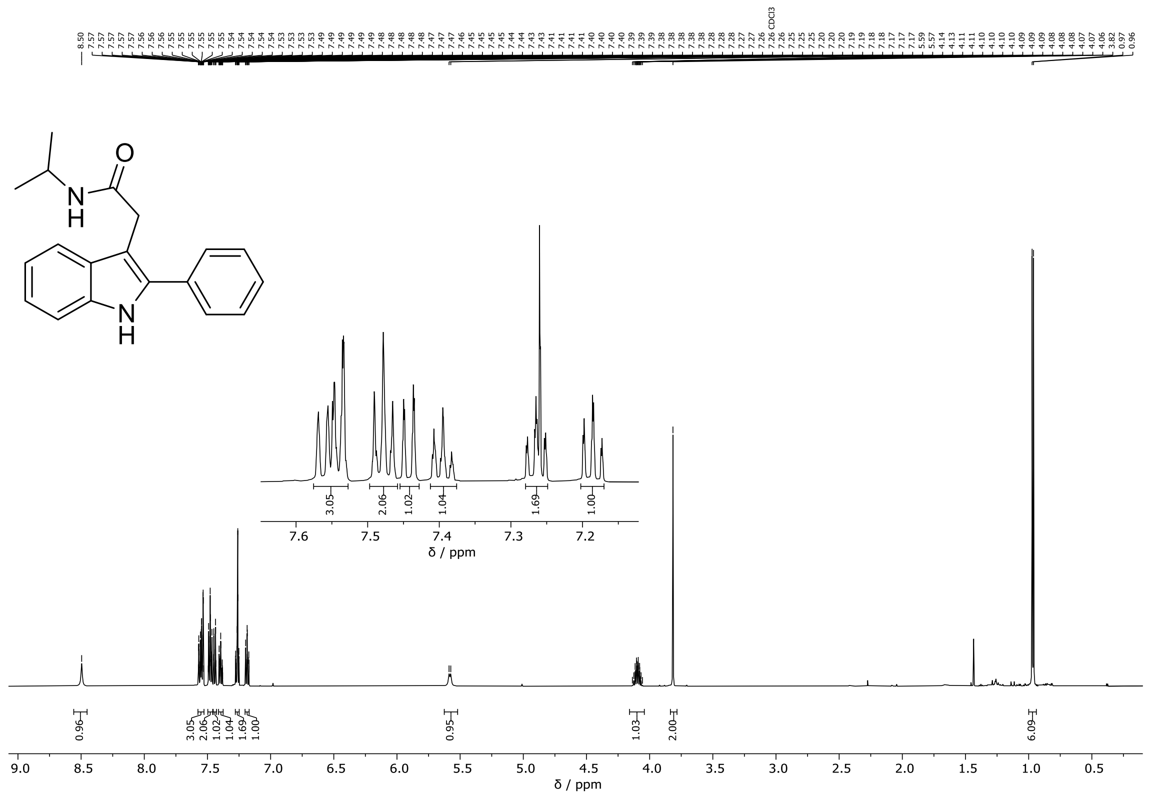

^{13}C-NMR (151 MHz, $CDCl_3$)

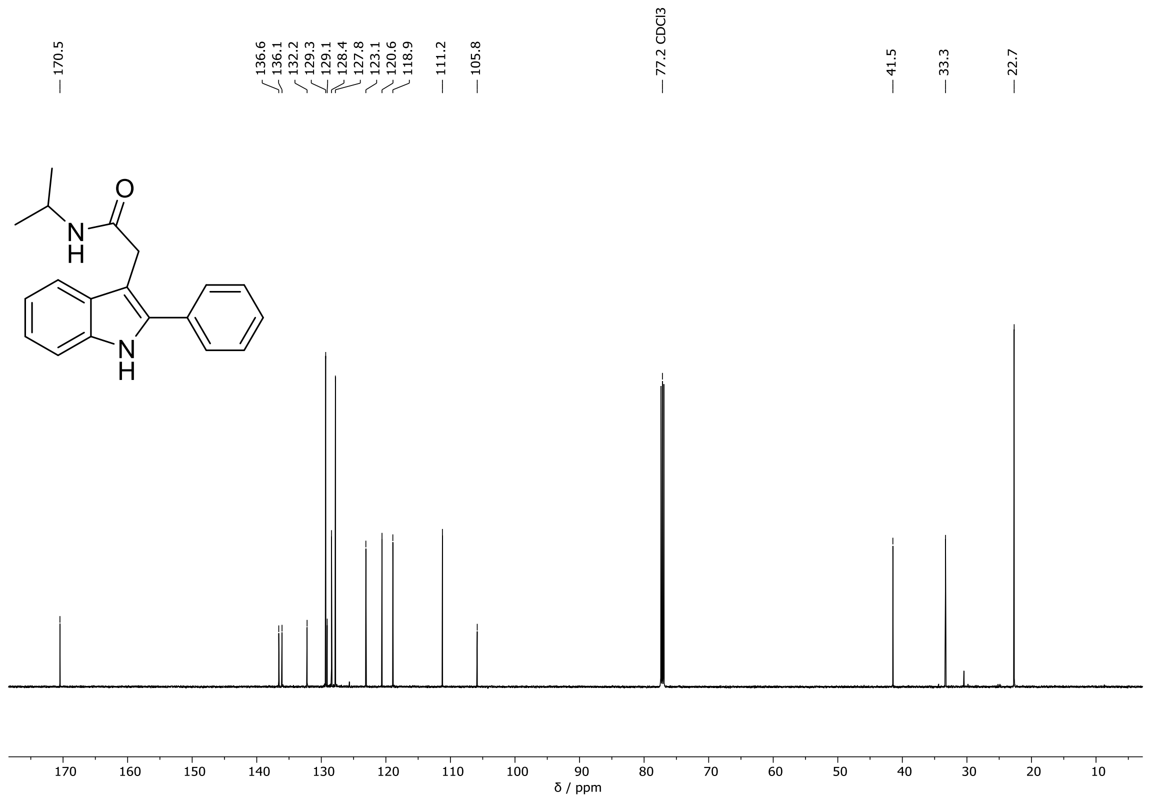

2-(2-phenyl-1*H*-indol-3-yl)-1-(pyrrolidin-1-yl)ethan-1-one (2y):

^{1}H-NMR (599 MHz, $CDCl_3$)

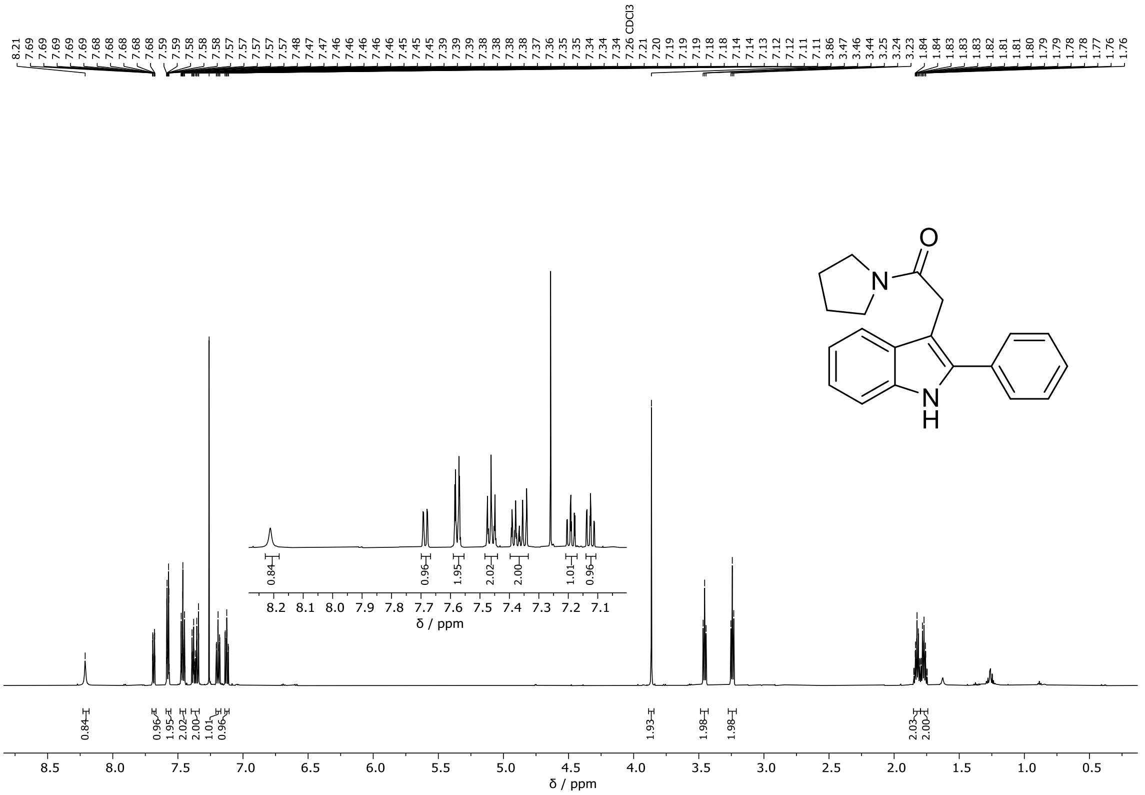

^{13}C-NMR (151 MHz, $CDCl_3$)

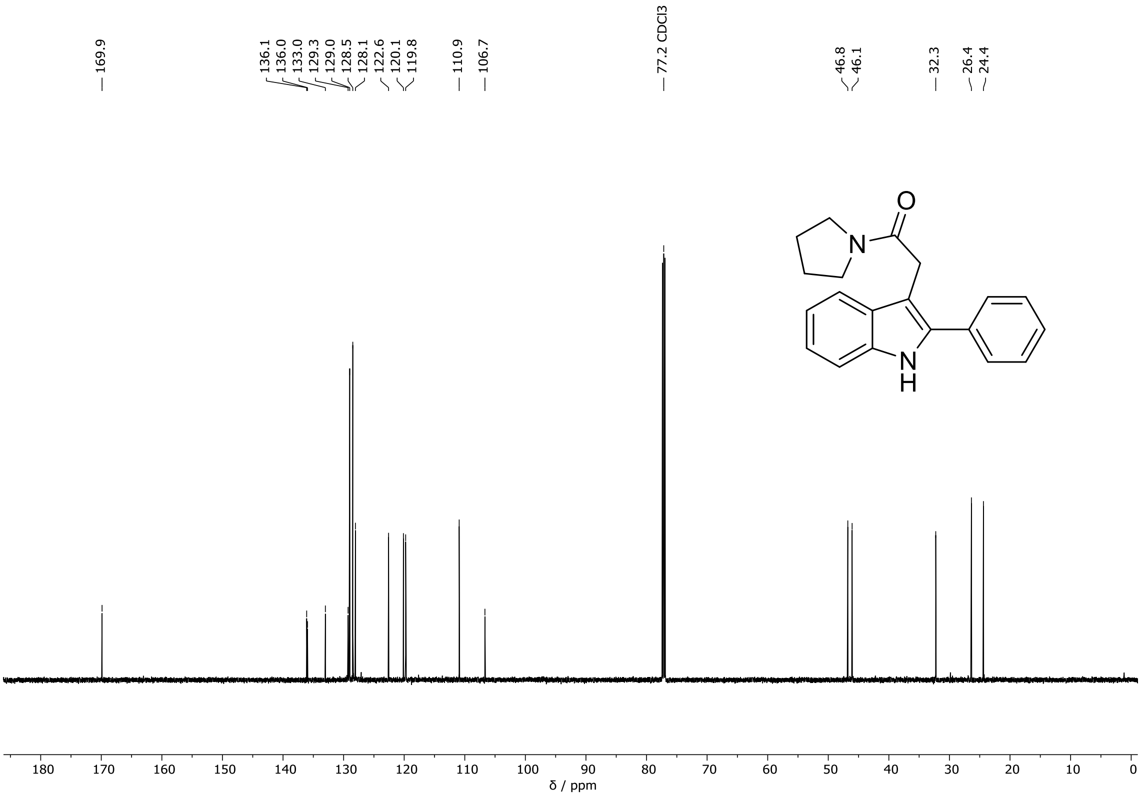

3-benzyl-2-phenyl-1*H*-indole (2z):

^{1}H-NMR (400 MHz, $CDCl_3$)

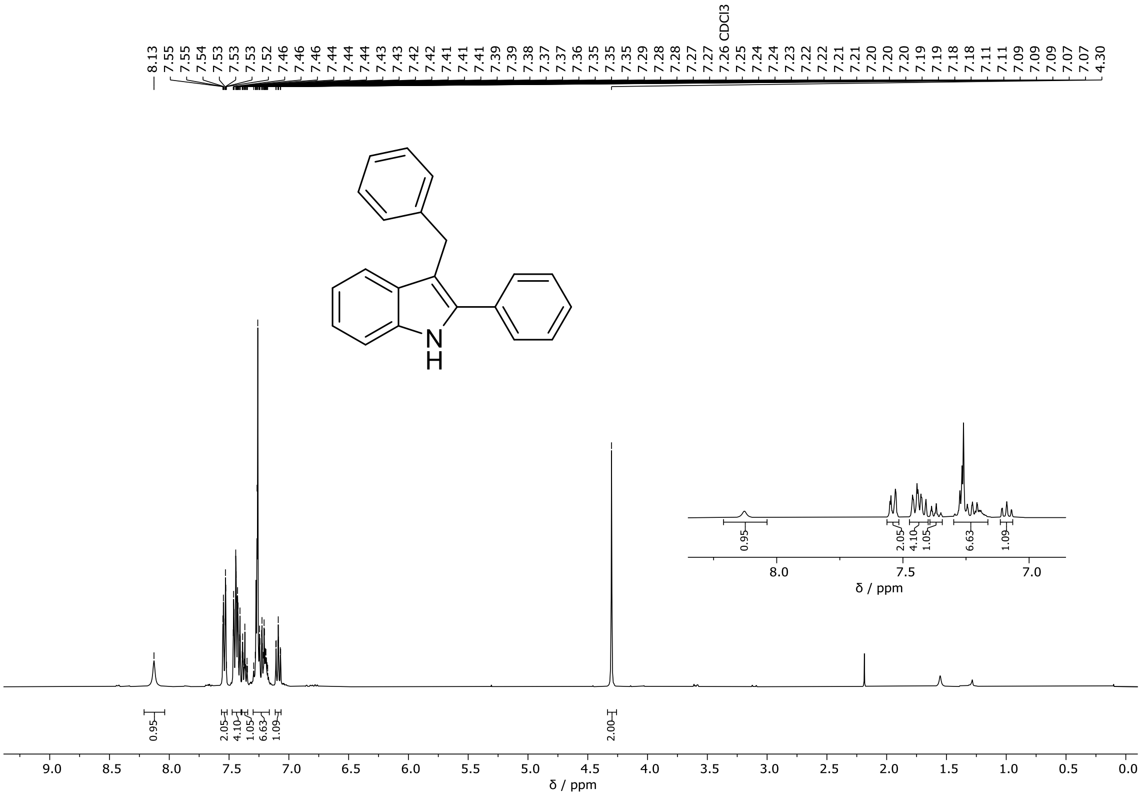

^{13}C-NMR (101 MHz, $CDCl_3$)

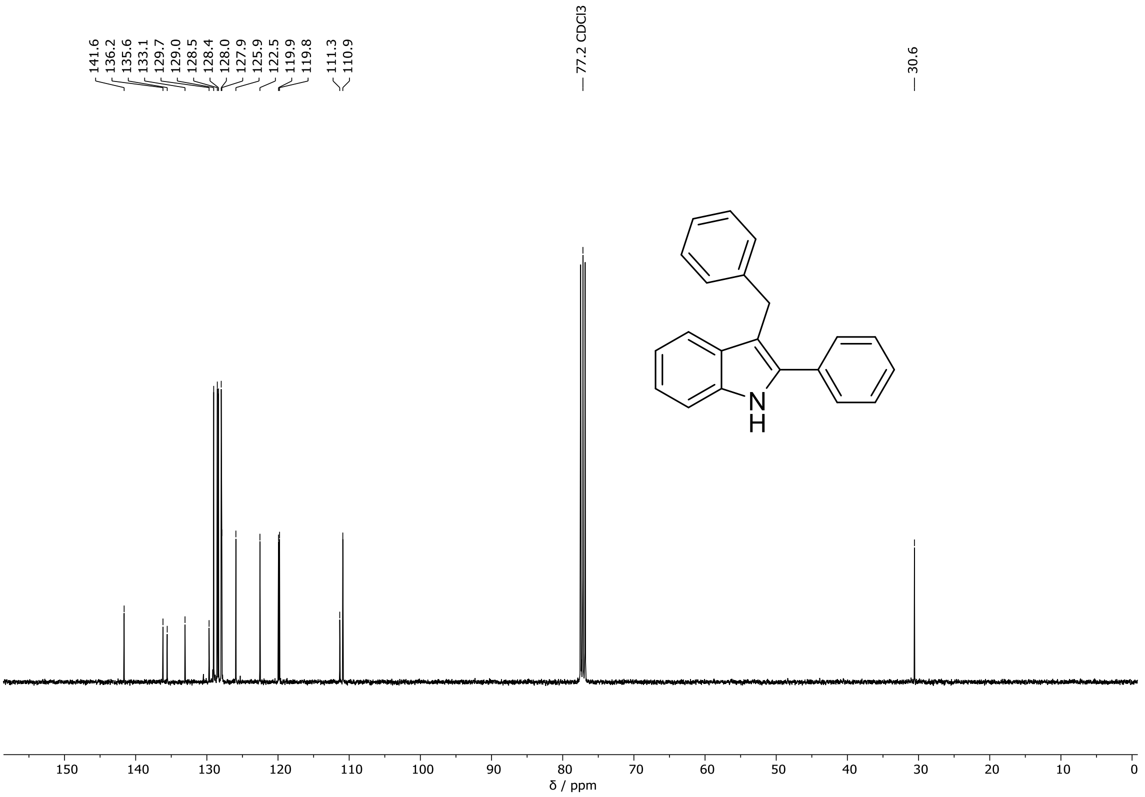

3-benzyl-2-isopropyl-1*H*-indole (2aa):

^{1}H-NMR (599 MHz, CDCl$_3$)

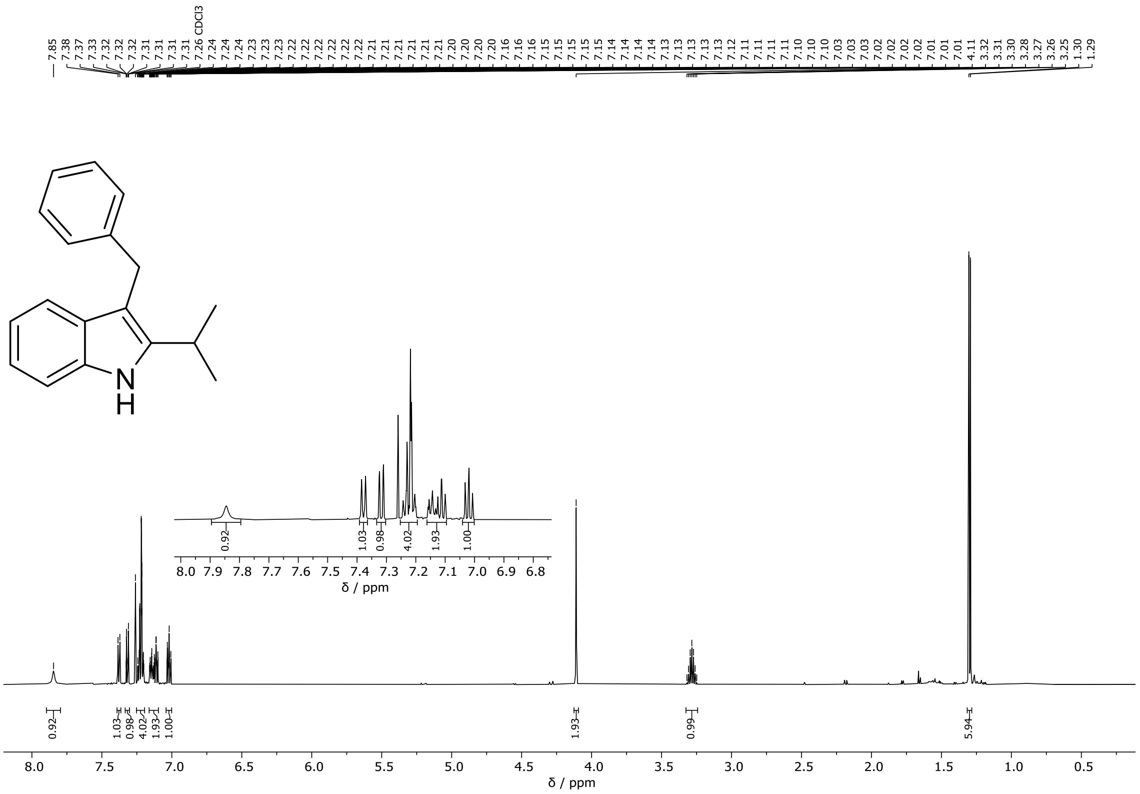

^{13}C-NMR (151 MHz, CDCl$_3$)

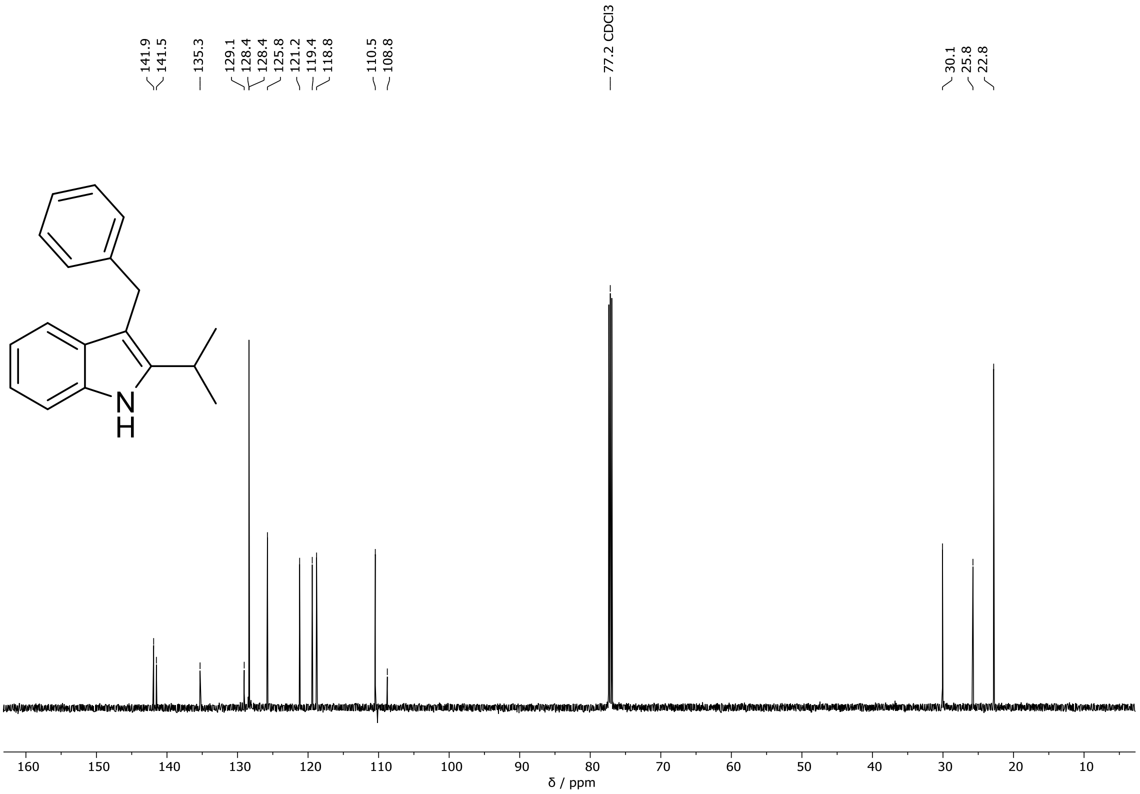

ethyl 2-(2-(tert-butyl)-1*H*-indol-3-yl)acetate (2ab):

^{1}H-NMR (599 MHz, $CDCl_3$)

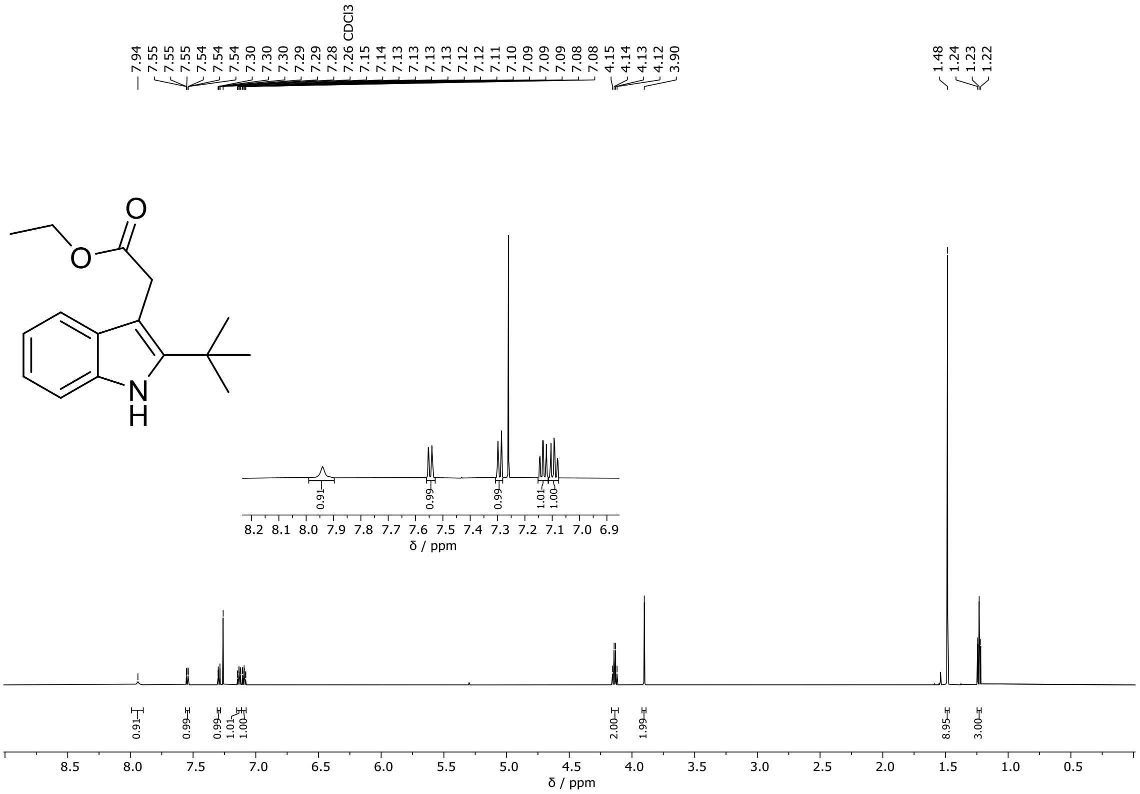

^{13}C-NMR (151 MHz, $CDCl_3$)

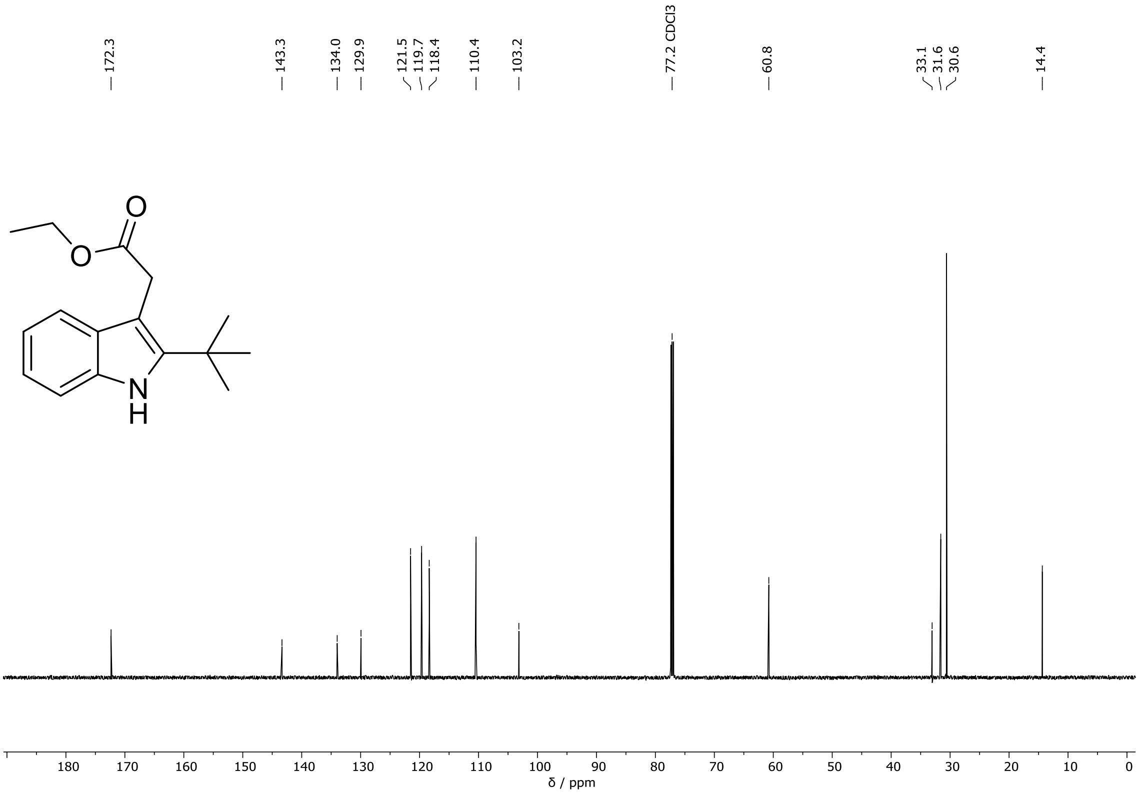

ethyl 2-(2-isopropyl-1*H*-indol-3-yl)acetate (2ac):

^{1}H-NMR (400 MHz, $CDCl_3$)

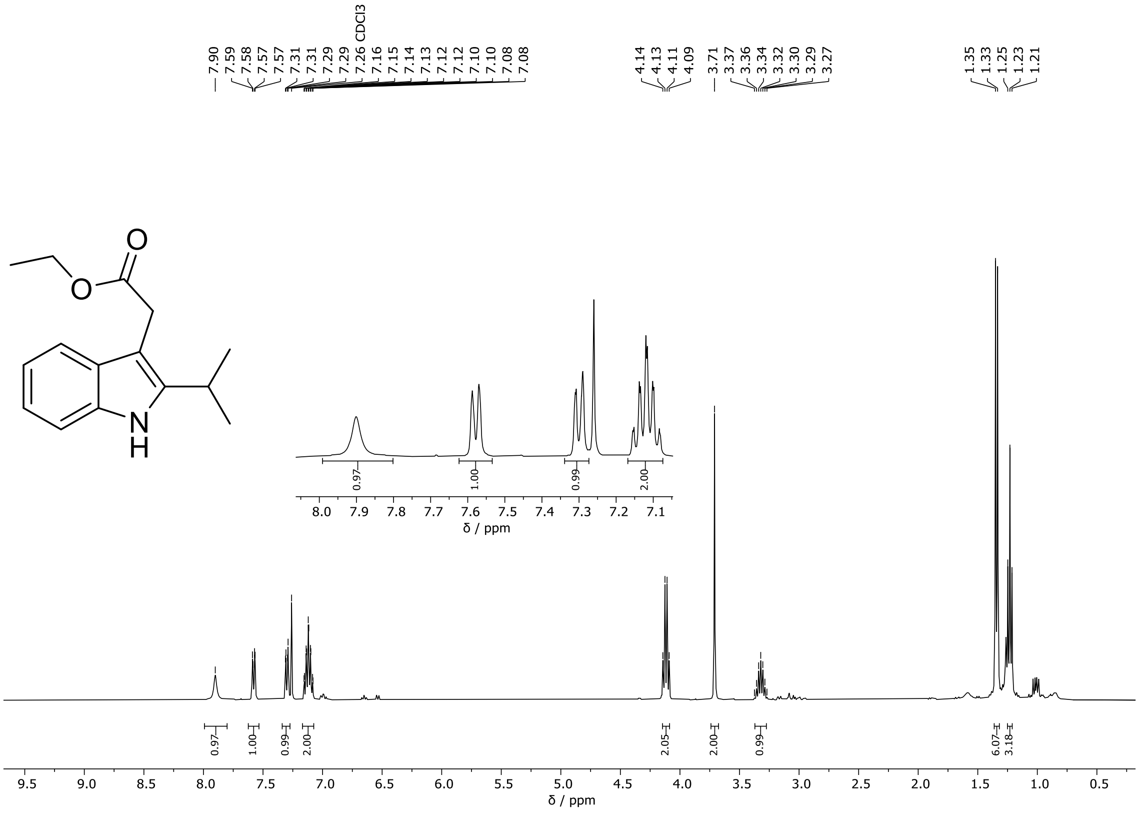

^{13}C-NMR (101 MHz, $CDCl_3$)

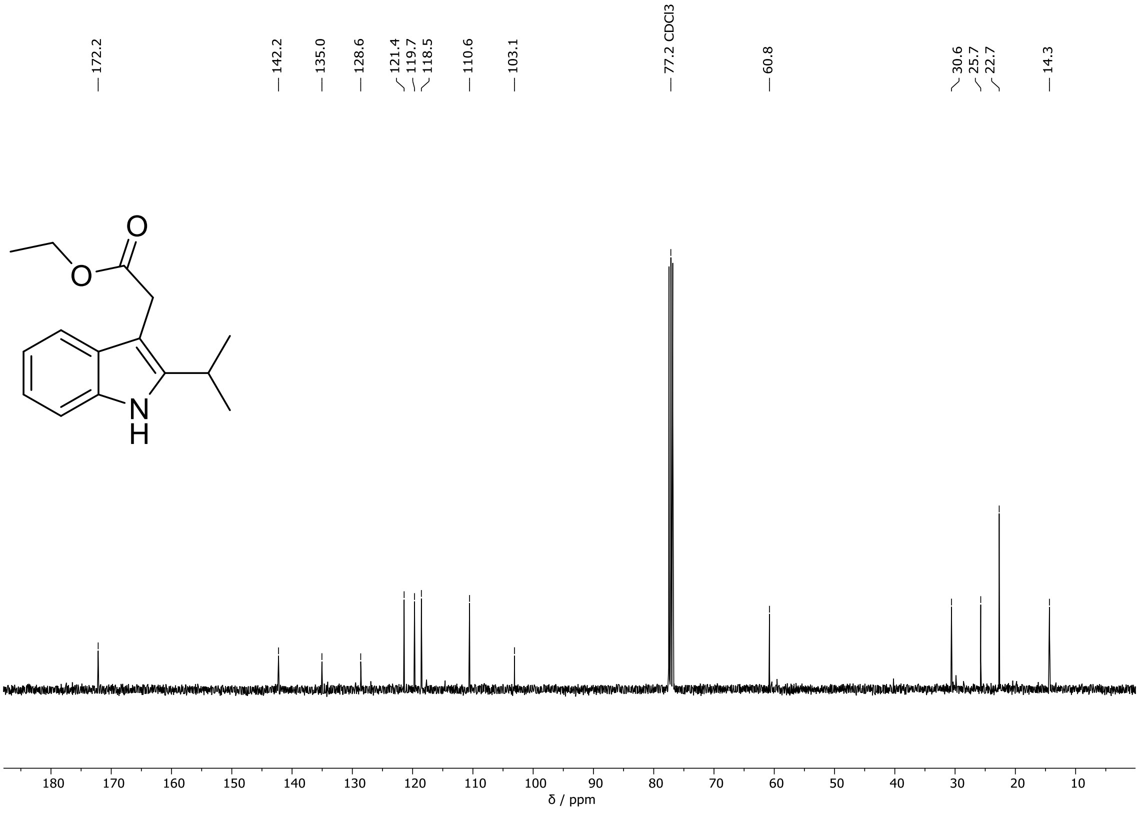

***N,N*-diethyl-2-(2-methyl-1*H*-indol-3-yl)acetamide (2ad):**

^{1}H-NMR (599 MHz, $CDCl_3$)

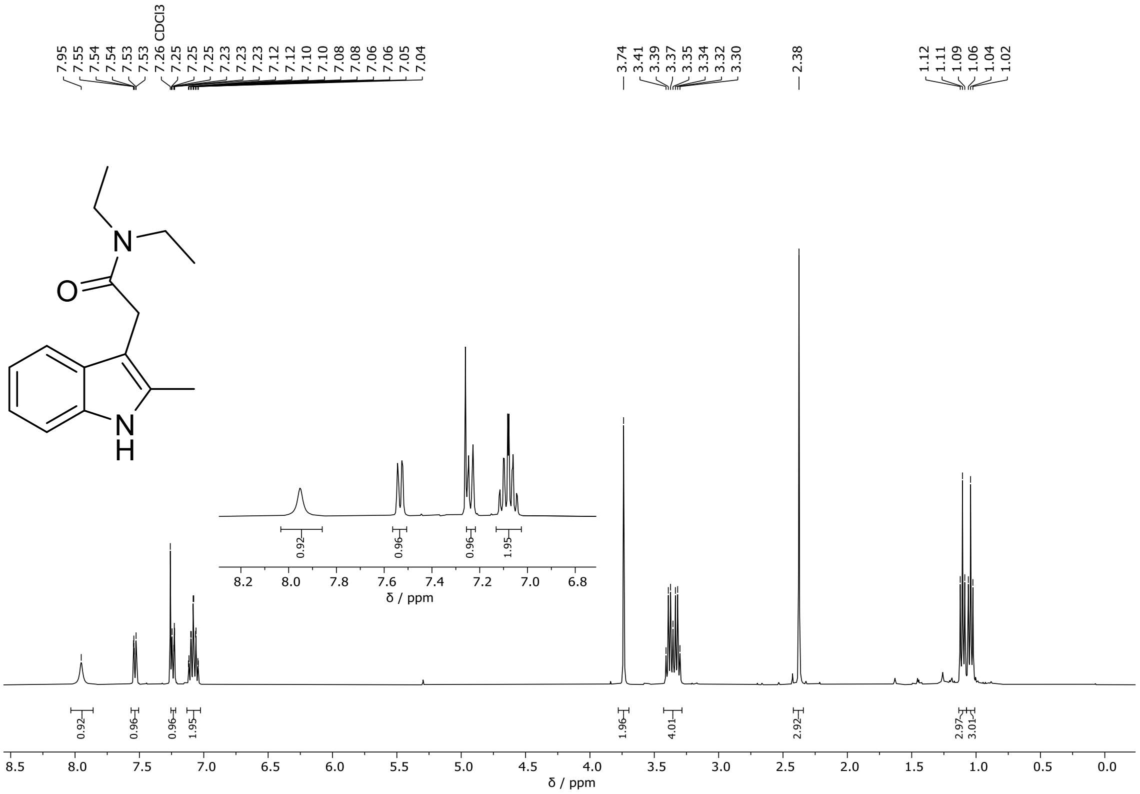

^{13}C-NMR (151 MHz, $CDCl_3$)

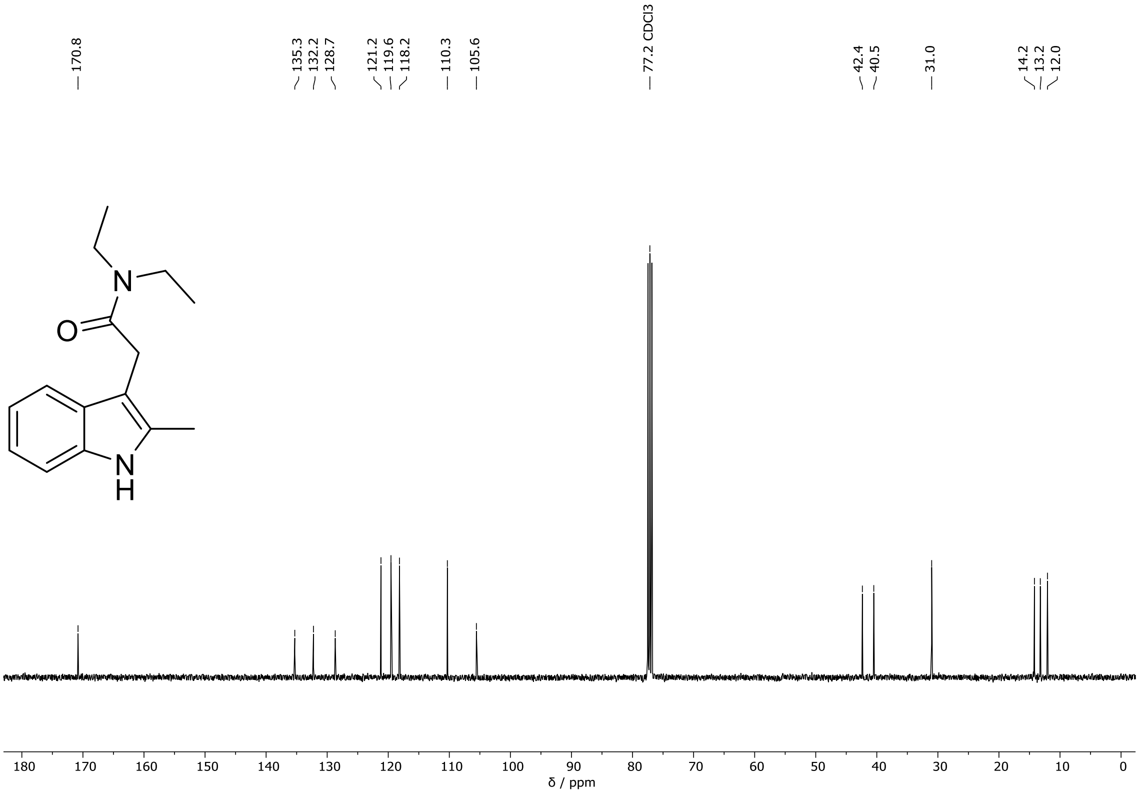

1-phenyl-2-(2-(trifluoromethyl)-1*H*-indol-3-yl)ethan-1-one (2ae):

^{1}H-NMR (500 MHz, $CDCl_3$)

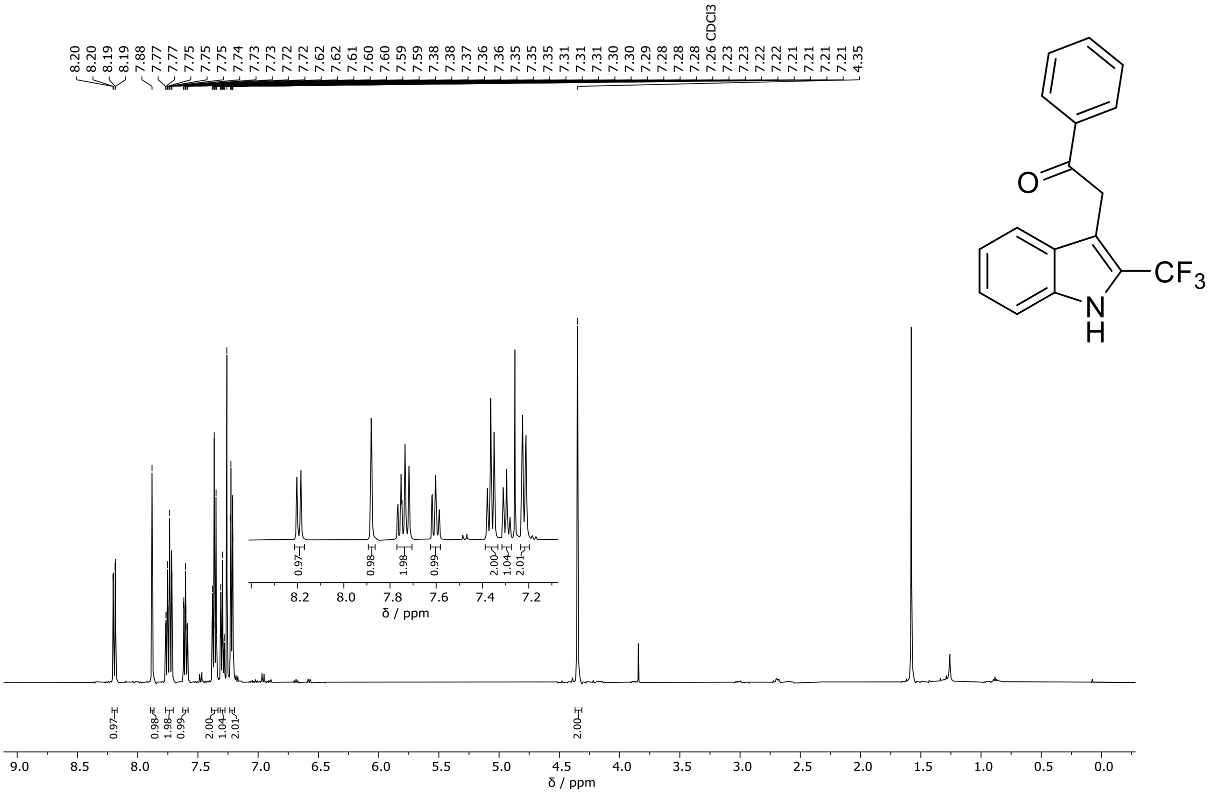

^{13}C-NMR (126 MHz, $CDCl_3$)

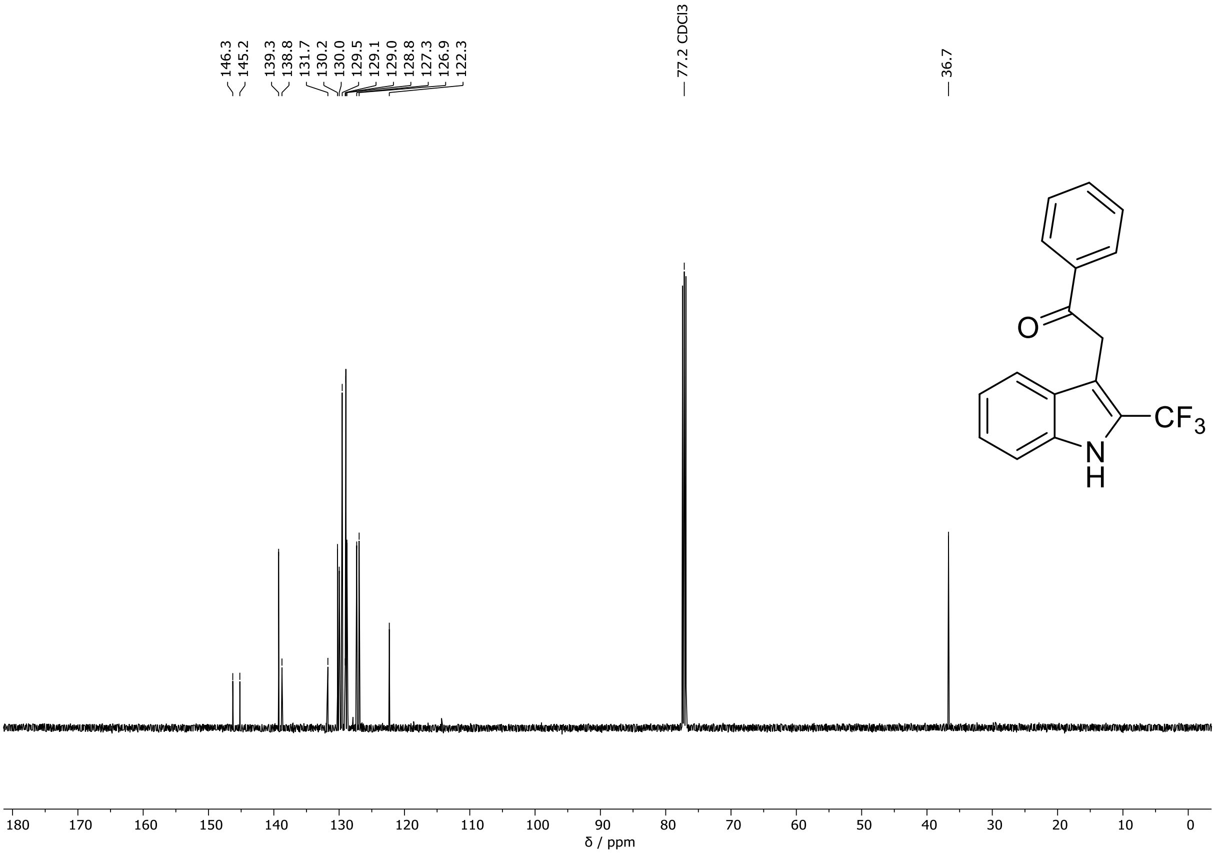

^{19}F-NMR (470 MHz, $CDCl_3$)

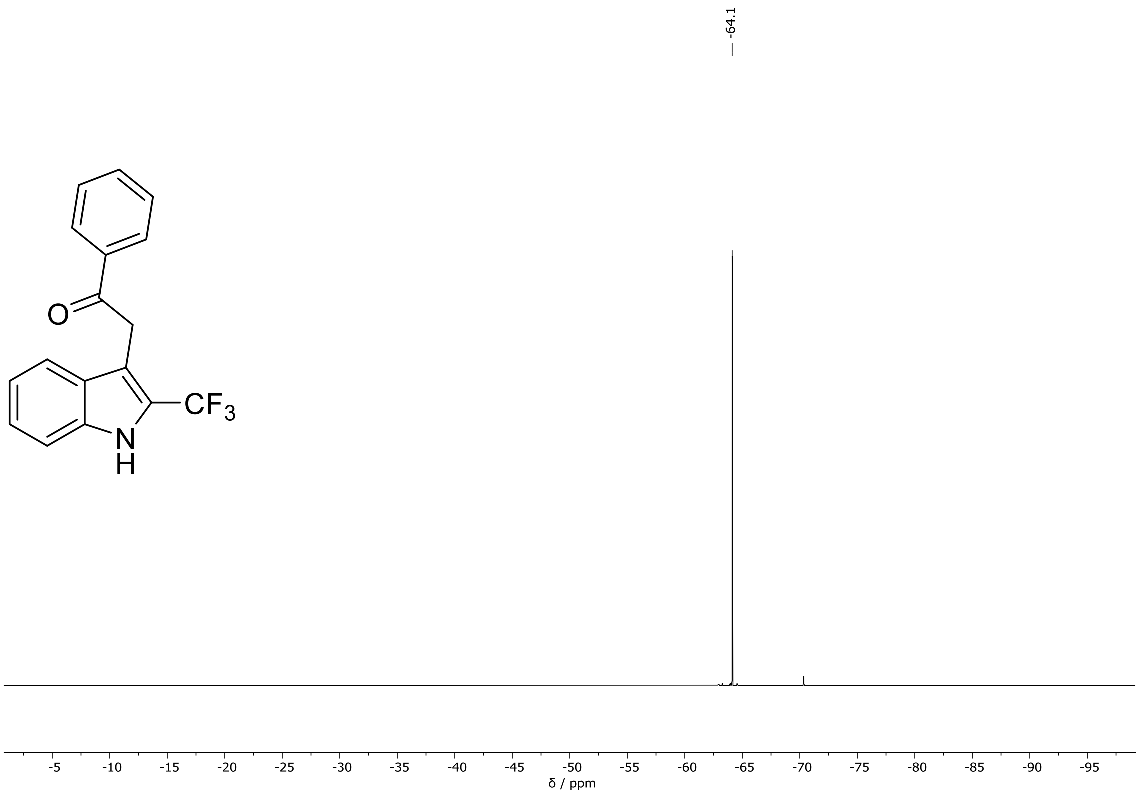

11*H*-benzo[*a*]carbazole (2af):

^{1}H-NMR (400 MHz, $CDCl_3$)

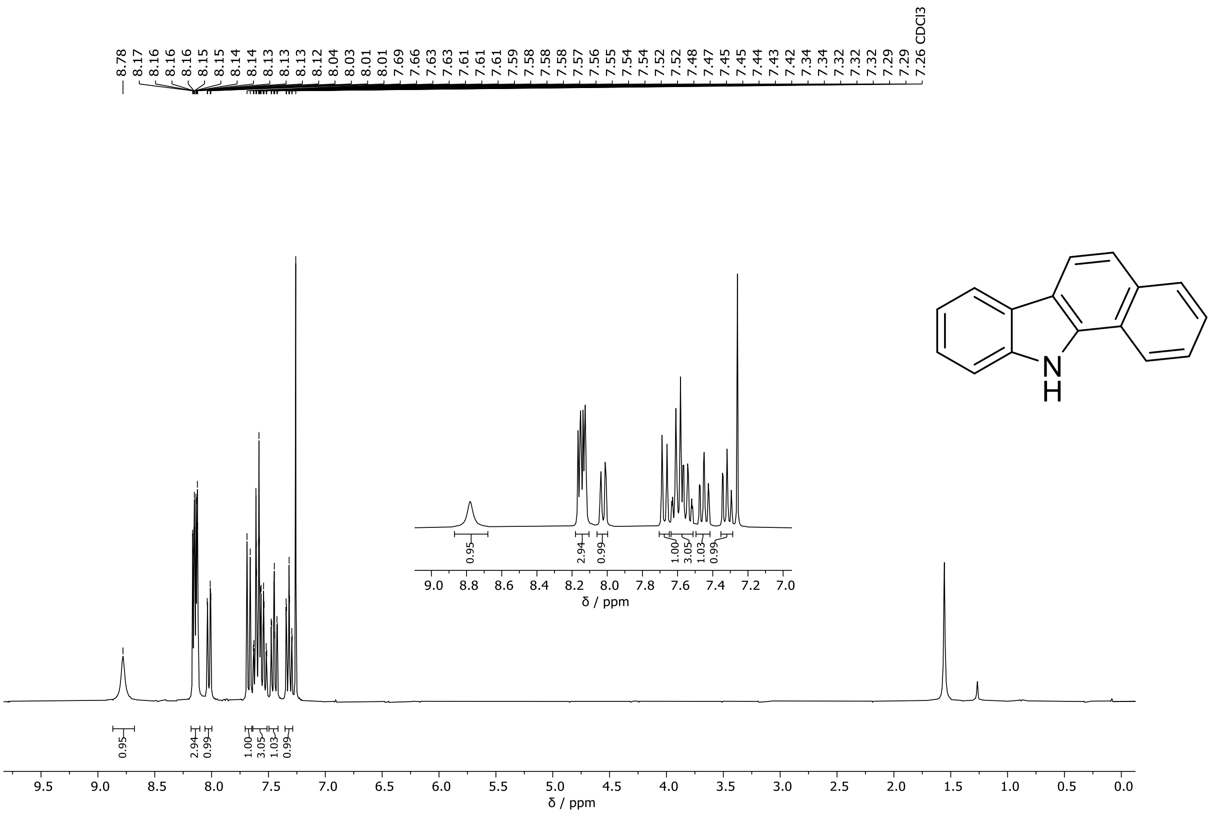

^{13}C-NMR (101 MHz, $CDCl_3$)

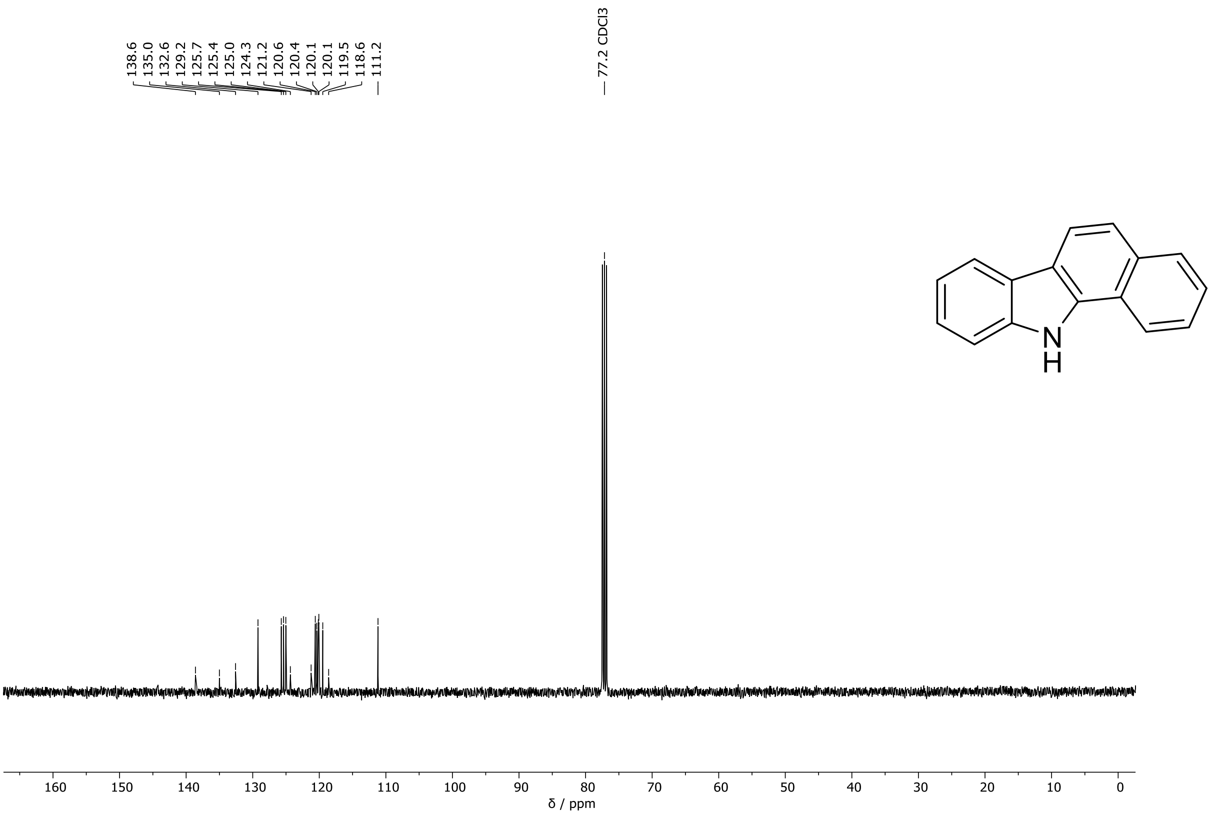

18-azatetracyclo[9.7.0.02,7.012,17]octadeca-1(11),2,4,6,12,14,16-heptaene (2ag):

^{1}H-NMR (400 MHz, $CDCl_3$)

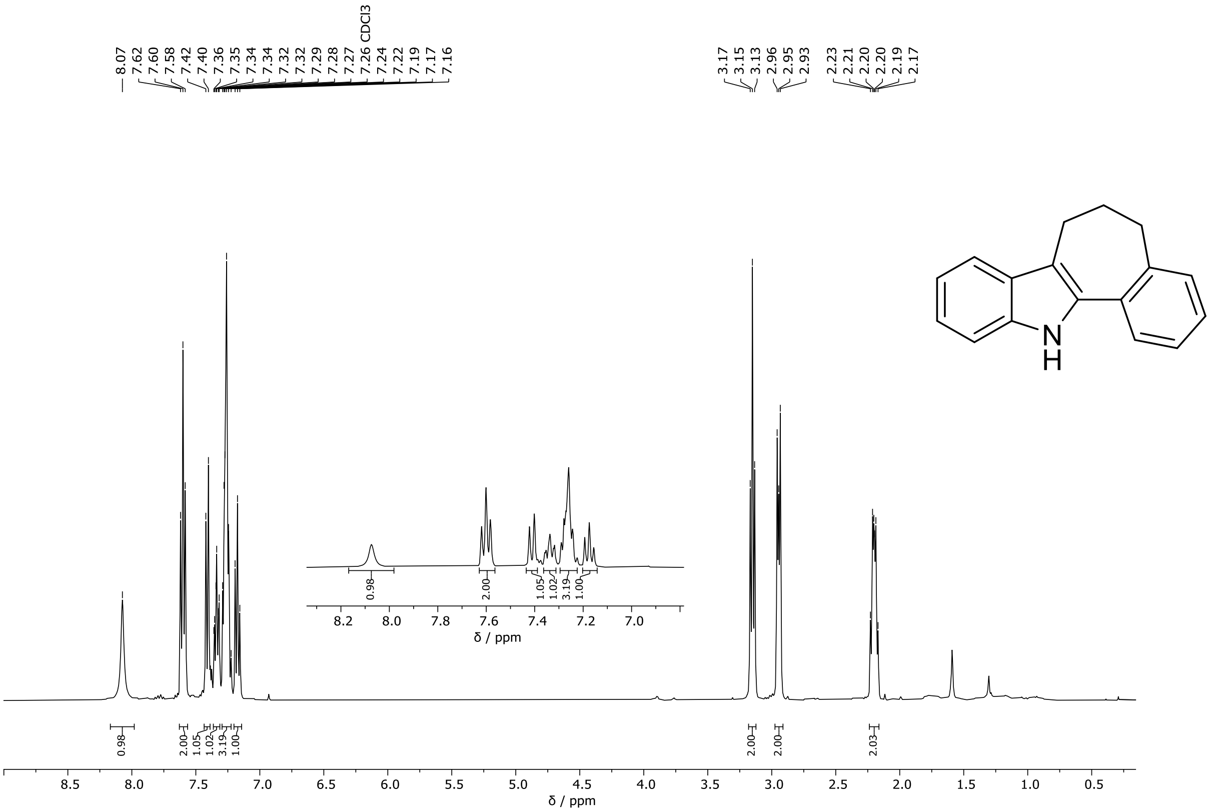

^{13}C-NMR (101 MHz, $CDCl_3$)

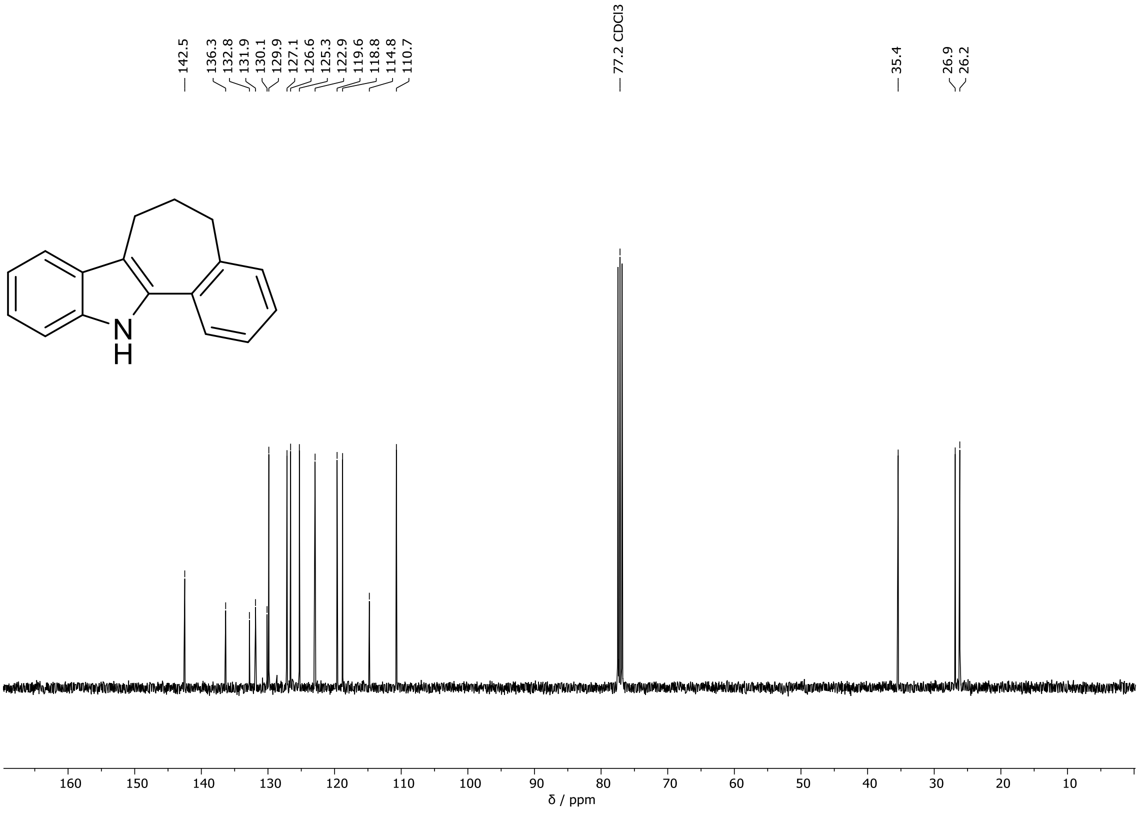

6,7,8,13-tetrahydro-5*H*-benzo[7,8]cycloocta[1,2-*b*]indole (2ah):

^{1}H-NMR (400 MHz, $CDCl_3$)

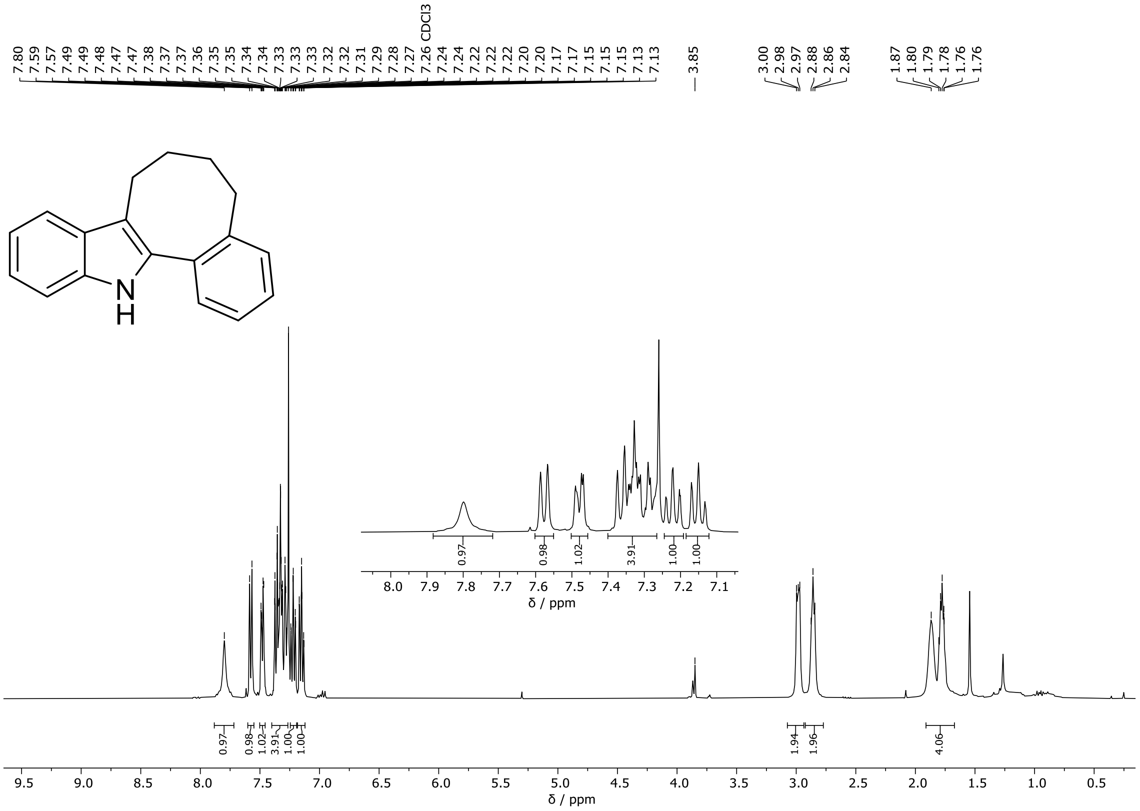

^{13}C-NMR (101 MHz, $CDCl_3$)

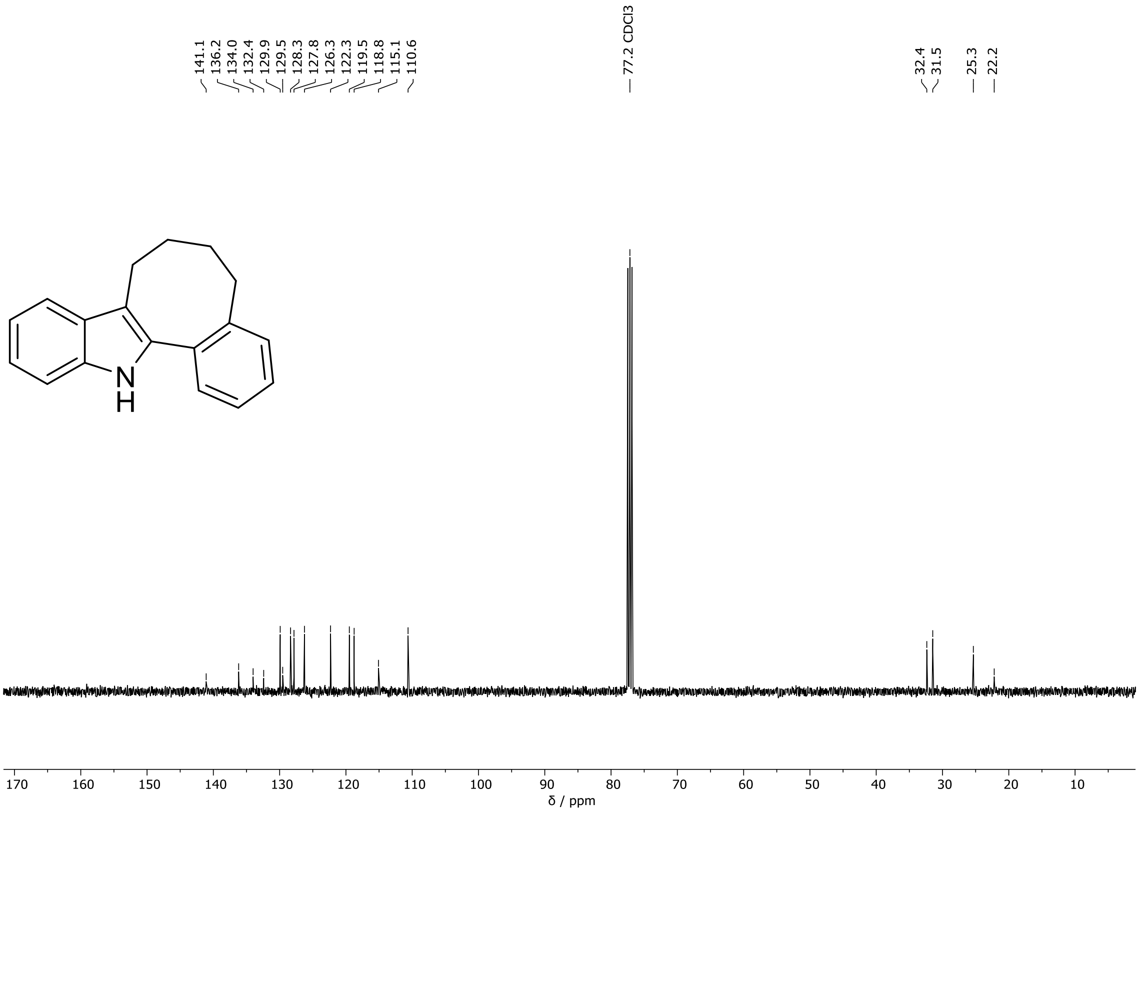

7.3. NMR Spectra of Side Products

2-phenyl-1*H*-indole (3y):

^{1}H-NMR (599 MHz, $CDCl_3$)

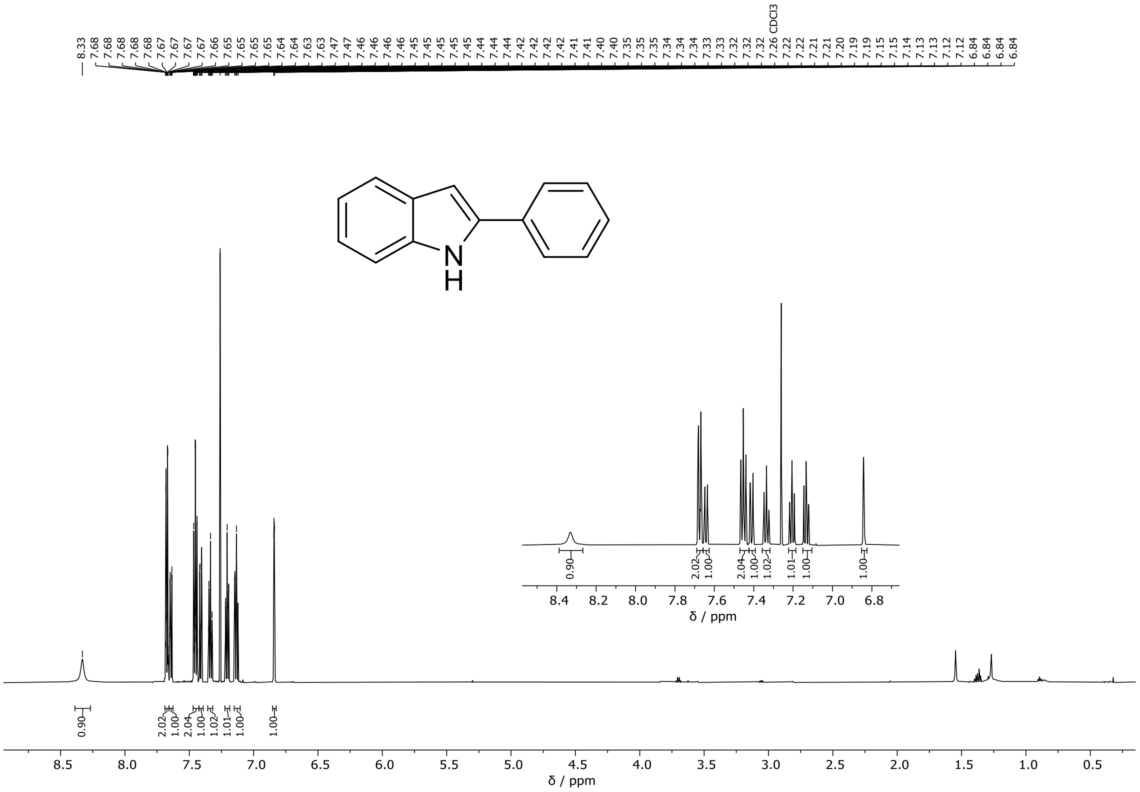

^{13}C-NMR (151 MHz, $CDCl_3$)

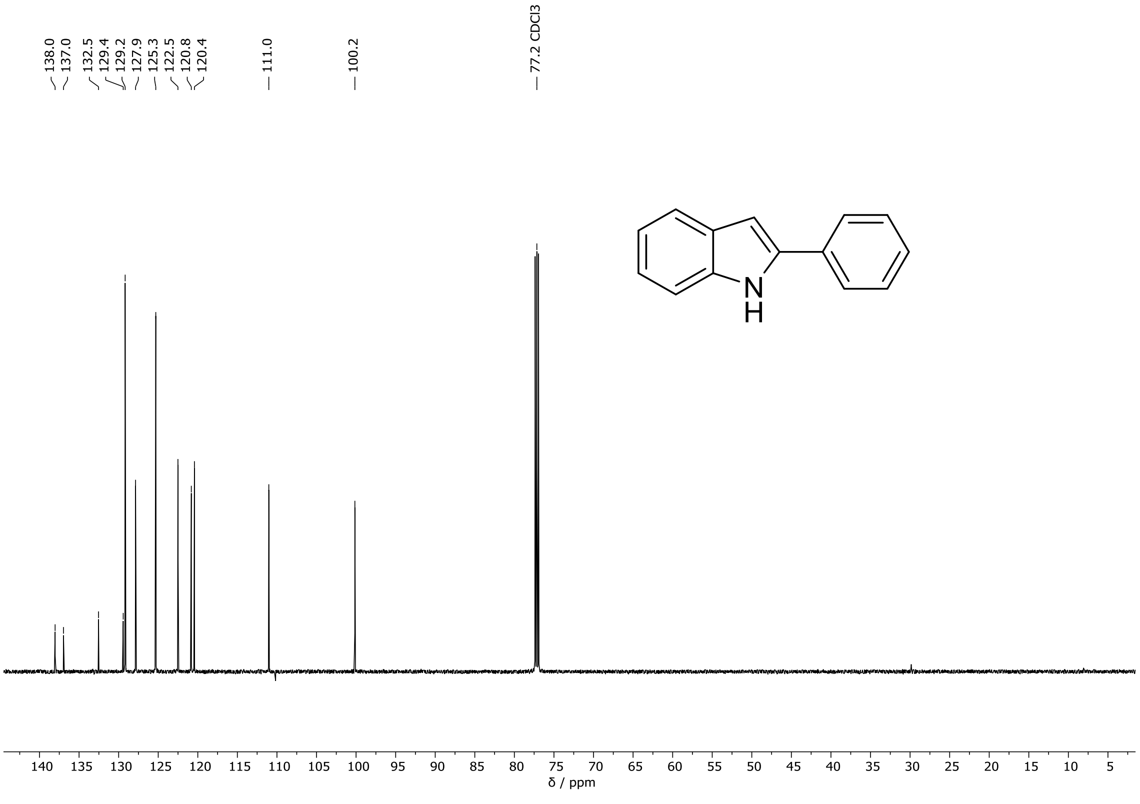

3-isopropyl-2-phenyl-5,10-dihydrobenzo[*g*]quinoline (3ai):

^{1}H-NMR (500 MHz, $CDCl_3$)

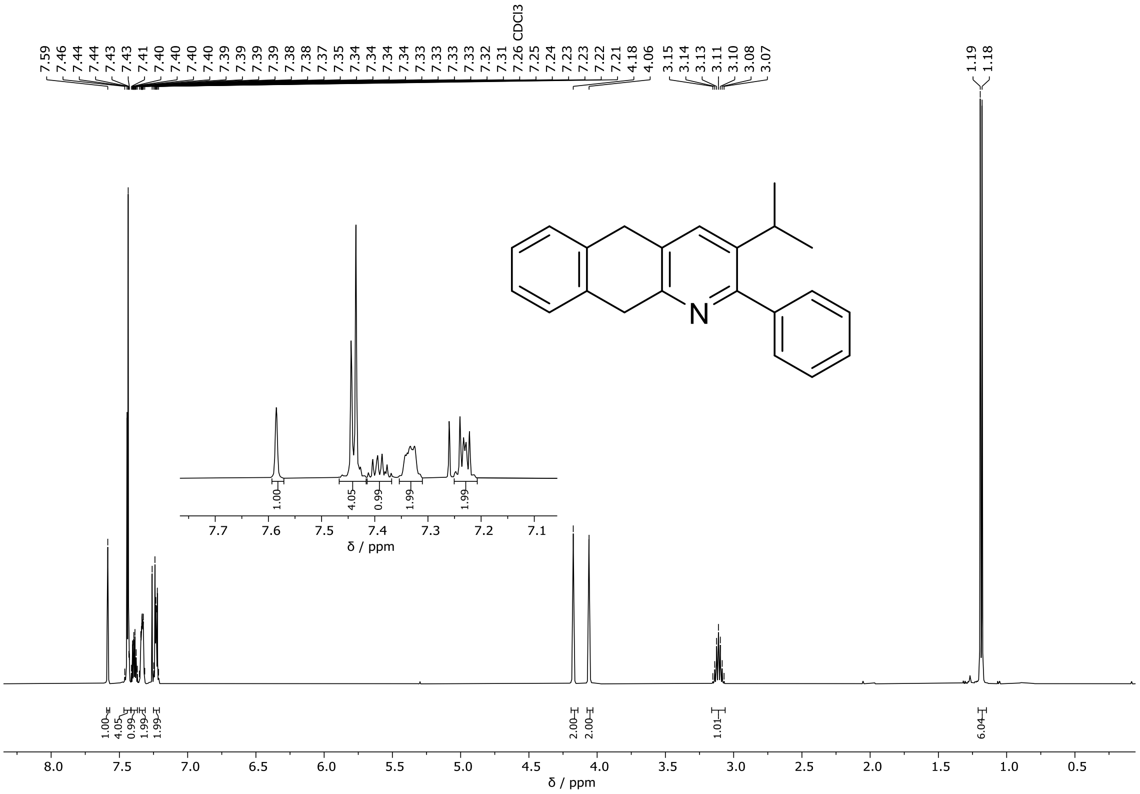

^{13}C-NMR (126 MHz, $CDCl_3$)

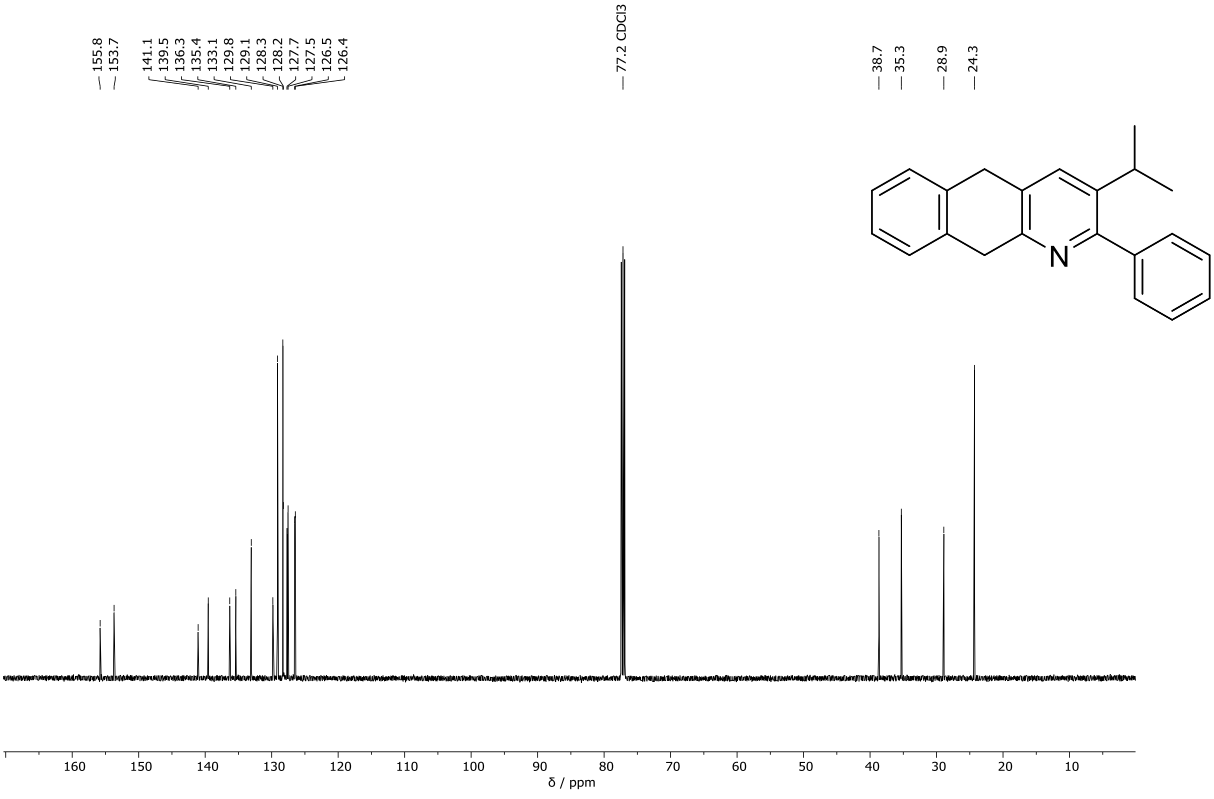

cis-6-methoxy-3-methyl-2-phenyl-1,2,3,4-tetrahydroquinoline (3aj):

^{1}H-NMR (400 MHz, $CDCl_3$)

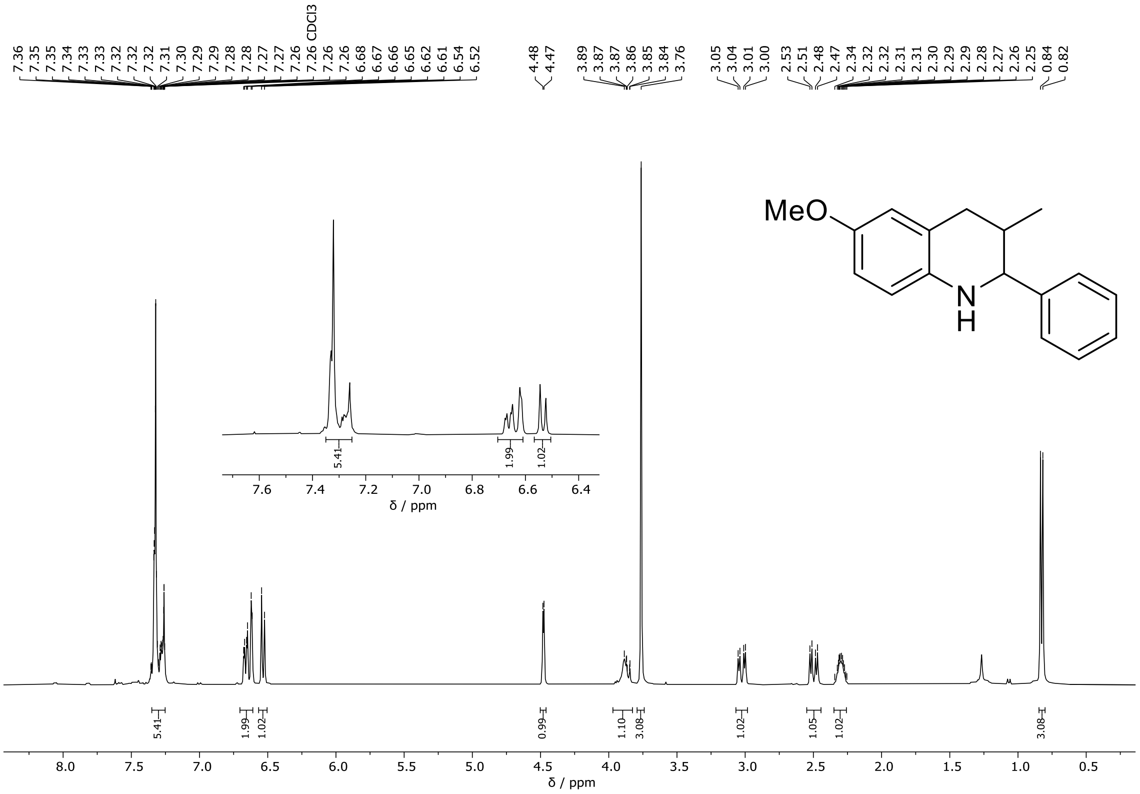

^{13}C-NMR (101 MHz, $CDCl_3$)

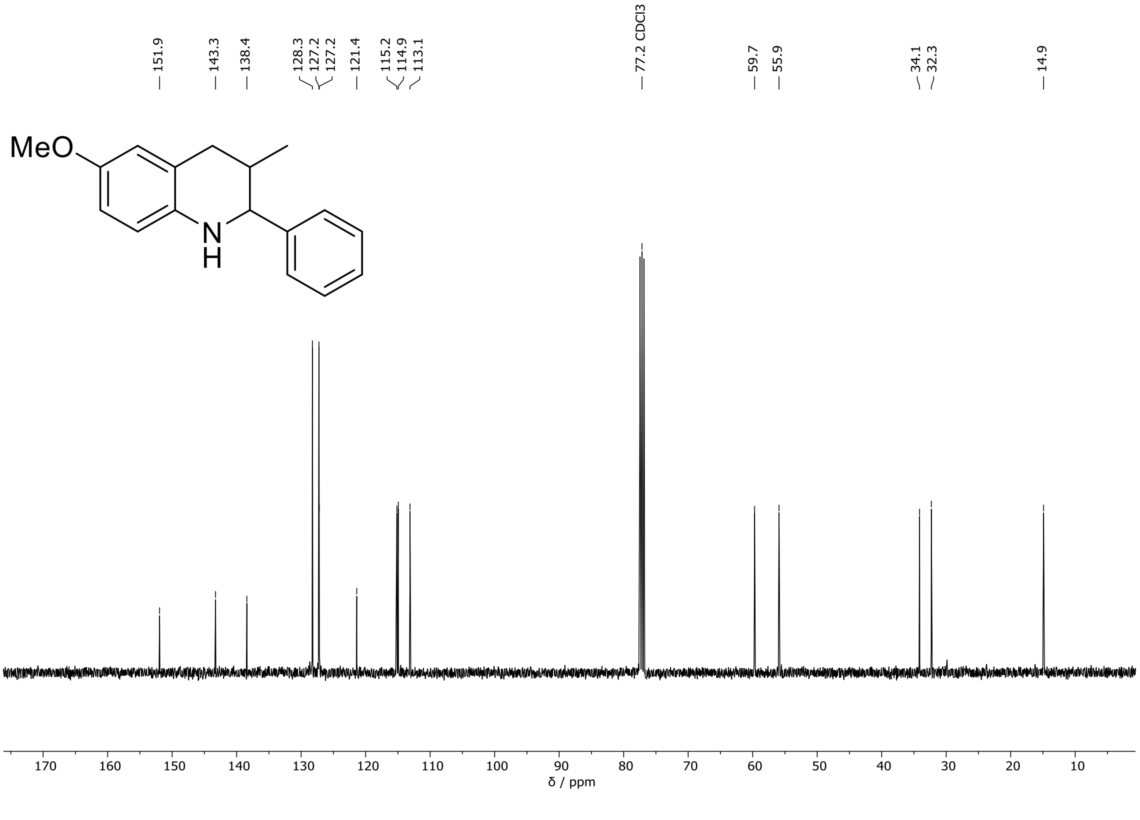

3-methyl-2-phenyl-1*H*-indole (3ak):

^{1}H-NMR (400 MHz, $CDCl_3$)

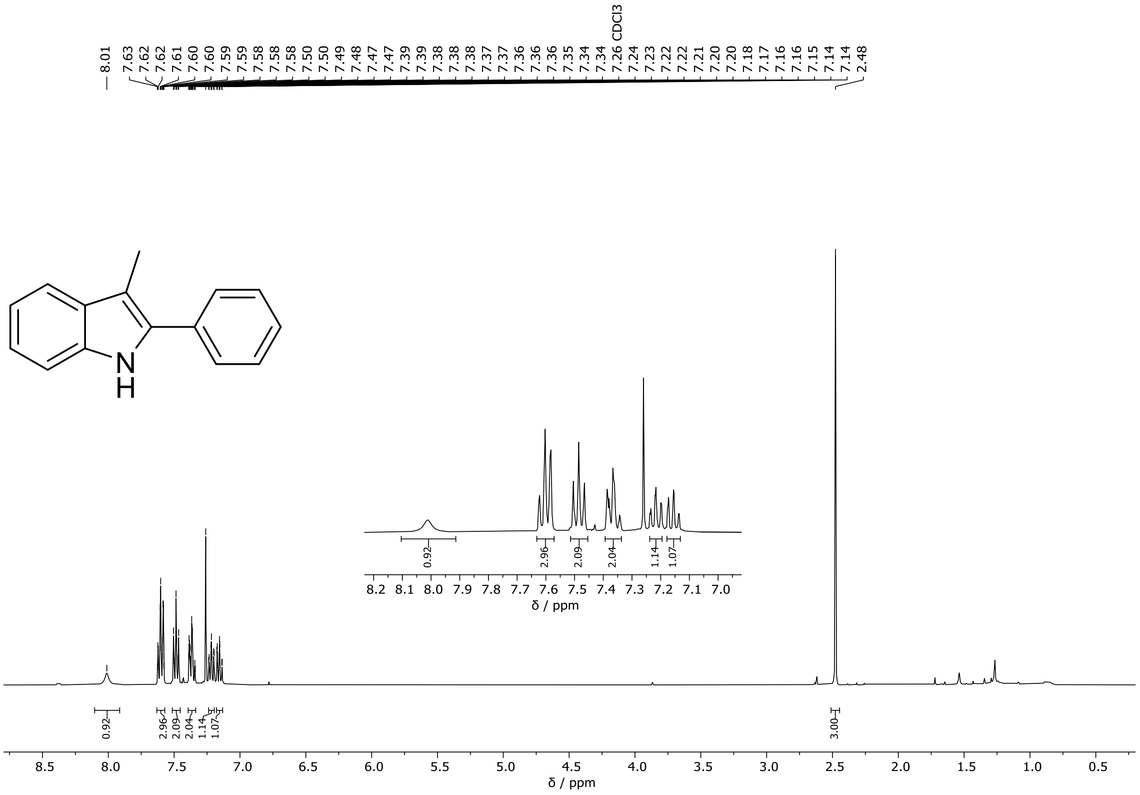

^{13}C-NMR (101 MHz, $CDCl_3$)

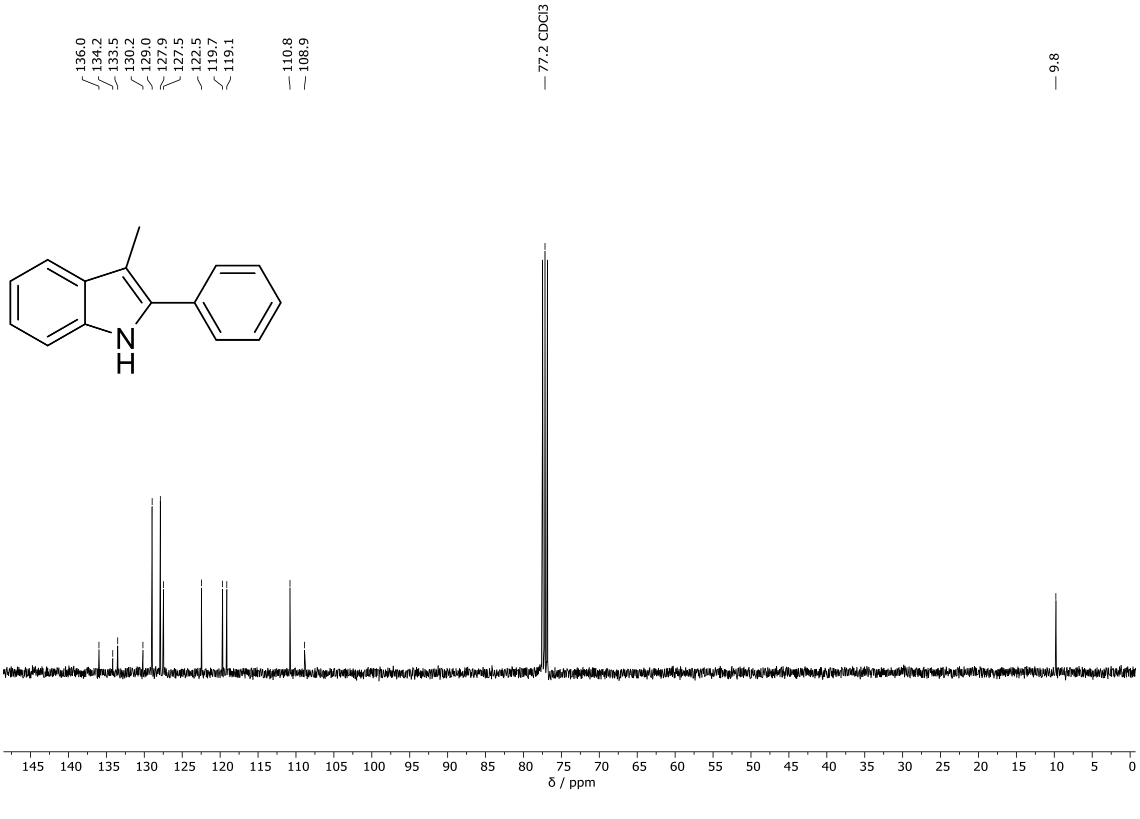

3-cyclopentyl-2,3-dihydro-1*H*-cyclopenta[*b*]quinoline (3al):

^{1}H-NMR (599 MHz, $CDCl_3$)

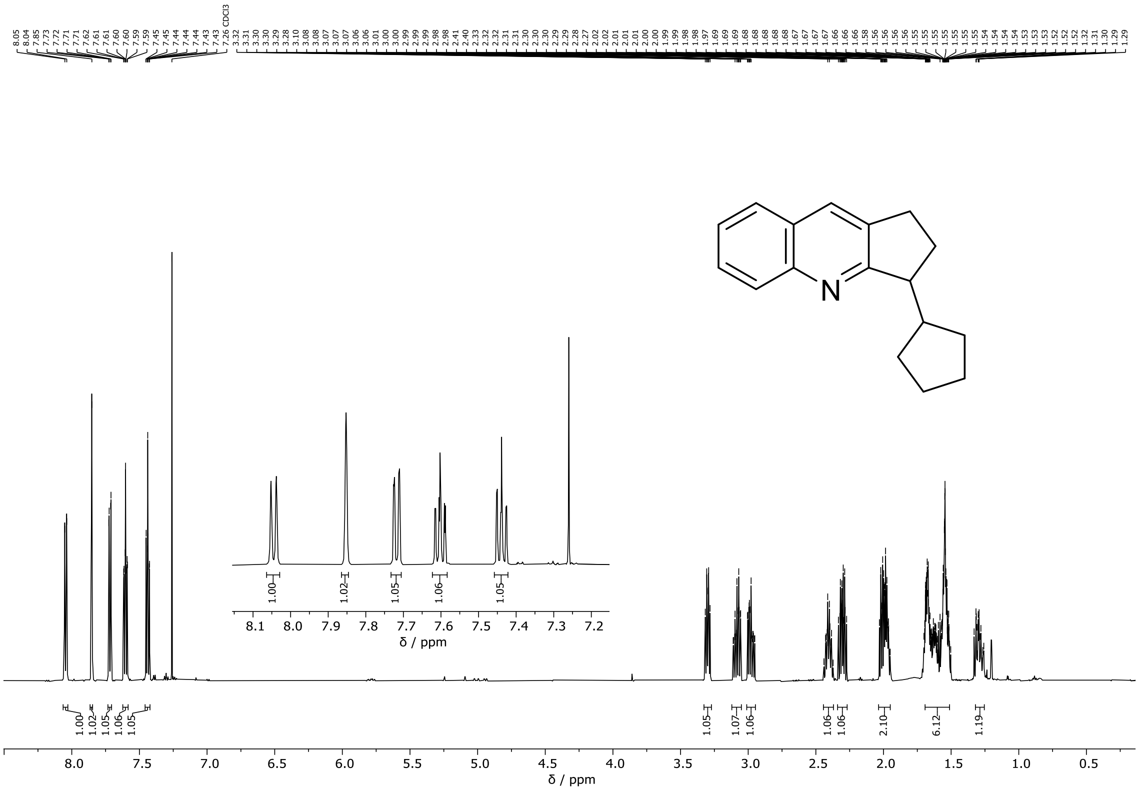

^{13}C-NMR (151 MHz, $CDCl_3$)

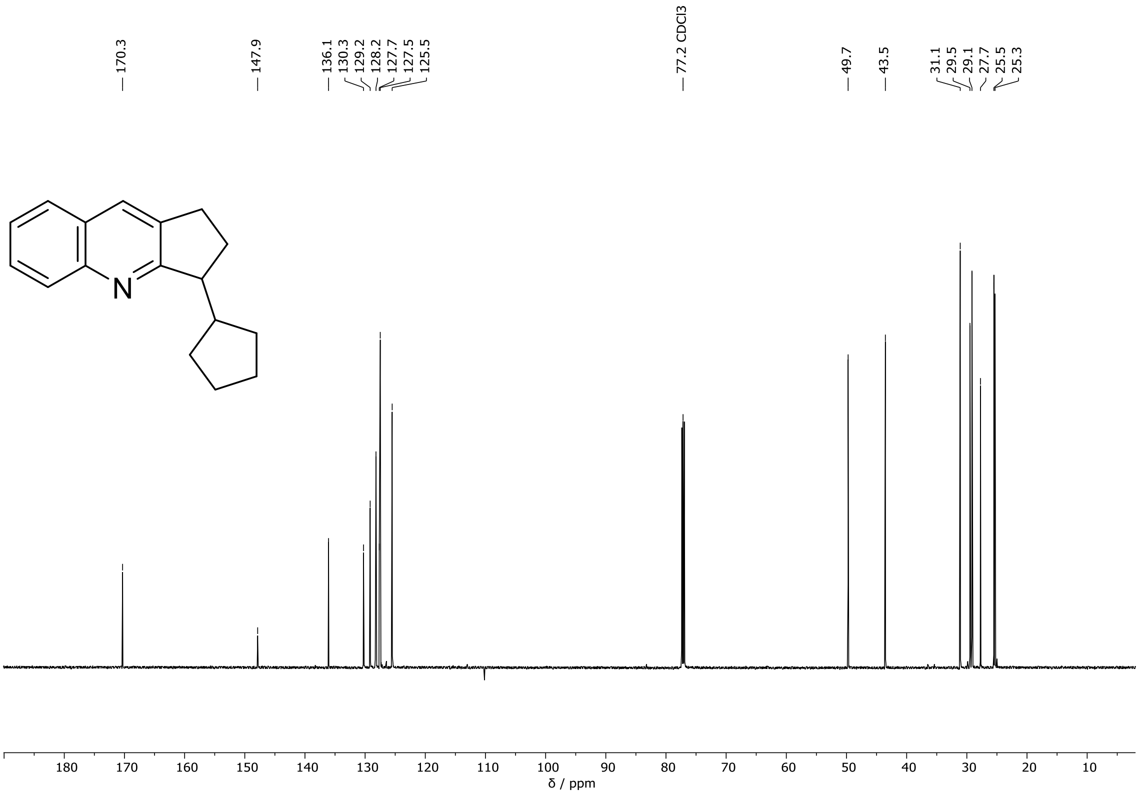

8. References

1. M. Christmann, *Org. Lett.,* 2025, **27**, 4–7.
2. R. Martínez, D. J. Ramón and M. Yus, *J. Org. Chem*., 2008, **73**, 9778–9780.
3. N. Marquise, G. Bretel, F. Lassagne, F. Chevallier, T. Roisnel, V. Dorcet, Y. S. Halauko, O. A. Ivashkevich, V. E. Matulis, P. C. Gros and F. Mongin, *RSC Adv*., 2014, **4**, 19602–19612.
4. E. M. Dauncey, S. P. Morcillo, J. J. Douglas, N. S. Sheikh and D. Leonori, *Angew. Chem. Int. Ed*., 2018, **57**, 744–748.
5. J. Zhang, C. Mück-Lichtenfeld and A. Studer, *Nature*, 2023, **619**, 506–513.
6. J. M. Anderson, N. D. Measom, J. A. Murphy and D. L. Poole*, Org. Lett*., 2023, **25**, 2053–2057.
7. K. Fobi and R. A. Bunce, *Molecules*, 2022, **27**, 4123.
8. P. Gangireddy, V. Patro, L. Lam, M. Morimoto and L. S. Liebeskind, *J. Org. Chem*., 2017, **82**, 3513–3529.
9. Z. Zhang, C. Liu, X. Li, T. Song, Z. Wu, X. Liang, Y. Zhao, X. Shen and H. Chen, *Eur. J. Med. Chem*., 2013, **60**, 410–420.
10. H. Naruto and H. Togo, *Org. Biomol. Chem*., 2019, **17**, 5760–5770.
11. Z. Zhang and H. Du, *Org. Lett*., 2015, **17**, 6266–6269.
12. A. M. Berman, J. C. Lewis, R. G. Bergman and J. A. Ellman, *J. Am. Chem. Soc*., 2008, **130**, 14926–14927.
13. J. Wei, H. Liang, C. Ni, R. Sheng and J. Hu, *Org. Lett*., 2019, **21**, 937–940.
14. B. Gao, Z. Han, W. Meng, X. Feng and H. Du, *J. Org. Chem*., 2023, **88**, 3335–3339.
15. L. Zhao, Y. Chen, C. Zhang, H. Chen, X. Zheng, W. Xue, J. Xu, H. Fu and R. Li, *J. Org. Chem*., 2025, **90**, 4959–4972.
16. J. Woo, C. Stein, A. H. Christian and M. D. Levin, *Nature*, 2023, **623**, 77–82.
17. X.-H. Hu and X.-P. Hu, *Org. Lett*., 2019, **21**, 10003–10006.
18. J. Xu, J. Sun, J. Zhao, B. Huang, X. Li and Y. Sun, *RSC Adv*., 2017, **7**, 36242–36245.
19. R. Rubio-Presa, S. Suárez-Pantiga, M. R. Pedrosa and R. Sanz, *Adv Synth Catal*, 2018, **360**, 2216–2220.
20. F. Zeng, B. Ma, Y. Wang, H. Li, M. Zhang and Z. Li, *Adv Synth Catal*, 2025, **367**, e202500222.
21. C. S. Cho and W. X. Ren, *J. Organomet. Chem*., 2007, **692**, 4182–4186.
22. X. Zhang, X. Ma, W. Qiu, J. Evans and W. Zhang, *Green Chem*., 2019, **21**, 349–354.
23. G. Zhang, J. Wu, H. Zeng, S. Zhang, Z. Yin and S. Zheng, *Org. Lett*., 2017, **19**, 1080–1083.
24. X. Zhang, J. Chen, S. Yong and Y. Zhao, *Tetrahedron Lett*., 2023, **128**, 154700.

25. Q. Ma, M. Li, Z. Chen, S.-F. Ni, J. S. Wright, L.-R. Wen and L.-B. Zhang, *Green Chem.*, 2022, **24**, 4425–4431.
26. J. Zhang, N. Spreckelmeyer, J. Lammert, M. Wiethoff, M. J. Milner, C. Mück-Lichtenfeld and A. Studer, *Angew. Chem. Int. Ed.*, 2025, **64**, e202502864.
27. D. Zhao, Z. Shi and F. Glorius, *Angew. Chem. Int. Ed.,* 2013, **52**, 12426–12429.
28. H. A. Houck, K. De Bruycker, C. Barner-Kowollik, J. M. Winne and F. E. Du Prez, *Macromolecules*, 2018, **51**, 3156–3164.
29. Q. Nguyen, T. Nguyen and T. G. Driver, *J. Am. Chem. Soc.*, 2013, **135**, 620–623.
30. Y.-D. Wu, J.-R. Ma, W.-M. Shu, K.-L. Zheng and A.-X. Wu, *Tetrahedron*, 2016, **72**, 4821–4826.
31. Q. Shi, H. Hu, M. Du, Y. Sun, Y. Li and Y. Li, *Org. Lett.*, 2023, **25**, 7100–7104.
32. A. Patra, S. Mukherjee, T. K. Das, S. Jain, R. G. Gonnade and A. T. Biju, *Angew. Chem. Int. Ed.,* 2017, **56**, 2730–2734.
33. A. P. Kozikowski, D. Ma, J. Brewer, S. Sun, E. Costa, E. Romeo and A. Guidotti, *J. Med. Chem.*, 1993, **36**, 2908–2920.
34. Z. Liu, M. Cai, F. Yin, Y. Li and H. Zhu, *Tetrahedron Lett.*, 2024, **144**, 155155.
35. D. Forberg, T. Schwob and R. Kempe, *Nat. Commun.*, 2018, **9**, 1751.
36. S. M. Barolo, A. E. Lukach and R. A. Rossi, *J. Org. Chem.*, 2003, **68**, 2807–2811.
37. H. Long, K. Xu, S. Chen, J. Lin, D. Wu, B. Wu, X. Tian and L. Ackermann, *Org. Lett.*, 2019, **21**, 3053–3056.
38. N. G. Connelly and W. E. Geiger, *Chem. Rev.*, 1996, **96**, 877–910.
39. S. Song, Y. Lai, Z. Tuo, J. Zhong and W. Zhou, *Angew. Chem. Int. Ed.*, 2023, **62**, e202305983.
40. J. Woo, T. Zeqiri, A. H. Christian, M. C. Ryan and M. D. Levin, *J. Am. Chem. Soc.*, 2025, **147**, 20120–20131.
41. L.-F. Dai, Y.-X. Jiang, D.-L. Yu, G.-Q. Sun, S.-S. Yan, W. Zhang, J.-H. Ye and D.-G. Yu, *ACS Catal.*, 2025, **15**, 7792–7799.
